# Phytochemical Investigation and Cytotoxic Activity of *Annona amazonica* (Annonaceae): Comprehensive Spectroscopic Characterization of *N*-Formylactinodaphnine

**DOI:** 10.3390/molecules31193431

**Published:** 2026-09-27

**Authors:** Jéssica Fernandes Auzier, Thayline Rayany Batista Menezes, Maria Lúcia Belém Pinheiro, Maria Victoria Lima de Castro, Milena Botelho Pereira Soares, Renato Sonchini Gonçalves, Josean Fechine Tavares, Daniel Pereira Bezerra, Emmanoel Vilaça Costa

**Affiliations:** 1 Postgraduate Program in Chemistry, Federal University of Amazonas (UFAM), Manaus 69080-900, AM, Brazil; jessicafauzier27@gmail.com; 2 Department of Chemistry, Federal University of Amazonas (UFAM), Manaus 69080-900, AM, Brazil; thaylinerayany4@gmail.com (T.R.B.M.); lbelem1@gmail.com (M.L.B.P.); 3 Gonçalo Moniz Institute, Oswaldo Cruz Foundation (IGM-FIOCRUZ/BA), Salvador 40296-710, BA, Brazil; mvictorialimac@gmail.com (M.V.L.d.C.); milena.soares@fiocruz.br (M.B.P.S.); 4 Department of Engineering and Exact Sciences, Setor Palotina, Federal University of Paraná (UFPR), Palotina 85950-000, PR, Brazil; renato.sonchini@ufpr.br; 5 Postgraduate Program in Natural and Synthetic Bioactive Products, Health Sciences Center, Federal University of Paraíba (UFPB), João Pessoa 58051-970, PB, Brazil; josean@ltf.ufpb.br

**Keywords:** *Annona amazonica*, aporphine and tetrahydroprotoberberine alkaloids, terpenoids, acanthoic acid, cytotoxicity, chemophenetic context

## Abstract

*Annona amazonica* R. E. Fries (Annonaceae) is a tropical species native to Central and South America whose phytochemical profile remains poorly investigated. In the present study, the bark of *A. amazonica* was subjected to phytochemical investigation using conventional chromatographic techniques, leading to the isolation and identification of 18 metabolites comprising a diterpene, sesquiterpenes, steroids, ketosteroids, and alkaloids. Attention was given to *N*-formylactinodaphnine (**16**), which was isolated and comprehensively characterized by high-resolution electrospray ionization mass spectrometry (HRESIMS), infrared spectroscopy (IR), and one- and two-dimensional nuclear magnetic resonance (1D and 2D NMR) spectroscopy. The present study provides a complete spectroscopic dataset for this compound and documents its occurrence in *A. amazonica*. The other isolated metabolites further expand the known phytochemical profile of the species. The effects of the isolated compounds on cellular viability were evaluated against HepG2, HCT116, MDA-MB-231, MCF-7, and U-87 MG human cancer cell lines and MRC-5 fibroblasts using the Alamar Blue assay after 72 h of incubation. Actinodaphnine (**14**) activity was detected across all five evaluated cancer cell lines, with IC_50_ values ranging from 5.59 to 11.73 μg/mL. *N*-Methylactinodaphnine (cassythicine) (**15**) had an IC_50_ value of 4.27 μg/mL against HCT116 cells; because of the limited amount of isolated material, compound **15** was evaluated only against HCT116 and MRC-5 cells. Overall, the results expand the phytochemical knowledge of *A. amazonica*, provide comprehensive spectroscopic documentation of *N*-formylactinodaphnine (**16**) from this species, and identify alkaloids with measurable in vitro activity in the cell-based viability assay.

## 1. Introduction

The genus *Annona* L. belongs to the family Annonaceae and comprises approximately 166–170 currently accepted species of trees, shrubs, and, less frequently, lianas [1,2]. It is among the best-known genera of the family, particularly because of the edible fruits of several species, including “graviola” (*Annona muricata* L.); “ata”, “pinha”, or “fruta-do-conde” (*Annona squamosa* L.); “cherimola” or “cherimoia” (*Annona cherimola* Mill.); and the hybrid “atemoya” (*A. cherimola* × *A. squamosa*). Vernacular names such as “pinha” and “araticum” are used to refer to other *Annona* species in Brazil. In addition to their edible fruits, several species have been traditionally used for medicinal purposes. Different plant parts have been prepared as decoctions, infusions, macerates, ointments, or medicinal baths for the treatment of conditions associated with bacterial and fungal infections, protozoal diseases, tumors, and insect bites, among other traditional applications [3,4].

Despite the economic and traditional importance of the genus, the phytochemical and biological investigation of *Annona* remains uneven, with research concentrated on a limited number of species, particularly *A. muricata*, *A. squamosa*, *A. reticulata* L., *A. cherimola*, *A. senegalensis* Pers., and *A. coriacea* Mart. [5,6,7,8,9,10]. The major classes of secondary metabolites reported from *Annona* species include alkaloids, annonaceous acetogenins, flavonoids, terpenoids, phenolic compounds, and cyclopeptides. These metabolites are associated with a broad range of biological activities, including cytotoxic, antimicrobial, antifungal, antiprotozoal, antimalarial, insecticidal, larvicidal, molluskicidal, anti-inflammatory, analgesic, antioxidant, neuropharmacological, immunosuppressive, and antidiabetic effects [5,6,7,8,9,10]. Thus, a considerable proportion of the genus remains relatively understudied, particularly with respect to species-specific phytochemical profiles and biological properties.

Among these understudied species is *Annona amazonica* R. E. Fries (*syn. Annona amazonica* var. *lancifolia* R. E. Fries), a tropical tree popularly known in the Brazilian Amazon as “envira”, “araticum”, “araticum árvore gigante”, and “ata”. The species can reach 20–30 m in height and is distinguished from other *Annona* species by its very small flowers, congenitally fused outer and inner petals, and glaucous fruits. It is distributed from Costa Rica to northern Brazil, where its fruits are traditionally consumed by local populations [11,12,13]. Previous phytochemical studies of *A. amazonica* have reported the presence of cyanogenic compounds in the leaves, seeds, and fruits [14]. Pinheiro et al. [15] reported the isolation of high yields of acanthoic acid from the stem and evaluated its trypanocidal activity. Alcantara et al. [16] subsequently identified (*E*)-caryophyllene and linalool as major constituents of essential oils obtained from leaves and branches, respectively. Castro et al. [17] further investigated the chemical composition and biological activity of essential oil from *A. amazonica* leaves. The major constituents were (*E*)-caryophyllene (32.01%), 1,8-cineole (13.93%), α-copaene (7.77%), α-humulene (7.15%), and α-pinene (5.13%). In vitro, the essential oil reduced the viability of HepG2 cells, with experimental findings including DNA fragmentation, cell shrinkage, and a reduction in mitochondrial membrane potential. In an in vivo model, treatment with the essential oil was associated with a 39.2% reduction in tumor growth [17]. These findings indicate the biological activity of the essential oil, but the specific mechanisms underlying these effects were not investigated in that study.

As part of our continuing phytochemical investigation of Amazonian Annonaceae species, the present study describes the chemical constituents isolated from the bark of *A. amazonica* and evaluates selected isolated metabolites in a cell-based viability assay. To the best of our knowledge, no previous phytochemical study has specifically investigated the bark of this species. This study expands the documented phytochemical profile of *A. amazonica* and provides a comprehensive spectroscopic characterization of *N*-formylactinodaphnine (**16**), which was isolated during the investigation. In addition, the effects of selected isolated metabolites on the viability of a panel of human cancer cell lines and MRC-5 fibroblasts were evaluated.

## 2. Results and Discussion

### 2.1. Structural Elucidation and Identification of the Isolated Compounds

A phytochemical investigation of the bark of *A. amazonica* led to the isolation of eighteen compounds (**1**–**18**), comprising a diterpene (**1**), sesquiterpenes (**2** and **9**), steroids (**3**–**5**), ketosteroids (**6**–**8**), aporphine alkaloids (**10**–**16**), and tetrahydroprotoberberine alkaloids (**17** and **18**) (Figures S1–S273). Compound **16** was identified as *N*-formylactinodaphnine. The name *N*-formylactinodaphnine had previously been used by Lee et al. [18] for a neutral product generated by deliberate *N*-formylation of an alkaloid fraction containing actinodaphnine with ethyl formate in DMF at 90 °C for 1 day. Although that study referred to the resulting product as *N*-formylactinodaphnine, it did not report its isolation from a plant source or provide independent structural characterization of the derivative, including molecular formula, mass spectrometric data, or NMR spectroscopic data. Therefore, the present study does not claim the first use of the name *N*-formylactinodaphnine or the first chemical generation of this derivative. Rather, the contribution of the present study is the isolation of **16** as a natural product from *A. amazonica* bark and its comprehensive spectroscopic characterization.

Compounds **1**–**15** and **17**–**18** were identified by spectroscopic analysis (Figures S1–S273) and comparison with published data. The structures of all the isolated compounds are shown in Figure 1. Compound **16** was obtained as a brown, amorphous solid. Its HRESIMS spectrum showed a protonated molecular ion [M + H]^+^ at *m*/*z* 340.1177 (calcd. for C_19_H_18_NO_5_^+^, *m*/*z* 340.1185; Δ = −2.3 ppm), which is consistent with the molecular formula C_19_H_17_NO_5_. The IR spectrum showed absorptions at 3271 and 1651 cm^−1^, which are consistent with a phenolic hydroxyl group and an amide-type carbonyl functionality, respectively [19,20,21].

The ^1^H NMR spectrum of **16** (CDCl_3_, 500 MHz) displayed duplicated resonances for several signals, including two formyl-proton singlets at δ_H_ 8.25 and 8.38 and duplicated aliphatic resonances adjacent to nitrogen. These features indicate the presence of two distinguishable NMR environments in solution and are consistent with the restricted rotation of the *N*-formyl amide bond [19,20,21]. Because variable-temperature NMR and EXSY experiments were not performed, direct exchange between the two states was not demonstrated.

Apart from the duplicated resonances, the NMR data were consistent with an oxygenated aporphine skeleton. Each conformational state contains 19 chemically distinct carbon positions; consequently, more than 19 ^13^C NMR resonances may be observed when corresponding signals from the two states are resolved separately. Three aromatic singlets, a methylenedioxy group, and a methoxy group further supported the highly substituted aporphine framework summarized in Table 1.

The HSQC correlation map allowed assignment of the protonated carbons, whereas the ^1^H–^1^H COSY correlation map established the spin systems corresponding to H-4/H-5 and H-6a/H-7. The location of the methylenedioxy bridge between C-1 and C-2 was supported by HMBC correlations from the -OCH_2_O- protons (δ_H_ 5.97 and 6.10) to C-1 (δ_C_ 142.5) and C-2 (δ_C_ 147.1). The methoxy group was assigned to C-10 on the basis of the three-bond HMBC correlation between the methoxy protons (δ_H_ 3.93) and C-10 (δ_C_ 145.6). Long-range HMBC correlations from H-3 to C-1, C-2, C-3b, and C-4 (Table 1 and Figure 2), together with correlations from H-11 to C-1a, C-7a, and C-9 and from H-8 to C-7, C-10, and C-11a (Table 1 and Figure 2), established the substitution pattern of the aromatic rings, which was consistent with that reported for actinodaphnine (**14**).

The formyl functionality was supported by the molecular formula derived from HRESIMS, the IR absorption at 1651 cm^−1^, the characteristic formyl-proton resonances at δ_H_ 8.25 and 8.38, and their corresponding one-bond HSQC correlations with carbonyl carbon resonances at δ_C_ 162.35 and 162.30, respectively. These HSQC correlations establish the presence of two HCO units but do not, by themselves, establish their attachment to nitrogen. Additional support for the *N*-formyl assignment was provided by the HMBC correlations from the formyl proton at δ_H_ 8.25, assigned to the major state, to C-5 (δ_C_ 42.4) and C-6a (δ_C_ 49.9), and from the formyl proton at δ_H_ 8.38, assigned to the minor state, to C-5 (δ_C_ 36.4) and C-6a (δ_C_ 53.7). Together with the duplicated formyl resonances, these correlations support the presence of two conformational states of the *N*-formyl derivative in solution (Figure 2).

The assignment of **16** as *N*-formylactinodaphnine was therefore based on the combined spectroscopic evidence, including its molecular formula, amide-type carbonyl resonance, aromatic substitution pattern consistent with that of actinodaphnine (**14**), and changes in the resonances of the nitrogen-containing tetrahydroisoquinoline moiety.

Relative integration of the two resolved formyl-proton signals gave normalized integrals of 1.000 for δ_H_ 8.25 and 0.436 for δ_H_ 8.38, corresponding to an approximate population ratio of 69.6:30.4. This ratio is reported as approximately 70:30 because it was obtained by direct integration of a single 500 MHz ^1^H NMR spectrum rather than from replicate quantitative NMR measurements; consequently, no formal statistical uncertainty was assigned. The major state was tentatively assigned as *Z* on the basis of the NOESY (Figure 2) correlation between δ_H_ 8.25 (CHO) and 3.80 (H-5), whereas the minor state was tentatively assigned as *E* on the basis of correlations between δ_H_ 8.38 (CHO) and 4.58 (H-6a) and between δ_H_ 8.38 (CHO) and 2.74 (H-7). These NOESY correlations support the proposed spatial assignments but do not establish the rate or occurrence of chemical exchange between the two states (Figure 2).

Restricted rotation about the amide C–*N* bond provides a plausible explanation for the duplicated NMR environments observed for **16** and is consistent with previous observations for *N*-formyl aporphines [19,20,21]. Accordingly, the duplicated resonances are interpreted as being consistent with *Z*/*E* amide rotamers in solution rather than as definitive evidence of slow interconversion between them. The absence of variable-temperature NMR or EXSY data prevents further characterization of the exchange process under the conditions investigated.

Taken together, the HRESIMS, IR, HSQC, COSY, HMBC, and NOESY data support the identification of compound **16** as *N*-formylactinodaphnine. The assignment is based on the overall spectroscopic dataset and is not dependent on the formyl H/C relationship observed in the HMBC correlation map. Lee et al. [18] previously used the name *N*-formylactinodaphnine for a chemically generated derivative but did not report its isolation as a natural product or provide independent spectroscopic characterization of that material. The present study therefore documents the isolation of **16** from *A. amazonica* bark and provides its comprehensive spectroscopic characterization.

Importantly, the absolute configuration at C-6a of compound **16** was not established in the present study; therefore, the structural assignment is limited to the connectivity and relative stereochemical/conformational features supported by the spectroscopic data, without implying an experimentally established absolute configuration.

The remaining isolated compounds were identified as acanthoic acid (**1**) [15], caryophyllene oxide (**2**) [22], β-sitosterol (**3**) [23,24], stigmasterol (**4**) [23], campesterol (**5**) [24], β-sitostenone (**6**) [25], stigmasta-4,22-dien-3-one (**7**) [25], campest-4-en-3-one (**8**) [25], cadinane-4β,5α,10β-triol (**9**) [26], liriodenine (**10**) [27], lysicamine (**11**) [28], anonaine (**12**) [29], nornuciferine (**13**) [30], actinodaphnine (**14**) [31], *N*-methylactinodaphnine (cassythicine) (**15**) [15], discretamine (**17**) [29], and 10-demethyldiscretine (**18**) [32]. Their identities were established by spectroscopic analysis (Figures S1–S273) and comparison with the corresponding literature data. Table 1 and Table 2 summarize the NMR assignments obtained for the compounds analyzed in the present study. These tables are intended to document the assignments used for the isolated compounds and are not presented as systematic reviews of previously published NMR assignments; discrepancies with literature data are discussed where applicable.

Acanthoic acid (**1**) was the predominant diterpenoid recovered during the processing of the hexane extract. From the 30.0-g portion subjected to the isolation procedure, 12.7178 g of compound **1** was obtained after the initial recrystallization, followed by an additional 5.055 g from subsequent chromatographic purification and 117.5 mg from a purified subfraction. The combined amount of purified acanthoic acid obtained from these isolation steps was therefore 17.8903 g. Because only 30.0 g of the 37.02 g crude hexane extract was subjected to this isolation workflow, this value represents recovered purified material from the processed portion and should not be interpreted as an estimate of the total acanthoic acid content of the crude extract.

### 2.2. Chemophenetic (Chemotaxonomic) Context

The present phytochemical investigation expands the documented chemical profile of *A. amazonica* by recording the occurrence of aporphine alkaloids, a diterpene, sesquiterpenoids, steroids, and ketosteroids in the analyzed bark sample. Aporphine alkaloids are widely distributed in the genus *Annona* and throughout the family Annonaceae and therefore provide a relevant genus- and family-level phytochemical context. However, their broad distribution limits their value as species-specific chemophenetic (chemotaxonomic) markers [5,32,33,34]. Because the present study was based on a single collection and employed selective extraction, fractionation, and isolation procedures, the frequency with which individual metabolites were recovered should not be interpreted as a quantitative measure of their relative abundance in the plant material.

Before the present study, the reported chemical constituents of *A. amazonica* included acanthoic acid (**1**), caryophyllene oxide (**2**), β-sitosterol (**3**), stigmasterol (**4**), liriodenine (**10**), and *N*-methylactinodaphnine (cassythicine) (**15**) [15]. In the present investigation, campesterol (**5**), β-sitostenone (**6**), stigmasta-4,22-dien-3-one (**7**), campest-4-en-3-one (**8**), cadinane-4β,5α,10β-triol (**9**), lysicamine (**11**), anonaine (**12**), nornuciferine (**13**), actinodaphnine (**14**), *N*-formylactinodaphnine (**16**), discretamine (**17**), and 10-demethyldiscretine (**18**) were identified in *A. amazonica* for the first time. These findings expand the documented distribution of these metabolites within the species and, except for the characterization of **16** discussed above, do not imply structural novelty.

Actinodaphnine (**14**), *N*-methylactinodaphnine (cassythicine) (**15**), and *N*-formylactinodaphnine (**16**) constitute a closely related structural series. Their co-occurrence in *A. amazonica* is consistent with the established biosynthetic framework of aporphine alkaloids, which are generally derived from benzylisoquinoline precursors, particularly reticuline, through oxidative coupling and subsequent transformations leading to the aporphine skeleton [35,36,37]. The structural similarity among **14**–**16** is compatible with possible metabolic transformations involving *N*-demethylation and *N*-formylation. However, the present data do not establish a specific precursor–product relationship or the sequence of these transformations. Thus, their co-occurrence is considered suggestive of a possible biosynthetic connection rather than evidence of a defined metabolic pathway.

Acanthoic acid (**1**), an *ent*-pimarane-type diterpenoid, was the predominant diterpenoid recovered during the processing of the hexane extract. Its substantial recovery was associated with the formation of abundant crystalline material during extraction and purification. Nevertheless, because the isolation procedure was applied to a single 30.0-g portion of the crude hexane extract and involved selective fractionation and recrystallization, the recovered amount should be regarded as an isolation yield from the processed material rather than as a quantitative estimate of its abundance in the plant or crude extract. The occurrence of acanthoic acid further highlights the presence of *ent*-pimarane-type diterpenoids in *A. amazonica*, but the present data do not provide sufficient evidence for broader chemophenetic (chemotaxonomic) or evolutionary interpretations.

The isolation of cadinane-4β,5α,10β-triol (**9**) further expands the documented sesquiterpenoid profile of *A. amazonica*. However, its occurrence in a single sample is insufficient to establish its value as a chemophenetic marker. Such an assessment would require comparative data from multiple specimens, populations, and related species, preferably obtained under standardized analytical conditions.

Overall, the isolated-compound profile broadens the documented phytochemical knowledge of *A. amazonica*. The present findings provide useful distributional and structural information but do not support the assignment of a distinctive species-level chemophenetic position or conclusions regarding evolutionary diversification. Such interpretations would require replicated sampling, comparative metabolomic or phytochemical profiling, appropriate phylogenetic context, and statistical chemophenetic analyses.

### 2.3. Cytotoxicity Assay

The hexane and methanolic extracts, together with the neutral (NCP) and alkaloid-rich (ACP) phases obtained by acid–base fractionation of the methanolic extract, were initially screened at 50 μg/mL against HepG2 and HCT116 cells (Table 3). Among the tested samples, the alkaloid-rich phase showed the highest inhibition values, reaching 55.81% against HepG2 cells and 63.32% against HCT116 cells, and was therefore selected for subsequent phytochemical investigation. This workflow is considered bioactivity-informed selection rather than full bioassay-guided fractionation because the chromatographic subfractions were not subjected to iterative biological testing.

Chromatographic separation of the alkaloid-rich phase afforded compounds **10**–**18**. Compounds **14**–**18** were subsequently evaluated against HepG2, HCT116, MDA-MB-231, MCF-7, U-87 MG, and MRC-5 cells (Table 4). Compounds **10**–**13** were not evaluated in the present study because their effects on cellular viability were previously investigated by our research group [38,39,40,41,42]. Accordingly, the present assays focused on compounds for which new biological data were generated. Acanthoic acid (**1**), recovered predominantly from the processed hexane fraction, was also included in the evaluation.

Among the isolated compounds tested, actinodaphnine (**14**) showed measurable activity against all five cancer cell lines, with IC_50_ values ranging from 5.59 to 11.73 μg/mL. Its IC_50_ value against MRC-5 fibroblasts was 6.84 μg/mL, resulting in selectivity indices ranging from 0.58 to 1.22 and indicating limited selectivity toward the cancer cell lines under the conditions tested. *N*-Methylactinodaphnine (cassythicine) (**15**) had an IC_50_ value of 4.27 μg/mL against HCT116 cells and 12.16 μg/mL against MRC-5 fibroblasts. Because of the limited amount of isolated material, compound **15** was not evaluated against the other cancer cell lines. *N*-Formylactinodaphnine (**16**) showed relatively low activity, with IC_50_ values of 21.07 and 22.69 μg/mL against HCT116 and MDA-MB-231 cells, respectively, and IC_50_ values above 25 μg/mL against the other cancer cell lines tested.

The activity profiles of compounds **14**–**16** provide descriptive information for this structurally related aporphinoid series. However, because these compounds were not evaluated against identical cell panels and several IC_50_ values were close to or above the highest concentration tested, the present data do not allow a reliable assessment of the effect of *N*-substitution on cellular activity or the establishment of a definitive structure–activity relationship.

Discretamine (**17**) and 10-demethyldiscretine (**18**) generally showed weak cytotoxic activity against the tested cell lines. Compound **18** exhibited an IC_50_ value of 22.08 μg/mL against HCT116, whereas for the other cases in which the IC_50_ exceeded the highest concentration tested, 50% inhibition was not reached within the tested concentration range. Acanthoic acid (**1**) showed measurable activity against HepG2 and HCT116 cells under the conditions employed. Because the activity of the crude hexane extract and that of the isolated compound were determined in different experimental contexts, the present data do not establish a direct relationship between the activity of acanthoic acid and that of the crude extract.

The activity observed for actinodaphnine (**14**) is consistent with previous reports. Stévigny et al. [31] reported the cytotoxic activity of actinodaphnine (**14**) against human melanoma (Mel-5) and promyelocytic leukemia (HL-60) cells. Hsieh et al. [43] subsequently reported the effects of actinodaphnine (**14**) in Mahlavu human hepatoma cells, including the induction of apoptosis, increased reactive oxygen species (ROS) and nitric oxide (NO) production, mitochondrial membrane depolarization, caspase-3/7 activation, and the downregulation of NF-κB signaling. These findings provide a mechanistic context for the previously reported activity of **14**.

The activity observed for the alkaloid-rich phase cannot be attributed to a specific constituent or to additive or synergistic interactions on the basis of the present data. The observed inhibition may therefore reflect the activity of one or more constituents present in the tested phase, including compounds that were isolated, compounds not evaluated in the present assays, minor constituents, or unidentified metabolites. Dedicated combination experiments and quantitative compositional analyses are needed to assess potential pharmacological interactions.

## 3. Materials and Methods

### 3.1. General Experimental Procedures

Fourier transform infrared (FTIR) spectra were recorded on an IRSpirit FTIR spectrophotometer (Shimadzu, Kyoto, Japan). Optical rotations were measured in CHCl_3_ using a Jasco P-2000 polarimeter (Jasco, Tokyo, Japan) at the sodium D line (589 nm).

One- and two-dimensional NMR spectra were acquired in CDCl_3_ (chloroform-*d*), CD_3_OD (methanol-*d*_4_), or mixtures of these solvents at 298 K using AVANCE III HD and AVANCE NEO NMR spectrometers (Bruker, Billerica, MA, USA) operating at 11.75 and 14.1 T, corresponding to ^1^H frequencies of 500 and 600 MHz and ^13^C frequencies of 125 and 150 MHz, respectively. Chemical shifts (δ) are reported in ppm relative to the tetramethylsilane signal at 0.00 ppm as an internal reference, and coupling constants (*J*) are given in Hz. The NMR spectrometers were equipped with 5-mm multinuclear inverse-detection probes with *z* gradients for 1D and 2D NMR experiments. HSQC and HMBC experiments were optimized for average ^1^*J*(C,H) and long-range *^n^J*(C,H) coupling constants of 140 and 8 Hz, respectively.

For low-resolution mass spectrometry (LR-MS), the isolated compounds were dissolved in HPLC-grade methanol to prepare stock solutions at 1 mg/mL. Aliquots (5 µL) of the stock solutions were further diluted to 5 µg/mL and analyzed by direct infusion using a TSQ Quantum Access triple quadrupole mass spectrometer (Thermo Scientific, San Jose, CA, USA) equipped with electrospray ionization (ESI) or atmospheric pressure chemical ionization (APCI) sources operated in positive or negative ion mode.

High-resolution mass spectra were acquired using a Shimadzu HPLC system (Shimadzu, Kyoto, Japan) coupled to a micrOTOF mass spectrometer (Bruker Daltonics, Billerica, MA, USA) equipped with an electrospray ionization (ESI) source operated in positive mode. The instrumental parameters were as follows: capillary voltage, 4.5 kV; end plate offset, −500 V; nebulizer pressure, 3.9 bar; dry gas (N_2_) flow rate, 8.0 L/min; and dry gas temperature, 200 °C. Mass spectra were acquired over an *m*/*z* range of 50–1500.

Silica gel 60 (Sigma–Aldrich, San Luis, MO, USA; 70–230 mesh) was used for column chromatography (CC). Silica gel 60 F_254_ plates (Macherey-Nagel, Düren, Germany; 0.25 mm, aluminum-backed) were used for analytical TLC, whereas silica gel 60 F_254_ plates (Macherey-Nagel; 1.00 mm, glass-backed) were used for preparative TLC (PTLC). Compounds were visualized under UV light at 254 and 365 nm, followed, when needed, by spraying with *p*-anisaldehyde reagent and heating on a hot plate or by spraying with Dragendorff’s reagent.

Gas chromatography–mass spectrometry (GC–MS) analyses were performed using a Trace Ultra gas chromatograph coupled to an ISQ single-quadrupole mass spectrometer (Thermo Scientific, Waltham, MA, USA). The system was equipped with a TriPlus autosampler and a TR-5MS fused-silica capillary column (30 m × 0.25 mm i.d., 0.25 µm film thickness). Helium (99.999% purity) was used as the carrier gas at a constant flow rate of 1.2 mL/min. The GC oven temperature was programmed from 120 °C, held for 2 min, to 320 °C at 20 °C/min and then held at 320 °C for 10 min, resulting in a total analysis time of 22 min. Samples were dissolved in dichloromethane (CH_2_Cl_2_), and 0.5 µL was injected in split mode at a split ratio of 1:50. The injector temperature was maintained at 250 °C, and the GC–MS transfer line temperature was 280 °C. Mass spectra were acquired in full-scan mode using electron ionization (EI) at 70 eV over an *m*/*z* range of 40–600 with a scan time of 0.5 s.

### 3.2. Plant Material

Bark from a single individual of *A. amazonica* was collected on 3 December 2021, at the Adolpho Ducke Reserve (02°54′49.9″ S, 59°58′43.6″ W), Manaus, Amazonas State, Brazil. The botanical material was identified by Prof. Dr. Antonio Carlos Webber, a plant taxonomist at the Department of Biology of the Universidade Federal do Amazonas (DB/UFAM). A voucher sample (No. 12098) was deposited at the DB/UFAM Herbarium. Access to the genetic heritage was registered in the *Sistema Nacional de Gestão do Patrimônio Genético e do Conhecimento Tradicional Associado* (SISGEN) under record A70EDCD.

### 3.3. Preparation of NaHCO_3_-Treated Silica Gel

Silica gel for column chromatography (0.063–0.200 mm) was treated with a 10% aqueous solution of sodium bicarbonate (NaHCO_3_). Briefly, a 10% NaHCO_3_ solution was prepared by dissolving 100 g of sodium bicarbonate in distilled water and adjusting the final volume to 1 L. Silica gel (500 g) was suspended in the NaHCO_3_ solution until completely immersed and maintained under periodic stirring for 1 h, followed by standing for 24 h at room temperature.

The treated silica gel was collected by filtration under reduced pressure, transferred to a porcelain dish, and dried at 200 °C with occasional stirring until a free-flowing powder was obtained. The resulting NaHCO_3_-treated silica gel was used as the stationary phase for column chromatography.

### 3.4. Extraction and Isolation

The bark of *A. amazonica* was initially air-dried at room temperature for 24 h and subsequently dried in a forced-air circulation oven at 40 °C for 48 h. The dried material was pulverized using a four-knife mill grinder (Marconi, Piracicaba, SP, Brazil), yielding 1621.5 g of powdered bark. The powdered material was exhaustively extracted by maceration with hexane (6 × 3.5 L), followed by methanol (MeOH; 6 × 3.5 L). The resulting extracts were concentrated under reduced pressure at 40–50 °C using a rotary evaporator, yielding 37.02 g of hexane extract and 177.94 g of MeOH extract.

The hexane extract underwent abundant crystallization after solvent removal. Because of the large amount of crystalline material formed, a 30.0-g portion of the hexane extract was subjected to successive washings with hexane, followed by recrystallization from a hexane–CH_2_Cl_2_ mixture (4:1, *v*/*v*), yielding compound **1** (12,717.8 mg) as a white crystalline solid and a mother-liquor-enriched fraction (17,301.6 mg). Because this enriched fraction still contained a high proportion of compound **1**, a 12.0 g aliquot was subjected to silica gel column chromatography (6.0 × 60.0 cm, i.d. × height). Elution was performed successively with hexane (100%), hexane–CH_2_Cl_2_ (80:20, 70:30, 50:50, 30:70, and 20:80, *v*/*v*), CH_2_Cl_2_ (100%), CH_2_Cl_2_–EtOAc (80:20, 70:30, 50:50, 30:70, and 20:80, *v*/*v*), EtOAc (100%), and EtOAc–MeOH (90:10, *v*/*v*). Fractions of 500 mL were collected throughout the chromatographic separation, affording fourteen pooled fractions (F1–F14).

Fraction F3 (1741.5 mg), eluted with hexane–CH_2_Cl_2_ (70:30, *v*/*v*), and fraction F4 (3991.1 mg), eluted with hexane–CH_2_Cl_2_ (50:50, *v*/*v*), consisted of a white crystalline solid containing a small amount of colorless oil. These fractions were combined and subjected to washing with hexane, followed by recrystallization from hexane–CH_2_Cl_2_ (4:1, *v*/*v*), affording additional compound **1** (5.055 g) and an enriched fraction (620.0 mg). Approximately 150.0 mg of this enriched fraction was purified by preparative TLC using hexane–EtOAc (90:10, *v*/*v*) with two consecutive developments, yielding compounds **1** (117.5 mg) and **2** (12.7 mg).

Fraction F5 (2699.7 mg), eluted with hexane–CH_2_Cl_2_ (30:70, *v*/*v*), was obtained as a white crystalline solid similar in appearance to compound **1**. Fraction F7 (479.9 mg), eluted with CH_2_Cl_2_ (100%), was purified by preparative TLC using hexane–EtOAc (80:20, *v*/*v*) with two consecutive developments, affording a mixture of compounds **3**–**5** (14.0 mg) and a mixture of compounds **6**–**8** (12.7 mg). Fraction F11 (209.6 mg), eluted with CH_2_Cl_2_–EtOAc (30:70, *v*/*v*), was further purified by preparative TLC using CHCl_3_–MeOH (90:10, *v*/*v*) with two consecutive developments, affording compound **9** (5.3 mg).

TLC analysis after spraying with Dragendorff’s reagent indicated a high abundance of nitrogen-containing compounds in the MeOH extract, suggesting the presence of alkaloids. An aliquot of the MeOH extract (167.26 g) was therefore subjected to acid–base extraction. The aliquot was transferred to an Erlenmeyer flask and treated with 300 mL of CHCl_3_, resulting in a heterogeneous suspension containing CHCl_3_-soluble material and an insoluble fraction weighing approximately 100 g. The insoluble fraction was retained in the system during the subsequent acid extraction and was neither separately collected nor discarded at this stage.

The entire suspension was subjected to acid–base partitioning at approximately 25 °C over a total period of 48 h. The suspension was initially extracted with 3 L of 3% (*v*/*v*) aqueous HCl using 300-mL portions. During acid extraction, the insoluble fraction remained in the extraction system and was exposed to the acidic medium together with the extract. After each extraction, the system was allowed to stand until complete phase separation was achieved. The acidic aqueous phase was then carefully separated from the chloroform phase and from the insoluble material remaining in the system. The insoluble fraction, approximately 100 g, was discarded only after the acid extraction and separation of the acidic aqueous phase were completed.

The combined organic phase afforded the neutral chloroform phase (NCP, 11.38 g), whereas the combined acidic aqueous phase constituted the acidic aqueous phase (AAP). The AAP (pH 3.0) was subsequently basified with NH_4_OH to a pH of 11.0. The resulting basic aqueous phase was exhaustively extracted with CHCl_3_ (4 L total) using 300-mL portions. The extraction was continued until no positive response was observed in the Dragendorff test for the aqueous phase under the experimental conditions employed. The combined CHCl_3_ extracts afforded an alkaloid-rich phase (ACP, 2.37 g), whereas the remaining aqueous phase was designated the basic aqueous phase (BAP). Although the BAP contained a relatively large amount of material, this material was predominantly highly water-soluble and was not amenable to the chromatographic procedures employed for the subsequent phytochemical investigation. Therefore, the BAP was not further investigated. The ACP was concentrated under reduced pressure and subsequently subjected to chromatographic separation and phytochemical investigation.

A portion of the alkaloid-rich phase (ACP, 2.30 g) was subjected to column chromatography on silica gel (3.0 × 60 cm, i.d. × height) previously treated with 10% aqueous NaHCO_3_, as described in Section 3.3 (*Preparation of NaHCO_3_-Treated Silica Gel*). Elution was performed successively with hexane (100%); hexane–CH_2_Cl_2_ (90:10, 80:20, 70:30, 60:40, 50:50, 40:60, 30:70, 20:80, and 10:90, *v*/*v*); CH_2_Cl_2_ (100%); CH_2_Cl_2_–EtOAc (90:10, 80:20, 70:30, 60:40, 50:50, 40:60, 30:70, 20:80, and 10:90, *v*/*v*); EtOAc (100%); EtOAc–MeOH (95:5, 90:10, 85:15, 80:20, 75:25, 70:30, 60:40, and 50:50, *v*/*v*); and finally MeOH (100%). Approximately 200 mL of each solvent system was used, and fractions were collected in 30-mL vials, yielding 157 fractions in total. For selected solvent gradients, additional volumes of 100 mL or more were used when necessary to ensure complete elution of the constituents from the column. Fractions were monitored by TLC using CH_2_Cl_2_–MeOH (95:5, 90:10, 85:15, and 80:20, *v*/*v*) as the development system. On the basis of their chromatographic profiles, similar fractions were combined to afford sixteen pooled fractions (FA1–FA16).

Fraction FA3 (53.0 mg), eluted with hexane–CH_2_Cl_2_ (40:60 and 30:70, *v*/*v*), was purified by preparative TLC using CH_2_Cl_2_–MeOH (97:3, *v*/*v*) with three consecutive developments, yielding compounds **10** (4.7 mg), **11** (4.4 mg), and **12** (1.8 mg).

Fraction FA4 (28.1 mg), eluted with hexane–CH_2_Cl_2_ (10:90, *v*/*v*) and CH_2_Cl_2_ (100%), and FA5 (23.9 mg), eluted with CH_2_Cl_2_ (100%) and CH_2_Cl_2_–EtOAc (90:10, *v*/*v*), exhibited similar TLC profiles and were therefore combined (52.0 mg). The resulting fraction was purified by preparative TLC using CH_2_Cl_2_–MeOH (97:3, *v*/*v*) with three consecutive developments, affording compounds **10** (4.4 mg) and **13** (2.1 mg).

Fraction FA6 (48.6 mg), eluted with CH_2_Cl_2_–EtOAc (90:10, *v*/*v*), was purified by preparative TLC using CH_2_Cl_2_–MeOH (97:3, *v*/*v*) with three consecutive developments, affording compounds **10** (3.6 mg) and **14** (6.5 mg).

Fractions FA7 (44.2 mg), FA8 (49.4 mg), and FA9 (62.5 mg), all eluted with CH_2_Cl_2_–EtOAc (80:20, *v*/*v*), FA10 (54.4 mg), eluted with CH_2_Cl_2_–EtOAc (70:30, *v*/*v*), and FA11 (329.0 mg), eluted with CH_2_Cl_2_–EtOAc (70:30, 60:40, 50:50, 40:60, and 30:70, *v*/*v*), exhibited similar chromatographic profiles and were therefore combined to afford 539.5 mg of pooled material. The combined material was rechromatographed over a silica gel column (3.0 × 30.0 cm, i.d. × height) previously treated with a 10% aqueous NaHCO_3_ solution under the same chromatographic conditions used for the initial fractionation of the alkaloid-rich phase (ACP). Approximately 100 mL of mobile phase was used for each solvent system, and eluates were collected in 20-mL vials, yielding 95 fractions in total. Additional volumes of 50 mL or more were used for selected solvent gradients when necessary to ensure complete elution of the constituents. The collected fractions were analyzed by TLC using CH_2_Cl_2_–MeOH (95:5, 90:10, 85:15, and 80:20, *v*/*v*) as the development system. Fractions with similar chromatographic profiles were combined to afford sixteen pooled fractions, designated FA7–11.1 to FA7–11.16.

Fractions FA7–11.8 (42.5 mg), eluted with CH_2_Cl_2_–EtOAc (70:30, *v*/*v*), and FA7–11.9 (55.4 mg), eluted with CH_2_Cl_2_–EtOAc (60:40, *v*/*v*), exhibited similar chromatographic profiles and were therefore combined (97.9 mg). The resulting fraction was subsequently purified by preparative TLC using CH_2_Cl_2_–MeOH (97:3, *v*/*v*) three times, affording compounds **14** (5.1 mg), **15** (4.4 mg), **17** (6.1 mg), and **18** (13.4 mg).

Fraction FA7–11.10 was purified by recrystallization from CH_2_Cl_2_–ethanol (1:1, *v*/*v*), affording additional compound **18** (22.5 mg).

Fraction FA12 (63.2 mg), eluted with CH_2_Cl_2_–EtOAc (30:70 and 20:80, *v*/*v*), was purified by preparative TLC using CH_2_Cl_2_–MeOH (97:3, *v*/*v*) with three consecutive developments, affording compounds **14** (7.6 mg), **17** (10.0 mg), **18** (18.9 mg), and **16** (3.8 mg).

### 3.5. Identification and Characterization of Isolated Compounds

**Acanthoic acid (1):** White crystalline solid (recrystallized from hexane–CH_2_Cl_2_, 4:1, *v*/*v*). [α]^25^D −42 (*c* 1.4, CHCl_3_). The structure was established by analysis of the ^1^H and ^13^C NMR spectroscopic data, which were in agreement with previously reported data [15]. GC–MS: *m*/*z* 302 [M]^•+^.

**Caryophyllene oxide (2):** Colorless oil. The structure was established by analysis of the ^1^H and ^13^C NMR spectroscopic data, which were in agreement with previously reported data [22].

**Mixture of β-sitosterol (3), stigmasterol (4), and campesterol (5):** White needle-shaped crystals (recrystallized from hexane–CH_2_Cl_2_, 3:1, *v*/*v*). The structures were established by analysis of the ^1^H and ^13^C NMR spectroscopic data, which were in agreement with previously reported data [23,24]. GC–MS: *m*/*z* 414, 412, and 400 [M]^•+^, respectively.

**Mixture of β-sitostenone (6), stigmasta-4,22-dien-3-one (7), and campest-4-en-3-one (8):** White, amorphous solid. The structures were established by analysis of the ^1^H and ^13^C NMR spectroscopic data, which were in agreement with previously reported data [25]. GC–MS: *m*/*z* 412, 410, and 398 [M]^•+^, respectively.

**Cadinane-4β,5α,10β-triol (9):** White needle-shaped crystals (recrystallized from CH_2_Cl_2_–MeOH, 2:1, *v*/*v*). The structure was established by analysis of the ^1^H and ^13^C NMR spectroscopic data, which were in agreement with previously reported data [26].

**Liriodenine (10):** Yellow needle-shaped crystals (recrystallized from CH_2_Cl_2_–ethanol, 3:1, *v*/*v*). The structure was established by analysis of the ^1^H and ^13^C NMR spectroscopic data (Table 2), which were in agreement with previously reported data [27]. LR-APCI(+)-MS: *m*/*z* 276 [M + H]^+^.

**Lysicamine (11):** Yellow needle-shaped crystals (recrystallized from CH_2_Cl_2_–ethanol, 3:1, *v*/*v*). The structure was established by analysis of the ^1^H and ^13^C NMR spectroscopic data, which were in agreement with previously reported data [28]. LR-APCI(+)-MS: *m*/*z* 292 [M + H]^+^.

**Anonaine (12):** Brown amorphous solid. The structure was established by analysis of the ^1^H and ^13^C NMR spectroscopic data, which were in agreement with previously reported data [29]. LR-APCI(+)-MS: *m*/*z* 266 [M + H]^+^.

**Nornuciferine (13):** Brown amorphous solid. The structure was established by analysis of the ^1^H and ^13^C NMR spectroscopic data, which were in agreement with previously reported data [30]. LR-APCI(+)-MS: *m*/*z* 282 [M + H]^+^.

**Actinodaphnine (14):** Brown amorphous solid. The structure was established by analysis of the ^1^H and ^13^C NMR spectroscopic data (Table 1), which were in agreement with previously reported data [31]. LR-APCI(+)-MS: *m/z* 312 [M + H]^+^.

***N*-Methylactinodaphnine (cassythicine) (15):** Brown amorphous solid. The structure was established by analysis of the ^1^H and ^13^C NMR spectroscopic data (Table 1), which were in agreement with previously reported data [15]. LR-APCI(+)-MS: *m*/*z* 326 [M + H]^+^.

***N*-Formylactinodaphnine (16*E*/16*Z*):** Brown amorphous solid. IR (KBr), *ν*_max_ cm^−1^: 3271, 2923, 2852, 1651, 1608, 1591, 1461, 1444, 1410, 1393, 1364, 1326, 1292, 1269, 1243, 1194, 1102, 1074, 1048, 1013, 976, 942, 873, 830, 750, 640, 600. The structure was established by analysis of the ^1^H and ^13^C NMR spectroscopic data (Table 1), including COSY, HSQC, and HMBC correlations. HRESIMS: m/z 340.1177 [M + H]^+^ (calcd for C_19_H_18_NO_5_^+^, *m*/*z* 340.1185; Δ = −2.3 ppm). LR-APCI(−)-MS: m/z 338 [M − H]^−^.

**Discretamine (17):** Yellow–orange amorphous solid. The structure was established by analysis of the ^1^H and ^13^C NMR spectroscopic data (Table 2), which were in agreement with previously reported data [29]. LR-APCI(+)-MS: *m*/*z* 328 [M + H]^+^.

**10-Demethyldiscretine (18):** Yellow–orange amorphous solid. The structure was established by analysis of the ^1^H and ^13^C NMR spectroscopic data (Table 2), which were in agreement with previously reported data [32]. LR-APCI(+)-MS: *m*/*z* 328 [M + H]^+^.

The isolated materials used for the cytotoxicity assays were evaluated for spectral homogeneity by inspection of their ^1^H NMR spectra. The spectra predominantly showed the expected resonances for each identified compound, with no major additional signals attributable to substantial impurities.

### 3.6. In Vitro Cytotoxicity Assay

#### 3.6.1. Cell Lines and Culture Conditions

HepG2 (human hepatocellular carcinoma; ATCC^®^ HB-8065™), HCT116 (human colorectal carcinoma; ATCC^®^ CCL-247™), MCF-7 (human breast adenocarcinoma; ATCC^®^ HTB-22™), and MRC-5 (human lung fibroblast; ATCC^®^ CCL-171™) cell lines were obtained from the American Type Culture Collection (ATCC; Manassas, VA, USA). MDA-MB-231 (human breast carcinoma; BCRJ Code 0164) and U-87 MG (human glioblastoma; BCRJ Code 0241) cell lines were obtained from the Rio de Janeiro Cell Bank (BCRJ, Rio de Janeiro, Brazil). Cells were cultured according to the recommendations provided by the ATCC cell bank [44]. Briefly, cells were maintained in DMEM-F12 supplemented with 10% fetal bovine serum (FBS) and 10 µg/mL gentamicin (Sigma–Aldrich) at 37 °C in a humidified atmosphere containing 5% CO_2_. Cells were routinely subcultured when they reached approximately 80–90% confluence. All the cell lines were tested for mycoplasma contamination using a mycoplasma stain kit (Sigma–Aldrich) and were confirmed to be free of detectable contamination.

#### 3.6.2. Cell Viability Assay

Cell viability was evaluated using the resazurin-based Alamar Blue assay according to a previously described method [45], with the modifications and experimental details described below. Cells were seeded in 96-well plates at a density of 7 × 10^3^ cells/well in a final volume of 200 µL and allowed to adhere for 12–24 h before treatment. Test compounds were dissolved in dimethyl sulfoxide (DMSO; Vetec Química Fina Ltd.a., Duque de Caxias, RJ, Brazil) to prepare stock solutions, which were subsequently diluted in culture medium to obtain the desired concentrations. The final concentration of DMSO in the wells was maintained up to 0.5% (*v*/*v*). Cells treated with the corresponding concentration of DMSO in the absence of test compounds were used as vehicle controls. Untreated cells were included as an additional control. Following treatment, the cells were incubated for 72 h at 37 °C in a humidified atmosphere containing 5% CO_2_. At the end of the treatment period, 20 µL of resazurin stock solution (0.312 mg/mL; Sigma–Aldrich Co.) was added to each well, followed by incubation for 4 h at 37 °C in a humidified atmosphere containing 5% CO_2_. The absorbance was then measured at 570 and 600 nm using a SpectraMax 190 microplate reader (Molecular Devices, Sunnyvale, CA, USA).

The Alamar Blue assay was analyzed according to the procedure recommended in the manufacturer’s datasheet. Briefly, the absorbance of Alamar Blue in the culture medium was measured at a higher wavelength and a lower wavelength. The absorbance of the medium alone was also measured at the higher and lower wavelengths. The absorbance of the medium alone was subtracted from the absorbance of the medium plus Alamar Blue at the higher wavelength, and this value was designated AOHW. Similarly, the absorbance of the medium alone was subtracted from the absorbance of the medium plus Alamar Blue at the lower wavelength, and this value was designated AOLW. A correction factor, R_0_, was calculated from AOHW and AOLW, where R_0_ = AOLW/AOHW. The Alamar Blue reduction was then expressed as follows: Reduction = A_LW_ − (A_HW_ × R_0_). The Alamar Blue reduction values obtained for the test wells were normalized to the vehicle control, which was defined as 100% viability, to obtain the relative cell viability.

For IC_50_ determination, the compounds were evaluated at eight serial concentrations ranging from 0.20 to 25 µg/mL, whereas doxorubicin hydrochloride (purity ≥95%; Laboratory IMA S.A.I.C., Buenos Aires, Argentina) was evaluated at eight serial concentrations ranging from 0.08 to 10 µg/mL. Each concentration was tested in technical duplicate, and the experiments were independently repeated three times (three biological replicates). For each compound, the data from the three independent biological experiments were combined into a single concentration–response dataset for 4PL fitting. Half-maximal inhibitory concentration (IC_50_) values were determined by nonlinear regression analysis of the concentration–response curves using a four-parameter logistic (4PL) model in GraphPad Prism version 8 (GraphPad Software, San Diego, CA, USA). The Bottom, Top, Hill slope, and IC_50_ parameters were left unconstrained during curve fitting. IC_50_ values were defined as the concentration corresponding to the midpoint of the fitted 4PL curve, that is, the concentration corresponding to 50% of the response between the fitted lower and upper asymptotes, rather than necessarily representing 50% viability relative to the vehicle control. IC_50_ values are reported together with their corresponding 95% confidence intervals (CIs).

## 4. Conclusions

A phytochemical investigation of the bark of *A. amazonica* led to the isolation and identification of 18 secondary metabolites, comprising a diterpene, sesquiterpenes, steroids, ketosteroids, and alkaloids, thereby expanding the documented phytochemical profile of the species. Initial screening of the extracts and fractions revealed the alkaloid-rich phase as the sample with the highest inhibition values under the experimental conditions employed, and this phase was subsequently prioritized for phytochemical investigation.

Among the isolated metabolites, *N*-formylactinodaphnine (**16**) was isolated and comprehensively characterized by HRESIMS, IR, and 1D and 2D NMR spectroscopy. Its duplicated NMR resonances are consistent with two distinct conformational environments, plausibly associated with restricted rotation about the amide C–*N* bond, with an approximate major-to-minor population ratio of 70:30. The tentative *Z*/*E* assignments were supported by NOESY correlations, although conformational exchange was not directly investigated. The spectroscopic data provide a comprehensive characterization of the isolated material and document the occurrence of *N*-formylactinodaphnine in *A. amazonica*.

Cell-based viability assays revealed that actinodaphnine (**14**) exhibited measurable activity against all five cancer cell lines evaluated, although its activity toward MRC-5 fibroblasts indicated limited selectivity under the conditions tested. *N*-Methylactinodaphnine (cassythicine) (**15**) also showed measurable activity against HCT116 cells, but its evaluation was limited by the amount of isolated material.

Overall, the results of the present study expand the documented chemical profile of *A. amazonica*, provide a comprehensive spectroscopic characterization of *N*-formylactinodaphnine (**16**) isolated from the species, and document measurable effects of alkaloids on cellular viability under the conditions evaluated. These findings should be interpreted in the context of a single collection and the unequal extent of biological evaluation among the isolated compounds. Further studies involving replicated sampling, broader biological evaluation, and mechanism-oriented assays will be necessary to determine the biological significance and pharmacological potential of the metabolites identified in *A. amazonica*.

Nevertheless, the present study has some limitations, including the single plant collection, restricted evaluation of compound **15**, and the absence of direct mechanistic and conformational-exchange measurements. Further studies addressing these aspects are warranted to confirm and extend the present findings.

## Figures and Tables

**Figure 1:**
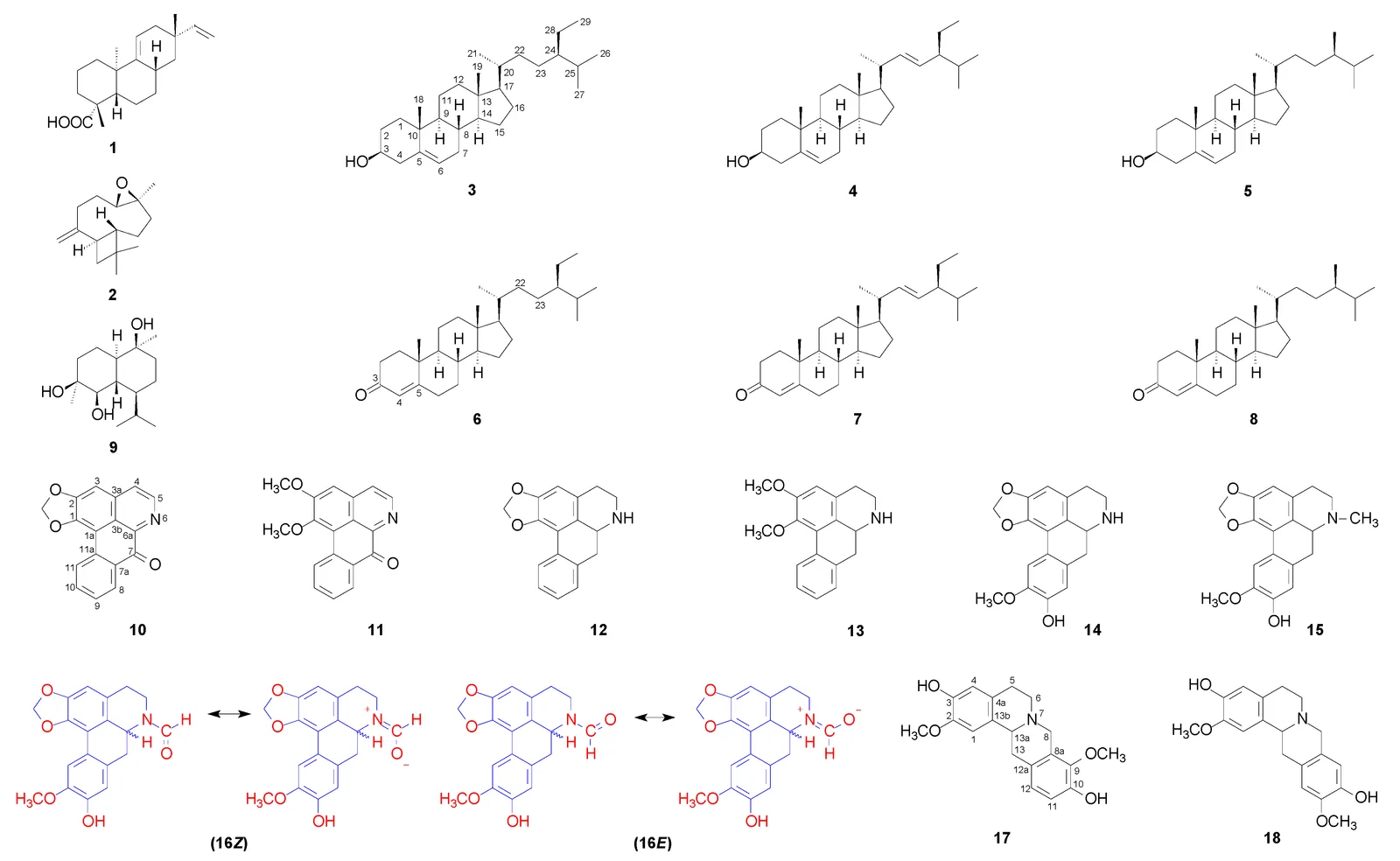
Chemical structures of the compounds isolated from the bark of *A. amazonica*.

**Figure 2:**
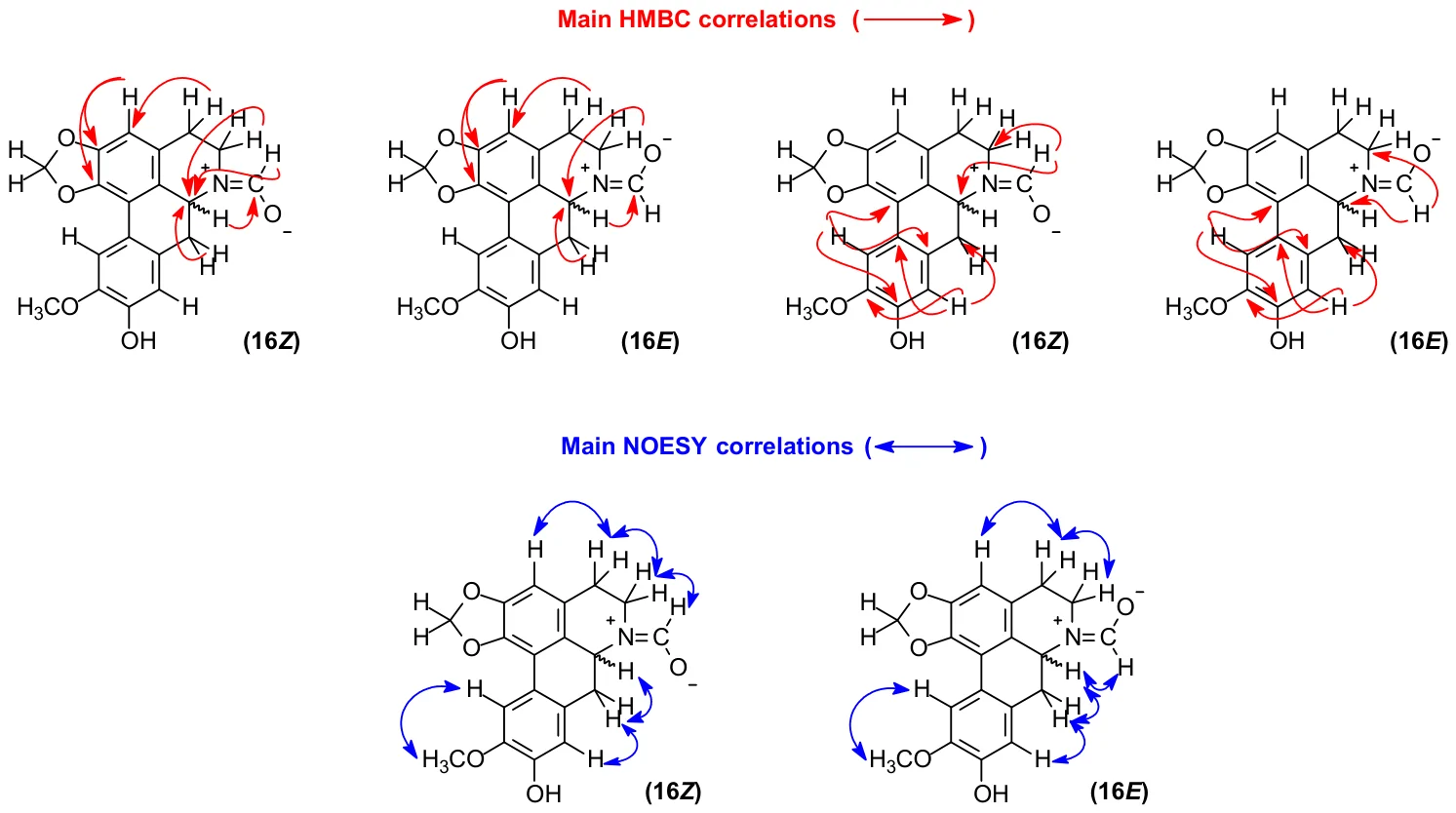
HMBC correlations and selected NOESY correlations supporting the tentative conformational assignments of *N*-formylactinodaphnine (**16**). The diagnostic correlations are δ_H_ 8.25 (CHO)/3.80 (H-5) for the major *Z*-assigned state and δ_H_ 8.38 (CHO)/4.58 (H-6a) and δ_H_ 8.38 (CHO)/2.74 (H-7) for the minor *E*-assigned state. The assignments are based on through-space correlations; direct exchange was not experimentally demonstrated.

**Table 1:** NMR spectroscopic data (500 MHz for ^1^H and 125 MHz for ^13^C) for *N*-formylactinodaphnine (**16**) and *N*-methylactinodaphnine (cassythicine) (**15**) recorded in CDCl_3_ and actinodaphnine (**14**) recorded in CDCl_3_ containing a small amount of CD_3_OD.

Position	16*Z* Rotamer		16*E* Rotamer		14		15	
	*δ*_C_	*δ*_H_ Mult. (*J* in Hz)	*δ*_C_	*δ*_H_ Mult. (*J* in Hz)	*δ*_C_	*δ*_H_ Mult. (*J* in Hz)	*δ*_C_	*δ*_H_ Mult. (*J* in Hz)
1	142.5	-	142.3	-	141.7	-	141.7	-
1a	118.0	-	117.6	-	116.7	-	116.8	-
2	147.1	-	147.4	-	146.9	-	146.7	-
3	106.9	6.53 *s*	107.3	6.56 *s*	107.0	6.49 *s*	106.6	6.51 *s*
3a	126.9	-	127.9	-	126.3	-	125.7	-
3b	124.3	-	123.7	-	126.5	-	128.8	-
4	31.3	2.71 *m* 2.86 *m*	30.0	2.68 *m* 2.77 *m*	28.8	2.63 *m* 2.98 *m*	28.8	2.63 *m* 3.15 *m*
5	42.4	3.39 *td* (12.5; 2.7) 3.80 *ddd* (12.7; 4.5; 1.8)	36.4	3.07 *m* 4.46 *ddd* (12.6; 4.5; 3.2)	42.9	2.98 *m* 3.37 *m*	53.3	2.56 *m* 3.09 *m*
6a	49.9	5.02 *dd* (13.9; 4.5)	53.7	4.58 *dd* (14.6; 4.3)	53.5	3.91 *dd* (14.0; 5.0)	62.2	3.20 *m*
7	33.2	2.73 *t* (13.9) 3.12 *dd* (13.9; 4.5)	37.4	2.74 *m* 3.08 *m*	35.8	2.69 *dd* (14.2; 14.0) 2.81 *dd* (14.2; 5.0)	33.6	2.63 *br d* (14.2) 3.04 *dd* (14.2; 4.3)
7a	129.2	-	128.4	-	128.4	-	128.6	-
8	115.1	6.84 *s*	114.6	6.82 *s*	114.8	6.74 *s*	114.3	6.81 *s*
9	145.7	-	145.9	-	145.6	-	145.1	-
10	145.6	-	145.6	-	146.0	-	145.4	-
11	110.3	7.66 *s*	110.37	7.66 *s*	110.5	7.61 *s*	109.9	7.63 *s*
11a	122.7	-	122.9	-	122.8	-	122.9	-
1-OCH_2_O-2	101.10	5.97 *d* (1.4) 6.10 *d* (1.4)	101.19	5.98 *d* (1.4) 6.11 *d* (1.4)	100.7	5.90 *d* (1.4) 6.05 *d* (1.4)	100.7	5.92 *d* (1.5) 6.07 *d* (1.5)
9-OH	-	- *	-	- *	-	- *	-	- *
10-OCH_3_	56.40	3.93 *s*	56.43	3.95 *s*	56.2	3.88 *s*	56.1	3.92 *s*
*N*-CHO	162.35	8.25 *br s*	162.30	8.38 *br s*	-	-	-	-
*N*-CH_3_	-	-	-	-	-	-	43.5	2.56 *s*

Note: Data for compounds **15** and **16** were obtained in CDCl_3_; data for compound **14** were obtained in CDCl_3_ containing a small amount of CD_3_OD. All the spectra were recorded at 298 K with TMS as an internal reference (0.00 ppm). (*) Phenolic OH was not observed because of exchange.

**Table 2:** NMR spectroscopic data in CDCl_3_ plus CD_3_OD (600 MHz for ^1^H and 150 MHz for ^13^C) for liriodenine (**10**) and CDCl_3_ plus CD_3_OD (500 MHz for ^1^H and 125 MHz for ^13^C) for discretamine (**17**) and 10-demethyldiscretine (**18**).

Position	10		Position	17		18	
	*δ*_C_	*δ*_H_ Mult. (*J* in Hz)		*δ*_C_	*δ*_H_ Mult. (*J* in Hz)	*δ*_C_	*δ*_H_ Mult. (*J* in Hz)
1	148.4		1	108.2	6.71 *s*	107.7	6.71 *s*
1a	107.9		2	145.8	-	145.2	-
2	152.1		3	144.5	-	144.0	-
3	103.4	7.15 *s*	4	114.7	-	114.3	6.67 *s*
3a	136.1		4a	125.8	-	127.4	-
3b	123.3		5	28.2	2.67 *m* 3.10 *m*	28.7	2.63 *m* 3.10 *m*
4	124.7	7.76 *d* (5.1)	6	51.4	2.68 *m* 3.22 *m*	51.3	2.58 *m* 3.11 *m*
5	144.4	8.77 *d* (5.1)	7	-	-	-	-
6a	144.9	-	8	53.7	3.60 *d* (15.6) 4.23 *d* (15.6)	58.0	3.63 *d* (14.7) 3.90 *d* (14.7)
7	182.6	-	8a	127.3	-	126.9	-
7a	131.1	-	9	143.6	-	111.8	6.633 *s*
8	128.6	8.49 *ddd* (7.8; 1.4; 0.5)	10	147.1	-	143.8	-
9	128.7	7.55 *ddd* (7.8; 7.2; 1.0)	11	115.2	6.77 *d* (8.2)	145.3	-
10	134.2	7.72 *ddd* (8.2; 7.2; 1.4)	12	124.6	6.80 *d* (8.2)	110.7	6.638 *s*
11	127.5	8.57 *d* (8.2; 1.0; 0.5)	12a	126.2	-	125.5	-
11a	133.0	-	13	35.6	2.84 *dd* (15.8; 11.6) 3.28 *dd* (15.8; 3.8)	36.4	2.83 *dd* (15.7; 11.3) 3.22 *dd* (15.7; 3.7)
1-OCH_2_O-2	102.8	6.37 *s*	13a	59.5	3.64 *dd* (11.6; 3.8)	59.7	3.57 *dd* (11.3; 3.7)
			13b	128.3	-	129.1	-
			2-OCH_3_	56.1	3.88 *s*	56.0	3.89 *s*
			3-OH	-	- *		- *
			9-OCH_3_	60.3	3.82 *s*	-	-
			10-OH	-	- *	-	- *
			11-OCH_3_	-	-	55.9	3.86 *s*

Note: The data were obtained at 298 K with TMS as an internal reference (0.00 ppm) in CDCl_3_ plus CD_3_OD. (*) Phenolic OH was not observed because of exchange.

**Table 3:** Cytotoxic screening of extracts and phases from *A. amazonica* bark against human tumor cell lines.

Sample	Growth Inhibition %	
	HepG2	HCT116
Hexane extract	4.84 ± 0.79	16.00 ± 1.83
Methanol extract	1.11 ± 0.61	0.78 ± 0.18
Neutral chloroform phase (NCP)	5.43 ± 0.24	0.28 ± 0.03
Alkaloid-rich chloroform phase (ACP)	55.81 ± 1.53	63.32 ± 2.79
Doxorubicin	81.17 ± 0.85	83.74 ± 4.08

Note: Data are expressed as percentage growth inhibition (%GI) ± standard error of the mean (SEM) from two independent experiments (biological replicates) performed in triplicate (technical replicates), as determined by the Alamar Blue assay after 72 h of exposure to HepG2 and HCT116 cells. The extracts and fractions were initially screened at a single concentration of 50 μg/mL, whereas doxorubicin was tested as the positive control at a single concentration of 25 μg/mL. Doxorubicin was used as the positive control.

**Table 4:** Cytotoxic activity of the isolated compounds from the bark of *A. amazonica*.

Compounds	IC_50_, μg/mL (μM), 95% CI ^a^, and Selectivity Index (SI) ^b^					
	HepG2	HCT116	MDA-MB-231	MCF-7	U-87 MG	MRC-5
Acanthoic acid (**1**)	14.63 (48.37) 10.67–20.07 SI > 1.71	21.25 (70.26) 13.25–34.10 SI > 1.18	>25 (82.66)	>25 (82.66)	>25 (82.66)	>25 (82.66)
Actinodaphnine (**14**)	7.21 (23.16) 6.10–8.53 SI = 0.95	5.59 (17.96) 4.34–7.21 SI = 1.22	8.12 (26.08) 5.93–11.10 SI = 0.84	11.73 (37.68) 9.01–15.26 SI = 0.58	10.92 (35.07) 8.99–13.25 SI = 0.63	6.84 (21.97) 6.23–8.39
*N*-Methylactinodaphnine (cassythicine) (**15**)	NT	4.27 (13.12) 3.27–5.59 SI = 2.85	NT	NT	NT	12.16 (37.37) 8.62–17.17
*N*-Formylactinodaphnine (**16**)	>25 (73.67)	21.07 (62.09) 12.75–34.84 SI > 1.19	22.69 (66.86) 17.79–26.04 SI > 1.10	>25 (73.67)	>25 (73.67)	>25 (73.67)
Discretamine (**17**)	>25 (76.36)	>25 (76.36)	>25 (76.36)	>25 (76.36)	>25 (76.36)	>25 (76.36)
10-Demethyldiscretine (**18**)	>25 (76.36)	22.08 (67.45) 11.16–33.67 SI > 1.13	>25 (76.36)	>25 (76.36)	>25 (76.36)	>25 (76.36)
Doxorubicin	0.43 (0.74) 0.37–0.50 SI = 4.56	0.57 (0.98) 0.39–0.85 SI = 3.44	0.62 (1.07) 0.37–1.02 SI = 3.16	0.36 (0.62) 0.20–0.63 SI = 5.44	0.66 (1.14) 0.32–1.34 SI = 2.97	1.96 (3.38) 1.37–2.80

Note: ^a^ Data are presented as IC_50_ values in μg/mL (μM) with 95% confidence intervals (CIs) obtained by nonlinear regression from three independent experiments (biological replicates) performed in duplicate (technical replicates) after 72 h of exposure in the Alamar Blue assay. Cell lines: HepG2, HCT116, MDA-MB-231, MCF-7, U-87 MG, and noncancerous MRC-5 fibroblasts. ^b^ SI: selectivity index, calculated as IC_50_(MRC-5)/IC_50_(cancer cell line). Doxorubicin hydrochloride was used as a positive control. NT, not tested because of an insufficient sample amount.

## Data Availability

The data presented in this study are available in the Supplementary Materials.

## Supplementary Materials

The following supporting information can be downloaded at: https://www.mdpi.com/article/10.3390/molecules31193431/s1, Figures S1–S273: ^1^H and ^13^C 1D and 2D NMR, IR and MS spectra for all the compounds.

## References

[1] Chatrou L.W. (1999). The Annonaceae and the Annonaceae project: A brief overview of the state of affairs. Acta Hortic..

[2] WFO World Flora Online. Version [2026]. http://www.worldfloraonline.org.

[3] Corrêa M.P. (1984). Dicionário das Plantas Úteis do Brasil e das Exóticas Cultivadas.

[4] Gemtchújnicov I.D. (1976). Manual de Taxonomia Vegetal: Plantas de Interesse Econômico.

[5] Leite D.O.D., Nonato C.F.A., Camilo C.J., Carvalho N.K.G., Nobrega M.G.L.A., Pereira R.C.P., Costa J.G.M. (2020). *Annona* Genus: Traditional Uses, Phytochemistry and Biological Activities. Curr. Pharm. Des..

[6] Al-Kazman B.S.M., Hamett J.E., Hanrahan J.R. (2022). Traditional uses, phytochemistry and pharmacological activities of Annonacae. Molecules.

[7] Nugraha A.S., Damayanti Y.D., Wangchuk P., Keller P.A. (2019). Anti-infective and anti-cancer properties of the *Annona* species: Their ethnomedicinal uses, alkaloid diversity, and pharmacological activities. Molecules.

[8] Kusmardiyani S., Suharli Y.A., Insanu M., Fidrianny I. (2020). Phytochemistry and pharmacological activities of *Annona* genus: A review. Curr. Res. Biosci. Biotechnol..

[9] Dey P., Gogoia H., Goyary D., Mandal S. (2024). Unveiling the bioactive phytocompounds of various *Annona* species with their potential role in pharmacological progression. S. Afr. J. Bot..

[10] Corrêa P.L.C., Martínez-Váquez M., Dela-Cruz-Chacón I., Mimi C.O., Ferreira G. (2026). Anticancer activity of *Annona* species: A systematic review. Phytochem. Rev..

[11] Maas P.J.M., Maas H., Miralha J.M.S., Junikka L. (2007). Flora da reserva Ducke, Amazonas, Brasil: Annonaceae. Rodriguésia.

[12] The Herbarium Catalogue, Royal Botanic Gardens, Kew. http://www.kew.org/herbcat.

[13] Mendes-Silva I., Lopes J.C., Silva L.V., Bazante M.L. *Annona* in flora e funga do Brasil. Jardim Botânico do Rio de Janeiro. https://floradobrasil.jbrj.gov.br/FB117059.

[14] Thomsen K., Brimer L. (1997). Cyanogenic constituents in woody plants in natural lowland rain forest in Costa Rica. Bot. J. Linn. Soc..

[15] Pinheiro M.L.B., Xavier C.M., De Souza A.D., Rabelo D.D.M., Batista C.L., Batista R.L., Costa E.V., Campos F.R., Barison A., Valdez R.H. (2009). Acanthoic acid and other constituents from the stem of *Annona amazonica* (Annonaceae). J. Braz. Chem. Soc..

[16] Alcântara J.M., Lucena J.M.V.M., Marques M.O.M., Lima J.A.O., Lima M.P. (2024). Chemical composition and antibacterial activity of the essential oils of the Amazon *Annona* species. Orbital Electron. J. Chem..

[17] Castro M.V.L., de Lima M.C.F., da C. Barbosa G.A., Carvalho S.G., Coelho A.M.R.M., de S. Santos L., Silva V.R., Dias R.B., Soares M.B.P., Costa E.V. (2025). In vitro and in vivo efficacy of the essential oil from the leaves of *Annona amazonica* R.E. Fries (Annonaceae) against liver cancer. Molecules.

[18] Lee S.-S., Lai Y.-C., Chen C.-K., Tseng L.-H., Wang C.-Y. (2007). Characterization of Isoquinoline Alkaloids from *Neolitsea sericea* var. *aurata* by HPLC-SPE-NMR. J. Nat. Prod..

[19] Pachaly P., Adnan A.Z., Will G. (1992). NMR-Assignments of *N-*Acylaporphine Alkaloids from *Tinospora crispa*. Planta Med..

[20] Achenbach H., Renner C., Addae-Mensah I. (1982). Inhaltsstoffe Westafrikanischer Arzneipflanzen, X. Untersuchung der Inhaltsstoffe von *Hexalobus crispiflorus*. Liebigs Ann. Chem..

[21] Barinas J.A., Suárez L.E. (2011). Chemical constituents of *Talauma arcabucoana* (Magnoliaceae): Their brine shrimp lethality and antimicrobial activity. Nat. Prod. Res..

[22] Ramírez-Santos J., Calzada F., García-Hernández N., Barbosa E., Velázquez C., Valdes M. (2024). Caryophyllene oxide, a bicyclic terpenoid isolated from *Annona macroprophyllata* with Antitumor Activity: In vivo, In vitro, and in silico studies. Int. J. Mol. Sci..

[23] Duguma T., Melaku Y., Garg A., Ensermu U. (2024). In vitro antibacterial, DPPH radical scavenging activities, and in silico molecular modeling of isolated compounds from the roots of *Clematis hirsuta*. Adv. Pharmacol. Pharm. Sci..

[24] Uttu A.J., Sallau M.S., Ibrahim H., Iyun O.R.A. (2023). Isolation, characterization, and docking studies of campesterol and β-sitosterol from *Strychnos innocua* (Delile) root bark. J. Taibah Univ. Med. Sci..

[25] do Nascimento I.A. (2013). Estudo Fitoquímico de Mimosa Artemisiana Heringer e Paula. Ph.D. Thesis.

[26] Kuo Y.H., Chyu C.F., Lin H.C. (2003). Cadinane-type sesquiterpenes from the roots of *Taiwania cryptomerioides* Hayata. Chem. Pharm. Bull..

[27] Costa E.V., Pinheiro M.L.B., Souza A.D.L.d., Barison A., Campos F.R., Valdez R.H., Ueda-Nakamura T., Filho B.P.D., Nakamura C.V. (2011). Trypanocidal activity of oxoaporphine and pyrimidine-β-carboline alkaloids from the branches of *Annona foetida* Mart. (Annonaceae). Molecules.

[28] de Oliveira Teles M.N., Dutra L.M., Barison A., Costa E.V. (2015). Alkaloids from leaves of *Annona salzmannii* and *Annona vepretorum* (Annonaceae). Biochem. Syst. Ecol..

[29] Costa E.V., Sampaio M.F.C., Salvador M.S., Nepel A., Barison A. (2015). Chemical constituents from the stem of *Annona pickelii* (Annonaceae). Quim. Nova.

[30] Dutra L.M., Costa E.V., Moraes V.R.S., Nogueira P.C.L., Vendramin M.E., Barison A., Prata A.P.N. (2012). Chemical constituents from the leaves of *Annona pickelii* (Annonaceae). Biochem. Syst. Ecol..

[31] Stévigny C., Block S., De Pauw-Gillet M.C., de Hoffmann E., Llabrès G., Adjakidjé V., Quetin-Leclercq J. (2002). Cytotoxic aporphine alkaloids from *Cassytha filiformis*. Planta Med..

[32] Hocquemiller R., Debitus C., Roblot F., Cavé A., Jacquemin H. (1984). Alcaloïdes des Annonacées, XLVIII. Alcaloïdes des Écorces de *Guatteria discolor*. J. Nat. Prod..

[33] Leboeuf M., Cave A., Bhaumik P.K., Mukherjee B., Mukherjee R. (1982). The phytochemistry of the Annonaceae. Phytochemistry.

[34] Guinaudeau H., Leboeuf M., Cavé A. (1994). Aporphinoid Alkaloids, V. J. Nat. Prod..

[35] Dewick P.M. (2009). Medicinal Natural Products: A Biosynthetic Approach.

[36] Shamma M., Guinaudeau H. (1984). Biogenetic pathways for the aporphinoid alkaloids. Tetrahedron.

[37] Cave A., Leboeuf M., Waterman P.G., Pelletier S.W. (1987). The Aporphinoid alkaloids of the Annonaceae. Alkaloids: Chemical and Biological Perspectives.

[38] Costa E.V., Freitas J.G.C., Manickchand S.P., Araújo M.S., Silva V.R., Santos L.S., Koolen H.H.F., Silva F.M.A., Soares M.B.P., Bezerra D.P. (2024). Gouregine, an α-gem-dimethyltetradehydrocularine alkaloid, and other aporphinoid alkaloids from the bark of *Guatteria olivacea* (Annonaceae) and their in vitro cytotoxic activities. Molecules.

[39] De Souza C.A.S., Nardelli V.B., Paz W.H.P., Pinheiro M.L.B., Rodrigues A.C.B.C., Bomfim L.M., Soares M.B.P., Bezerra D.P., Chaar J.S., Koolen H.H.F. (2020). Asarone-derived phenylpropanoids and isoquinoline-derived alkaloids from the bark of *Duguetia pycnastera* (Annonaceae) and their cytotoxicities. Quim. Nova.

[40] Costa E.V., Pinheiro M.L.B., Maia B.H.L.N.S., Marques F.A., Ruiz A.L.T.G., Marchetti G.M., Carvalho J.E., Soares M.B.P., Costa C.O.S., Galvão A.F.C. (2016). 7,7-Dimethylaporphine and Other Alkaloids from the Bark of *Guatteria friesiana*. J. Nat. Prod..

[41] Menezes L.R.A., Costa C.O.D., Rodrigues A.C.B.d.C., Santo F.R.d.E., Nepel A., Dutra L.M., Silva F.M.A., Soares M.B.P., Barison A., Costa E.V. (2016). Cytotoxic Alkaloids from the Stem of *Xylopia laevigata*. Molecules.

[42] Santos M.F.C., Fontes J.E.N., Dutra L.M., Bomfim L.M., Costa C.O.D., Moraes V.R.S., Barison A., Soares M.B.P., Silva F.M.A., Almeida J.R.G.S. (2018). Alkaloids from Leaves of *Guatteria pogonopus* (Annonaceae) and Their Cytotoxicities. Quim. Nova.

[43] Hsieh T.-J., Liu T.-Z., Lu F.-J., Hsieh P.-Y., Chen C.-H. (2006). Actinodaphnine induces apoptosis through increased nitric oxide, reactive oxygen species and down-regulation of NF-κB signaling in human hepatoma Mahlavu cells. Food Chem. Toxicol..

[44] (2024). ATCC Animal Cell Culture Guide. Get Time-Tested Tips for Culturing ATCC Animal Cells. https://www.atcc.org/resources/culture-guides/animal-cell-culture-guide.

[45] Ahmed S.A., Gogal R.M., Walsh J.E. (1994). A new rapid and simple non-radioactive assay to monitor and determine the proliferation of lymphocytes: An alternative to [^3^H]-thymidine incorporation assay. J. Immunol. Methods.

