# Supplementary material for: Phytochemical Investigation and Cytotoxic Activity of Annona amazonica (Annonaceae): Comprehensive Spectroscopic Characterization of N-Formylactinodaphnine
Source: Molecules. 2026 Sep 27;31(19):3431. doi: 10.3390/molecules31193431 (PMC13666035; doi:10.3390/molecules31193431)
Supplement: Supplementary file 1 [file molecules-31-03431-s001.zip › molecules-4524785-supplementary.pdf]

Supplementary Materials

Phytochemical Investigation and Cytotoxic Activity of *Annona amazonica* (Annonaceae): Comprehensive Spectroscopic Char-acterization of *N*-Formylactinodaphnine

Jéssica Fernandes Auzier [1], Thayline Rayany Batista Menezes [2], Maria Lúcia Belém Pinheiro [2], Maria Victoria Lima de Castro [3], Milena Botelho Pereira Soares [3], Renato Sonchini Gonçalves [4], Josean Fechine Tavares [5], Daniel Pereira Bezerra [3], and Emmanoel Vilaça Costa [1,2,*]

[1] Postgraduate Program in Chemistry, Federal University of Amazonas (UFAM), Manaus 69080-900, AM, Brazil; jessicafauzier27@gmail.com (J.F.A); evc@ufam.edu.br or emmanoelvc@gmail.com (E.V.C.)

[2] Department of Chemistry, Federal University of Amazonas (UFAM), Amazonas, Manaus, 69080-900, Brazil; thaylinerayany4@gmail.com (T.R.B.M.); lbelem1@gmail.com (M.L.B.P.); evc@ufam.edu.br or emmanoelvc@gmail.com (E.V.C.)

[3] Gonçalo Moniz Institute, Oswaldo Cruz Foundation (IGM-FIOCRUZ/BA), Bahia, Salvador, 40296-710, Brazil; mvictorialimac@gmail.com (M.V.L.d.C.); milena.soares@fiocruz.br (M.B.P.S.); daniel.bezerra@fiocruz.br (D.P.B.)

[4] Department of Engineering and Exact Sciences, Setor Palotina, Federal University of Paraná (UFPR), Palotina 85950-000, PR, Brazil; renato.sonchini@ufpr.br (R.SG.)

[5] Postgraduate Program in Natural and Synthetic Bioactive Products, Health Sciences Center, Federal University of Paraíba (UFPB), João Pessoa, Paraíba, 58051-970, Brazil; josean@ltf.ufpb.br (J.F.T.)

***** Correspondence: evc@ufam.edu.br (E.V.C.); daniel.bezerra@fiocruz.br (D.P.B.); Tel./Fax: +55-92-3305-1181 Ramal 2870 (E.V.C.); Tel./Fax: +55-71-3176-2272 (D.P.B.)

Supplementary Figure Captions

Figure S1. Full ^{1}H NMR spectrum of **acanthoic acid** (**1**) recorded in $CDCl_3$ at 500 MHz.

Figure S2. Expanded view of the olefinic proton region in the ^{1}H NMR spectrum of **acanthoic acid** (**1**) recorded in $CDCl_3$ at 500 MHz.

Figure S3. Expanded view of the aliphatic proton region in the ^{1}H NMR spectrum of **acanthoic acid** (**1**) recorded in $CDCl_3$ at 500 MHz.

Figure S4. Full ^{13}C NMR spectrum of **acanthoic acid** (**1**) recorded in $CDCl_3$ at 125 MHz.

Figure S5. Expanded view of the carbon region (δ_C 187.2–106.3 ppm) in the ^{13}C NMR spectrum of **acanthoic acid** (**1**) recorded in $CDCl_3$ at 125 MHz.

Figure S6. Expanded view of the carbon region (δ_C 49.3–17.8 ppm) in the ^{13}C NMR spectrum of **acanthoic acid** (**1**) recorded in $CDCl_3$ at 125 MHz.

Figure S7. Full DEPT-135 ^{13}C NMR spectrum of **acanthoic acid** (**1**) recorded in $CDCl_3$ at 125 MHz.

Figure S8. Expanded view of the carbon region (δ_C 152.7–106.5 ppm) in the DEPT-135 ^{13}C NMR spectrum of **acanthoic acid** (**1**) recorded in $CDCl_3$ at 125 MHz.

Figure S9. Expanded view of the carbon region (δ_C 49.3–17.9 ppm) in the DEPT-135 ^{13}C NMR spectrum of **acanthoic acid** (**1**) recorded in $CDCl_3$ at 125 MHz.

Figure S10. ^{1}H–^{1}H COSY correlation map of **acanthoic acid** (**1**) recorded in $CDCl_3$ at 500 MHz.

Figure S11. Expanded view 1 of the ^{1}H–^{1}H COSY correlation map of **acanthoic acid** (**1**) recorded in $CDCl_3$ at 500 MHz.

Figure S12. Expanded view 2 of the ^{1}H–^{1}H COSY correlation map of **acanthoic acid** (**1**) recorded in $CDCl_3$ at 500 MHz.

Figure S13. One-bond ^{1}H–^{13}C HSQC correlation map of **acanthoic acid** (**1**) recorded in $CDCl_3$ at 500 MHz (^{1}H) and 125 MHz (^{13}C).

Figure S14. Expanded view 1 of the one-bond ^{1}H–^{13}C HSQC correlation map of **acanthoic acid** (**1**) recorded in $CDCl_3$ at 500 MHz (^{1}H) and 125 MHz (^{13}C).

Figure S15. Expanded view 2 of the one-bond ^{1}H–^{13}C HSQC correlation map of **acanthoic acid** (**1**) recorded in $CDCl_3$ at 500 MHz (^{1}H) and 125 MHz (^{13}C).

Figure S16. Long-range ^{1}H–^{13}C HMBC correlation map of **acanthoic acid** (**1**) recorded in $CDCl_3$ at 500 MHz (^{1}H) and 125 MHz (^{13}C).

Figure S17. Expanded view 1 of the long-range ^{1}H–^{13}C HMBC correlation map of **acanthoic acid** (**1**) recorded in $CDCl_3$ at 500 MHz (^{1}H) and 125 MHz (^{13}C).

Figure S18. Expanded view 2 of the long-range ^{1}H–^{13}C HMBC correlation map of **acanthoic acid** (**1**) recorded in $CDCl_3$ at 500 MHz (^{1}H) and 125 MHz (^{13}C).

Figure S19. Total ion chromatogram (GC–MS) of acanthoic acid (**1**) with a retention time of 7.91 min.

Figure S20. Mass spectrum of acanthoic acid (**1**), corresponding to the chromatographic peak at a retention time of 7.91 min, showing the molecular ion at *m/z* 302 ($M^{+•}$).

Figure S21. Full ^{1}H NMR spectrum of **caryophyllene oxide** (**2**) recorded in $CDCl_3$ at 500 MHz.

Figure S22. Expanded view of the olefinic proton region in the ^{1}H NMR spectrum of **caryophyllene oxide** (**2**) recorded in $CDCl_3$ at 500 MHz.

Figure S23. Expanded view of the aliphatic proton region in the ^{1}H NMR spectrum of **caryophyllene oxide** (**2**) recorded in $CDCl_3$ at 500 MHz.

Figure S24. Full ^{13}C NMR spectrum of **caryophyllene oxide** (**2**) recorded in $CDCl_3$ at 125 MHz.

Figure S25. Expanded view of the carbon region (δ_C 155.0–110.5 ppm) in the ^{13}C NMR spectrum of **caryophyllene oxide** (**2**) recorded in $CDCl_3$ at 125 MHz.

Figure S26. Expanded view of the carbon region (δ_C 65.6–15.8 ppm) in the ^{13}C NMR spectrum of **caryophyllene oxide** (**2**) recorded in $CDCl_3$ at 125 MHz.

Figure S27. Full DEPT-135 ^{13}C NMR spectrum of **caryophyllene oxide** (**2**) recorded in $CDCl_3$ at 125 MHz.

Figure S28. Expanded view of the carbon region (δ_C 118.6–13.4 ppm) in the DEPT-135 ^{13}C NMR spectrum of **caryophyllene oxide** (**2**) recorded in $CDCl_3$ at 125 MHz.

Figure S29. Expanded view of the carbon region (δ_C 65.5–15.3 ppm) in the DEPT-135 ^{13}C NMR spectrum of **caryophyllene oxide** (**2**) recorded in $CDCl_3$ at 125 MHz.

Figure S30. Full ^{1}H NMR spectrum of the mixture of **β-sitosterol** (**3**), **stigmasterol** (**4**), and **campesterol** (**5**), recorded in $CDCl_3$ at 500 MHz.

Figure S31. Expanded view of the olefinic and carbinolic proton region in the ^{1}H NMR spectrum of **β-sitosterol** (**3**), **stigmasterol** (**4**), and **campesterol** (**5**), recorded in $CDCl_3$ at 500 MHz.

Figure S32. Expanded view of the aliphatic proton region in the ^{1}H NMR spectrum of **β-sitosterol** (**3**), **stigmasterol** (**4**), and **campesterol** (**5**), recorded in $CDCl_3$ at 500 MHz.

Figure S33. Full ^{13}C NMR spectrum of the mixture of **β-sitosterol** (**3**), **stigmasterol** (**4**), and **campesterol** (**5**), recorded in $CDCl_3$ at 125 MHz.

Figure S34. Expanded view of the carbon region (δ_C 143.0–70.0 ppm) in the ^{13}C NMR spectrum of the mixture of **β-sitosterol** (**3**), **stigmasterol** (**4**), and **campesterol** (**5**), recorded in $CDCl_3$ at 125 MHz.

Figure S35. Expanded view of the carbon region (δ_C 58.6–10.6 ppm) in the ^{13}C NMR spectrum of the mixture of **β-sitosterol** (**3**), **stigmasterol** (**4**), and **campesterol** (**5**), recorded in $CDCl_3$ at 125 MHz.

Figure S36. Full DEPT-135 ^{13}C NMR spectrum of the mixture of **β-sitosterol** (**3**), **stigmasterol** (**4**), and **campesterol** (**5**), recorded in $CDCl_3$ at 125 MHz.

Figure S37. Expanded view of the carbon region (δ_C 141.5–70.0 ppm) in the DEPT-135 ^{13}C NMR spectrum of the mixture of **β-sitosterol** (**3**), **stigmasterol** (**4**), and **campesterol** (**5**), recorded in $CDCl_3$ at 125 MHz.

Figure S38. Expanded view of the carbon region (δ_C 59.0–10.4 ppm) in the DEPT-135 ^{13}C NMR spectrum of the mixture of **β-sitosterol** (**3**), **stigmasterol** (**4**), and **campesterol** (**5**), recorded in $CDCl_3$ at 125 MHz.

Figure S39. Total ion chromatogram (GC–MS) of the mixture of **β-sitosterol** (**3**), **stigmasterol** (**4**), and **campesterol** (**5**), with retention times of 16.32, 15.55, and 15.26 min, respectively.

Figure S40. Mass spectrum of **β-sitosterol** (**3**), corresponding to the chromatographic peak at a retention time of 16.32 min, showing the molecular ion at *m/z* 414 ($M^{+\bullet}$).

Figure S41. Mass spectrum of **stigmasterol** (**4**), corresponding to the chromatographic peak at a retention time of 15.55 min, showing the molecular ion at *m/z* 412 ($M^{+\bullet}$).

Figure S42. Mass spectrum of **campesterol** (**5**), corresponding to the chromatographic peak at a retention time of 15.26 min, showing the molecular ion at *m/z* 400 ($M^{+\bullet}$).

Figure S43. Full ^{1}H NMR spectrum of the mixture of **β-sitostenone** (**6**), **stigmasta-4,22-dien-3-one** (**7**), and **campest-4-en-3-one** (**8**), recorded in $CDCl_3$ at 500 MHz.

Figure S44. Expanded view of the olefinic and carbinolic proton region in the ^{1}H NMR spectrum of mixture of **β-sitostenone** (**6**), **stigmasta-4,22-dien-3-one** (**7**), and **campest-4-en-3-one** (**8**), recorded in $CDCl_3$ at 500 MHz.

Figure S45. Expanded view of the aliphatic proton region in the ^{1}H NMR spectrum of mixture of **β-sitostenone** (**6**), **stigmasta-4,22-dien-3-one** (**7**), and **campest-4-en-3-one** (**8**), recorded in $CDCl_3$ at 500 MHz.

Figure S46. Full ^{13}C NMR spectrum of the mixture of **β-sitostenone** (**6**), **stigmasta-4,22-dien-3-one** (**7**), and **campest-4-en-3-one** (**8**), recorded in $CDCl_3$ at 125 MHz.

Figure S47. Expanded view of the carbon region (δ_C 203.2–120.7 ppm) in the ^{13}C NMR spectrum of the mixture of **β-sitostenone** (**6**), **stigmasta-4,22-dien-3-one** (**7**), and **campest-4-en-3-one** (**8**), recorded in $CDCl_3$ at 125 MHz.

Figure S48. Expanded view of the carbon region (δ_C 58.7–10.2 ppm) in the ^{13}C NMR spectrum of the mixture of **β-sitostenone** (**6**), **stigmasta-4,22-dien-3-one** (**7**), and **campest-4-en-3-one** (**8**), recorded in $CDCl_3$ at 125 MHz.

Figure S49. Full DEPT-135 ^{13}C NMR spectrum of the mixture of **β-sitostenone** (**6**), **stigmasta-4,22-dien-3-one** (**7**), and **campest-4-en-3-one** (**8**), recorded in $CDCl_3$ at 125 MHz.

Figure S50. Expanded view of the carbon region (δ_C 142.7–48.0 ppm) in the DEPT-135 ^{13}C NMR spectrum of the mixture of **β-sitostenone** (**6**), **stigmasta-4,22-dien-3-one** (**7**), and **campest-4-en-3-one** (**8**), recorded in $CDCl_3$ at 125 MHz.

Figure S51. Expanded view of the carbon region (δ_C 57.8–10.3 ppm) in the DEPT-135 ^{13}C NMR spectrum of the mixture of **β-sitostenone** (**6**), **stigmasta-4,22-dien-3-one** (**7**), and **campest-4-en-3-one** (**8**), recorded in $CDCl_3$ at 125 MHz.

Figure S52. Total ion chromatogram (GC–MS) of the mixture of **β-sitostenone** (**6**), **stigmasta-4,22-dien-3-one** (**7**), and **campest-4-en-3-one** (**8**), with retention times of 16.32, 15.55, and 15.26 min, respectively.

Figure S53. Mass spectrum of **β-sitostenone** (**6**), corresponding to the chromatographic peak at a retention time of 18.25 min showing the molecular ion at *m/z* 412 ($M^{+\bullet}$).

Figure S54. Mass spectrum of **stigmasta-4,22-dien-3-one** (**7**), corresponding to the chromatographic peak at a retention time of 17.26 min showing the molecular ion at *m/z* 410 ($M^{+\bullet}$).

Figure S55. Mass spectrum of **campest-4-en-3-one** (**8**), corresponding to the chromatographic peak at a retention time of 16.91 min showing the molecular ion at *m/z* 398 ($M^{+\bullet}$).

Figure S56. Full ^{1}H NMR spectrum of **cadinane-4β,5α,10β-triol** (**9**) recorded in $CDCl_3$ at 500 MHz.

Figure S57. Expanded view 1 of the aliphatic proton region in the ^{1}H NMR spectrum of **cadinane-4β,5α,10β-triol** (**9**) recorded in $CDCl_3$ at 500 MHz.

Figure S58. Expanded view 2 of the aliphatic proton region in the ^{1}H NMR spectrum of **cadinane-4β,5α,10β-triol** (**9**) recorded in $CDCl_3$ at 500 MHz.

Figure S59. Full ^{13}C NMR spectrum of **cadinane-4β,5α,10β-triol** (**9**) recorded in $CDCl_3$ at 125 MHz.

Figure S60. Expanded view of the carbon region (δ_C 74.4–14.3 ppm) in the ^{13}C NMR spectrum of **cadinane-4β,5α,10β-triol** (**9**) recorded in $CDCl_3$ at 125 MHz.

Figure S61. Expanded view of the carbon region (δ_C 43.5–14.3 ppm) in the ^{13}C NMR spectrum of **cadinane-4β,5α,10β-triol** (**9**) recorded in $CDCl_3$ at 125 MHz.

Figure S62. Full DEPT-135 ^{13}C NMR spectrum of **cadinane-4β,5α,10β-triol** (**9**) recorded in $CDCl_3$ at 125 MHz.

Figure S63. Expanded view of the carbon region (δ_C 75.8–11.9 ppm) in the DEPT-135 ^{13}C NMR spectrum of **cadinane-4β,5α,10β-triol** (**9**) recorded in $CDCl_3$ at 125 MHz.

Figure S64. Expanded view of the carbon region (δ_C 43.3–14.6 ppm) in the DEPT-135 ^{13}C NMR spectrum of **cadinane-4β,5α,10β-triol** (**9**) recorded in $CDCl_3$ at 125 MHz.

Figure S65. Full ^1H NMR spectrum of **liriodenine** (**10**) recorded in $CDCl_3$ with a drop of CD_3OD at 600 MHz.

Figure S66. Expanded view 1 of the aromatic proton region in the ^1H NMR spectrum of **liriodenine** (**10**) recorded in $CDCl_3$ with a drop of CD_3OD at 600 MHz.

Figure S67. Expanded view 2 of the aromatic proton region in the ^1H NMR spectrum of **liriodenine** (**10**) recorded in $CDCl_3$ with a drop of CD_3OD at 600 MHz.

Figure S68. Full ^{13}C NMR spectrum of **liriodenine** (**10**) recorded in $CDCl_3$ with a drop of CD_3OD at 150 MHz.

Figure S69. Expanded view of the carbon region (δ_C 185.3–99.9 ppm) in the ^{13}C NMR spectrum of **liriodenine** (**10**) recorded in $CDCl_3$ with a drop of CD_3OD at 150 MHz.

Figure S70. Expanded view of the carbon region (δ_C 154.4–122.0 ppm) in the ^{13}C NMR spectrum of **liriodenine** (**10**) recorded in $CDCl_3$ with a drop of CD_3OD at 150 MHz.

Figure S71. Full DEPT-135 ^{13}C NMR spectrum of **liriodenine** (**10**) recorded in $CDCl_3$ with a drop of CD_3OD at 150 MHz.

Figure S72. Expanded view of the carbon region (δ_C 146.1–100.9 ppm) in the DEPT-135 ^{13}C NMR spectrum of **liriodenine** (**10**) recorded in $CDCl_3$ with a drop of CD_3OD at 150 MHz.

Figure S73. Expanded view of the carbon region (δ_C 145.4–123.5 ppm) in the DEPT-135 ^{13}C NMR spectrum of **liriodenine** (**10**) recorded in $CDCl_3$ with a drop of CD_3OD at 150 MHz.

Figure S74. ^1H–^1H COSY correlation map of **liriodenine** (**10**) recorded in $CDCl_3$ with a drop of CD_3OD at 600 MHz.

Figure S75. Expanded view 1 of the ^1H–^1H COSY correlation map of **liriodenine** (**10**) recorded in $CDCl_3$ with a drop of CD_3OD at 600 MHz.

Figure S76. Expanded view 2 of the ^1H–^1H COSY correlation map of **liriodenine** (**10**) recorded in $CDCl_3$ with a drop of CD_3OD at 600 MHz.

Figure S77. One-bond ^1H–^{13}C HSQC correlation map of **liriodenine** (**10**) recorded in $CDCl_3$ with a drop of CD_3OD at 600 MHz (^1H) and 150 MHz (^{13}C).

Figure S78. Expanded view 1 of the one-bond ^1H–^{13}C HSQC correlation map of **liriodenine** (**10**) recorded in $CDCl_3$ with a drop of CD_3OD at 600 MHz (^1H) and 150 MHz (^{13}C).

Figure S79. Expanded view 2 of the one-bond ^1H–^{13}C HSQC correlation of **liriodenine** (**10**) recorded in $CDCl_3$ with a drop of CD_3OD at 600 MHz (^1H) and 150 MHz (^{13}C).

Figure S80. Long-range ^1H–^{13}C HMBC correlation map of **liriodenine** (**10**) recorded in $CDCl_3$ with a drop of CD_3OD at 600 MHz (^1H) and 150 MHz (^{13}C).

Figure S81. Expanded view 1 of the long-range ^1H–^{13}C HMBC correlation map of **liriodenine** (**10**) recorded in $CDCl_3$ with a drop of CD_3OD at 600 MHz (^1H) and 150 MHz (^{13}C).

Figure S82. Expanded view 2 of the long-range ^1H–^{13}C HMBC correlation map of **liriodenine** (**10**) recorded in $CDCl_3$ with a drop of CD_3OD at 600 MHz (^1H) and 150 MHz (^{13}C).

Figure S83. LR-APCI(+)-MS spectrum of **liriodenine** (**10**), showing the protonated molecular ion at *m/z* 276 $[M + H]^+$.

Figure S84. LR-APCI(+)-MS/MS spectrum of the protonated molecule of **liriodenine** (**10**) at *m/z* 276 $[M + H]^+$.

Figure S85. Full ^1H NMR spectrum of **lysicamine** (**11**) recorded in $CDCl_3$ at 500 MHz.

Figure S86. Expanded view 1 of the aromatic proton region in the ^1H NMR spectrum of **lysicamine** (**11**) recorded in $CDCl_3$ at 500 MHz.

Figure S87. Expanded view 2 of the aromatic proton region in the ^1H NMR spectrum of **lysicamine** (**11**) recorded in $CDCl_3$ at 500 MHz.

Figure S88. Full ^{13}C NMR spectrum of **lysicamine** (**11**) recorded in $CDCl_3$ at 125 MHz.

Figure S89. Expanded view of the carbon region (δ_C 186.0–102.6 ppm) in the ^{13}C NMR spectrum of **lysicamine** (**11**) recorded in $CDCl_3$ at 125 MHz.

Figure S90. Expanded view of the carbon region (δ_C 158.8–118.2 ppm) in the ^{13}C NMR spectrum of **lysicamine** (**11**) recorded in $CDCl_3$ at 125 MHz.

Figure S91. Full DEPT-135 ^{13}C NMR spectrum of **lysicamine** (**11**) recorded in $CDCl_3$ at 125 MHz.

Figure S92. Expanded view of the carbon region (δ_C 146.9–105.9 ppm) in the DEPT-135 ^{13}C NMR spectrum of **lysicamine** (**11**) recorded in $CDCl_3$ at 125 MHz.

Figure S93. Expanded view of the carbon region (δ_C 129.0–128.3 ppm) in the DEPT-135 ^{13}C NMR spectrum of **lysicamine** (**11**) recorded in $CDCl_3$ at 125 MHz.

Figure S94. ^1H–^1H COSY correlation map of **lysicamine** (**11**) recorded in $CDCl_3$ at 500 MHz.

Figure S95. Expanded view 1 of the ^1H–^1H COSY correlation map of **lysicamine** (**11**) recorded in $CDCl_3$ at 500 MHz.

Figure S96. Expanded view 2 of the ^1H–^1H COSY correlation map of **lysicamine** (**11**) recorded in $CDCl_3$ at 500 MHz.

Figure S97. One-bond ^1H–^{13}C HSQC correlation map of **lysicamine** (**11**) recorded in $CDCl_3$ at 500 MHz (^1H) and 125 MHz (^{13}C).

Figure S98. Expanded view 1 of the one-bond ^1H–^{13}C HSQC correlation map of **lysicamine** (**11**) recorded in $CDCl_3$ at 500 MHz (^1H) and 125 MHz (^{13}C).

Figure S99. Expanded view 2 of the one-bond ^1H–^{13}C HSQC correlation of **lysicamine** (**11**) recorded in $CDCl_3$ at 500 MHz (^1H) and 125 MHz (^{13}C).

Figure S100. Long-range ^1H–^{13}C HMBC correlation map of **lysicamine** (**11**) recorded in $CDCl_3$ at 500 MHz (^1H) and 125 MHz (^{13}C).

Figure S101. Expanded view 1 of the long-range ^1H–^{13}C HMBC correlation map of **lysicamine** (**11**) recorded in $CDCl_3$ at 500 MHz (^1H) and 125 MHz (^{13}C).

Figure S102. Expanded view 2 of the long-range ^1H–^{13}C HMBC correlation map of **lysicamine** (**11**) recorded in $CDCl_3$ at 500 MHz (^1H) and 125 MHz (^{13}C).

Figure S103. LR-APCI(+)-MS spectrum of **lysicamine** (**11**), showing the protonated molecular ion at *m/z* 292 $[M + H]^+$.

Figure S104. LR-APCI(+)-MS/MS spectrum of the protonated molecule of **lysicamine** (**11**) at *m/z* 292 $[M + H]^+$.

Figure S105. Full ^1H NMR spectrum of **anonaine** (**12**) recorded in $CDCl_3$ at 600 MHz.

Figure S106. Expanded view 1 of the aromatic proton region in the ^1H NMR spectrum of **anonaine** (**12**) recorded in $CDCl_3$ at 600 MHz.

Figure S107. Expanded view 2 of the aliphatic proton region in the ^{1}H NMR spectrum **anonaine** (**12**) recorded in $CDCl_3$ at 600 MHz.

Figure S108. ^{1}H–^{1}H COSY correlation map of **anonaine** (**12**) recorded in $CDCl_3$ at 600 MHz.

Figure S109. Expanded view 1 of the ^{1}H–^{1}H COSY correlation map of **anonaine** (**12**) recorded in $CDCl_3$ at 600 MHz.

Figure S110. Expanded view 2 of the ^{1}H–^{1}H COSY correlation map of **anonaine** (**12**) recorded in $CDCl_3$ at 600 MHz.

Figure S111. One-bond ^{1}H–^{13}C HSQC correlation map of **anonaine** (**12**) recorded in $CDCl_3$ with a drop of CD_3OD at 600 MHz (^{1}H) and 150 MHz (^{13}C).

Figure S112. Expanded view 1 of the one-bond ^{1}H–^{13}C HSQC correlation map of **anonaine** (**12**) recorded in $CDCl_3$ at 600 MHz (^{1}H) and 150 MHz (^{13}C).

Figure S113. Expanded view 2 of the one-bond ^{1}H–^{13}C HSQC correlation of **anonaine** (**12**) recorded in $CDCl_3$ at 600 MHz (^{1}H) and 150 MHz (^{13}C).

Figure S114. Long-range ^{1}H–^{13}C HMBC correlation map of **anonaine** (**12**) recorded in $CDCl_3$ at 600 MHz (^{1}H) and 150 MHz (^{13}C).

Figure S115. Expanded view 1 of the long-range ^{1}H–^{13}C HMBC correlation map of **anonaine** (**12**) recorded in $CDCl_3$ at 600 MHz (^{1}H) and 150 MHz (^{13}C).

Figure S116. Expanded view 2 of the long-range ^{1}H–^{13}C HMBC correlation map of **anonaine** (**12**) recorded in $CDCl_3$ at 600 MHz (^{1}H) and 150 MHz (^{13}C).

Figure S117. LR-APCI(+)-MS spectrum of **anonaine** (**12**), showing the protonated molecular ion at *m/z* 266 $[M + H]^+$.

Figure S118. LR-APCI(+)-MS/MS spectrum of the protonated molecule of **anonaine** (**12**) at *m/z* 266 $[M + H]^+$.

Figure S119. Full ^{1}H NMR spectrum of **nornuciferine** (**13**) recorded in $CDCl_3$ at 600 MHz.

Figure S120. Expanded view 1 of the aromatic proton region in the ^{1}H NMR spectrum of **nornuciferine** (**13**) recorded in $CDCl_3$ at 600 MHz.

Figure S121. Expanded view 2 of the aliphatic proton region in the ^{1}H NMR spectrum nornuciferine (**13**) recorded in $CDCl_3$ at 600 MHz.

Figure S122. Full ^{13}C NMR spectrum of **nornuciferine** (**13**) recorded in $CDCl_3$ at 150 MHz.

Figure S123. Expanded view of the carbon region (δ_C 153.7–110.4 ppm) in the ^{13}C NMR spectrum of **nornuciferine** (**13**) recorded in $CDCl_3$ at 150 MHz.

Figure S124. Expanded view of the carbon region (δ_C 61.1–27.5 ppm) in the ^{13}C NMR spectrum of **nornuciferine** (**13**) recorded in $CDCl_3$ at 150 MHz.

Figure S125. One-bond ^{1}H–^{13}C HSQC correlation map of **nornuciferine** (**13**) recorded in $CDCl_3$ with a drop of CD_3OD at 600 MHz (^{1}H) and 150 MHz (^{13}C).

Figure S126. Expanded view 1 of the one-bond ^{1}H–^{13}C HSQC correlation map of **nornuciferine** (**13**) recorded in $CDCl_3$ at 600 MHz (^{1}H) and 150 MHz (^{13}C).

Figure S127. Expanded view 2 of the one-bond ^{1}H–^{13}C HSQC correlation of **nornuciferine** (**13**) recorded in $CDCl_3$ at 600 MHz (^{1}H) and 150 MHz (^{13}C).

Figure S128. Long-range ^{1}H–^{13}C HMBC correlation map of **nornuciferine** (**13**) recorded in $CDCl_3$ at 600 MHz (^{1}H) and 150 MHz (^{13}C).

Figure S129. Expanded view 1 of the long-range ^{1}H–^{13}C HMBC correlation map of **nornuciferine** (**13**) recorded in $CDCl_3$ at 600 MHz (^{1}H) and 150 MHz (^{13}C).

Figure S130. Expanded view 2 of the long-range ^{1}H–^{13}C HMBC correlation map of **nornuciferine** (**13**) recorded in $CDCl_3$ at 600 MHz (^{1}H) and 150 MHz (^{13}C).

Figure S131. LR-APCI(+)-MS spectrum of **nornuciferine** (**13**), showing the protonated molecular ion at *m/z* 282 $[M + H]^+$.

Figure S132. LR-APCI(+)-MS/MS spectrum of the protonated molecule of **nornuciferine** (**13**) at *m/z* 282 $[M + H]^+$.

Figure S133. Full ^{1}H NMR spectrum of **actinodaphnine** (**14**) recorded in $CDCl_3$ with a drop of CD_3OD at 500 MHz.

Figure S134. Expanded view 1 of the aromatic proton region in the ^{1}H NMR spectrum of **actinodaphnine** (**14**) recorded in $CDCl_3$ with a drop of CD_3OD at 500 MHz.

Figure S135. Expanded view 2 of the aliphatic proton region in the ^{1}H NMR spectrum **actinodaphnine** (**14**) recorded in $CDCl_3$ with a drop of CD_3OD at 500 MHz.

Figure S136. Full ^{13}C NMR spectrum of **actinodaphnine** (**14**) recorded in $CDCl_3$ with a drop of CD_3OD at 125 MHz.

Figure S137. Expanded view of the carbon region (δ_C 148.2–99.5 ppm) in the ^{13}C NMR spectrum of **actinodaphnine** (**14**) recorded in $CDCl_3$ with a drop of CD_3OD at 125 MHz.

Figure S138. Expanded view of the carbon region (δ_C 57.1–27.5 ppm) in the ^{13}C NMR spectrum of **actinodaphnine** (**14**) recorded in $CDCl_3$ with a drop of CD_3OD at 125 MHz.

Figure S139. Full DEPT-135 ^{13}C NMR spectrum of **actinodaphnine** (**14**) recorded in $CDCl_3$ with a drop of CD_3OD at 125 MHz.

Figure S140. Expanded view of the carbon region (δ_C 115.6–100.0 ppm) in the DEPT-135 ^{13}C NMR spectrum of **actinodaphnine** (**14**) recorded in $CDCl_3$ with a drop of CD_3OD at 125 MHz.

Figure S141. Expanded view of the carbon region (δ_C 57.2–28.4 ppm) in the DEPT-135 ^{13}C NMR spectrum of **actinodaphnine** (**14**) recorded in $CDCl_3$ with a drop of CD_3OD at 125 MHz.

Figure S142. ^{1}H–^{1}H COSY correlation map of **actinodaphnine** (**14**) recorded in $CDCl_3$ with a drop of CD_3OD at 500 MHz.

Figure S143. Expanded view 1 of the ^{1}H–^{1}H COSY correlation map of **actinodaphnine** (**14**) recorded in $CDCl_3$ with a drop of CD_3OD at 500 MHz.

Figure S144. Expanded view 2 of the ^{1}H–^{1}H COSY correlation map of **actinodaphnine** (**14**) recorded in $CDCl_3$ with a drop of CD_3OD at 500 MHz.

Figure S145. One-bond ^{1}H–^{13}C HSQC correlation map of **actinodaphnine** (**14**) recorded in $CDCl_3$ with a drop of CD_3OD at 500 MHz (^{1}H) and 125 MHz (^{13}C).

Figure S146. Expanded view 1 of the one-bond ^{1}H–^{13}C HSQC correlation map of **actinodaphnine** (**14**) recorded in $CDCl_3$ with a drop of CD_3OD at 500 MHz (^{1}H) and 125 MHz (^{13}C).

Figure S147. Expanded view 2 of the one-bond ^{1}H–^{13}C HSQC correlation of **actinodaphnine** (**14**) recorded in $CDCl_3$ with a drop of CD_3OD at 500 MHz (^{1}H) and 125 MHz (^{13}C).

Figure S148. Long-range ^{1}H–^{13}C HMBC correlation map of **actinodaphnine** (**14**) recorded in $CDCl_3$ with a drop of CD_3OD at 500 MHz (^{1}H) and 125 MHz (^{13}C).

Figure S149. Expanded view 1 of the long-range ^{1}H–^{13}C HMBC correlation map of **actinodaphnine** (**14**) recorded in $CDCl_3$ with a drop of CD_3OD at 500 MHz (^{1}H) and 125 MHz (^{13}C).

Figure S150. Expanded view 2 of the long-range ^{1}H–^{13}C HMBC correlation map of **actinodaphnine** (**14**) recorded in $CDCl_3$ with a drop of CD_3OD at 600 MHz (^{1}H) and 150 MHz (^{13}C).

Figure S151. LR-APCI(+)-MS spectrum of **actinodaphnine** (**14**), showing the protonated molecular ion at *m/z* 312 $[M + H]^+$.

Figure S152. LR-APCI(+)-MS/MS spectrum of the protonated molecule of **actinodaphnine** (**14**) at *m/z* 312 $[M + H]^+$.

Figure S153. Full ^{1}H NMR spectrum of ***N*-methylactinodaphnine** (**15**) recorded in $CDCl_3$ at 500 MHz.

Figure S154. Expanded view 1 of the aromatic proton region in the ^{1}H NMR spectrum of ***N*-methylactinodaphnine** (**15**) recorded in $CDCl_3$ at 500 MHz.

Figure S155. Expanded view 2 of the aliphatic proton region in the ^{1}H NMR spectrum ***N*-methylactinodaphnine** (**15**) recorded in $CDCl_3$ at 500 MHz.

Figure S156. Full ^{13}C NMR spectrum of ***N*-methylactinodaphnine** (**15**) recorded in $CDCl_3$ at 125 MHz.

Figure S157. Expanded view of the carbon region (δ_C 148.5–99.0 ppm) in the ^{13}C NMR spectrum of ***N*-methylactinodaphnine** (**15**) recorded in $CDCl_3$ at 125 MHz.

Figure S158. Expanded view of the carbon region (δ_C 63.6–27.2 ppm) in the ^{13}C NMR spectrum of ***N*-methylactinodaphnine** (**15**) recorded in $CDCl_3$ at 125 MHz.

Figure S159. Full DEPT-135 ^{13}C NMR spectrum of ***N*-methylactinodaphnine** (**15**) recorded in $CDCl_3$ at 125 MHz.

Figure S160. Expanded view of the carbon region (δ_C 115.6–99.3 ppm) in the DEPT-135 ^{13}C NMR spectrum of ***N*-methylactinodaphnine** (**15**) recorded in $CDCl_3$ at 125 MHz.

Figure S161. Expanded view of the carbon region (δ_C 57.2–28.4 ppm) in the DEPT-135 ^{13}C NMR spectrum of ***N*-methylactinodaphnine** (**15**) recorded in $CDCl_3$ at 500 MHz.

Figure S162. ^{1}H–^{1}H COSY correlation map ***N*-methylactinodaphnine** (**15**) recorded in $CDCl_3$ at 500 MHz.

Figure S163. Expanded view 1 of the ^{1}H–^{1}H COSY correlation map of ***N*-methylactinodaphnine** (**15**) recorded in $CDCl_3$ at 500 MHz.

Figure S164. Expanded view 2 of the ^{1}H–^{1}H COSY correlation map of ***N*-methylactinodaphnine** (**15**) recorded in $CDCl_3$ at 500 MHz.

Figure S165. One-bond ^{1}H–^{13}C HSQC correlation map of ***N*-methylactinodaphnine** (**15**) recorded in $CDCl_3$ at 500 MHz (^{1}H) and 125 MHz (^{13}C).

Figure S166. Expanded view 1 of the one-bond ^{1}H–^{13}C HSQC correlation map of ***N*-methylactinodaphnine** (**15**) recorded in $CDCl_3$ at 500 MHz (^{1}H) and 125 MHz (^{13}C).

Figure S167. Expanded view 2 of the one-bond ^{1}H–^{13}C HSQC correlation of ***N*-methylactinodaphnine** (**15**) recorded in $CDCl_3$ at 500 MHz (^{1}H) and 125 MHz (^{13}C).

Figure S168. Long-range ^{1}H–^{13}C HMBC correlation map of ***N*-methylactinodaphnine** (**15**) recorded in $CDCl_3$ at 500 MHz (^{1}H) and 125 MHz (^{13}C).

Figure S169. Expanded view 1 of the long-range ^{1}H–^{13}C HMBC correlation map of ***N*-methylactinodaphnine** (**15**) recorded in $CDCl_3$ at 500 MHz (^{1}H) and 125 MHz (^{13}C).

Figure S170. Expanded view 2 of the long-range ^{1}H–^{13}C HMBC correlation map of ***N*-methylactinodaphnine** (**15**) recorded in $CDCl_3$ at 500 MHz (^{1}H) and 125 MHz (^{13}C).

Figure S171. LR-APCI(+)-MS spectrum of ***N*-methylactinodaphnine** (**15**), showing the protonated molecular ion at *m/z* 326 $[M + H]^+$.

Figure S172. LR-APCI(+)-MS/MS spectrum of the protonated molecule of ***N*-methylactinodaphnine** (**15**) at *m/z* 326 $[M + H]^+$.

Figure S173. FTIR spectrum (KBr) of **(*Z*/*E*)-*N*-formylactinodaphnine** (**16**).

Figure S174. Figure S174. HRESIMS spectrum of **(*Z*/*E*)-*N*-formylactinodaphnine** (**16**), recorded in positive electrospray ionization mode, showing the protonated molecular ion at *m/z* 340.1177 $[M + H]^+$. The observed accurate mass is consistent with the molecular formula $C_{19}H_{17}NO_5$ (calcd for $C_{19}H_{18}NO_5^+$, m/z 340.1185; Δ = −2.3 ppm). The upper panel shows the corresponding base peak chromatogram (BPC), while the lower panel presents the accurate-mass spectrum of compound **16**.

Figure S175. Full ^1H NMR spectrum of **(*Z*/*E*)-*N*-formylactinodaphnine** (**16**) recorded in $CDCl_3$ at 500 MHz. Direct integration of the resolved formyl singlets at δH 8.25 and 8.38 gave normalized values of 1.000 and 0.436, respectively, corresponding to an approximate major:minor ratio of 69.6:30.4 (reported as ca. 70:30). No formal statistical uncertainty was assigned because the estimate derives from a single spectrum rather than replicate quantitative NMR measurements.

Figure S176. Expanded view 1 of the aromatic proton region in the ^1H NMR spectrum of **(*Z*/*E*)-*N*-formylactinodaphnine** (**16**) recorded in $CDCl_3$ at 500 MHz. Where duplicated resonances are resolved, the signal sets should be interpreted together with the major:minor assignment defined from the formyl signals in Figure S175.

Figure S177. Expanded view 2 of the aromatic proton region in the ^1H NMR spectrum of **(*Z*/*E*)-*N*-formylactinodaphnine** (**16**) recorded in $CDCl_3$ at 500 MHz. Where duplicated resonances are resolved, the signal sets should be interpreted together with the major:minor assignment defined from the formyl signals in Figure S175.

Figure S178. Expanded view 3 of the aromatic proton region in the ^1H NMR spectrum of **(*Z*/*E*)-*N*-formylactinodaphnine** (**16**) recorded in $CDCl_3$ at 500 MHz. Where duplicated resonances are resolved, the signal sets should be interpreted together with the major:minor assignment defined from the formyl signals in Figure S175.

Figure S179. Expanded view 4 of the aromatic proton region in the ^1H NMR spectrum of **(*Z*/*E*)-*N*-formylactinodaphnine** (**16**) recorded in $CDCl_3$ at 500 MHz. Where duplicated resonances are resolved, the signal sets should be interpreted together with the major:minor assignment defined from the formyl signals in Figure S175.

Figure S180. Expanded view 5 of the aliphatic proton region in the ^1H NMR spectrum **(*Z*/*E*)-*N*-formylactinodaphnine** (**16**) recorded in $CDCl_3$ at 500 MHz. Where duplicated resonances are resolved, the signal sets should be interpreted together with the major:minor assignment defined from the formyl signals in Figure S175.

Figure S181. Expanded view 6 of the aliphatic proton region in the ^1H NMR spectrum of **(*Z*/*E*)-*N*-formylactinodaphnine** (**16**) recorded in $CDCl_3$ at 500 MHz. Where duplicated resonances are resolved, the signal sets should be interpreted together with the major:minor assignment defined from the formyl signals in Figure S175.

Figure S182. Expanded view 7 of the aliphatic proton region in the ^1H NMR spectrum of **(*Z*/*E*)-*N*-formylactinodaphnine** (**16**) recorded in $CDCl_3$ at 500 MHz. Where duplicated resonances are resolved, the signal sets should be interpreted together with the major:minor assignment defined from the formyl signals in Figure S175.

Figure S183. Expanded view 8 of the aliphatic proton region in the ^1H NMR spectrum of **(*Z*/*E*)-*N*-formylactinodaphnine** (**16**) recorded in $CDCl_3$ at 500 MHz. Where duplicated resonances are resolved, the signal sets should be interpreted together with the major:minor assignment defined from the formyl signals in Figure S175.

Figure S184. Expanded view 9 of the aliphatic proton region in the ^{1}H NMR spectrum of ***(Z/E)-N*-formylactinodaphnine** (**16**) recorded in $CDCl_3$ at 500 MHz. Where duplicated resonances are resolved, the signal sets should be interpreted together with the major:minor assignment defined from the formyl signals in Figure S175.

Figure S185. Expanded view 10 of the aliphatic proton region in the ^{1}H NMR spectrum of ***(Z/E)-N*-formylactinodaphnine** (**16**) recorded in $CDCl_3$ at 500 MHz. Where duplicated resonances are resolved, the signal sets should be interpreted together with the major:minor assignment defined from the formyl signals in Figure S175.

Figure S186. Expanded view 11 of the aliphatic proton region in the ^{1}H NMR spectrum of ***(Z/E)-N*-formylactinodaphnine** (**16**) recorded in $CDCl_3$ at 500 MHz. Where duplicated resonances are resolved, the signal sets should be interpreted together with the major:minor assignment defined from the formyl signals in Figure S175.

Figure S187. Full ^{13}C NMR spectrum of ***(Z/E)-N*-formylactinodaphnine** (**16**) recorded in $CDCl_3$ at 125 MHz.

Figure S188. Expanded view of the carbon region (δ_C 164.1–99.2 ppm) in the ^{13}C NMR spectrum of ***(Z/E)-N*-formylactinodaphnine** (**16**) recorded in $CDCl_3$ at 125 MHz.

Figure S189. Expanded view of the carbon region (δ_C 164.4–162.1 ppm) in the ^{13}C NMR spectrum of ***(Z/E)-N*-formylactinodaphnine** (**16**) recorded in $CDCl_3$ at 125 MHz.

Figure S190. Expanded view of the carbon region (δ_C 147.6–142.2 ppm) in the ^{13}C NMR spectrum of ***(Z/E)-N*-formylactinodaphnine** (**16**) recorded in $CDCl_3$ at 125 MHz.

Figure S191. Expanded view of the carbon region (δ_C 129.7–117.3 ppm) in the ^{13}C NMR spectrum of ***(Z/E)-N*-formylactinodaphnine** (**16**) recorded in $CDCl_3$ at 125 MHz.

Figure S192. Expanded view of the carbon region (δ_C 118.7–100.5 ppm) in the ^{13}C NMR spectrum of ***(Z/E)-N*-formylactinodaphnine** (**16**) recorded in $CDCl_3$ at 125 MHz.

Figure S193. Expanded view of the carbon region (δ_C 57.4–30.0 ppm) in the ^{13}C NMR spectrum of ***(Z/E)-N*-formylactinodaphnine** (**16**) recorded in $CDCl_3$ at 125 MHz.

Figure S194. Expanded view of the carbon region (δ_C 56.6–53.5 ppm) in the ^{13}C NMR spectrum of ***(Z/E)-N*-formylactinodaphnine** (**16**) recorded in $CDCl_3$ at 125 MHz.

Figure S195. Expanded view of the carbon region (δ_C 43.4–29.4 ppm) in the ^{13}C NMR spectrum of ***(Z/E)-N*-formylactinodaphnine** (**16**) recorded in $CDCl_3$ at 125 MHz.

Figure S196. Full DEPT-135 ^{13}C NMR spectrum of ***(Z/E)-N*-formylactinodaphnine** (**16**) recorded in $CDCl_3$ at 125 MHz.

Figure S197. Expanded view of the carbon region (δ_C 166.2–99.3 ppm) in the DEPT-135 ^{13}C NMR spectrum of ***(Z/E)-N*-formylactinodaphnine** (**16**) recorded in $CDCl_3$ at 125 MHz.

Figure S198. Expanded view of the carbon region (δ_C 166.4–162.2 ppm) in the DEPT-135 ^{13}C NMR spectrum of ***(Z/E)-N*-formylactinodaphnine** (**16**) recorded in $CDCl_3$ at 125 MHz.

Figure S199. Expanded view of the carbon region (δ_C 115.6–100.8 ppm) in the DEPT-135 ^{13}C NMR spectrum of ***(Z/E)-N*-formylactinodaphnine** (**16**) recorded in $CDCl_3$ at 125 MHz.

Figure S200. Expanded view of the carbon region (δ_C 57.4–29.6 ppm) in the DEPT-135 ^{13}C NMR spectrum of ***(Z/E)-N*-formylactinodaphnine** (**16**) recorded in $CDCl_3$ at 125 MHz.

Figure S201. Expanded view of the carbon region (δ_C 56.5–53.3 ppm) in the DEPT-135 ^{13}C NMR spectrum of ***(Z/E)-N*-formylactinodaphnine** (**16**) recorded in $CDCl_3$ at 125 MHz.

Figure S202. ^{1}H–^{1}H COSY correlation map ***(Z/E)-N*-formylactinodaphnine** (**16**) recorded in $CDCl_3$ at 500 MHz.

Figure S203. Expanded view 1 of the ^{1}H–^{1}H COSY correlation map of **(*Z*/*E*)-*N*-formylactinodaphnine** (**16**) recorded in $CDCl_3$ at 500 MHz.

Figure S204. Expanded view 2 of the ^{1}H–^{1}H COSY correlation map of **(*Z*/*E*)-*N*-formylactinodaphnine** (**16**) recorded in $CDCl_3$ at 500 MHz.

Figure S205. Expanded view 3 of the ^{1}H–^{1}H COSY correlation map of **(*Z*/*E*)-*N*-formylactinodaphnine** (**16**) recorded in $CDCl_3$ at 500 MHz.

Figure S206. Expanded view 4 of the ^{1}H–^{1}H COSY correlation map of **(*Z*/*E*)-*N*-formylactinodaphnine** (**16**) recorded in $CDCl_3$ at 500 MHz.

Figure S207. Expanded view 5 of the ^{1}H–^{1}H COSY correlation map of **(*Z*/*E*)-*N*-formylactinodaphnine** (**16**) recorded in $CDCl_3$ at 500 MHz.

Figure S208. One-bond ^{1}H–^{13}C HSQC correlation map of **(*Z*/*E*)-*N*-formylactinodaphnine** (**16**) recorded in $CDCl_3$ at 500 MHz (^{1}H) and 125 MHz (^{13}C).

Figure S209. Expanded view 1 of the one-bond ^{1}H–^{13}C HSQC correlation map of **(*Z*/*E*)-*N*-formylactinodaphnine** (**16**) recorded in $CDCl_3$ at 500 MHz (^{1}H) and 125 MHz (^{13}C).

Figure S210. Expanded view 2 of the one-bond ^{1}H–^{13}C HSQC correlation map of **(*Z*/*E*)-*N*-formylactinodaphnine** (**16**) recorded in $CDCl_3$ at 500 MHz (^{1}H) and 125 MHz (^{13}C).

Figure S211. Expanded view 3 of the one-bond ^{1}H–^{13}C HSQC correlation map of **(*Z*/*E*)-*N*-formylactinodaphnine** (**16**) recorded in $CDCl_3$ at 500 MHz (^{1}H) and 125 MHz (^{13}C).

Figure S212. Expanded view 4 of the one-bond ^{1}H–^{13}C HSQC correlation map of **(*Z*/*E*)-*N*-formylactinodaphnine** (**16**) recorded in $CDCl_3$ at 500 MHz (^{1}H) and 125 MHz (^{13}C).

Figure S213. Expanded view 5 of the one-bond ^{1}H–^{13}C HSQC correlation map of **(*Z*/*E*)-*N*-formylactinodaphnine** (**16**) recorded in $CDCl_3$ at 500 MHz (^{1}H) and 125 MHz (^{13}C).

Figure S214. Expanded view 6 of the one-bond ^{1}H–^{13}C HSQC correlation map of **(*Z*/*E*)-*N*-formylactinodaphnine** (**16**) recorded in $CDCl_3$ at 500 MHz (^{1}H) and 125 MHz (^{13}C).

Figure S215. Expanded view 7 of the one-bond ^{1}H–^{13}C HSQC correlation map of **(*Z*/*E*)-*N*-formylactinodaphnine** (**16**) recorded in $CDCl_3$ at 500 MHz (^{1}H) and 125 MHz (^{13}C).

Figure S216. Expanded view 8 of the one-bond ^{1}H–^{13}C HSQC correlation map of **(*Z*/*E*)-*N*-formylactinodaphnine** (**16**) recorded in $CDCl_3$ at 500 MHz (^{1}H) and 125 MHz (^{13}C).

Figure S217. Long-range ^{1}H–^{13}C HMBC correlation map of **(*Z*/*E*)-*N*-formylactinodaphnine** (**16**) recorded in $CDCl_3$ at 500 MHz (^{1}H) and 125 MHz (^{13}C).

Figure S218. Expanded view 1 of the long-range ^{1}H–^{13}C HMBC correlation map of **(*Z*/*E*)-*N*-formylactinodaphnine** (**16**) recorded in $CDCl_3$ at 500 MHz (^{1}H) and 125 MHz (^{13}C).

Figure S219. Expanded view 2 of the long-range ^{1}H–^{13}C HMBC correlation map of **(*Z*/*E*)-*N*-formylactinodaphnine** (**16**) recorded in $CDCl_3$ at 500 MHz (^{1}H) and 125 MHz (^{13}C).

Figure S220. Expanded view 3 of the long-range ^{1}H–^{13}C HMBC correlation map of **(*Z*/*E*)-*N*-formylactinodaphnine** (**16**) recorded in $CDCl_3$ at 500 MHz (^{1}H) and 125 MHz (^{13}C).

Figure S221. Expanded view 4 of the long-range ^{1}H–^{13}C HMBC correlation map of **(*Z*/*E*)-*N*-formylactinodaphnine** (**16**) recorded in $CDCl_3$ at 500 MHz (^{1}H) and 125 MHz (^{13}C).

Figure S222. Expanded view 5 of the long-range ^{1}H–^{13}C HMBC correlation map of **(*Z*/*E*)-*N*-formylactinodaphnine** (**16**) recorded in $CDCl_3$ at 500 MHz (^{1}H) and 125 MHz (^{13}C).

Figure S223. Expanded view 6 of the long-range ^{1}H–^{13}C HMBC correlation map of **(*Z*/*E*)-*N*-formylactinodaphnine** (**16**) recorded in $CDCl_3$ at 500 MHz (^{1}H) and 125 MHz (^{13}C).

Figure S224. Expanded view 7 of the long-range ^{1}H–^{13}C HMBC correlation map of **(*Z*/*E*)-*N*-formylactinodaphnine** (**16**) recorded in $CDCl_3$ at 500 MHz (^{1}H) and 125 MHz (^{13}C).

Figure S225. Expanded view 8 of the long-range ^{1}H–^{13}C HMBC correlation map of **(*Z*/*E*)-*N*-formylactinodaphnine** (**16**) recorded in $CDCl_3$ at 500 MHz (^{1}H) and 125 MHz (^{13}C).

Figure S226. Expanded view 9 of the long-range ^{1}H–^{13}C HMBC correlation map of **(*Z*/*E*)-*N*-formylactinodaphnine** (**16**) recorded in $CDCl_3$ at 500 MHz (^{1}H) and 125 MHz (^{13}C).

Figure S227. Expanded view 10 of the long-range ^{1}H–^{13}C HMBC correlation map of **(*Z*/*E*)-*N*-formylactinodaphnine** (**16**) recorded in $CDCl_3$ at 500 MHz (^{1}H) and 125 MHz (^{13}C).

Figure S228. Expanded view 11 of the long-range ^{1}H–^{13}C HMBC correlation map of **(*Z*/*E*)-*N*-formylactinodaphnine** (**16**) recorded in $CDCl_3$ at 500 MHz (^{1}H) and 125 MHz (^{13}C).

Figure S229. Two-dimensional ^{1}H–^{1}H NOESY spectrum of **(*Z*/*E*)-*N*-formylactinodaphnine** (**16**) recorded in $CDCl_3$ at 500 MHz. Diagnostic regions are expanded and annotated in Figures S230 and S231.

Figure S230. Expanded and annotated region of the two-dimensional ^{1}H–^{1}H NOESY spectrum of **(*Z*/*E*)-*N*-formylactinodaphnine** (**16**) recorded in $CDCl_3$ at 500 MHz. Diagnostic spatial correlations used for the tentative conformational assignments are δ_H 8.25 (CHO)/3.80 (H-5) for the major *Z*-assigned state and δ_H 8.38 (CHO)/4.58 (H-6a) and δ_H 8.38 (CHO)/2.74 (H-7) for the minor *E*-assigned state.

Figure S231. Additional expanded NOESY region of **(*Z*/*E*)-*N*-formylactinodaphnine** (**16**) recorded in $CDCl_3$ at 500 MHz. The NOESY correlations support spatial assignments only and do not constitute direct evidence of chemical exchange between the two NMR states.

Figure S232. LR-APCI(−)-MS spectrum of **(*Z*/*E*)-*N*-formylactinodaphnine** (**16**), showing the deprotonated molecular ion at *m/z* 338 $[M - H]^-$.

Figure S233. LR-APCI(−)-MS/MS spectrum of the deprotonated molecule of **(*Z*/*E*)-*N*-formylactinodaphnine** (**16**) at *m/z* 338 $[M - H]^-$.

Figure S234. Full ^{1}H NMR spectrum of **discretamine** (**17**) recorded in $CDCl_3$ with a drop of CD_3OD at 500 MHz.

Figure S235. Expanded view 1 of the aromatic proton region in the ^{1}H NMR spectrum of **discretamine** (**17**) recorded in $CDCl_3$ with a drop of CD_3OD at 500 MHz.

Figure S236. Expanded view 2 of the aliphatic proton region in the ^{1}H NMR spectrum of **discretamine** (**17**) recorded in $CDCl_3$ with a drop of CD_3OD at 500 MHz.

Figure S237. Full ^{13}C NMR spectrum of **discretamine** (**17**) recorded in $CDCl_3$ with a drop of CD_3OD at 125 MHz.

Figure S238. Expanded view of the carbon region (δ_C 148.2–106.7 ppm) in the ^{13}C NMR spectrum of **discretamine** (**17**) recorded in $CDCl_3$ with a drop of CD_3OD at 125 MHz.

Figure S239. Expanded view of the carbon region (δ_C 62.0–27.5 ppm) in the ^{13}C NMR spectrum of **discretamine** (**17**) recorded in $CDCl_3$ with a drop of CD_3OD at 125 MHz.

Figure S240. Full DEPT-135 ^{13}C NMR spectrum of **discretamine** (**17**) recorded in $CDCl_3$ with a drop of CD_3OD at 125 MHz.

Figure S241. Expanded view of the carbon region (δ_C 125.5–107.6 ppm) in the DEPT-135 ^{13}C NMR spectrum of **discretamine** (**17**) recorded in $CDCl_3$ with a drop of CD_3OD at 125 MHz.

Figure S242. Expanded view of the carbon region (δ_C 61.7–26.7 ppm) in the DEPT-135 ^{13}C NMR spectrum of **discretamine** (**17**) recorded in $CDCl_3$ with a drop of CD_3OD at 125 MHz.

Figure S243. ^{1}H–^{1}H COSY correlation map of **discretamine** (**17**) recorded in $CDCl_3$ with a drop of CD_3OD at 500 MHz.

Figure S244. Expanded view 1 of the ^{1}H–^{1}H COSY correlation map of **discretamine** (**17**) recorded in $CDCl_3$ with a drop of CD_3OD at 500 MHz.

Figure S245. Expanded view 2 of the ^{1}H–^{1}H COSY correlation map of **discretamine** (**17**) recorded in $CDCl_3$ with a drop of CD_3OD at 500 MHz.

Figure S246. One-bond ^{1}H–^{13}C HSQC correlation map of **discretamine** (**17**) recorded in $CDCl_3$ with a drop of CD_3OD at 500 MHz (^{1}H) and 125 MHz (^{13}C).

Figure S247. Expanded view 1 of the one-bond ^{1}H–^{13}C HSQC correlation map of **discretamine** (**17**) recorded in $CDCl_3$ with a drop of CD_3OD at 500 MHz (^{1}H) and 125 MHz (^{13}C).

Figure S248. Expanded view 2 of the one-bond ^{1}H–^{13}C HSQC correlation of **discretamine** (**17**) recorded in $CDCl_3$ with a drop of CD_3OD at 500 MHz (^{1}H) and 125 MHz (^{13}C).

Figure S249. Long-range ^{1}H–^{13}C HMBC correlation map of **discretamine** (**17**) recorded in $CDCl_3$ with a drop of CD_3OD at 500 MHz (^{1}H) and 125 MHz (^{13}C).

Figure S250. Expanded view 1 of the long-range ^{1}H–^{13}C HMBC correlation map of **discretamine** (**17**) recorded in $CDCl_3$ with a drop of CD_3OD at 500 MHz (^{1}H) and 125 MHz (^{13}C).

Figure S251. Expanded view 2 of the long-range ^{1}H–^{13}C HMBC correlation map of **discretamine** (**17**) recorded in $CDCl_3$ with a drop of CD_3OD at 500 MHz (^{1}H) and 125 MHz (^{13}C).

Figure S252. LR-APCI(+)-MS spectrum of **discretamine** (**17**), showing the protonated molecular ion at *m/z* 328 $[M + H]^{+}$.

Figure S253. LR-APCI(+)-MS/MS spectrum of the protonated molecule of **discretamine** (**17**) at *m/z* 328 $[M + H]^{+}$.

Figure S254. Full ^{1}H NMR spectrum of **10-demethyldiscretine** (**18**) recorded in $CDCl_3$ with a drop of CD_3OD at 500 MHz.

Figure S255. Expanded view 1 of the aromatic proton region in the ^{1}H NMR spectrum of **10-demethyldiscretine** (**18**) recorded in $CDCl_3$ with a drop of CD_3OD at 500 MHz.

Figure S256. Expanded view 2 of the aliphatic proton region in the ^{1}H NMR spectrum of **10-demethyldiscretine** (**18**) recorded in $CDCl_3$ with a drop of CD_3OD at 500 MHz.

Figure S257. Full ^{13}C NMR spectrum of **10-demethyldiscretine** (**18**) recorded in $CDCl_3$ with a drop of CD_3OD at 125 MHz.

Figure S258. Expanded view of the carbon region (δ_C 147.1–106.9 ppm) in the ^{13}C NMR spectrum of **10-demethyldiscretine** (**18**) recorded in $CDCl_3$ with a drop of CD_3OD at 125 MHz.

Figure S259. Expanded view of the carbon region (δ_C 61.0–27.5 ppm) in the ^{13}C NMR spectrum of **10-demethyldiscretine** (**18**) recorded in $CDCl_3$ with a drop of CD_3OD at 125 MHz.

Figure S260. Full DEPT-135 ^{13}C NMR spectrum of **10-demethyldiscretine** (**18**) recorded in $CDCl_3$ with a drop of CD_3OD at 125 MHz.

Figure S261. Expanded view of the carbon region (δ_C 114.9–107.3 ppm) in the DEPT-135 ^{13}C NMR spectrum of **10-demethyldiscretine** (**18**) recorded in $CDCl_3$ with a drop of CD_3OD at 125 MHz.

Figure S262. Expanded view of the carbon region (δ_C 61.4–27.5 ppm) in the DEPT-135 ^{13}C NMR spectrum of **10-demethyldiscretine** (**18**) recorded in $CDCl_3$ with a drop of CD_3OD at 125 MHz.

Figure S263. ^{1}H–^{1}H COSY correlation map of **10-demethyldiscretine** (**18**) recorded in $CDCl_3$ with a drop of CD_3OD at 500 MHz.

Figure S264. Expanded view 1 of the ^{1}H–^{1}H COSY correlation map of **10-demethyldiscretine** (**18**) recorded in $CDCl_3$ with a drop of CD_3OD at 500 MHz.

Figure S265. Expanded view 2 of the ^{1}H–^{1}H COSY correlation map of **10-demethyldiscretine** (**18**) recorded in $CDCl_3$ with a drop of CD_3OD at 500 MHz.

Figure S266. One-bond ^{1}H–^{13}C HSQC correlation map of **10-demethyldiscretine** (**18**) recorded in $CDCl_3$ with a drop of CD_3OD at 500 MHz (^{1}H) and 125 MHz (^{13}C).

Figure S267. Expanded view 1 of the one-bond ^{1}H–^{13}C HSQC correlation map of **10-demethyldiscretine** (**18**) recorded in $CDCl_3$ with a drop of CD_3OD at 500 MHz (^{1}H) and 125 MHz (^{13}C).

Figure S268. Expanded view 2 of the one-bond ^{1}H–^{13}C HSQC correlation of **10-demethyldiscretine** (**18**) recorded in $CDCl_3$ with a drop of CD_3OD at 500 MHz (^{1}H) and 125 MHz (^{13}C).

Figure S269. Long-range ^{1}H–^{13}C HMBC correlation map of **10-demethyldiscretine** (**18**) recorded in $CDCl_3$ with a drop of CD_3OD at 500 MHz (^{1}H) and 125 MHz (^{13}C).

Figure S270. Expanded view 1 of the long-range ^{1}H–^{13}C HMBC correlation map of **10-demethyldiscretine** (**18**) recorded in $CDCl_3$ with a drop of CD_3OD at 500 MHz (^{1}H) and 125 MHz (^{13}C).

Figure S271. Expanded view 2 of the long-range ^{1}H–^{13}C HMBC correlation map of **10-demethyldiscretine** (**18**) recorded in $CDCl_3$ with a drop of CD_3OD at 500 MHz (^{1}H) and 125 MHz (^{13}C).

Figure S272. LR-APCI(+)-MS spectrum of **10-demethyldiscretine** (**18**), showing the protonated molecular ion at *m/z* 328 $[M + H]^+$.

Figure S273. LR-APCI(+)-MS/MS spectrum of the protonated molecule of **10-demethyldiscretine** (**18**) at *m/z* 328 $[M + H]^+$.

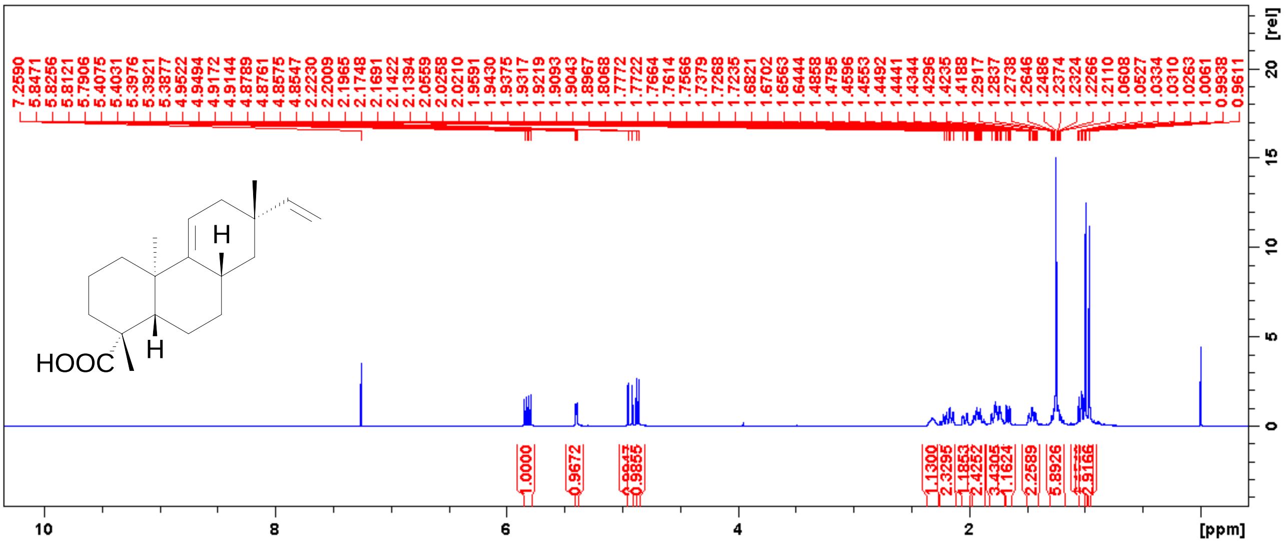

Figure S1. Full ^{1}H NMR spectrum of **acanthoic acid** (**1**) recorded in $CDCl_3$ at 500 MHz.

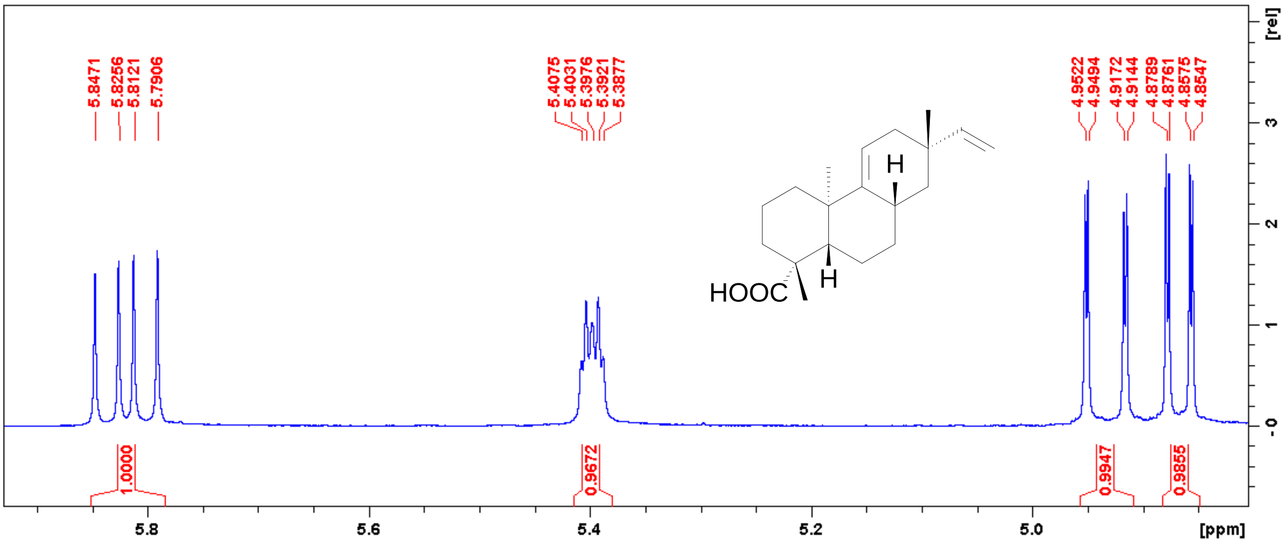

Figure S2. Expanded view of the olefinic proton region in the ^{1}H NMR spectrum of **acanthoic acid** (**1**) recorded in $CDCl_3$ at 500 MHz.

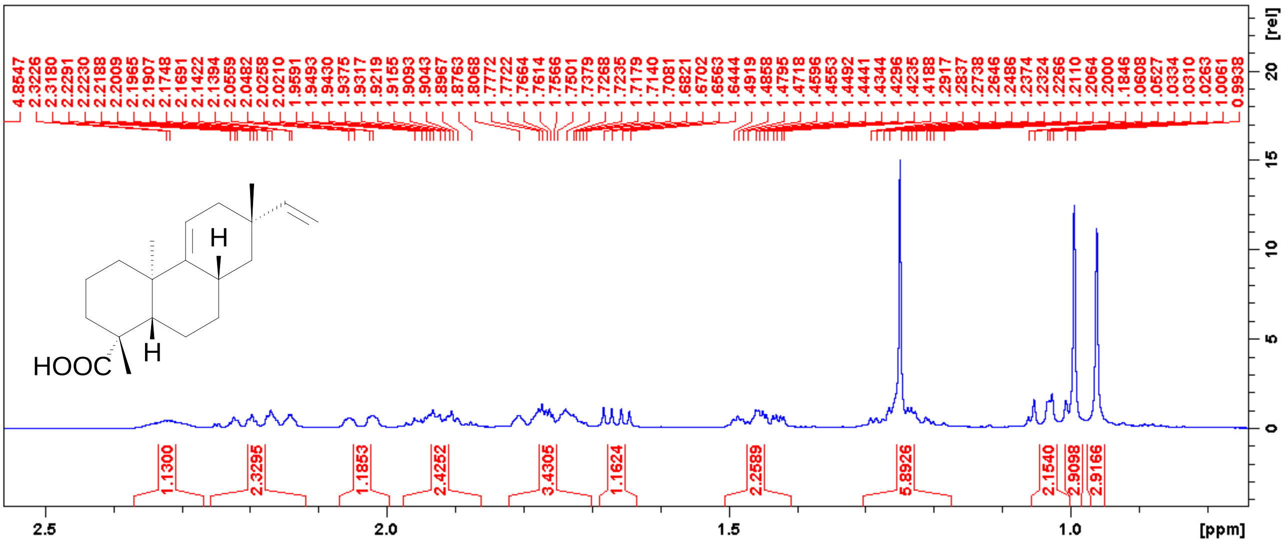

Figure S3. Expanded view of the aliphatic proton region in the ^{1}H NMR spectrum of **acanthoic acid** (**1**) recorded in $CDCl_3$ at 500 MHz.

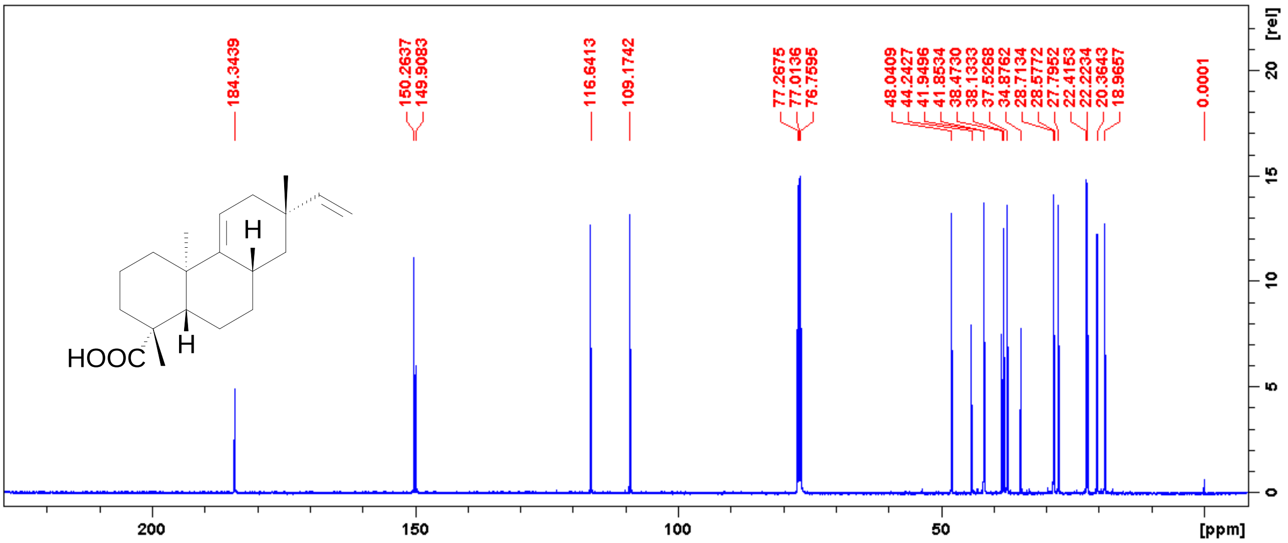

Figure S4. Full ^{13}C NMR spectrum of **acanthoic acid** (**1**) recorded in $CDCl_3$ at 125 MHz.

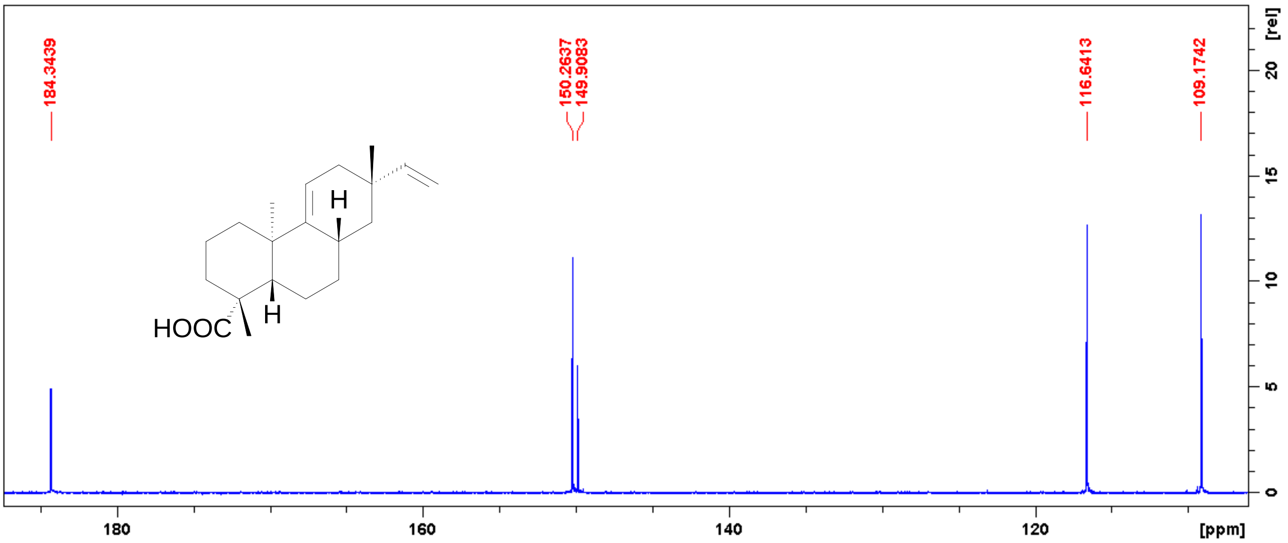

Figure S5. Expanded view of the carbon region (δ_C 187.2–106.3 ppm) in the ^{13}C NMR spectrum of **acanthoic acid** (**1**) recorded in $CDCl_3$ at 125 MHz.

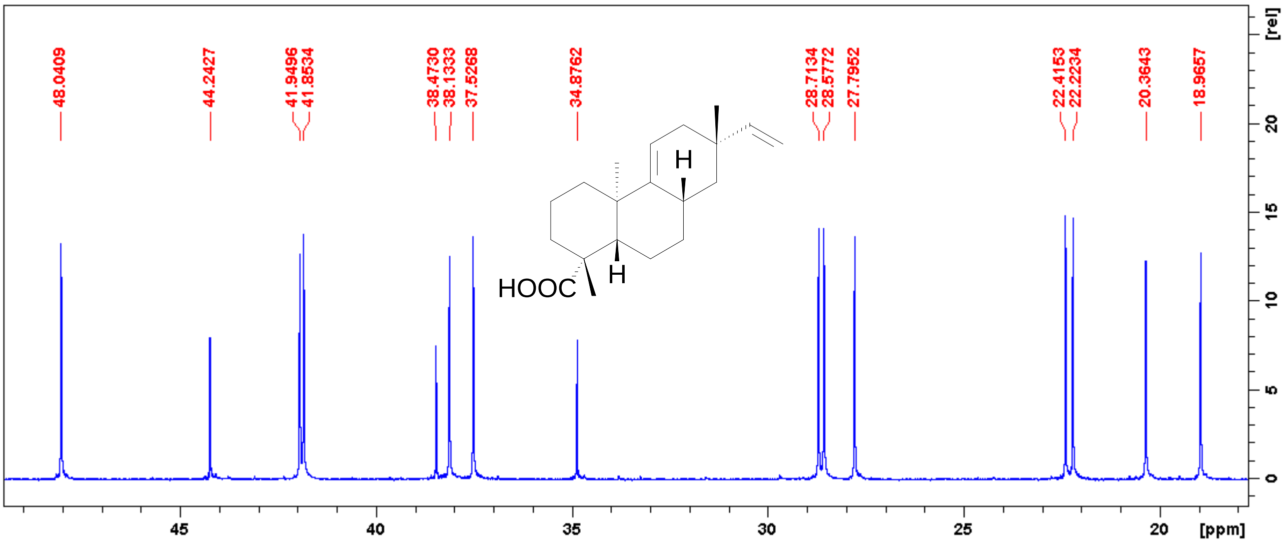

Figure S6. Expanded view of the carbon region (δ_C 49.3–17.8 ppm) in the ^{13}C NMR spectrum of **acanthoic acid** (**1**) recorded in $CDCl_3$ at 125 MHz.

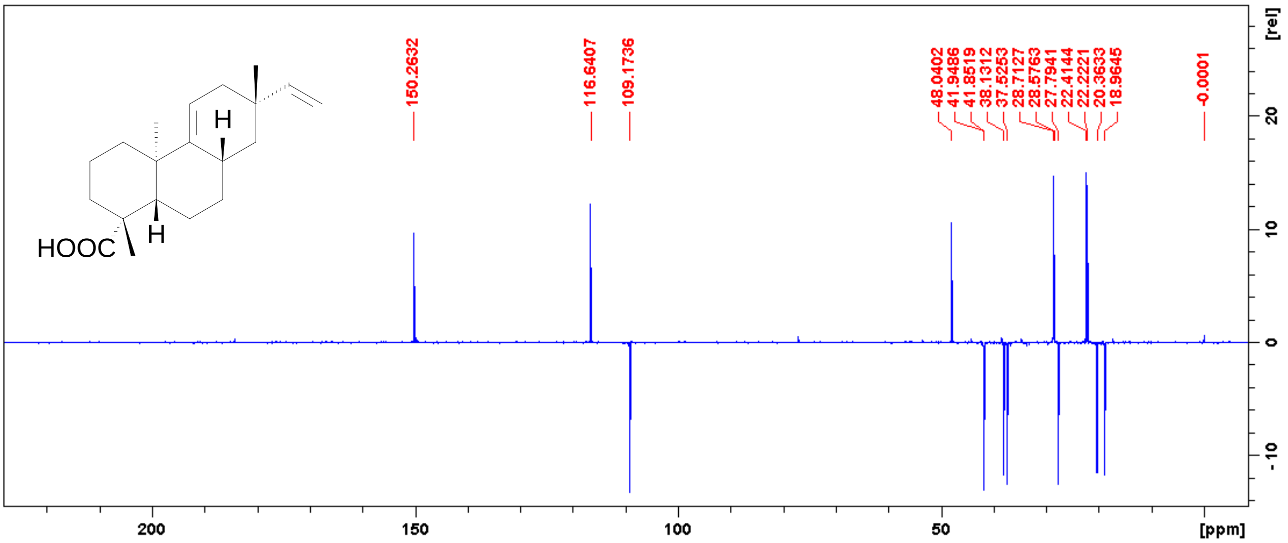

Figure S7. Full DEPT-135 ^{13}C NMR spectrum of **acanthoic acid** (**1**) recorded in $CDCl_3$ at 125 MHz.

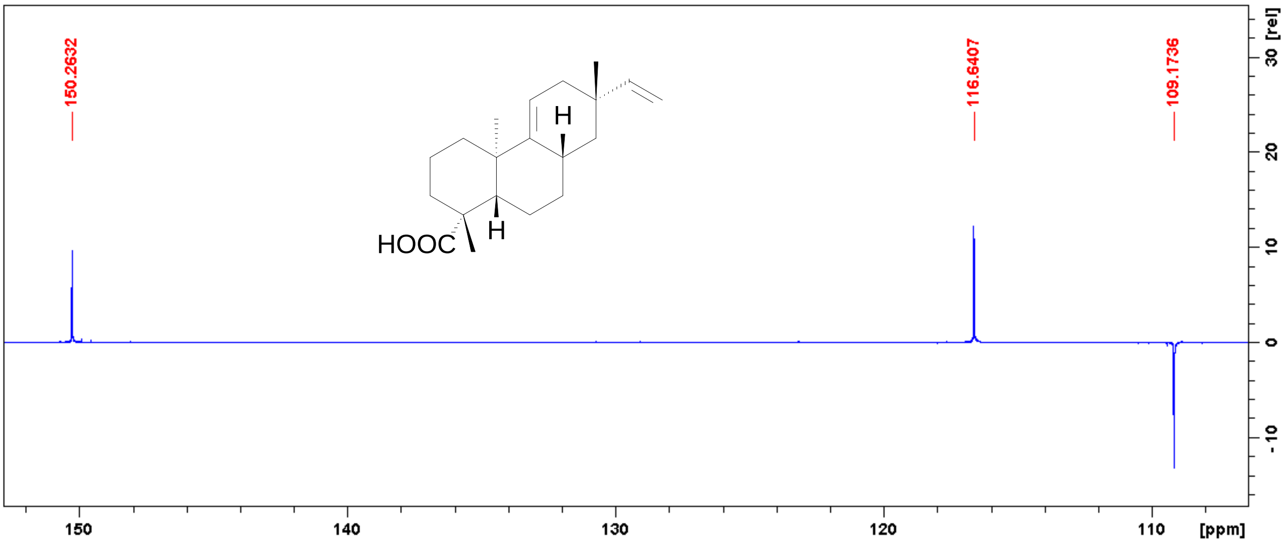

Figure S8. Expanded view of the carbon region (δ_C 152.7–106.5 ppm) in the DEPT-135 ^{13}C NMR spectrum of **acanthoic acid** (**1**) recorded in $CDCl_3$ at 125 MHz.

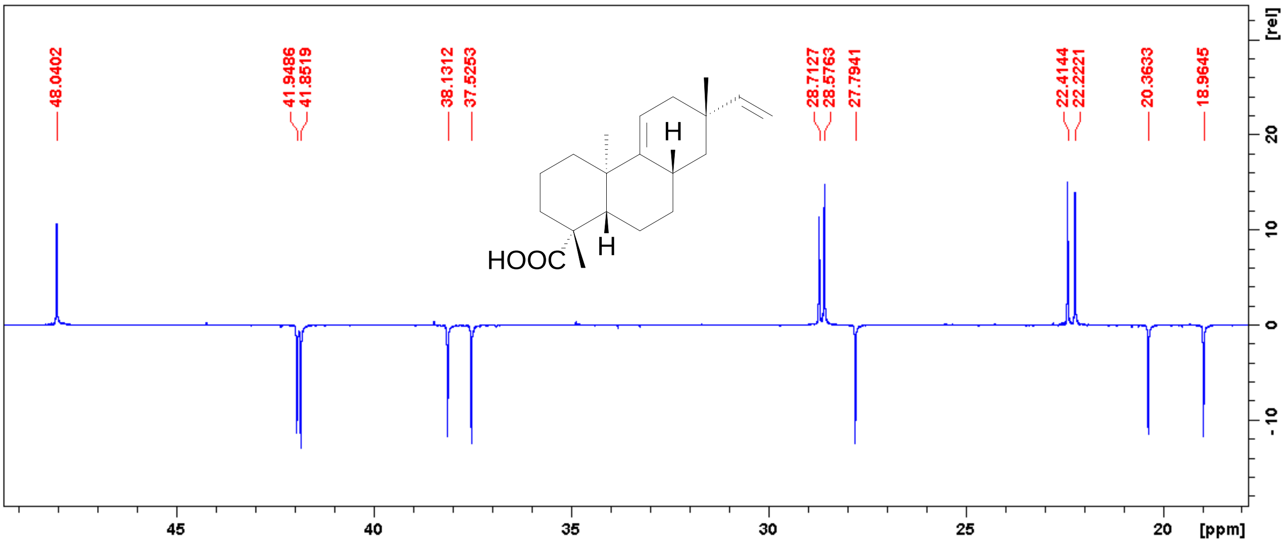

Figure S9. Expanded view of the carbon region (δ_C 49.3–17.9 ppm) in the DEPT-135 ^{13}C NMR spectrum of **acanthoic acid** (**1**) recorded in $CDCl_3$ at 125 MHz.

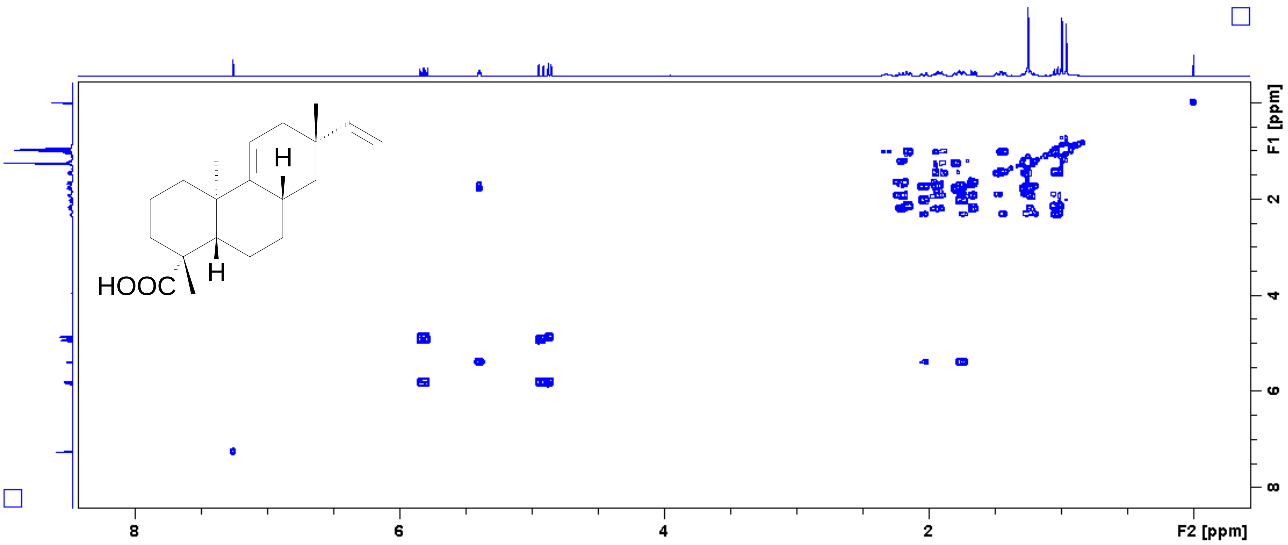

Figure S10. ^{1}H–^{1}H COSY correlation map of **acanthoic acid** (**1**) recorded in $CDCl_3$ at 500 MHz.

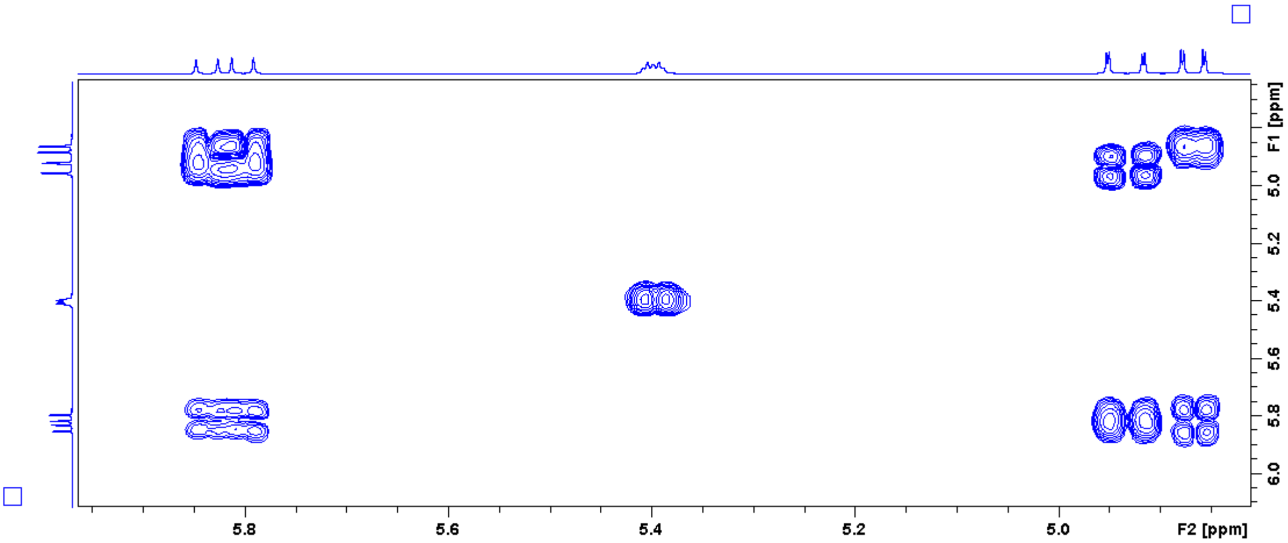

Figure S11. Expanded view 1 of the ^{1}H–^{1}H COSY correlation map of **acanthoic acid** (**1**) recorded in $CDCl_3$ at 500 MHz.

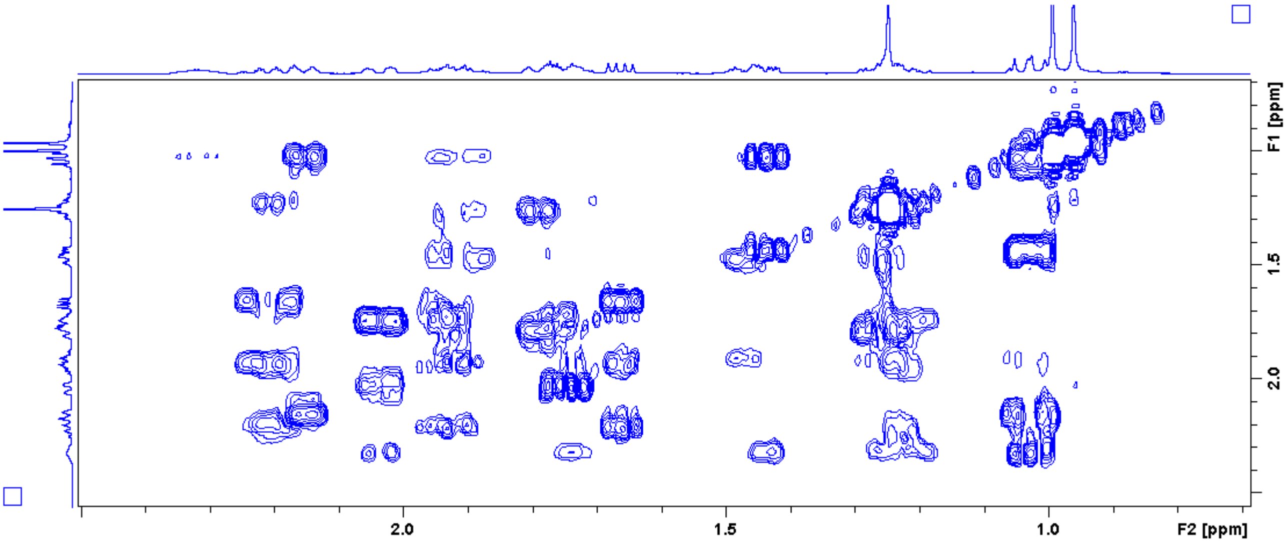

Figure S12. Expanded view 2 of the ^{1}H–^{1}H COSY correlation map of **acanthoic acid** (**1**) recorded in $CDCl_3$ at 500 MHz.

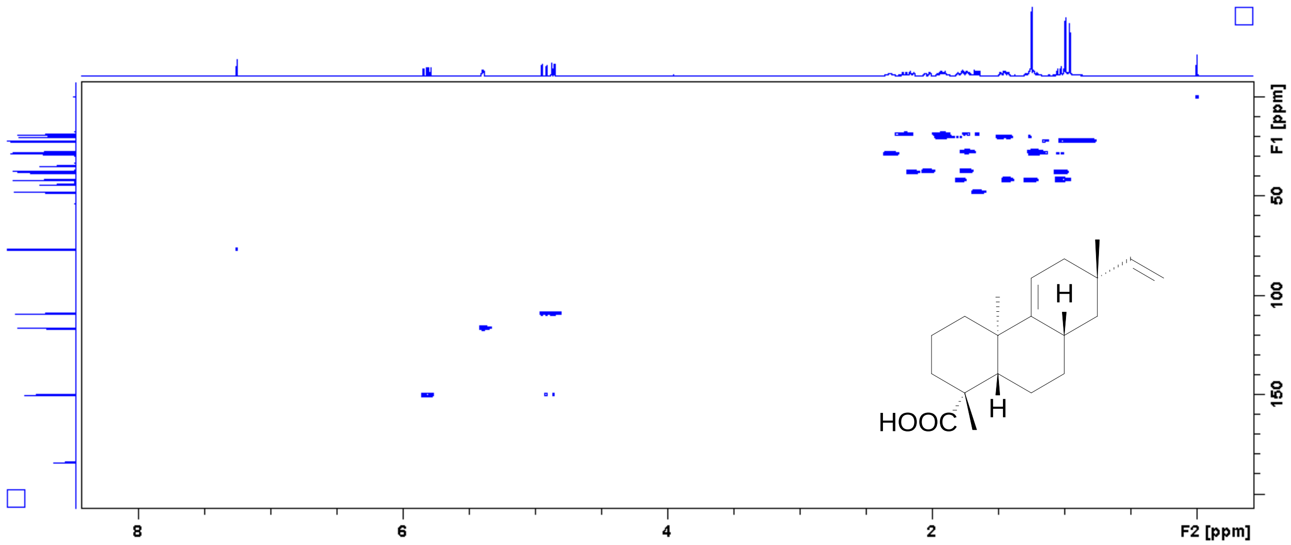

Figure S13. One-bond ^1H–^{13}C HSQC correlation map of **acanthoic acid** (**1**) recorded in $CDCl_3$ at 500 MHz (^1H) and 125 MHz (^{13}C).

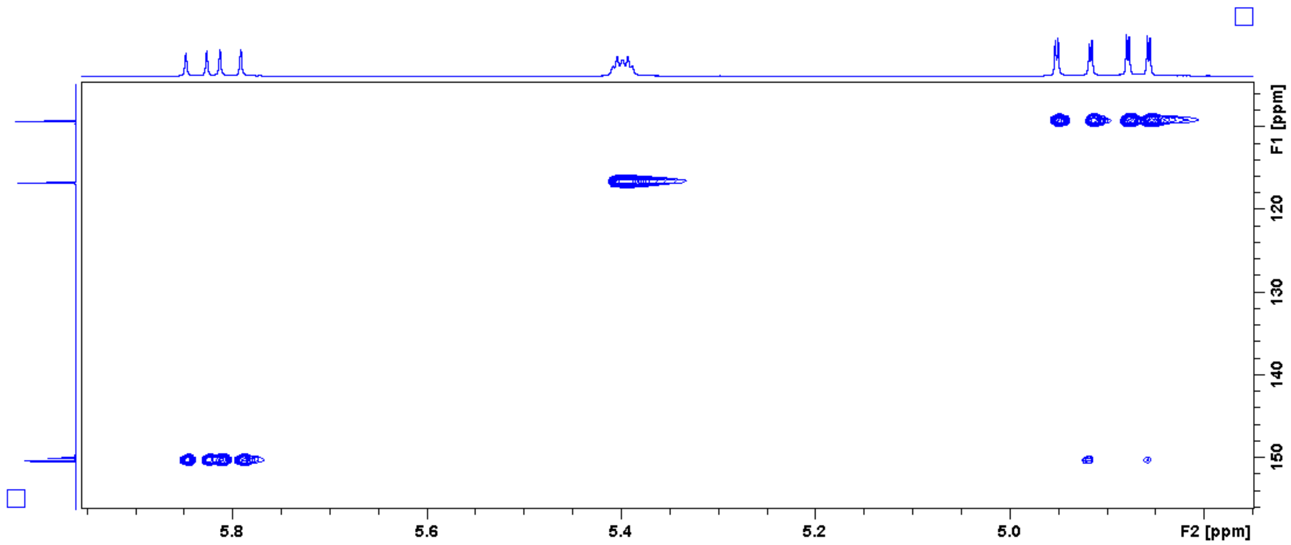

Figure S14. Expanded view 1 of the one-bond ^1H–^{13}C HSQC correlation map of **acanthoic acid** (**1**) recorded in $CDCl_3$ at 500 MHz (^1H) and 125 MHz (^{13}C).

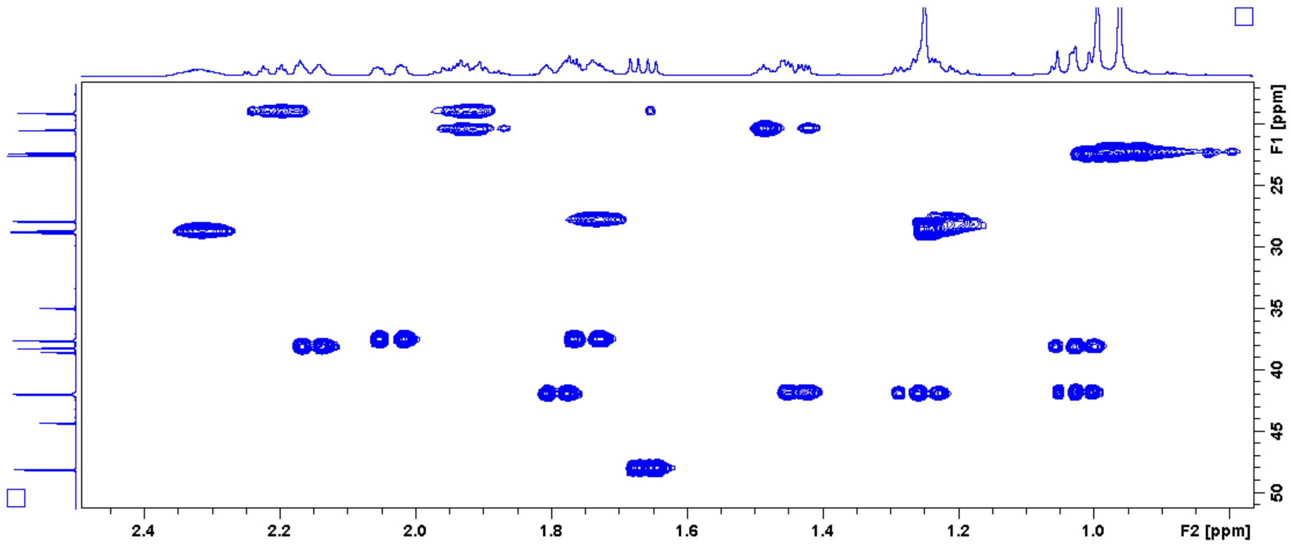

Figure S15. Expanded view 2 of the one-bond ^1H–^{13}C HSQC correlation map of **acanthoic acid** (**1**) recorded in $CDCl_3$ at 500 MHz (^1H) and 125 MHz (^{13}C).

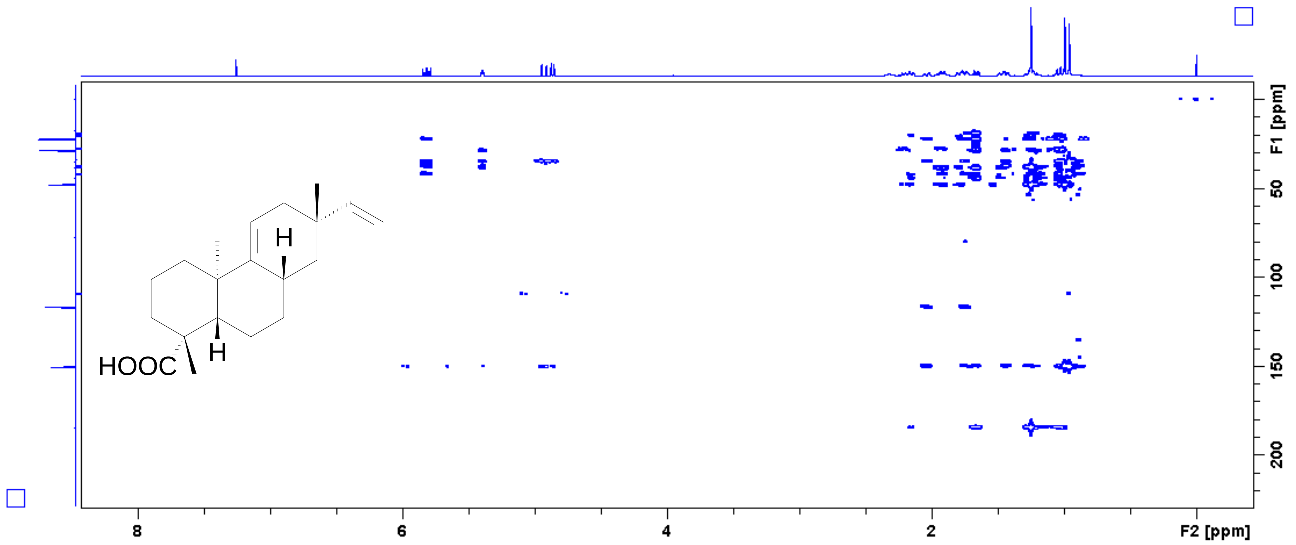

Figure S16. Long-range ^{1}H–^{13}C HMBC correlation map of **acanthoic acid** (**1**) recorded in $CDCl_3$ at 500 MHz (^{1}H) and 125 MHz (^{13}C).

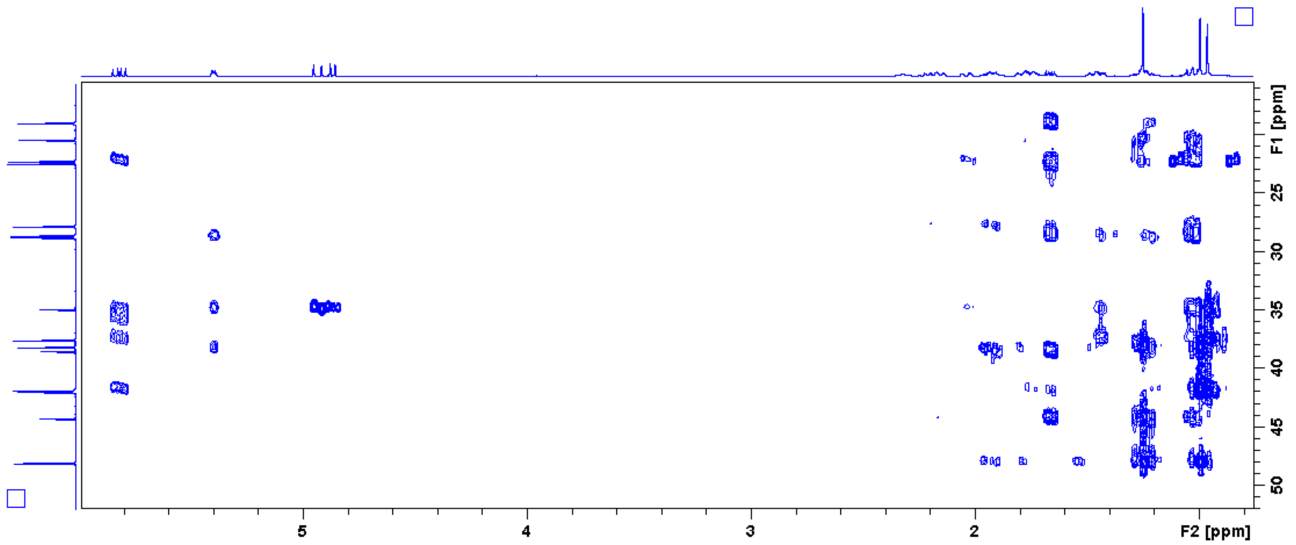

Figure S17. Expanded view 1 of the long-range ^{1}H–^{13}C HMBC correlation map of **acanthoic acid** (**1**) recorded in $CDCl_3$ at 500 MHz (^{1}H) and 125 MHz (^{13}C).

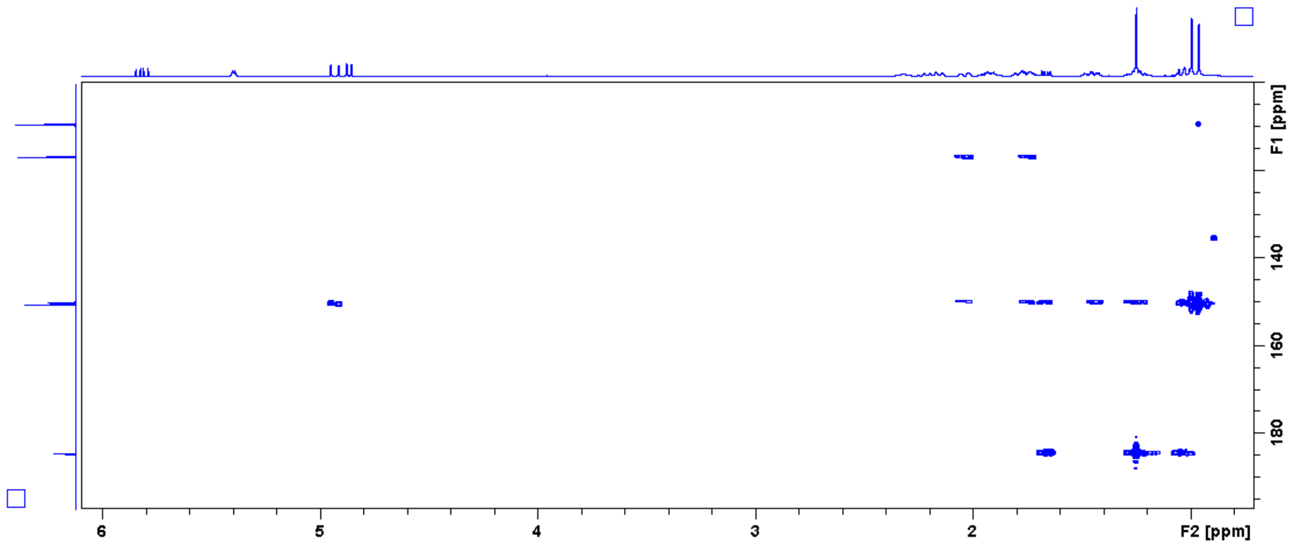

Figure S18. Expanded view 2 of the long-range ^{1}H–^{13}C HMBC correlation map of **acanthoic acid** (**1**) recorded in $CDCl_3$ at 500 MHz (^{1}H) and 125 MHz (^{13}C).

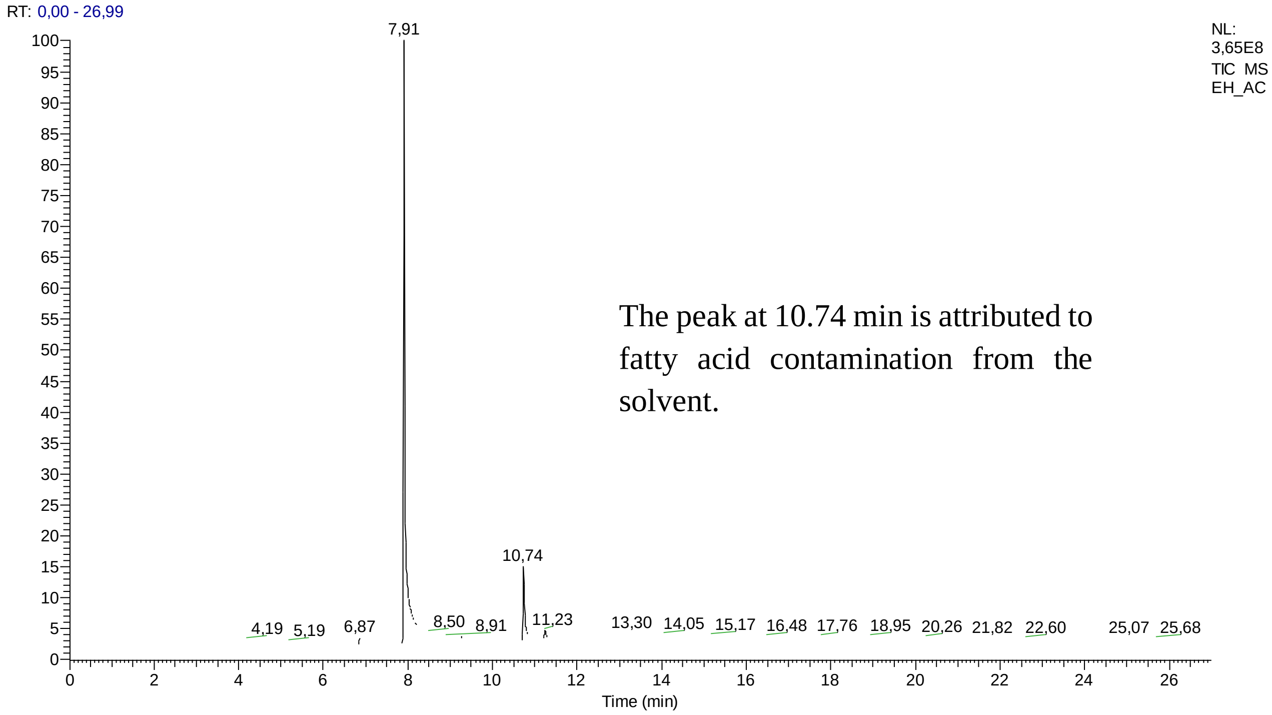

Figure S19. Total ion chromatogram (GC–MS) of **acanthoic acid** (**1**) with a retention time of 7.91 min.

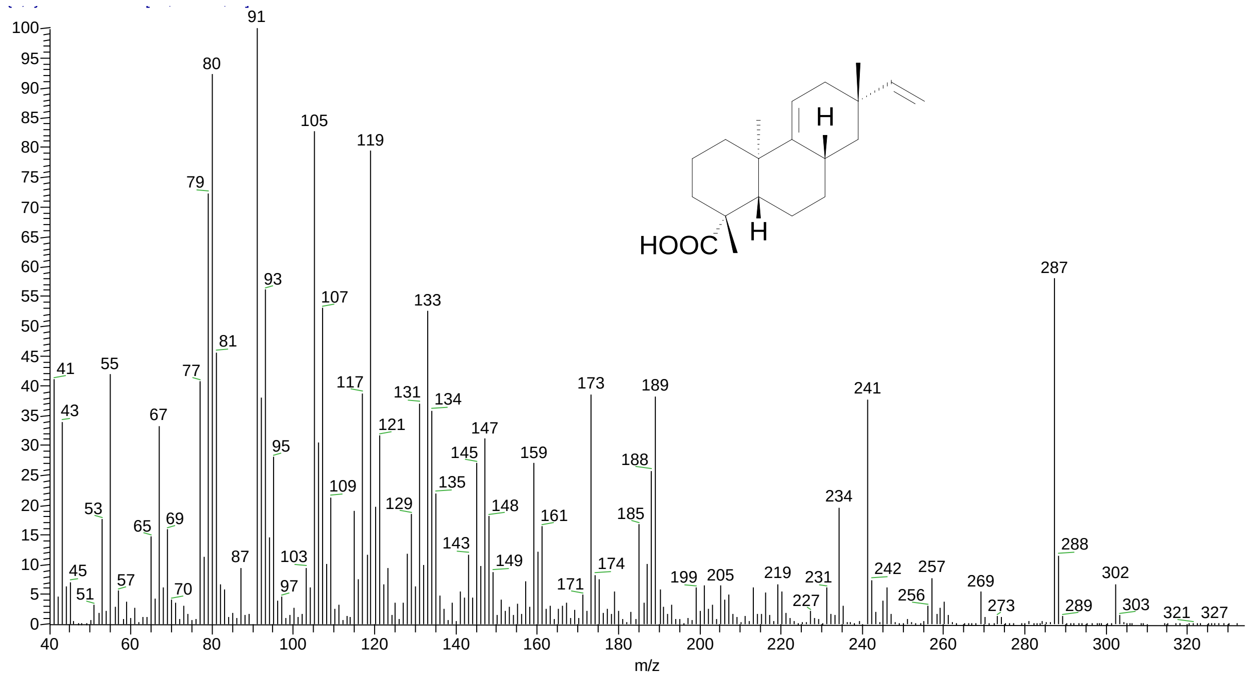

Figure S20. Mass spectrum of **acanthoic acid** (**1**), corresponding to the chromatographic peak at a retention time of 7.91 min, showing the molecular ion at m/z 302 ($M^{+\bullet}$).

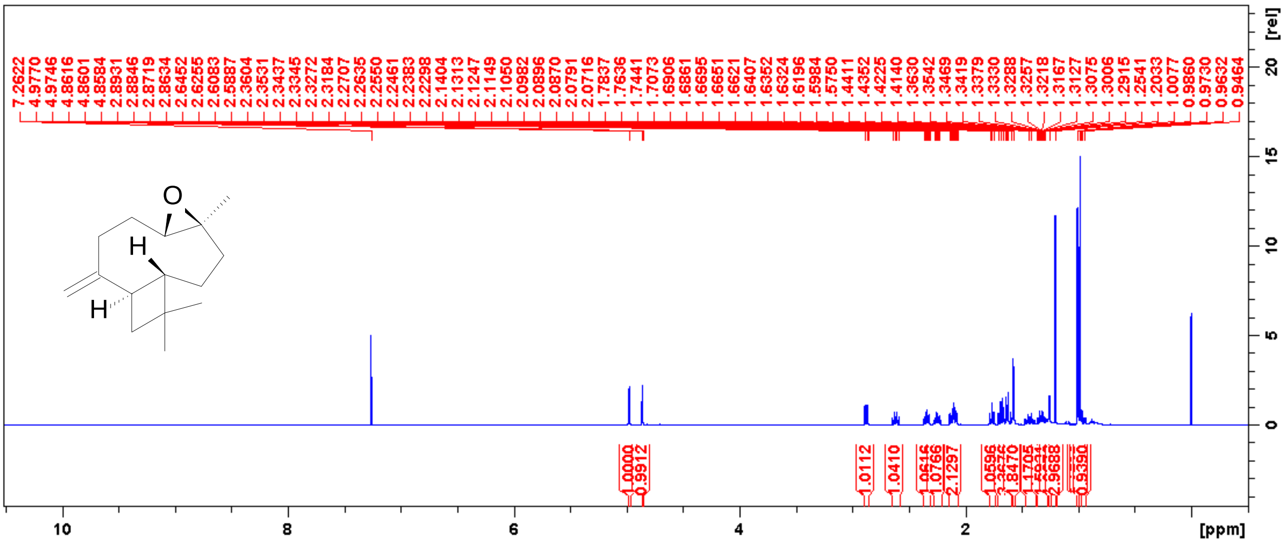

Figure S21. Full ^{1}H NMR spectrum of **caryophyllene oxide** (**2**) recorded in $CDCl_3$ at 500 MHz.

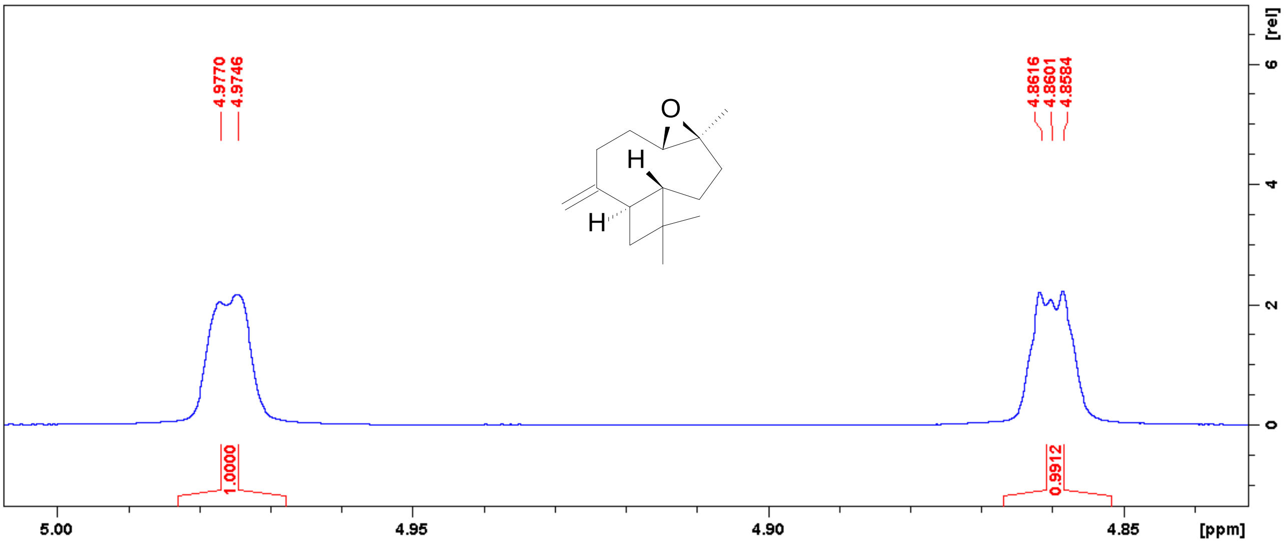

Figure S22. Expanded view of the olefinic proton region in the ^{1}H NMR spectrum of **caryophyllene oxide** (**2**) recorded in $CDCl_3$ at 500 MHz.

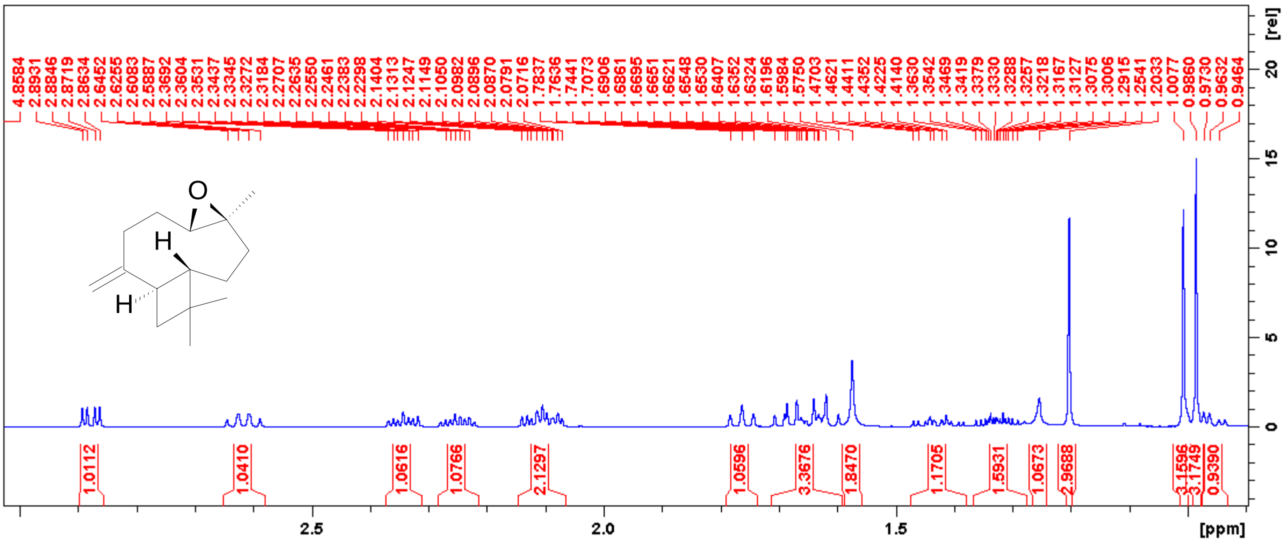

Figure S23. Expanded view of the aliphatic proton region in the ^{1}H NMR spectrum of **caryophyllene oxide** (**2**) recorded in $CDCl_3$ at 500 MHz.

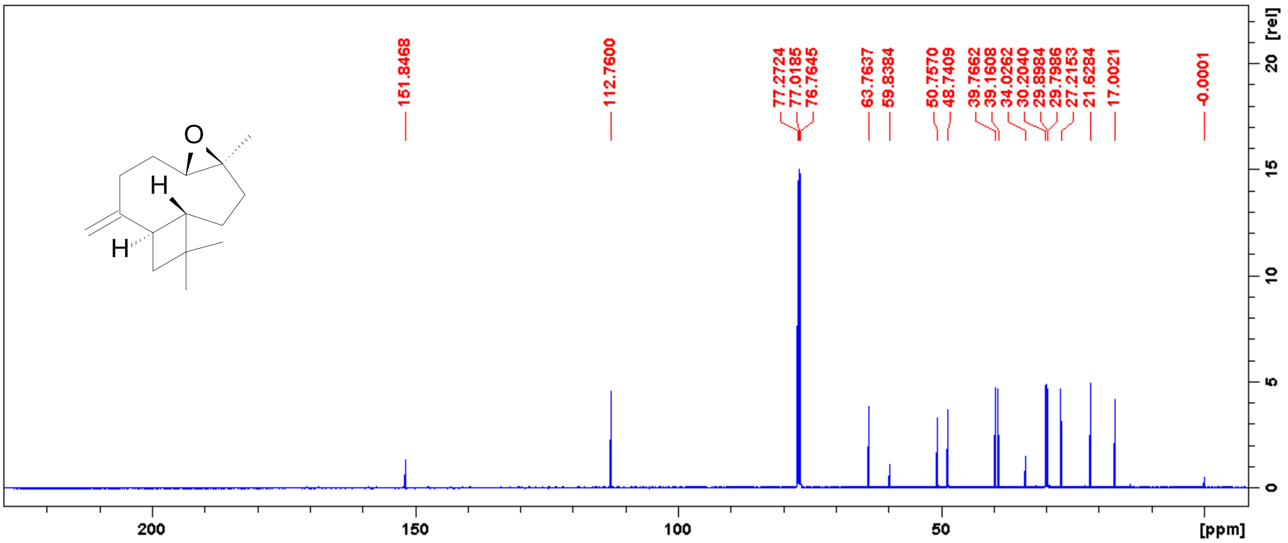

Figure S24. Full ^{13}C NMR spectrum of **caryophyllene oxide** (**2**) recorded in $CDCl_3$ at 125 MHz.

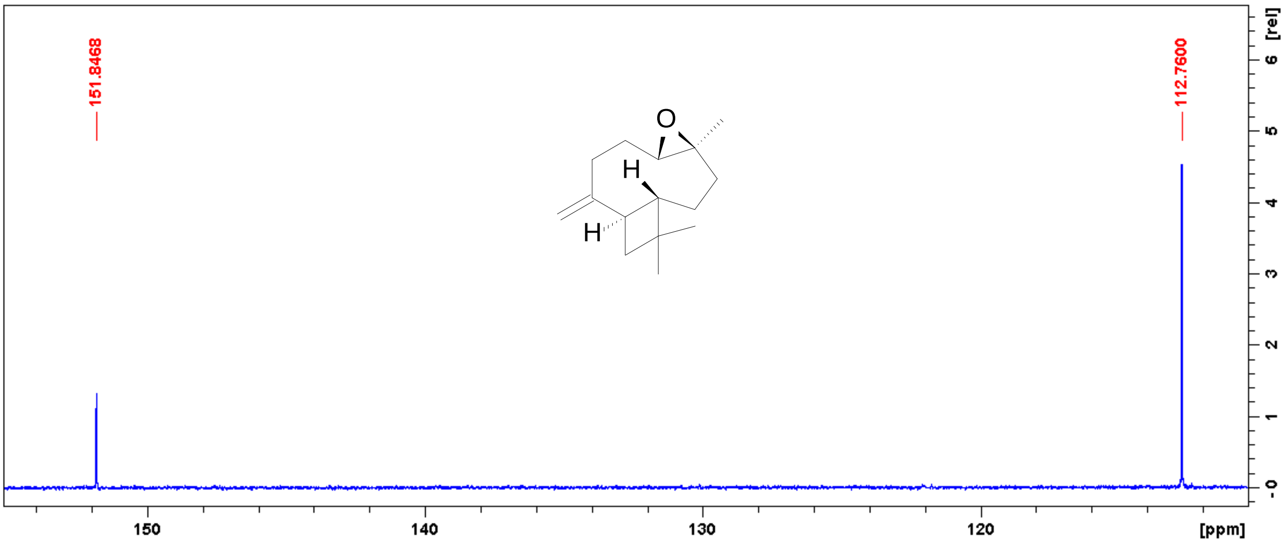

Figure S25. Expanded view of the carbon region (δ_C 155.0–110.5 ppm) in the ^{13}C NMR spectrum of **caryophyllene oxide** (**2**) recorded in $CDCl_3$ at 125 MHz.

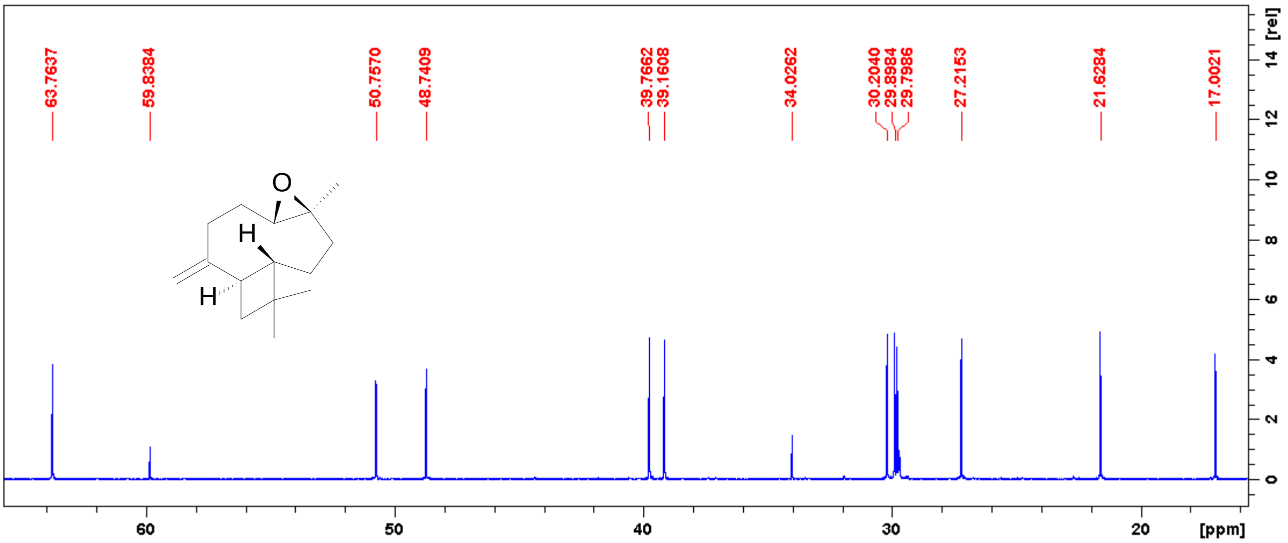

Figure S26. Expanded view of the carbon region (δ_C 65.6–15.8 ppm) in the ^{13}C NMR spectrum of **caryophyllene oxide** (**2**) recorded in $CDCl_3$ at 125 MHz.

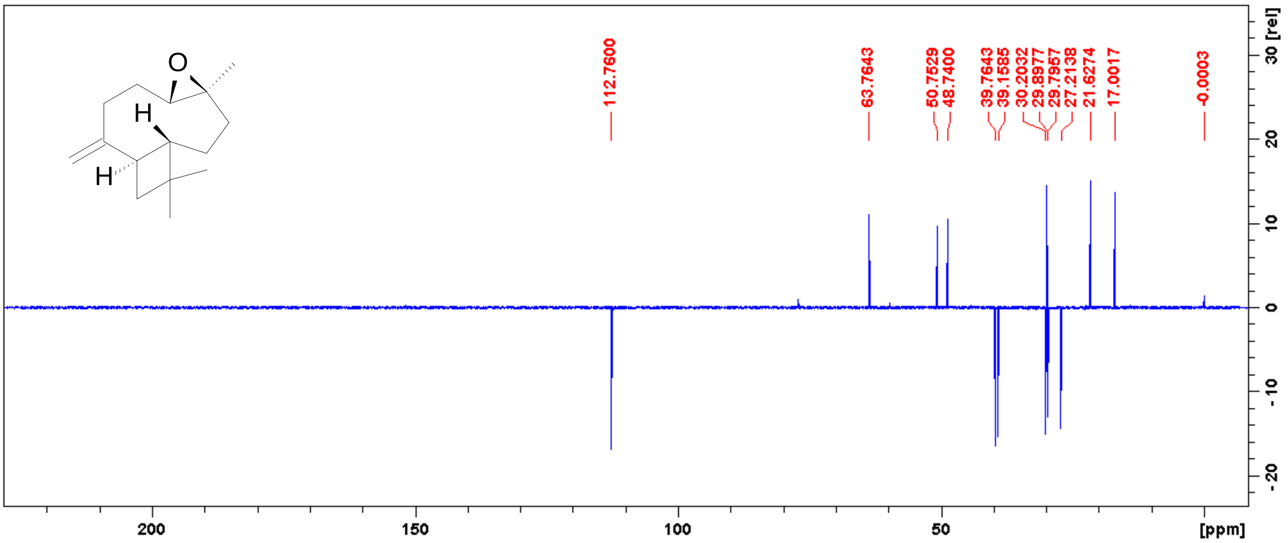

Figure S27. Full DEPT-135 ^{13}C NMR spectrum of **caryophyllene oxide** (**2**) recorded in $CDCl_3$ at 125 MHz.

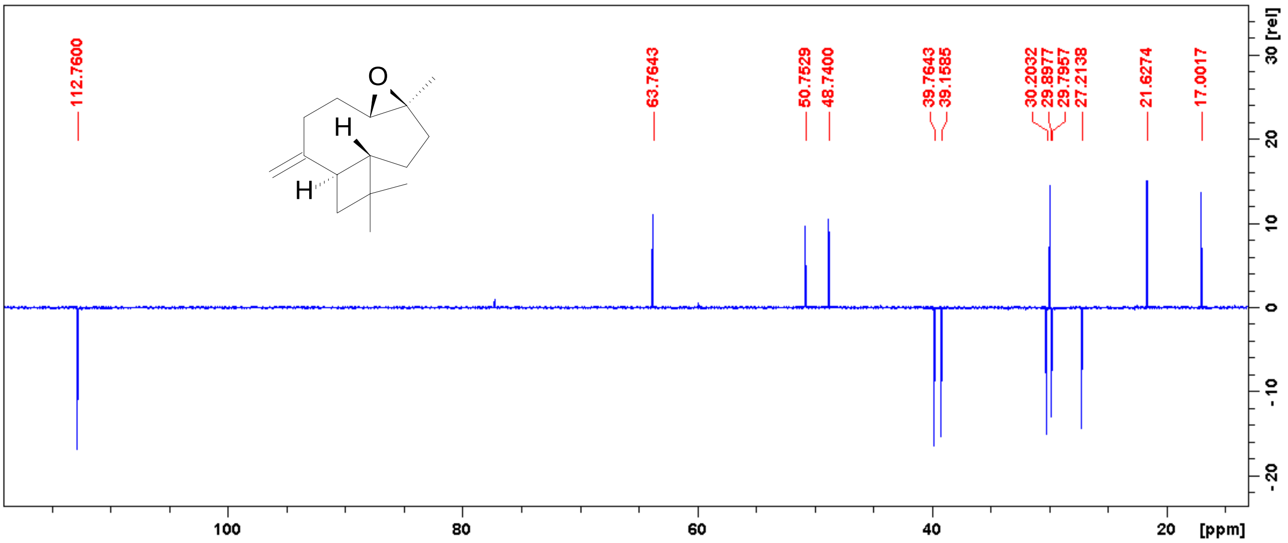

Figure S28. Expanded view of the carbon region (δ_C 118.6–13.4 ppm) in the DEPT-135 ^{13}C NMR spectrum of **caryophyllene oxide** (**2**) recorded in $CDCl_3$ at 125 MHz.

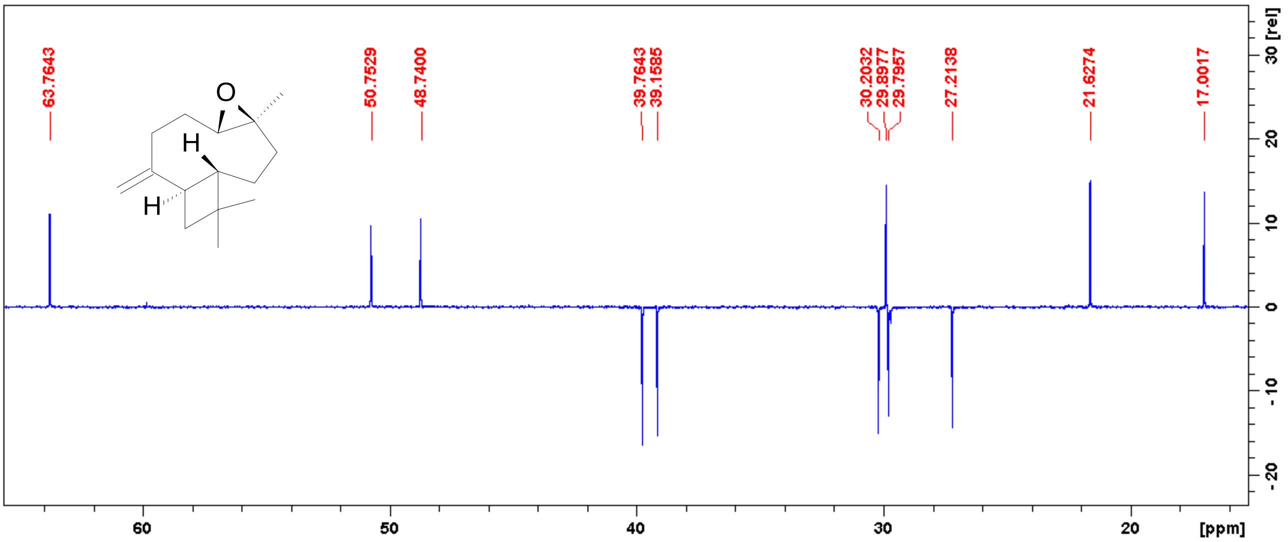

Figure S29. Expanded view of the carbon region (δ_C 65.5–15.3 ppm) in the DEPT-135 ^{13}C NMR spectrum of **caryophyllene oxide** (**2**) recorded in $CDCl_3$ at 125 MHz.

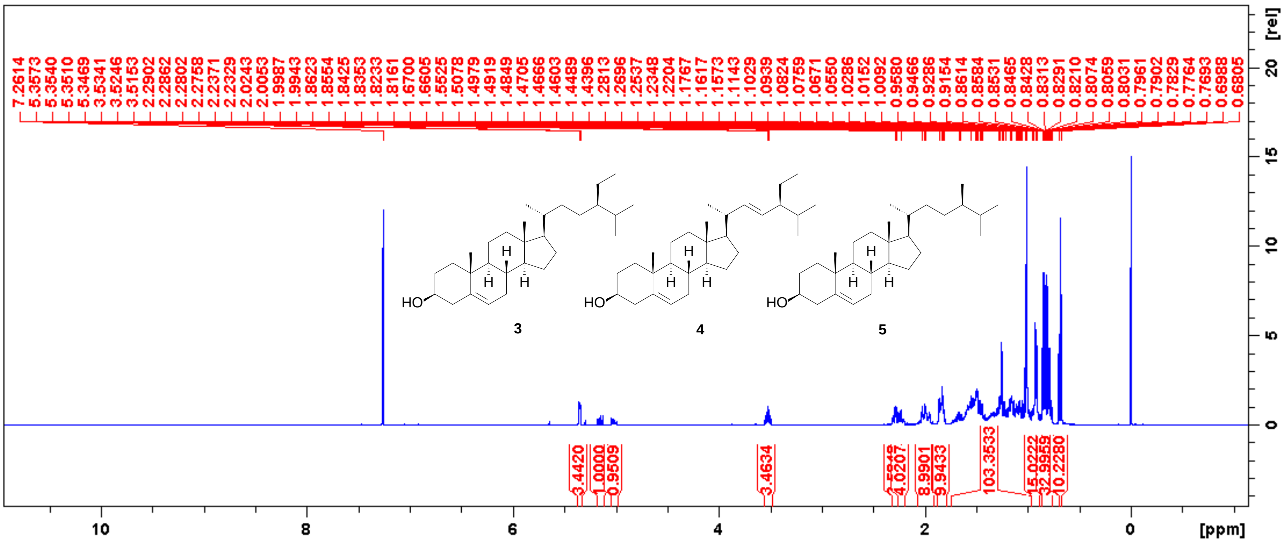

Figure S30. Full ^{1}H NMR spectrum of the mixture of **β-sitosterol** (**3**), **stigmasterol** (**4**), and **campesterol** (**5**), recorded in $CDCl_3$ at 500 MHz.

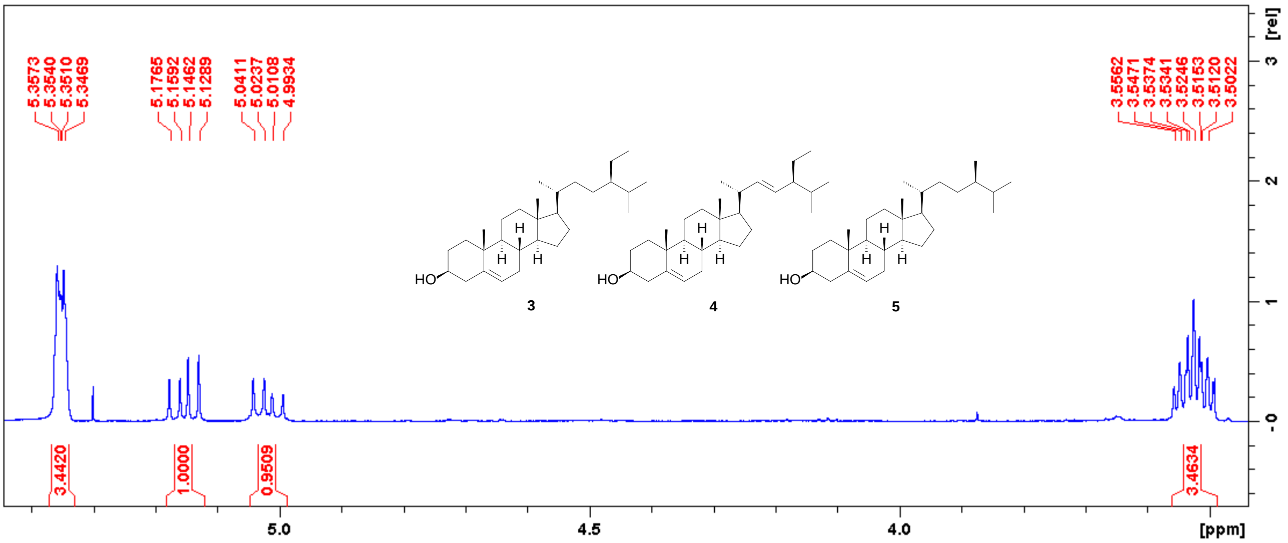

Figure S31. Expanded view of the olefinic and carbinolic proton region in the ^{1}H NMR spectrum of **β-sitosterol** (**3**), **stigmasterol** (**4**), and **campesterol** (**5**), recorded in $CDCl_3$ at 500 MHz.

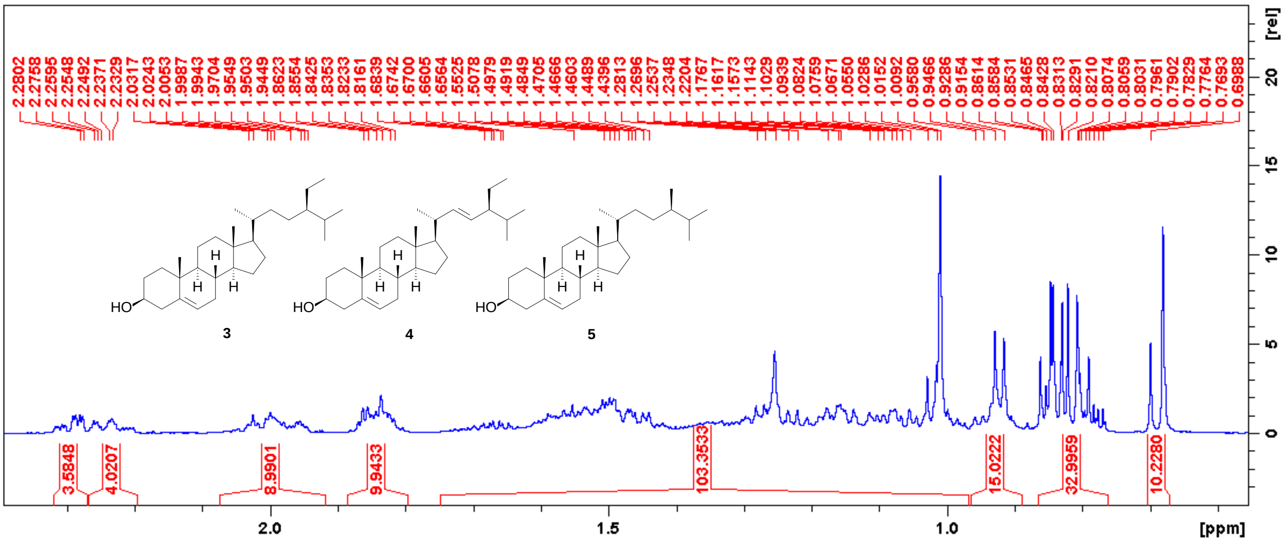

Figure S32. Expanded view of the aliphatic proton region in the ^{1}H NMR spectrum of **β-sitosterol** (**3**), **stigmasterol** (**4**), and **campesterol** (**5**), recorded in $CDCl_3$ at 500 MHz.

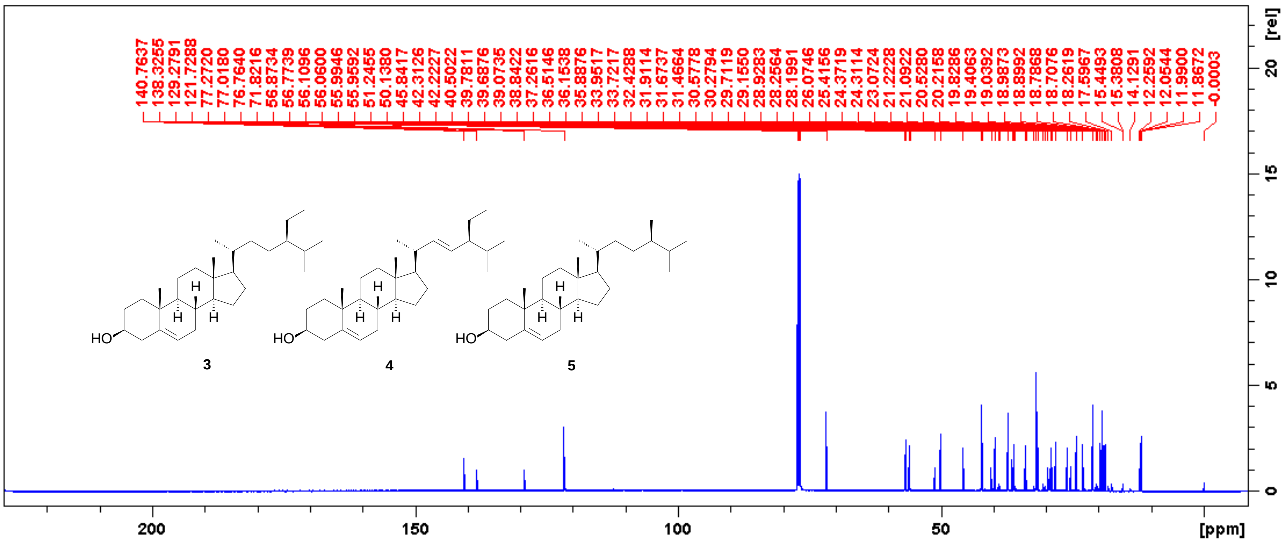

Figure S33. Full ^{13}C NMR spectrum of the mixture of **β-sitosterol** (**3**), **stigmasterol** (**4**), and **campesterol** (**5**), recorded in $CDCl_3$ at 125 MHz.

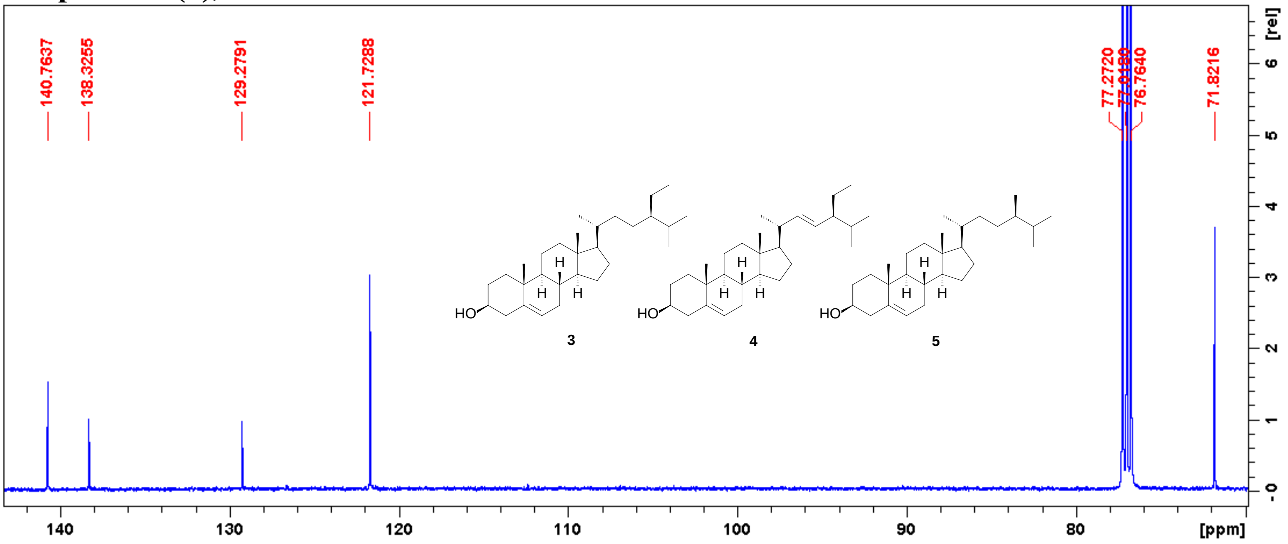

Figure S34. Expanded view of the carbon region (δ_C 143.0–70.0 ppm) in the ^{13}C NMR spectrum of the mixture of **β-sitosterol** (**3**), **stigmasterol** (**4**), and **campesterol** (**5**), recorded in $CDCl_3$ at 125 MHz.

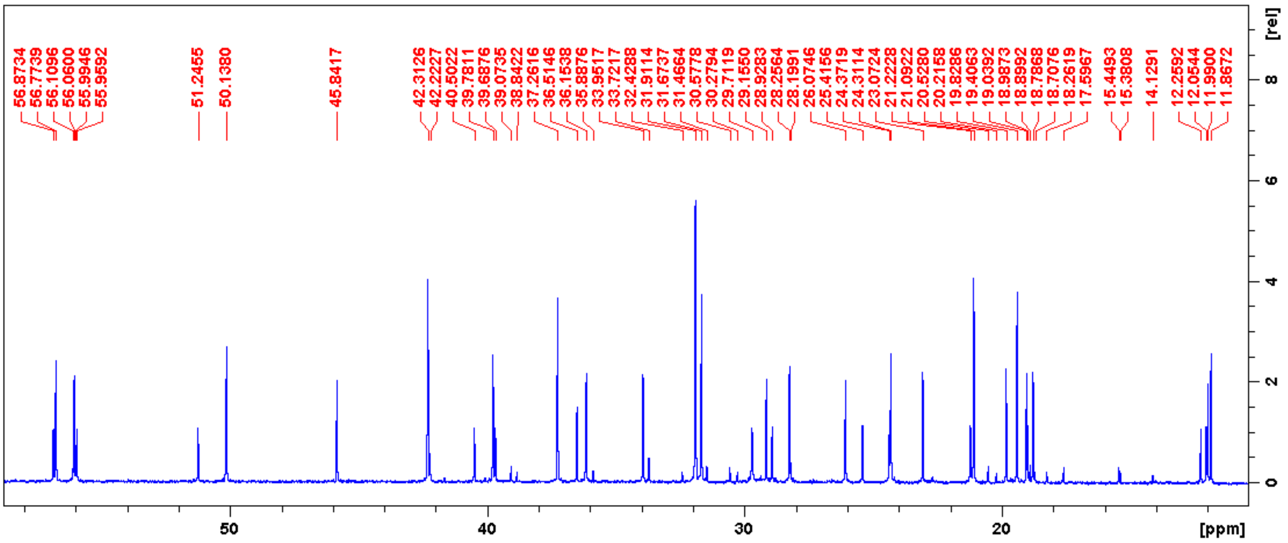

Figure S35. Expanded view of the carbon region (δ_C 58.6–10.6 ppm) in the ^{13}C NMR spectrum of the mixture of **β-sitosterol** (**3**), **stigmasterol** (**4**), and **campesterol** (**5**), recorded in $CDCl_3$ at 125 MHz.

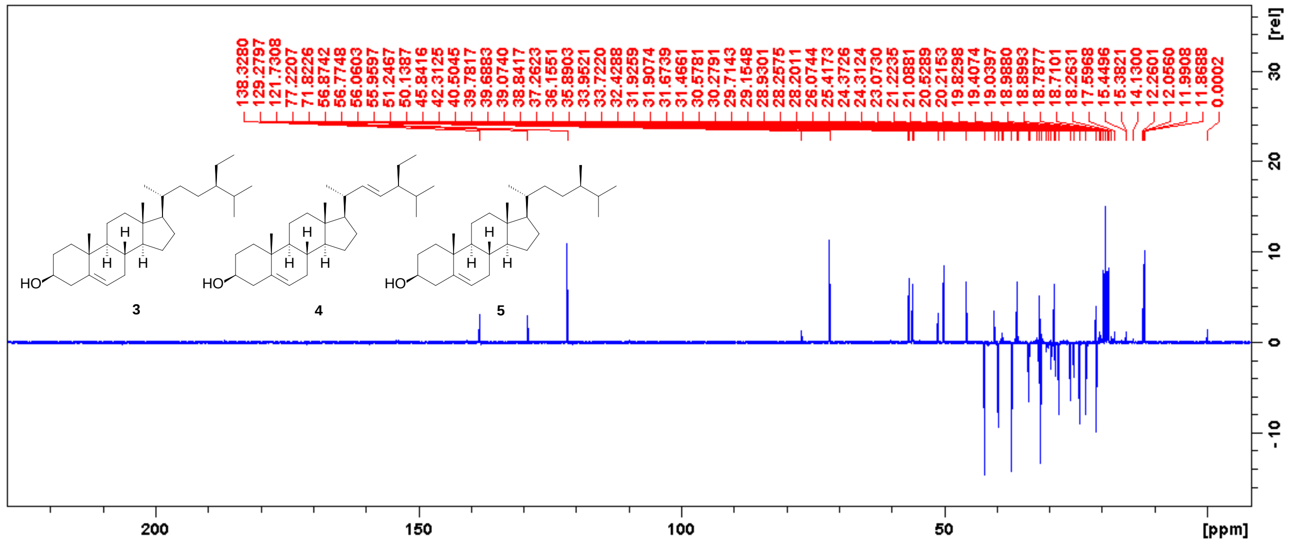

Figure S36. Full DEPT-135 ^{13}C NMR spectrum of the mixture of **β-sitosterol** (**3**), **stigmasterol** (**4**), and **campesterol** (**5**), recorded in $CDCl_3$ at 125 MHz.

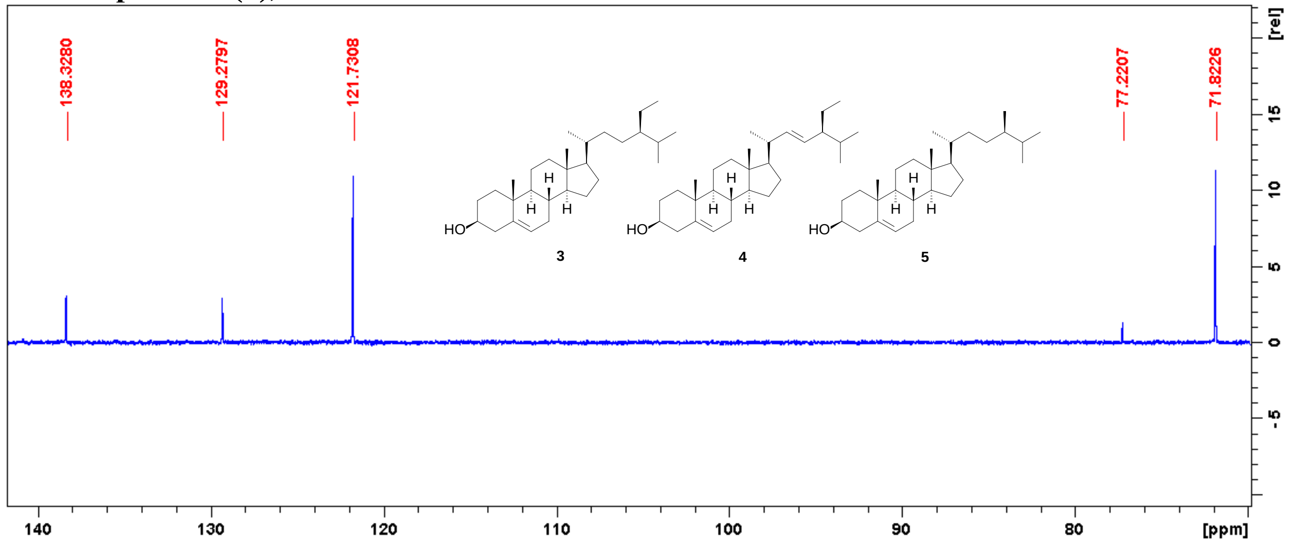

Figure S37. Expanded view of the carbon region (δ_C 141.5–70.0 ppm) in the DEPT-135 ^{13}C NMR spectrum of the mixture of **β-sitosterol** (**3**), **stigmasterol** (**4**), and **campesterol** (**5**), recorded in $CDCl_3$ at 125 MHz.

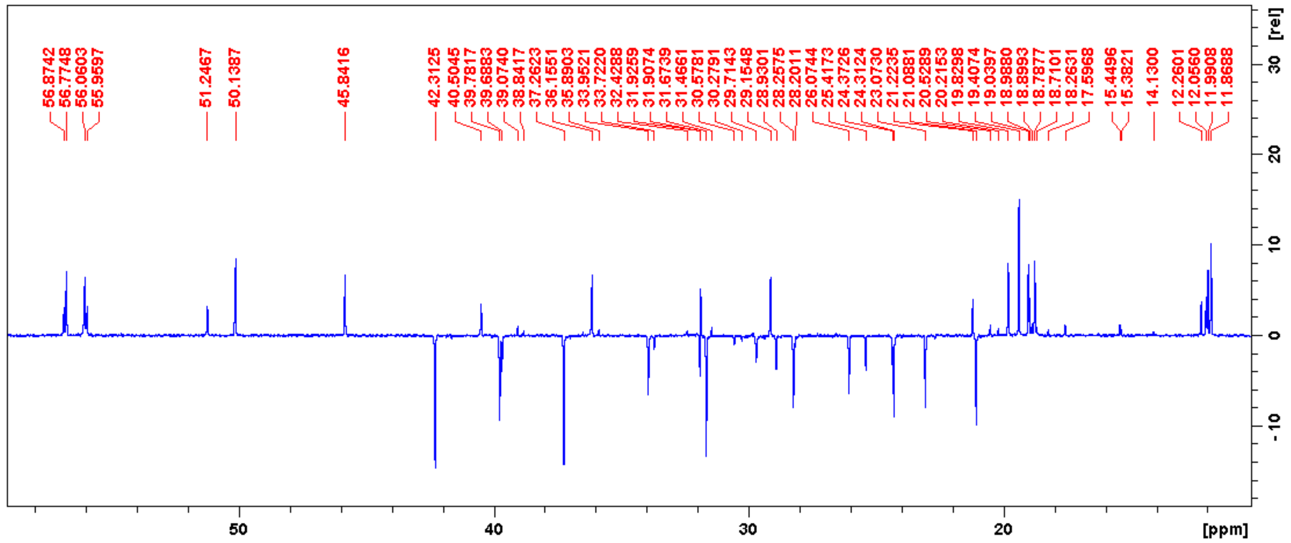

Figure S38. Expanded view of the carbon region (δ_C 59.0–10.4 ppm) in the DEPT-135 ^{13}C NMR spectrum of the mixture of **β-sitosterol** (**3**), **stigmasterol** (**4**), and **campesterol** (**5**), recorded in $CDCl_3$ at 125 MHz.

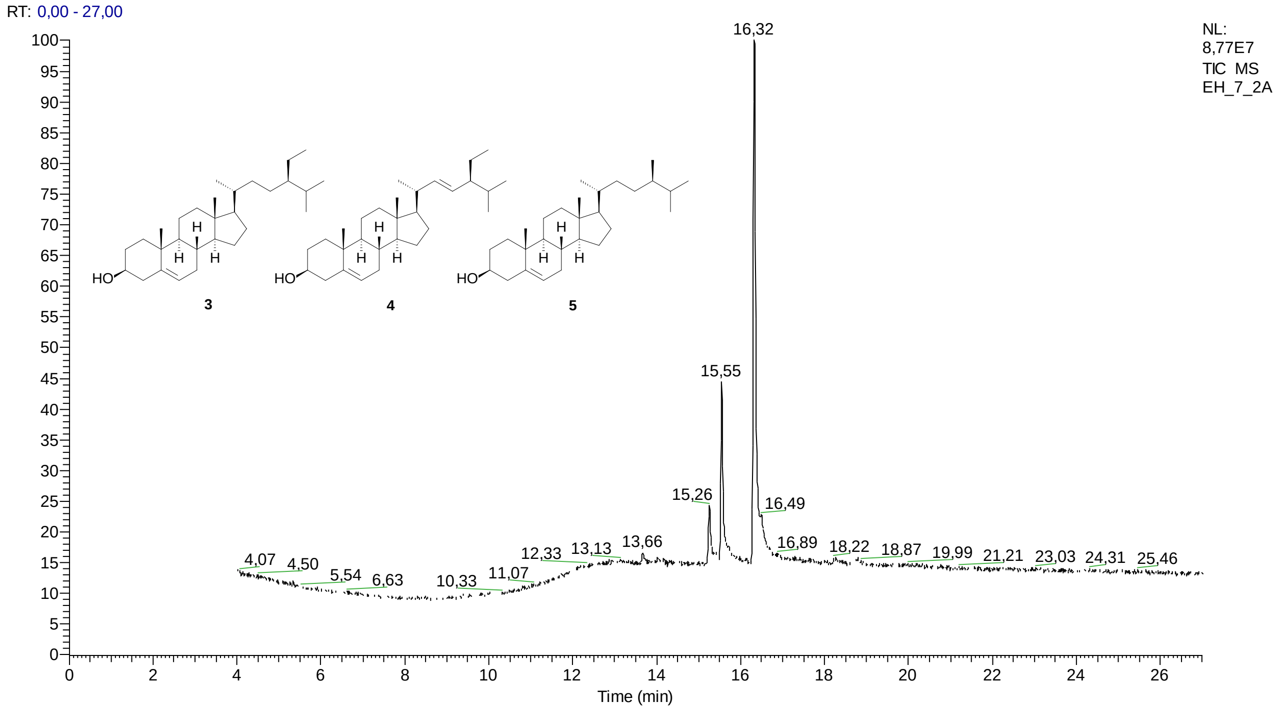

Figure S39. Total ion chromatogram (GC–MS) of the mixture of **β-sitosterol** (**3**), stigmasterol (**4**), and **campesterol** (**5**), with retention times of 16.32, 15.55, and 15.26 min, respectively.

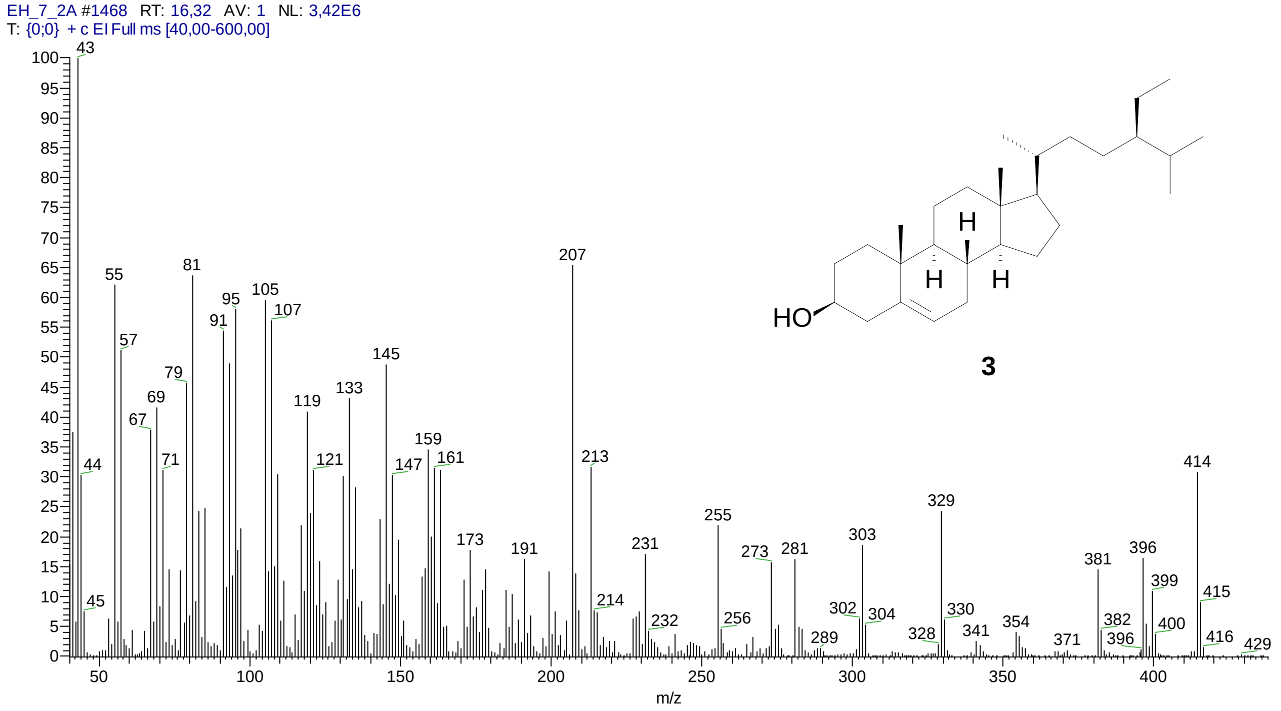

Figure S40. Mass spectrum of **β-sitosterol** (**3**), corresponding to the chromatographic peak at a retention time of 16.32 min, showing the molecular ion at *m/z* 414 ($M^{+\bullet}$).

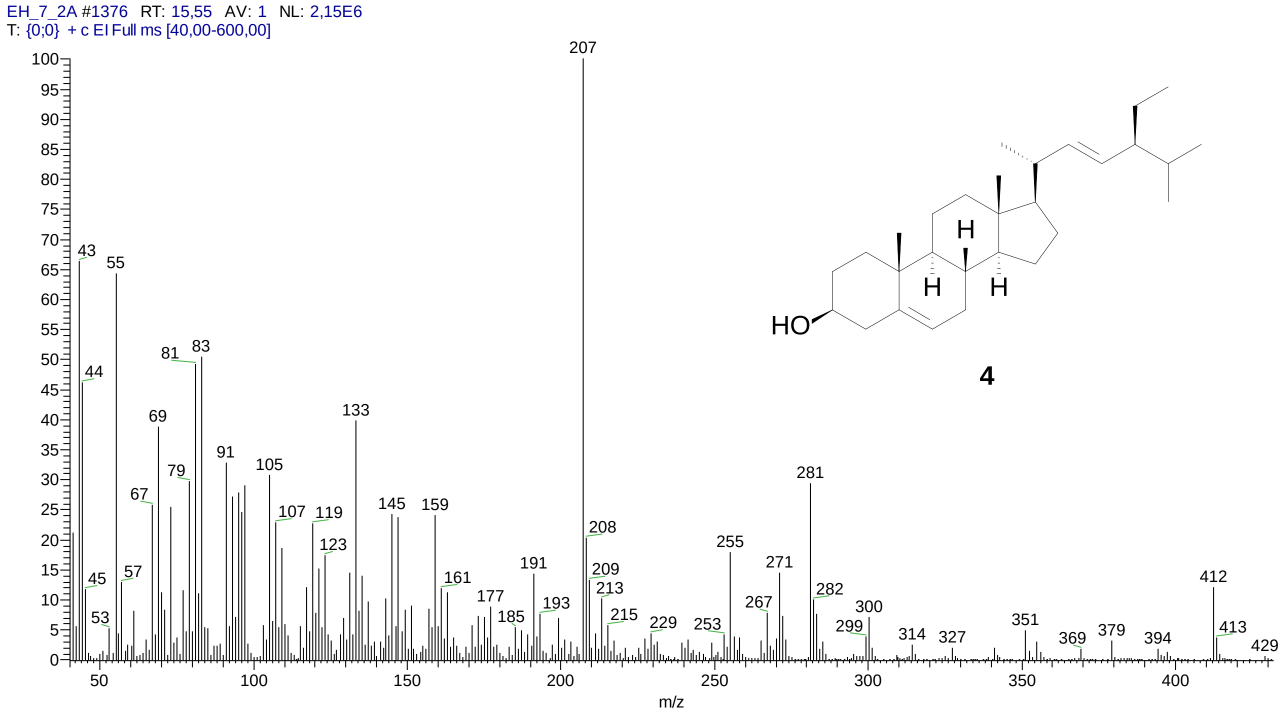

Figure S41. Mass spectrum of **stigmasterol** (**4**), corresponding to the chromatographic peak at a retention time of 15.55 min, showing the molecular ion at *m/z* 412 ($M^{+\bullet}$).

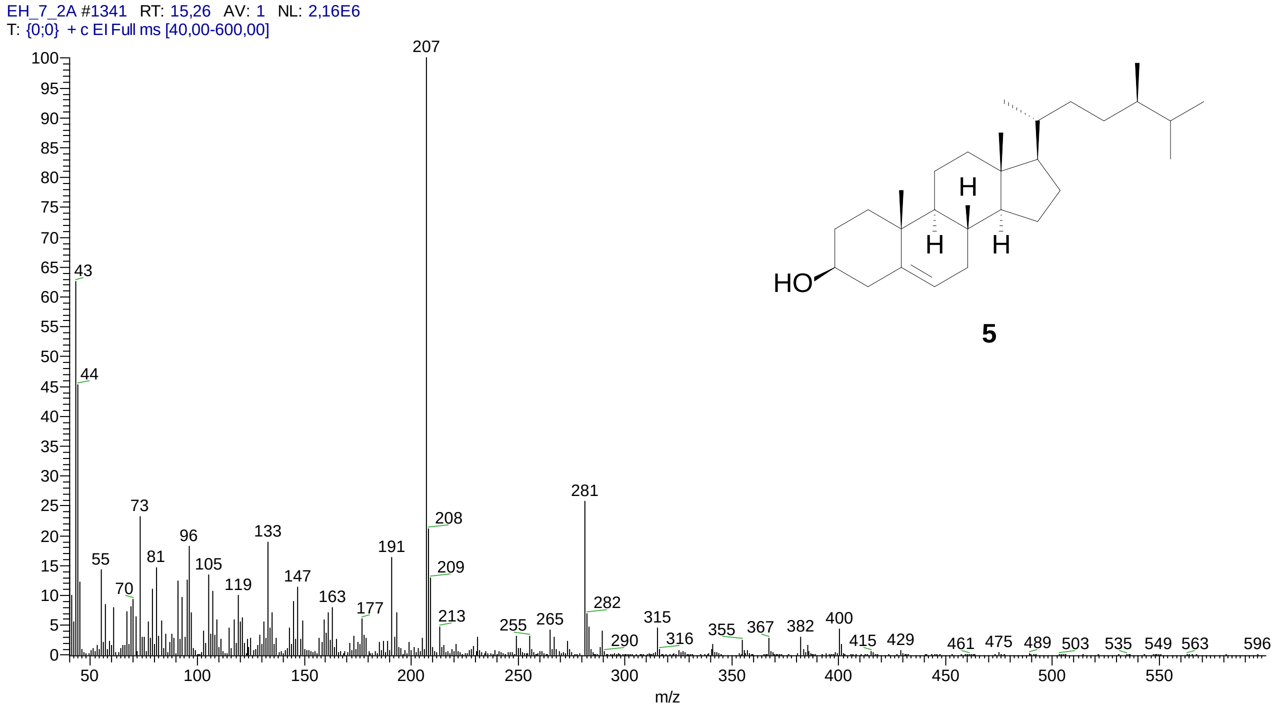

Figure S42. Mass spectrum of **campesterol** (**5**), corresponding to the chromatographic peak at a retention time of 15.26 min, showing the molecular ion at *m/z* 400 ($M^{+\bullet}$).

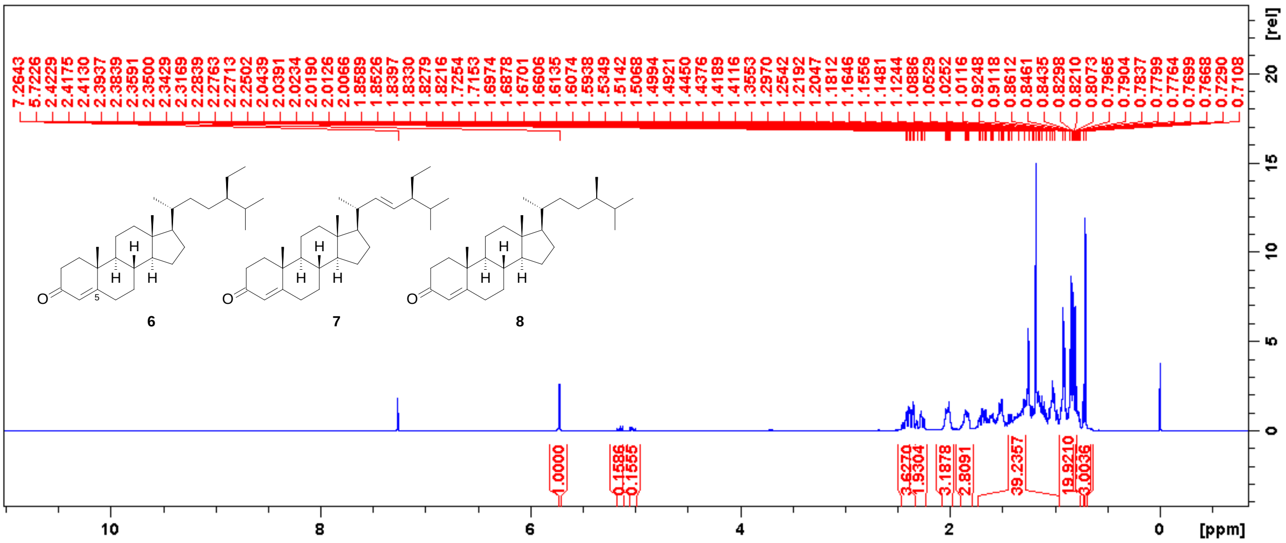

Figure S43. Full ^{1}H NMR spectrum of the mixture of **β-sitostenone** (**6**), **stigmasta-4,22-dien-3-one** (**7**), and **campest-4-en-3-one** (**8**), recorded in $CDCl_3$ at 500 MHz.

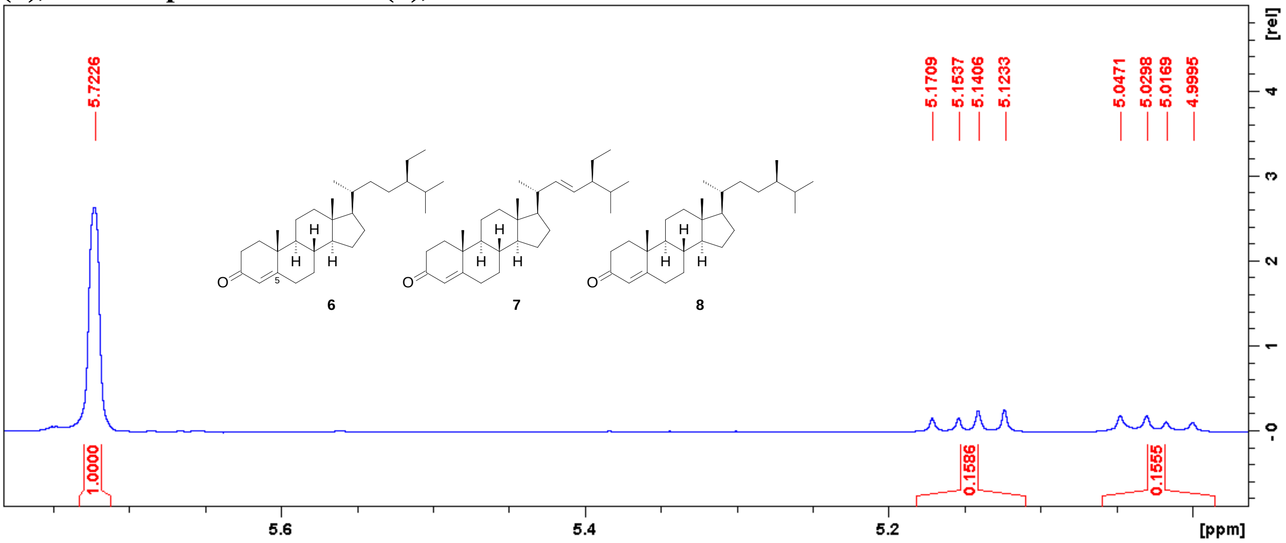

Figure S44. Expanded view of the olefinic and carbinolic proton region in the ^{1}H NMR spectrum of mixture of **β-sitostenone** (**6**), **stigmasta-4,22-dien-3-one** (**7**), and **campest-4-en-3-one** (**8**), recorded in $CDCl_3$ at 500 MHz.

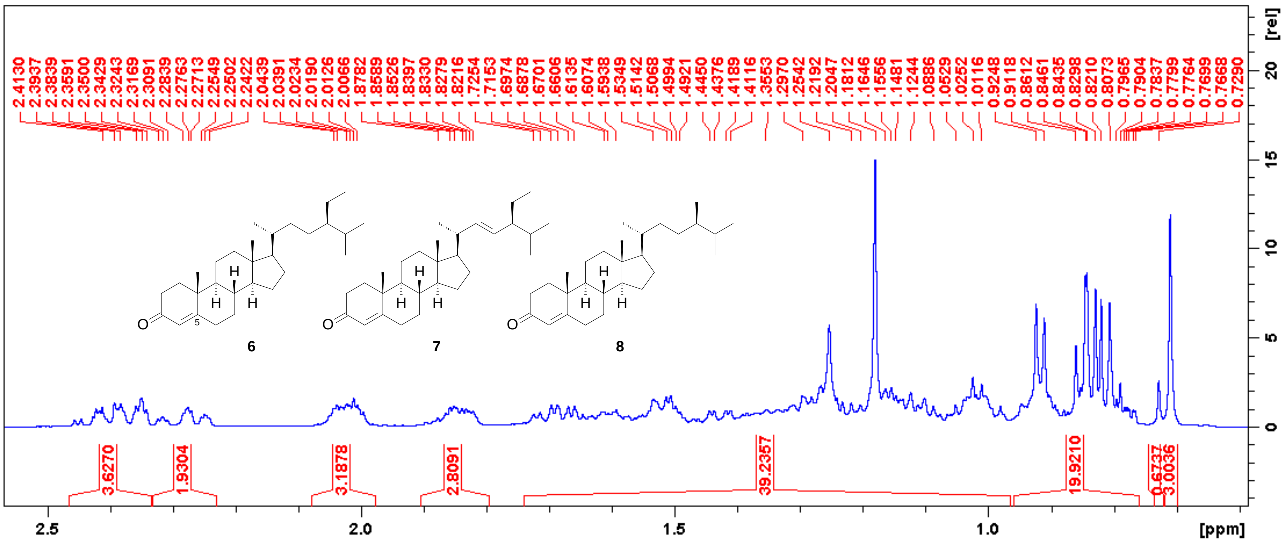

Figure S45. Expanded view of the aliphatic proton region in the ^{1}H NMR spectrum of mixture of **β-sitostenone** (**6**), **stigmasta-4,22-dien-3-one** (**7**), and **campest-4-en-3-one** (**8**), recorded in $CDCl_3$ at 500 MHz.

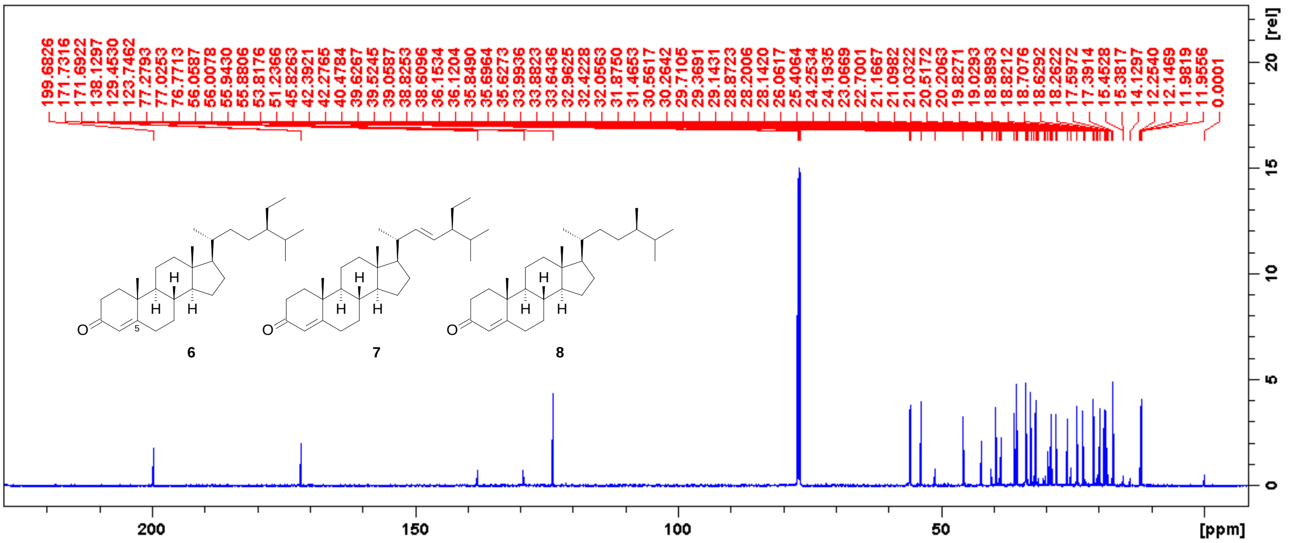

Figure S46. Full ^{13}C NMR spectrum of the mixture of **β-sitostenone** (**6**), **stigmasta-4,22-dien-3-one** (**7**), and **campest-4-en-3-one** (**8**), recorded in $CDCl_3$ at 125 MHz.

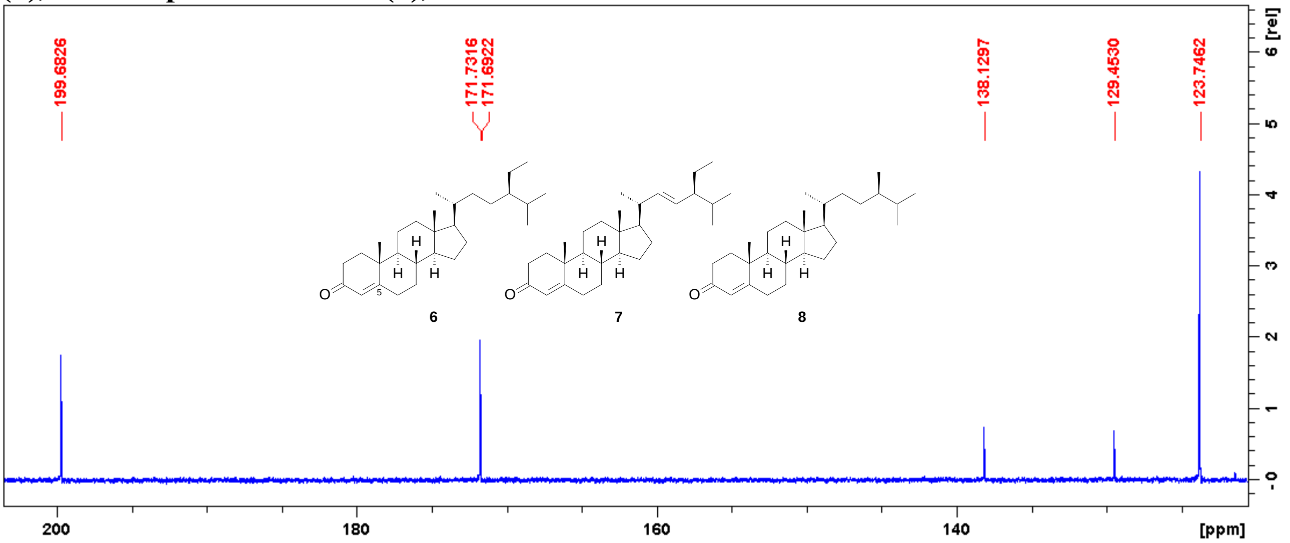

Figure S47. Expanded view of the carbon region (δ_C 203.2–120.7 ppm) in the ^{13}C NMR spectrum of the mixture of **β-sitostenone** (**6**), **stigmasta-4,22-dien-3-one** (**7**), and **campest-4-en-3-one** (**8**), recorded in $CDCl_3$ at 125 MHz.

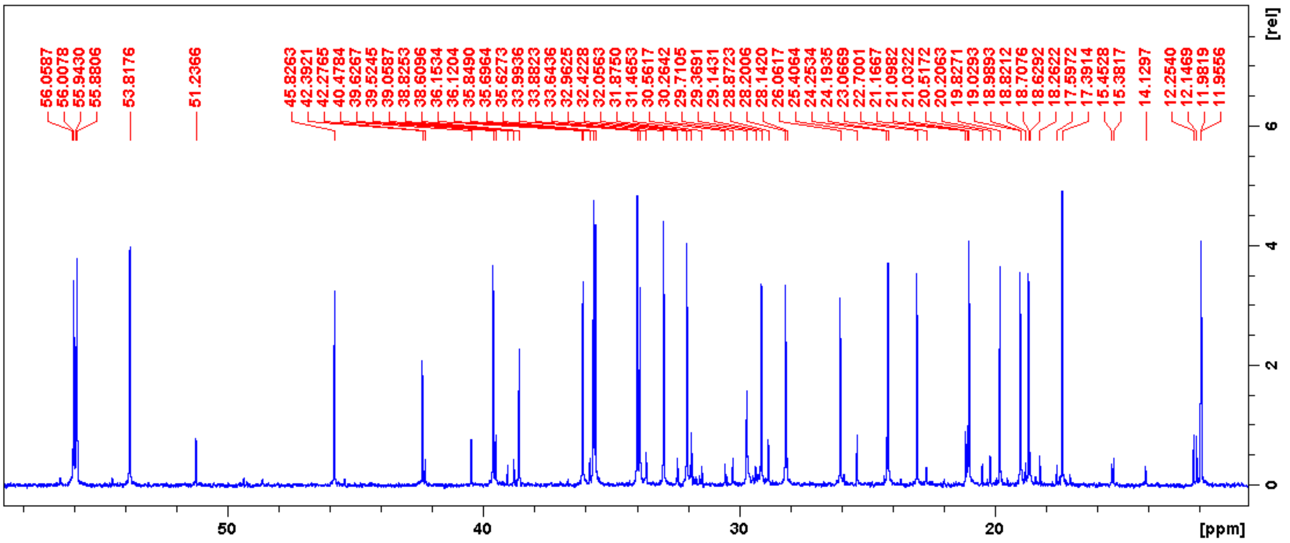

Figure S48. Expanded view of the carbon region (δ_C 58.7–10.2 ppm) in the ^{13}C NMR spectrum of the mixture of **β-sitostenone** (**6**), **stigmasta-4,22-dien-3-one** (**7**), and **campest-4-en-3-one** (**8**), recorded in $CDCl_3$ at 125 MHz.

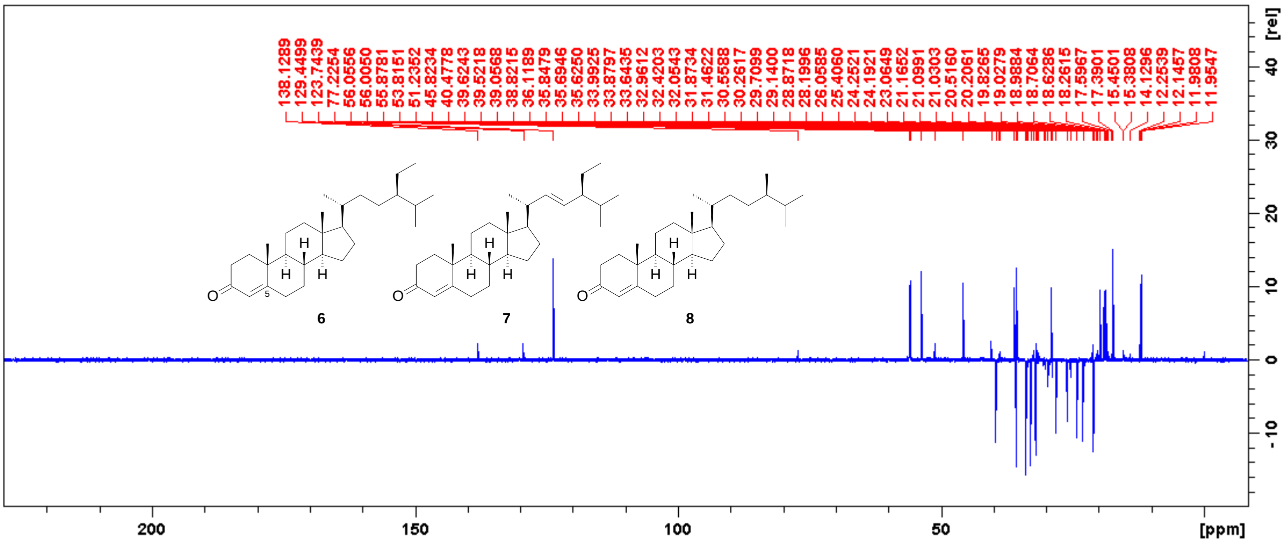

Figure S49. Full DEPT-135 ^{13}C NMR spectrum of the mixture of **β-sitostenone** (**6**), **stigmasta-4,22-dien-3-one** (**7**), and **campest-4-en-3-one** (**8**), recorded in $CDCl_3$ at 125 MHz.

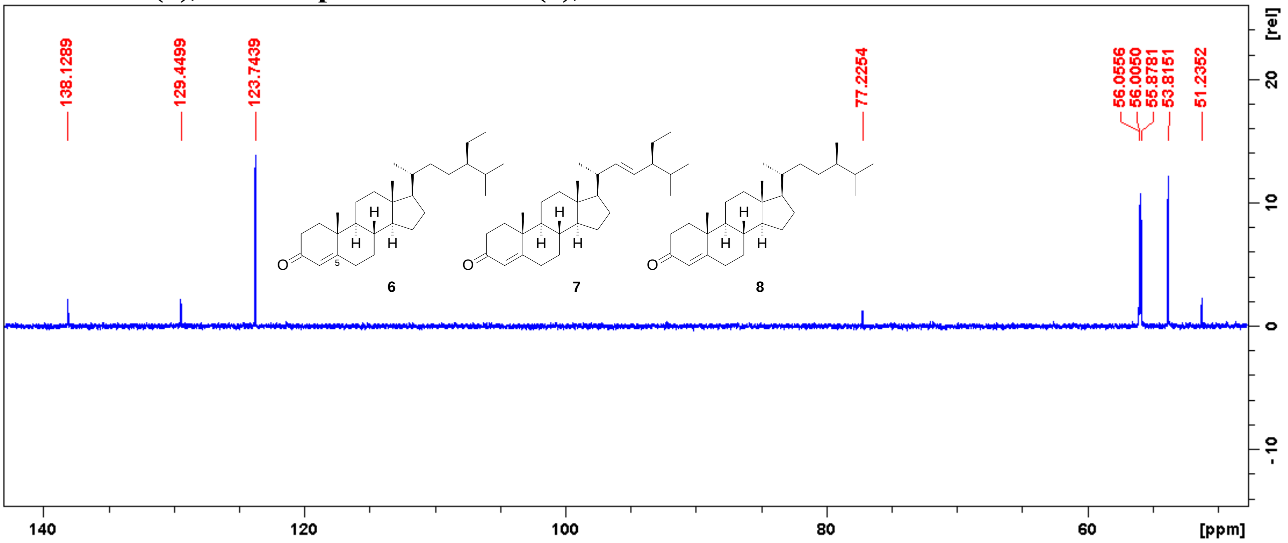

Figure S50. Expanded view of the carbon region (δ_C 142.7–48.0 ppm) in the DEPT-135 ^{13}C NMR spectrum of the mixture of **β-sitostenone** (**6**), **stigmasta-4,22-dien-3-one** (**7**), and **campest-4-en-3-one** (**8**), recorded in $CDCl_3$ at 125 MHz.

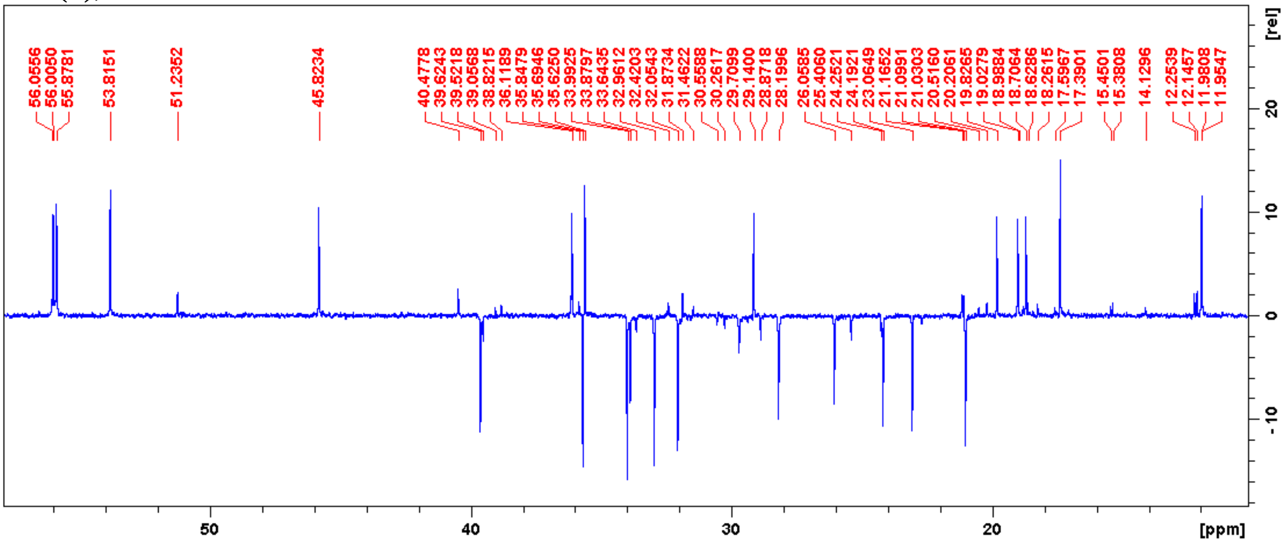

Figure S51. Expanded view of the carbon region (δ_C 57.8–10.3 ppm) in the DEPT-135 ^{13}C NMR spectrum of the mixture of **β-sitostenone** (**6**), **stigmasta-4,22-dien-3-one** (**7**), and **campest-4-en-3-one** (**8**), recorded in $CDCl_3$ at 125 MHz.

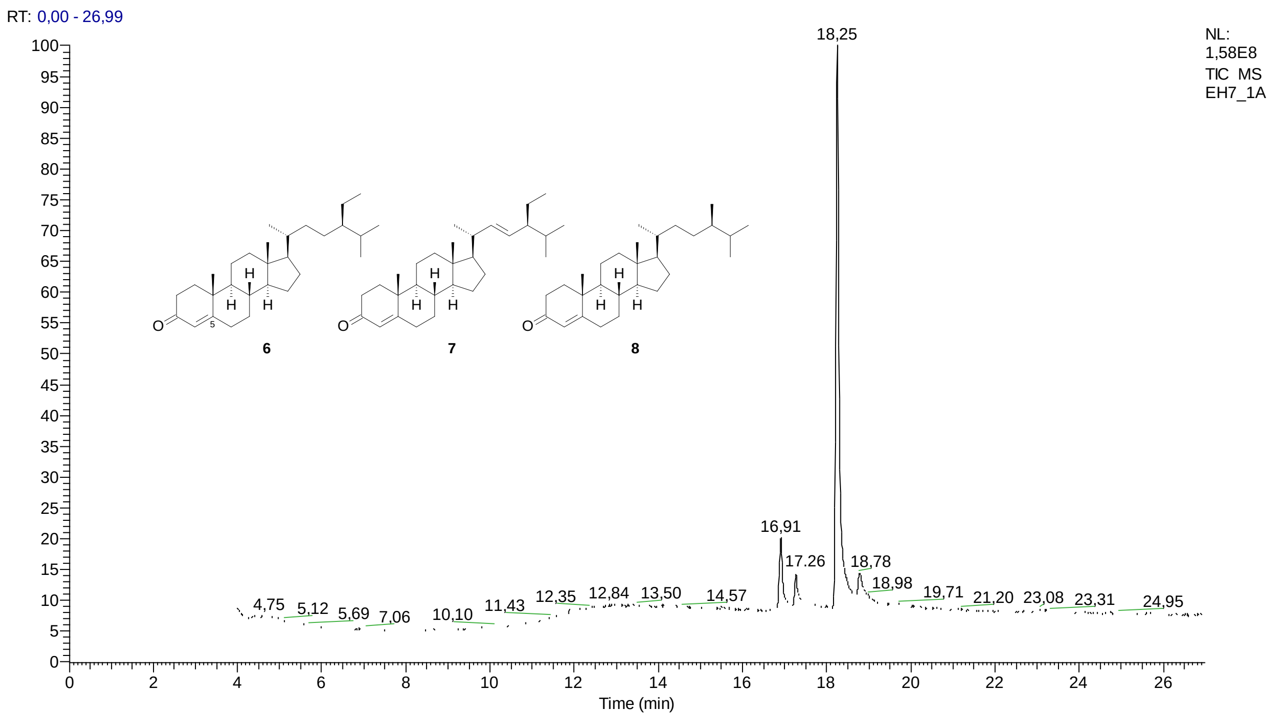

Figure S52. Total ion chromatogram (GC–MS) of the mixture of **β-sitostenone** (**6**), **stigmasta-4,22-dien-3-one** (**7**), and **campest-4-en-3-one** (**8**), with retention times of 18.25, 17.26, and 16.91 min, respectively.

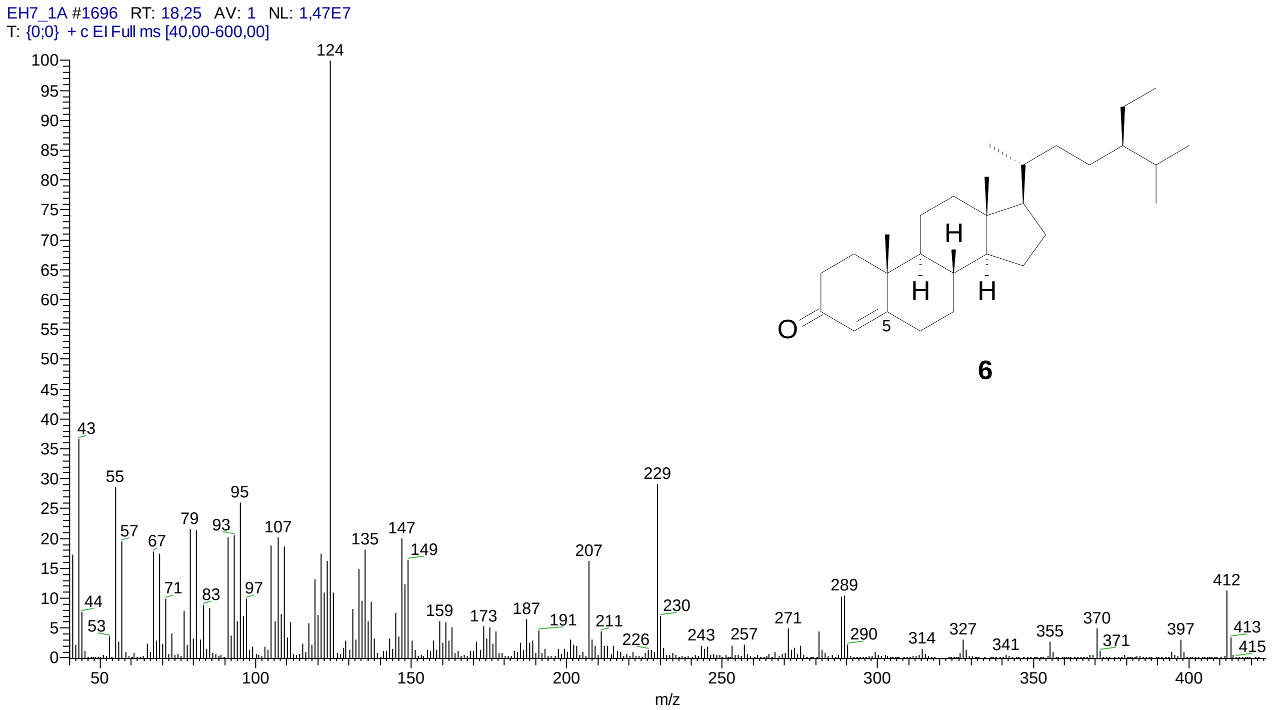

Figure S53. Mass spectrum of **β-sitostenone** (**6**), corresponding to the chromatographic peak at a retention time of 18.25 min showing the molecular ion at *m/z* 412 ($M^{+\bullet}$).

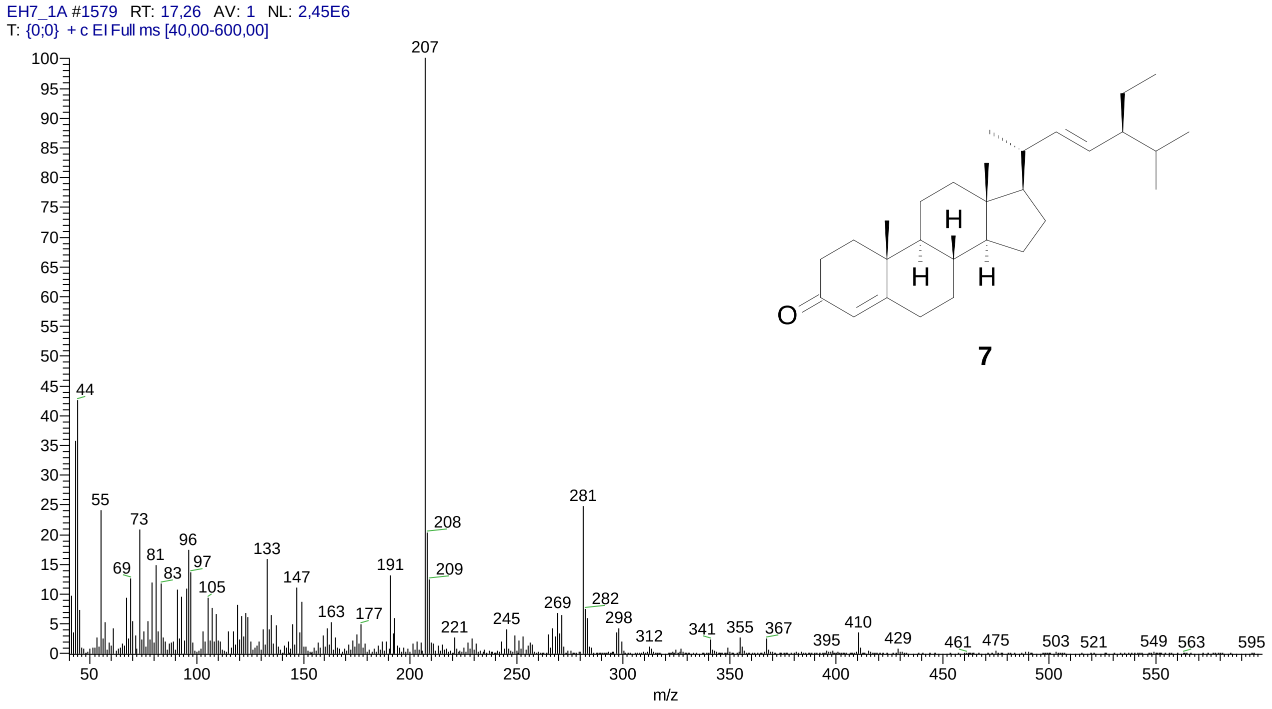

Figure S54. Mass spectrum of **stigmasta-4,22-dien-3-one** (**7**), corresponding to the chromatographic peak at a retention time of 17.26 min showing the molecular ion at *m/z* 410 ($M^{+\bullet}$).

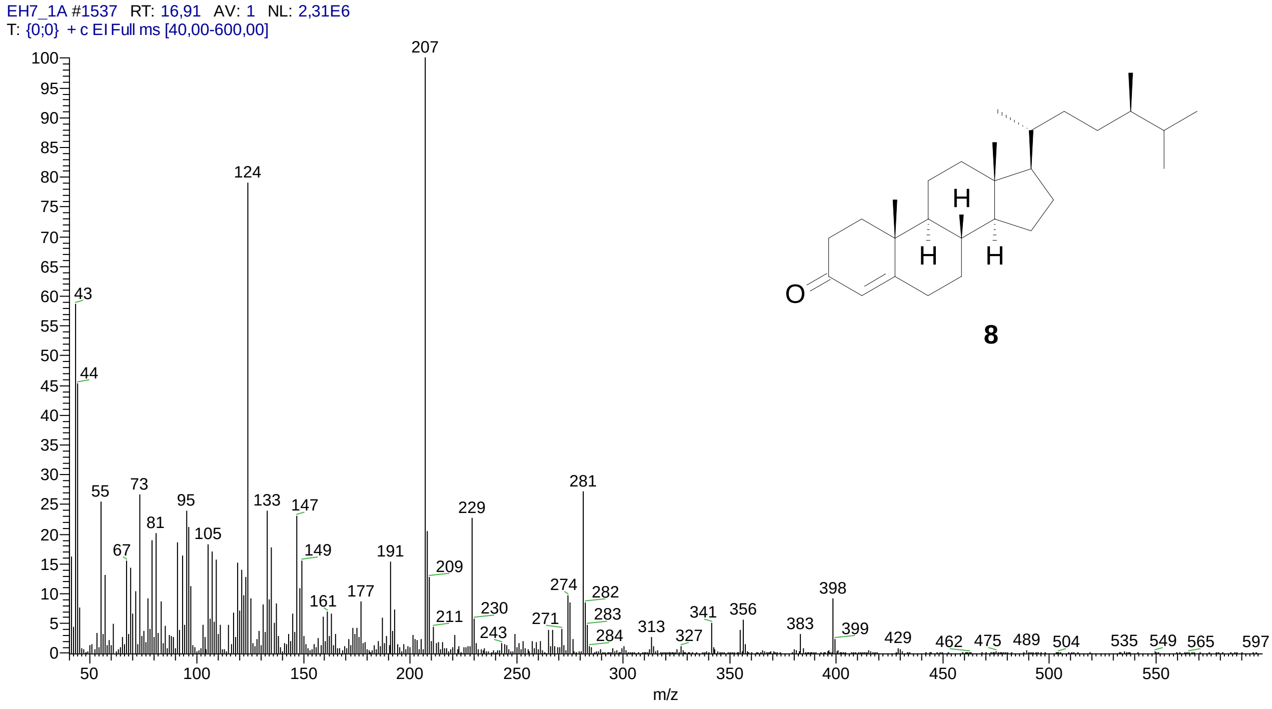

Figure S55. Mass spectrum of **campest-4-en-3-one** (**8**), corresponding to the chromatographic peak at a retention time of 16.91 min showing the molecular ion at *m/z* 398 ($M^{+\bullet}$).

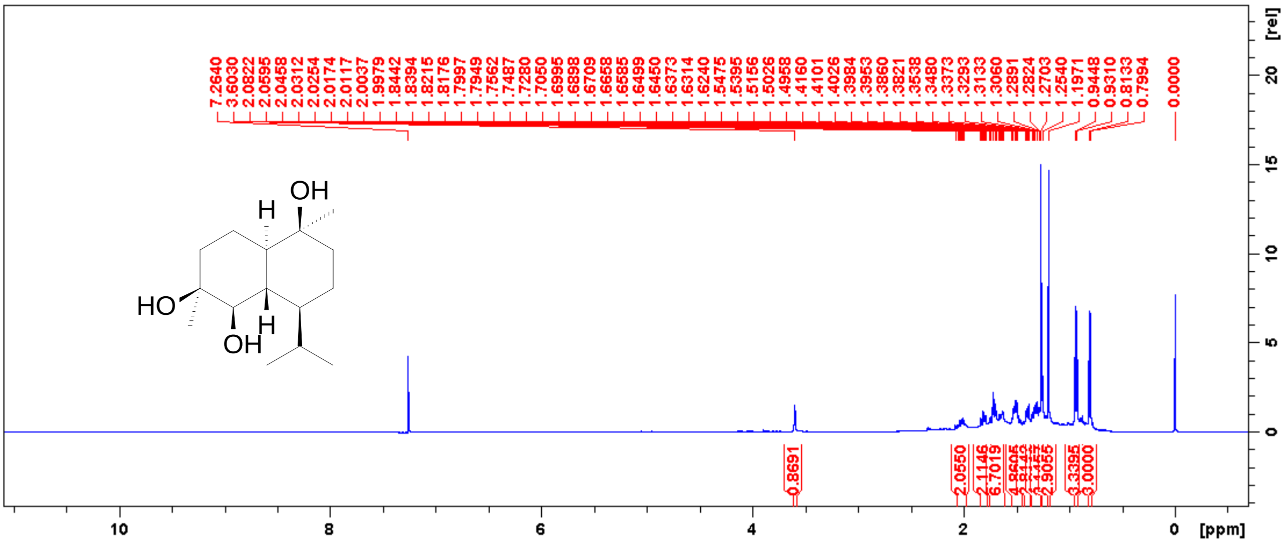

Figure S56. Full ^{1}H NMR spectrum of **cadinane-4β,5α,10β-triol** (**9**) recorded in $CDCl_3$ at 500 MHz.

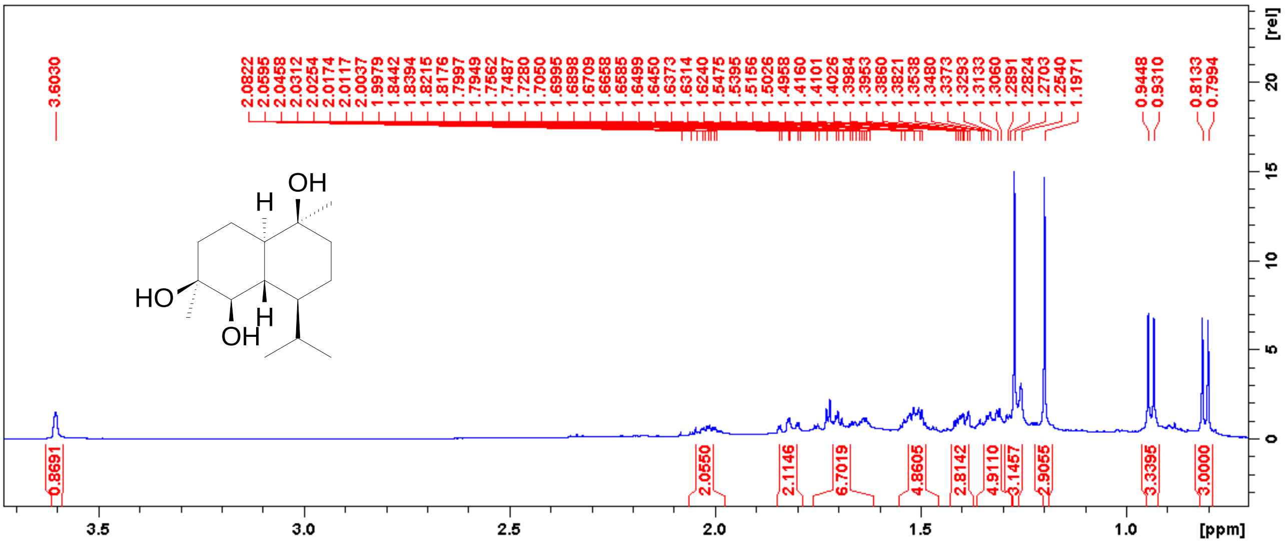

Figure S57. Expanded view 1 of the aliphatic proton region in the ^{1}H NMR spectrum of **cadinane-4β,5α,10β-triol** (**9**) recorded in $CDCl_3$ at 500 MHz.

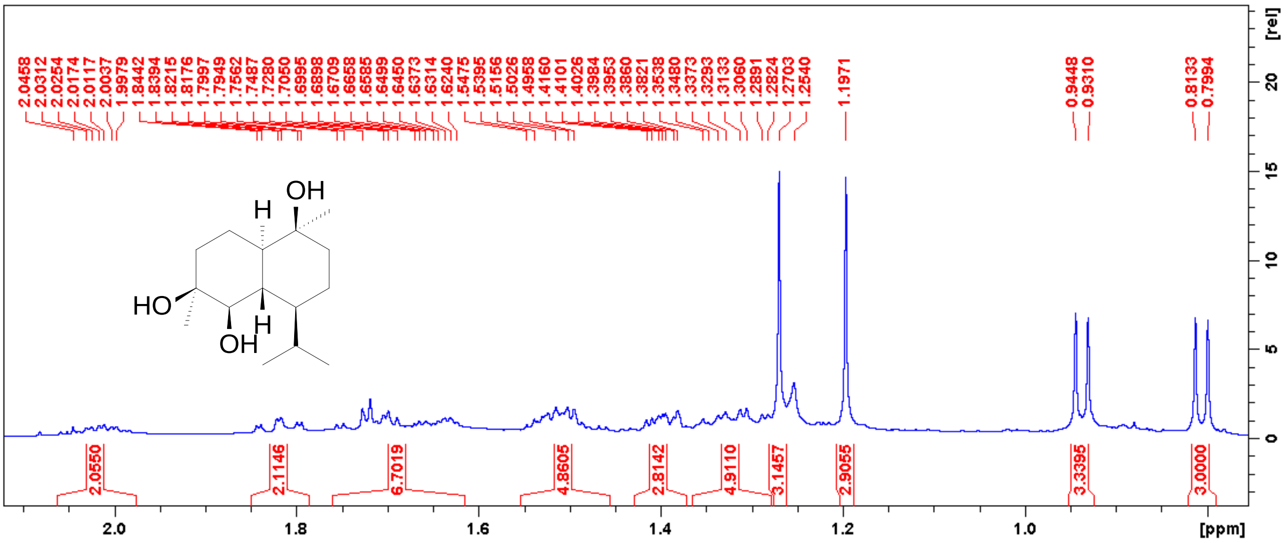

Figure S58. Expanded view 2 of the aliphatic proton region in the ^{1}H NMR spectrum of **cadinane-4β,5α,10β-triol** (**9**) recorded in $CDCl_3$ at 500 MHz.

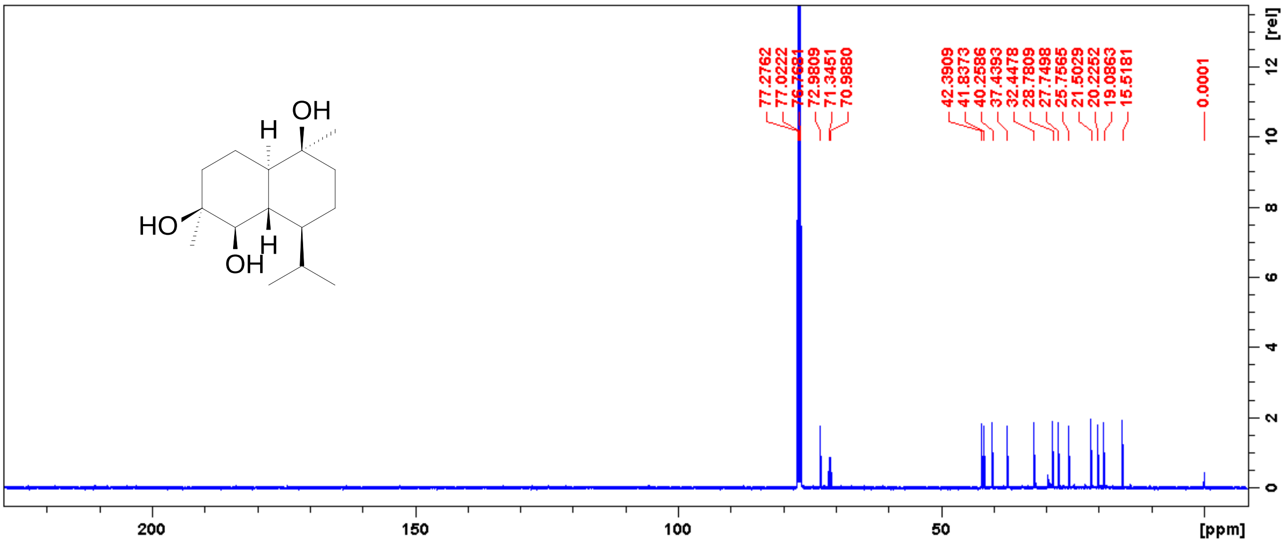

Figure S59. Full ^{13}C NMR spectrum of **cadinane-4β,5α,10β-triol** (**9**) recorded in $CDCl_3$ at 125 MHz.

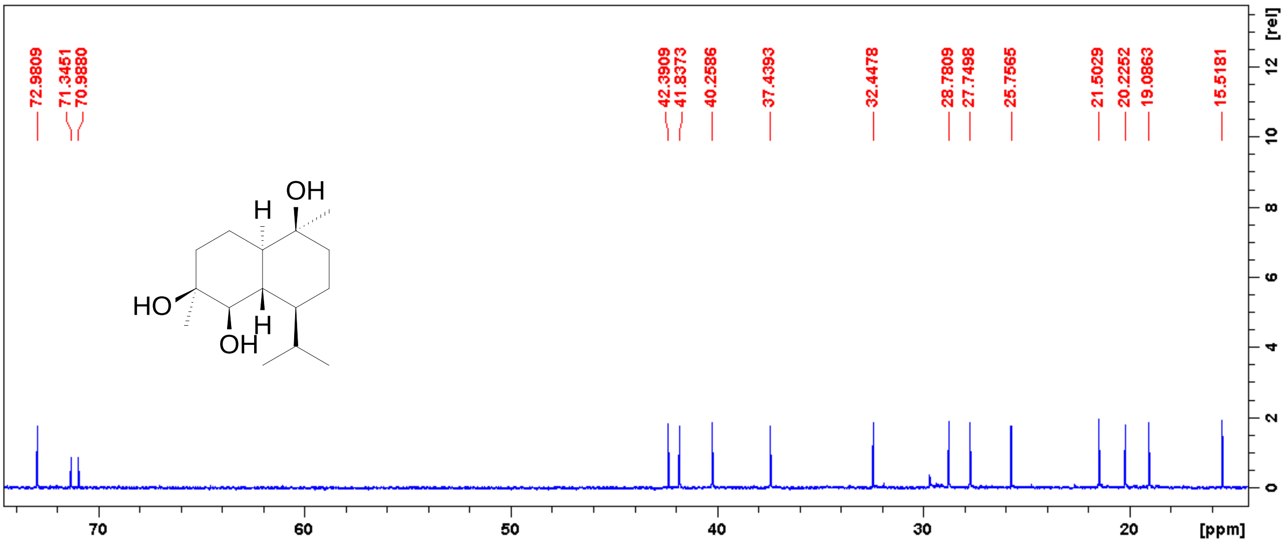

Figure S60. Expanded view of the carbon region (δ_C 74.4–14.3 ppm) in the ^{13}C NMR spectrum of **cadinane-4β,5α,10β-triol** (**9**) recorded in $CDCl_3$ at 125 MHz.

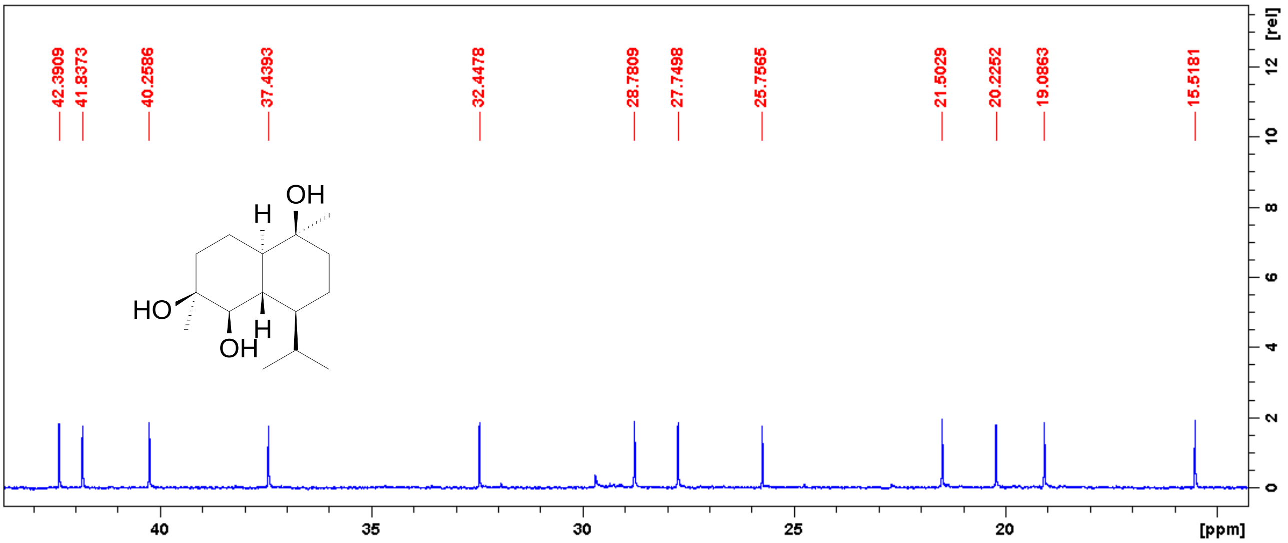

Figure S61. Expanded view of the carbon region (δ_C 43.5–14.3 ppm) in the ^{13}C NMR spectrum of **cadinane-4β,5α,10β-triol** (**9**) recorded in $CDCl_3$ at 125 MHz.

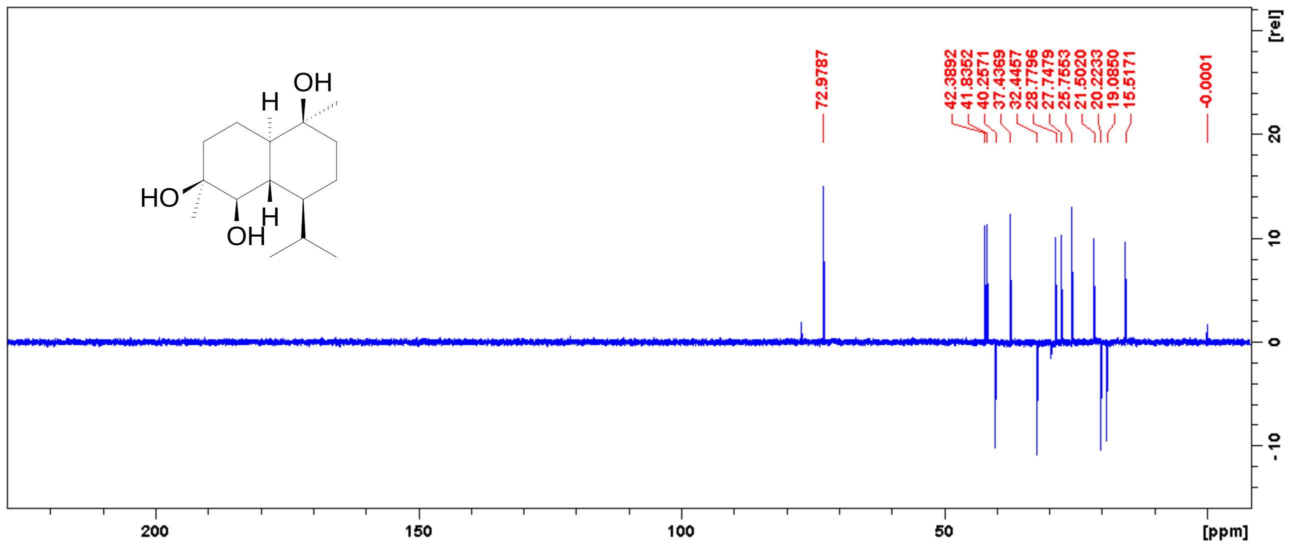

Figure S62. Full DEPT-135 ^{13}C NMR spectrum of **cadinane-4β,5α,10β-triol** (**9**) recorded in $CDCl_3$ at 125 MHz.

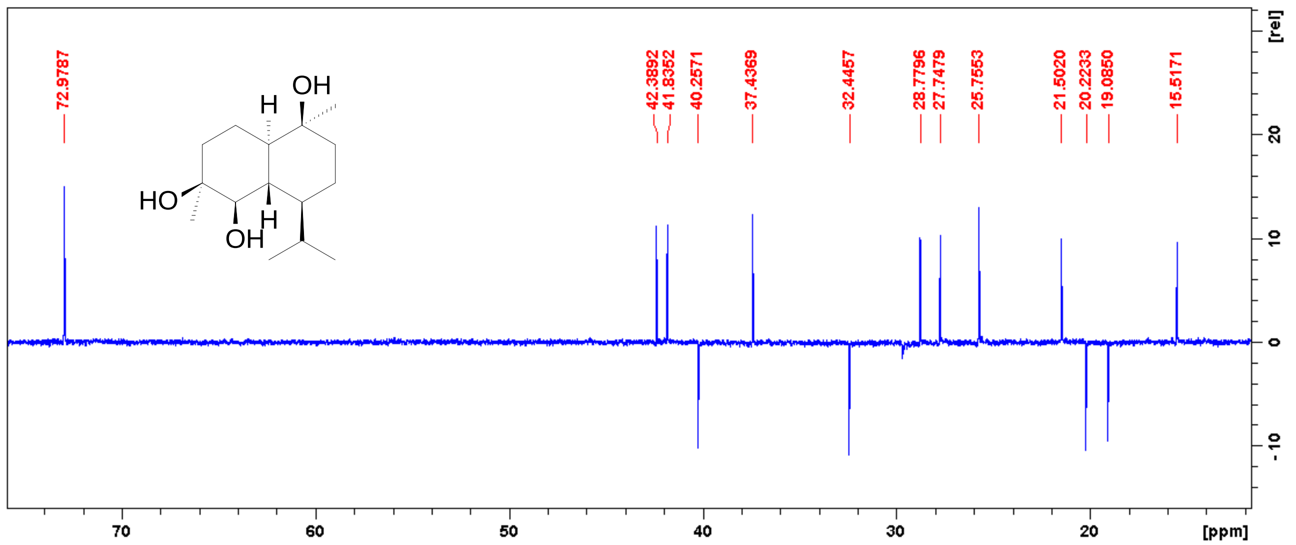

Figure S63. Expanded view of the carbon region (δ_C 75.8–11.9 ppm) in the DEPT-135 ^{13}C NMR spectrum of **cadinane-4β,5α,10β-triol** (**9**) recorded in $CDCl_3$ at 125 MHz.

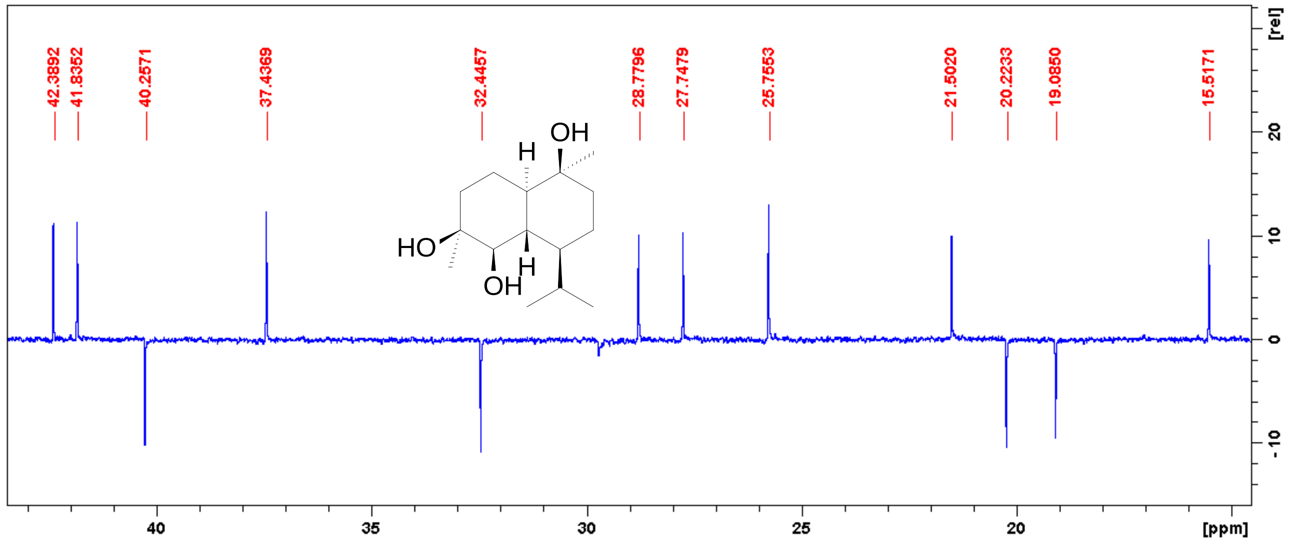

Figure S64. Expanded view of the carbon region (δ_C 43.3–14.6 ppm) in the DEPT-135 ^{13}C NMR spectrum of **cadinane-4β,5α,10β-triol** (**9**) recorded in $CDCl_3$ at 125 MHz.

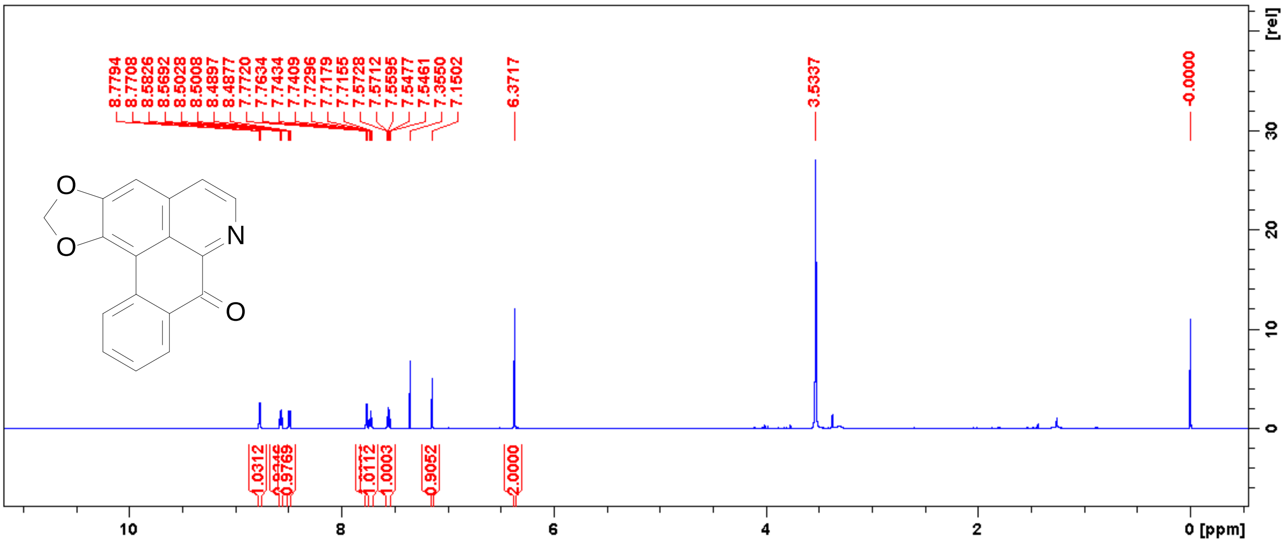

Figure S65. Full ^{1}H NMR spectrum of **liriodenine** (**10**) recorded in $CDCl_3$ with a drop of CD_3OD at 600 MHz.

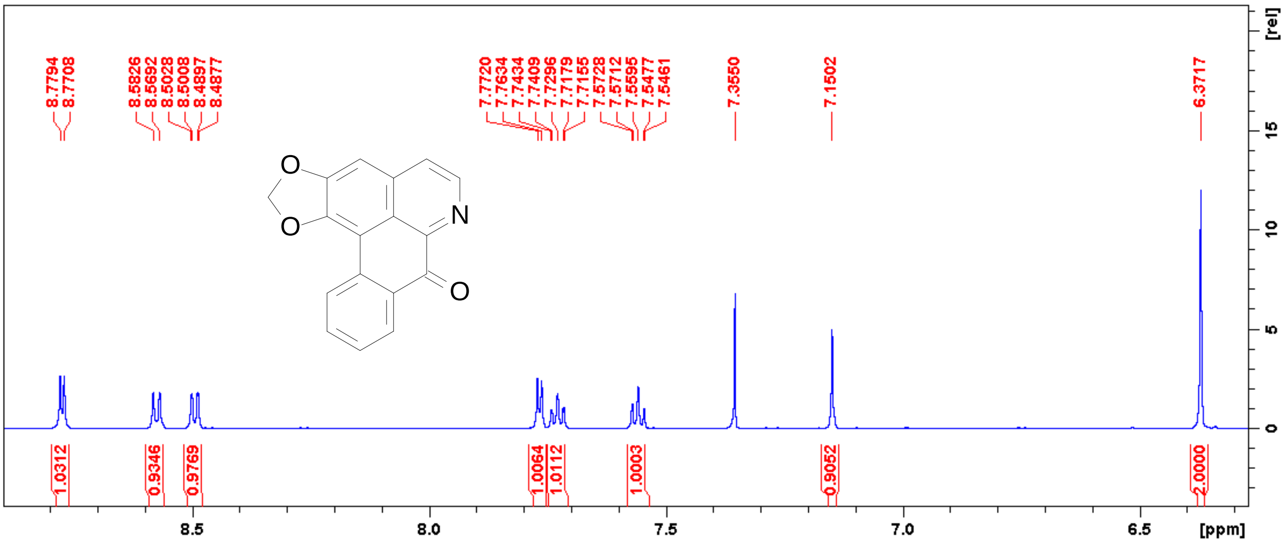

Figure S66. Expanded view 1 of the aromatic proton region in the ^{1}H NMR spectrum of **liriodenine** (**10**) recorded in $CDCl_3$ with a drop of CD_3OD at 600 MHz.

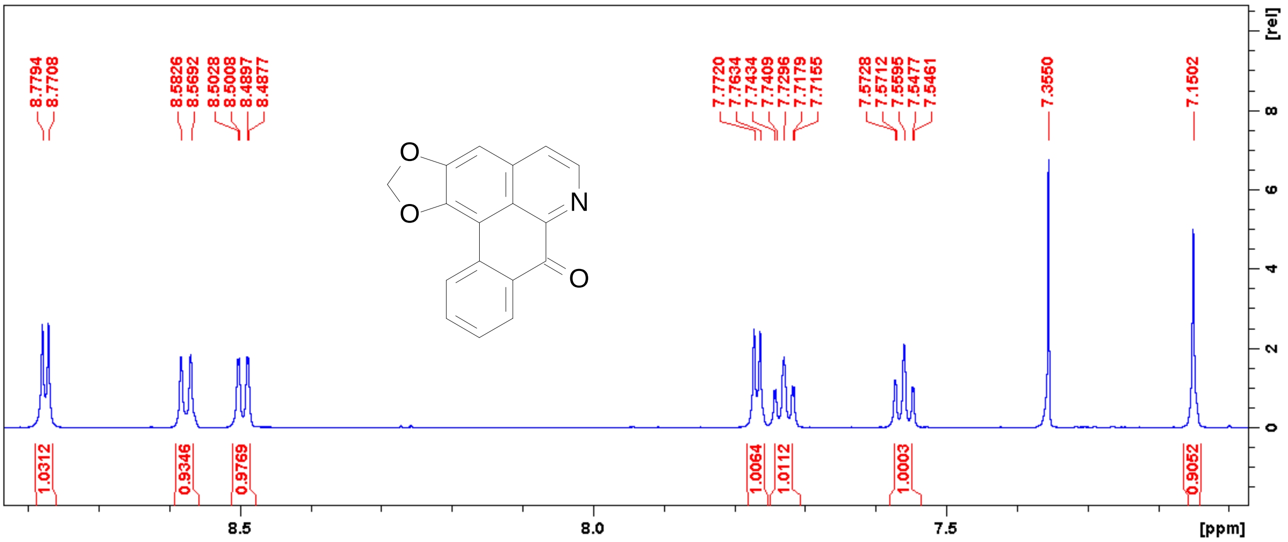

Figure S67. Expanded view 2 of the aromatic proton region in the ^{1}H NMR spectrum of **liriodenine** (**10**) recorded in $CDCl_3$ with a drop of CD_3OD at 600 MHz.

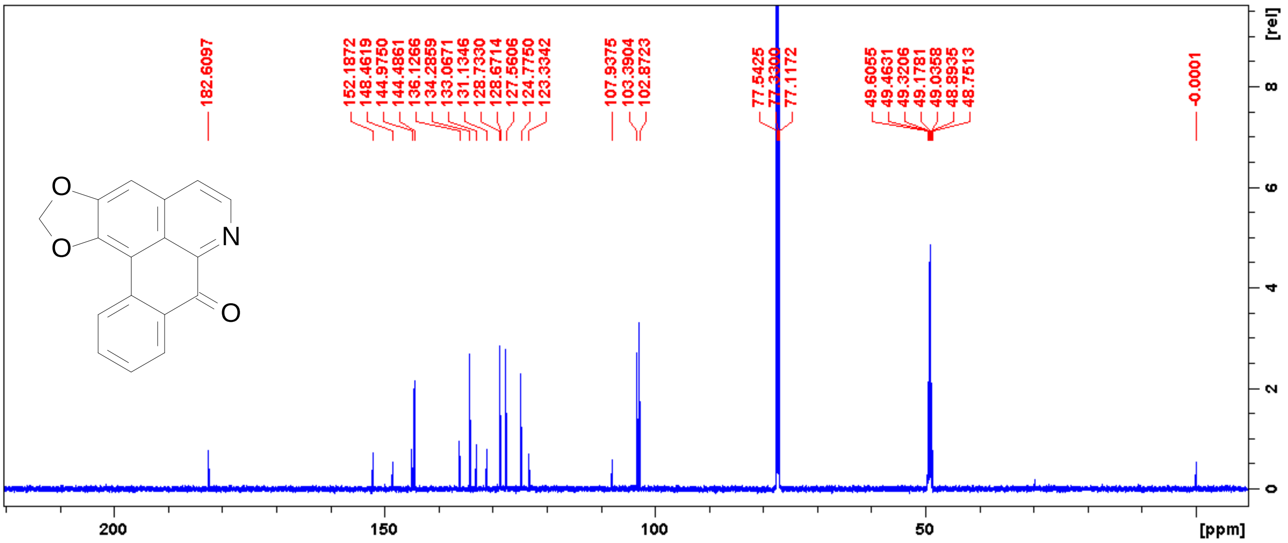

Figure S68. Full ^{13}C NMR spectrum of **liriodenine** (**10**) recorded in $CDCl_3$ with a drop of CD_3OD at 150 MHz.

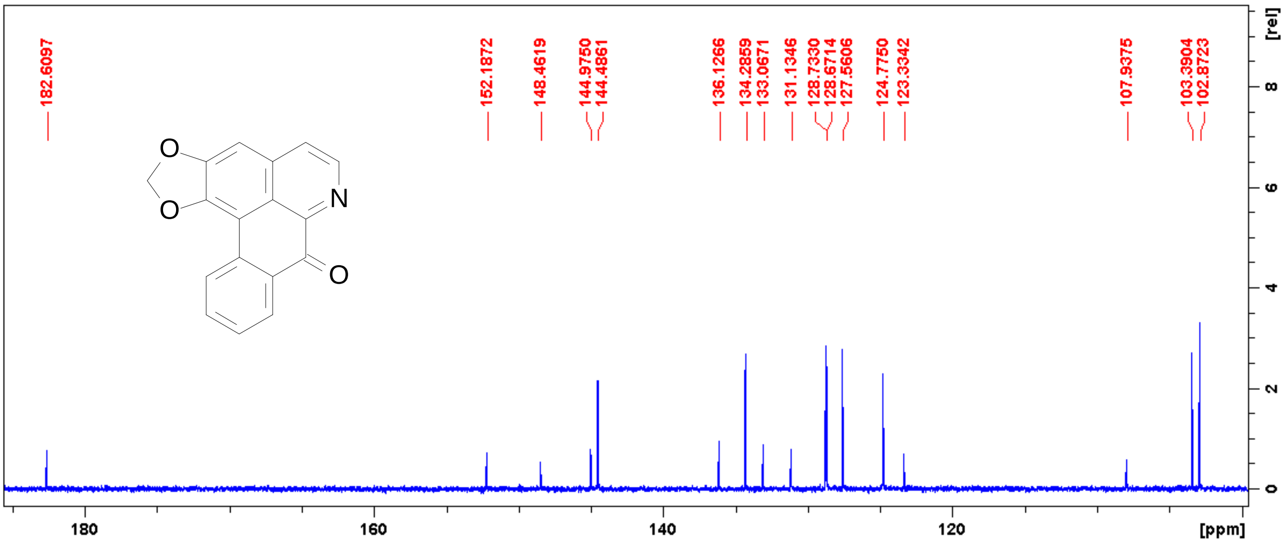

Figure S69. Expanded view of the carbon region (δ_C 185.3–99.9 ppm) in the ^{13}C NMR spectrum of **liriodenine** (**10**) recorded in $CDCl_3$ with a drop of CD_3OD at 150 MHz.

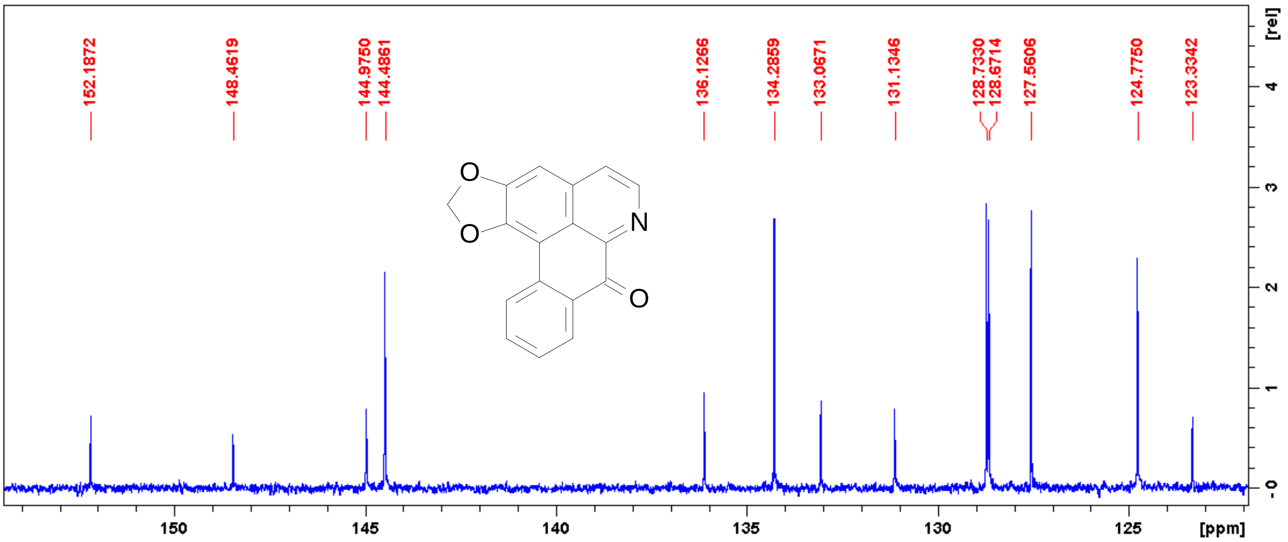

Figure S70. Expanded view of the carbon region (δ_C 154.4–122.0 ppm) in the ^{13}C NMR spectrum of **liriodenine** (**10**) recorded in $CDCl_3$ with a drop of CD_3OD at 150 MHz.

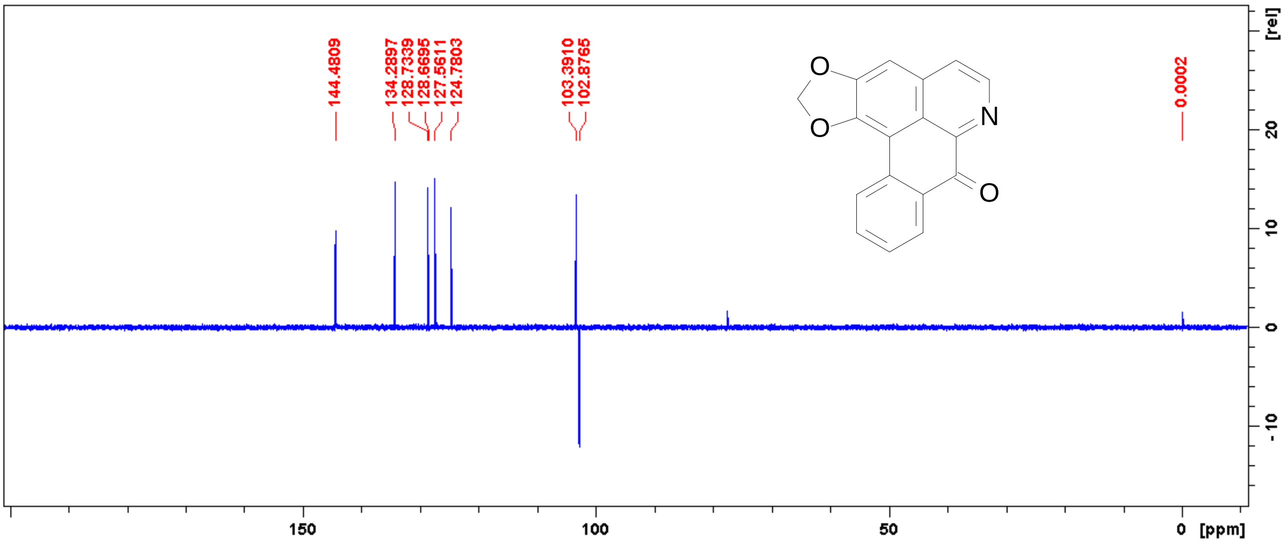

Figure S71. Full DEPT-135 ^{13}C NMR spectrum of **liriodenine** (**10**) recorded in $CDCl_3$ with a drop of CD_3OD at 150 MHz.

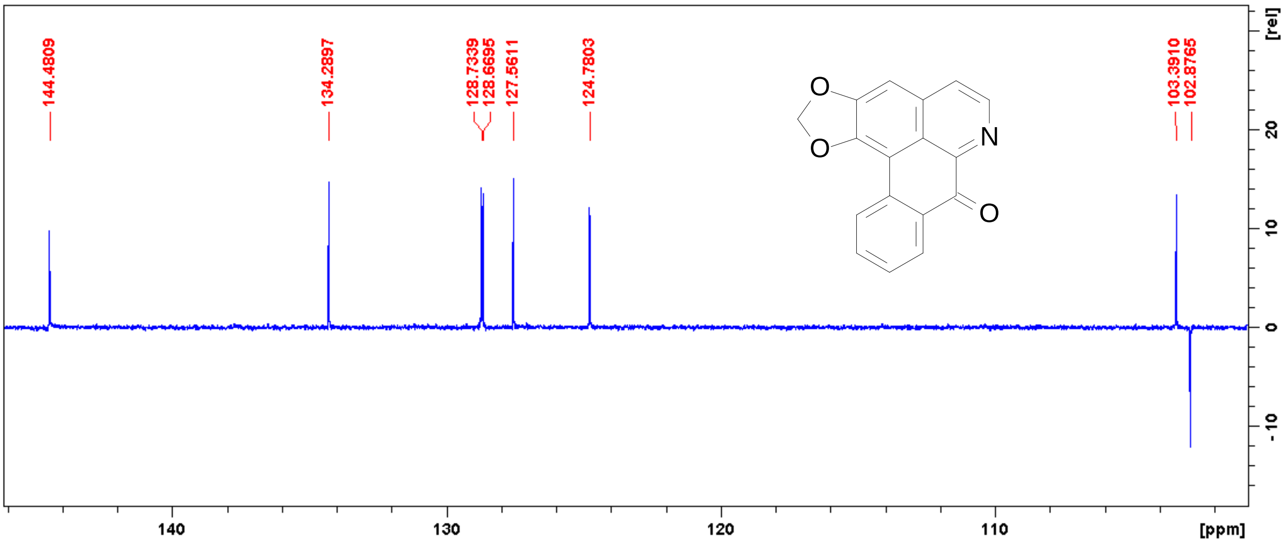

Figure S72. Expanded view of the carbon region (δ_C 146.1–100.9 ppm) in the DEPT-135 ^{13}C NMR spectrum of **liriodenine** (**10**) recorded in $CDCl_3$ with a drop of CD_3OD at 150 MHz.

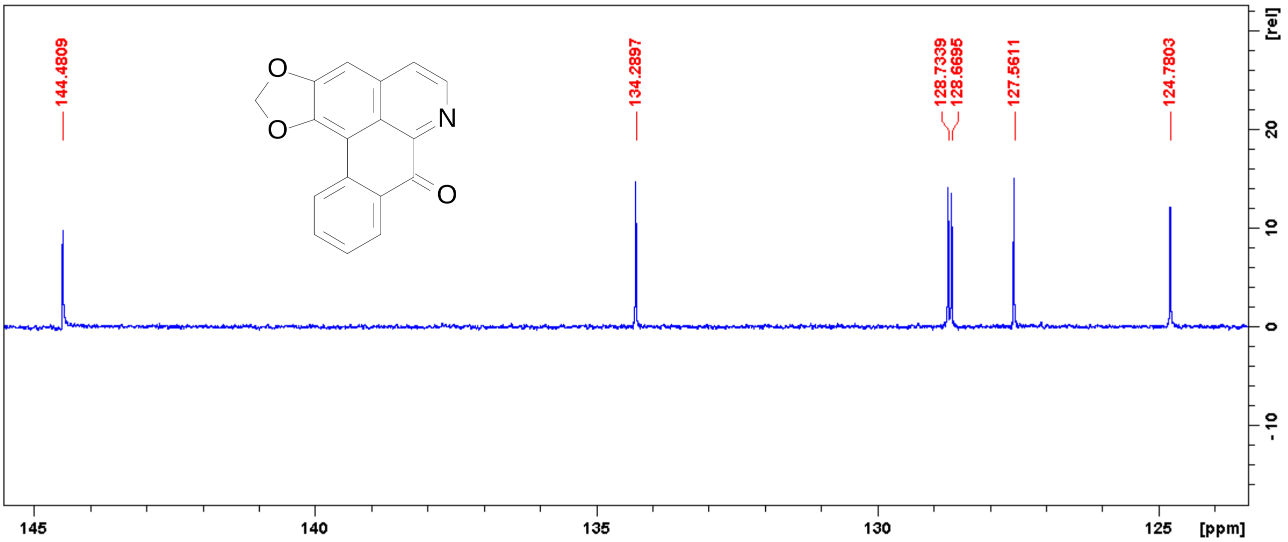

Figure S73. Expanded view of the carbon region (δ_C 145.4–123.5 ppm) in the DEPT-135 ^{13}C NMR spectrum of **liriodenine** (**10**) recorded in $CDCl_3$ with a drop of CD_3OD at 150 MHz.

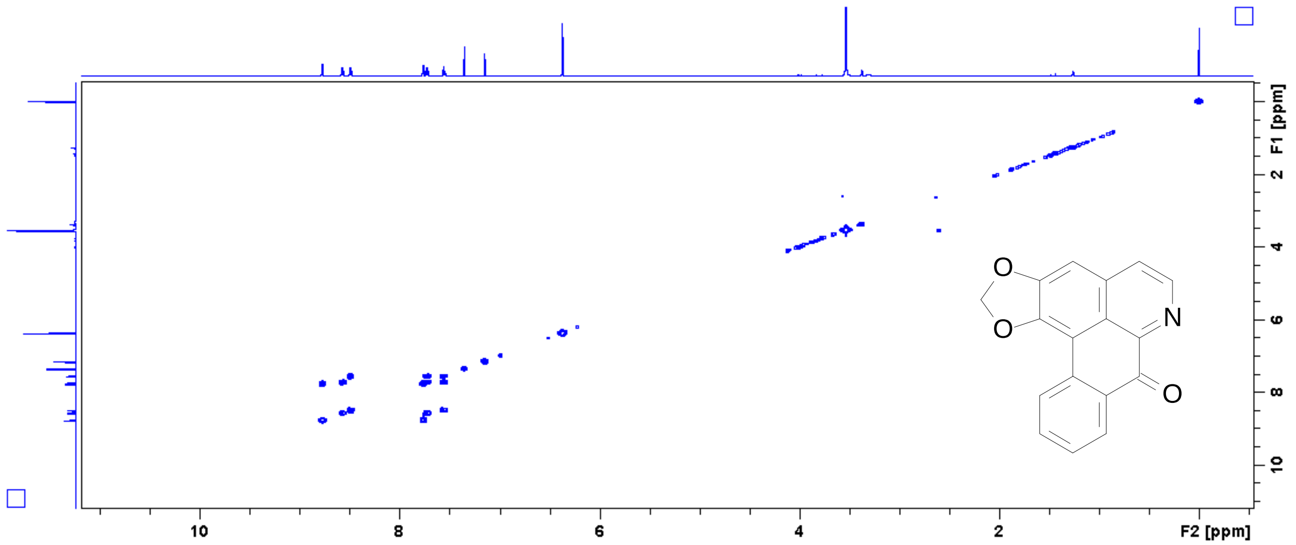

Figure S74. ^{1}H–^{1}H COSY correlation map of **liriodenine** (**10**) recorded in $CDCl_3$ with a drop of CD_3OD at 600 MHz.

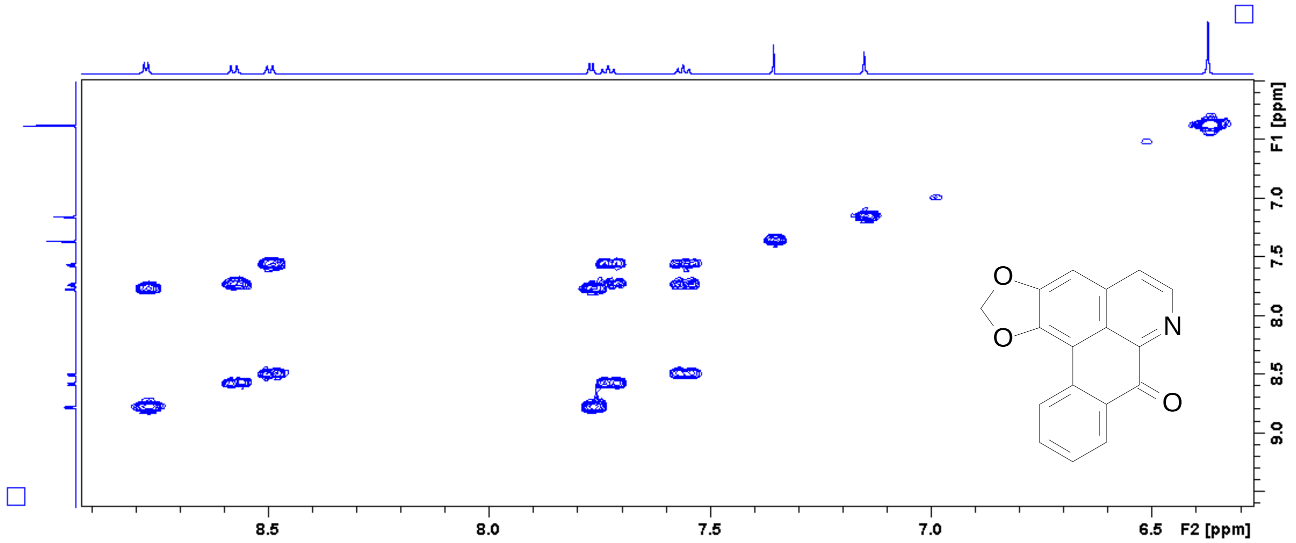

Figure S75. Expanded view 1 of the ^{1}H–^{1}H COSY correlation map of **liriodenine** (**10**) recorded in $CDCl_3$ with a drop of CD_3OD at 600 MHz.

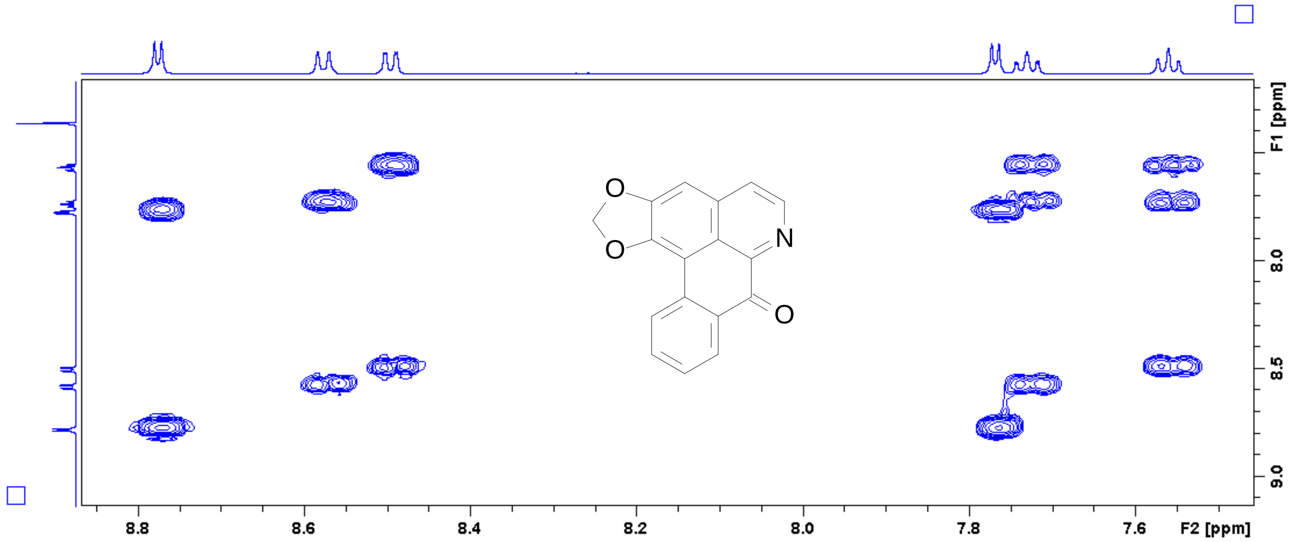

Figure S76. Expanded view 2 of the ^{1}H–^{1}H COSY correlation map of **liriodenine** (**10**) recorded in $CDCl_3$ with a drop of CD_3OD at 600 MHz.

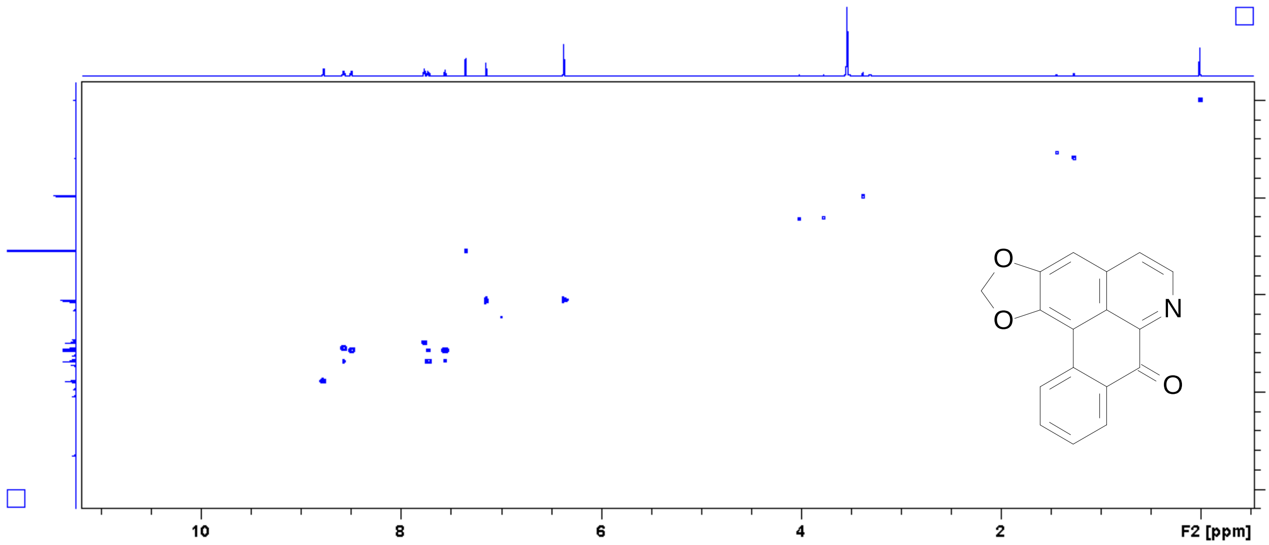

Figure S77. One-bond ^{1}H–^{13}C HSQC correlation map of **liriodenine** (**10**) recorded in $CDCl_3$ with a drop of CD_3OD at 600 MHz (^{1}H) and 150 MHz (^{13}C).

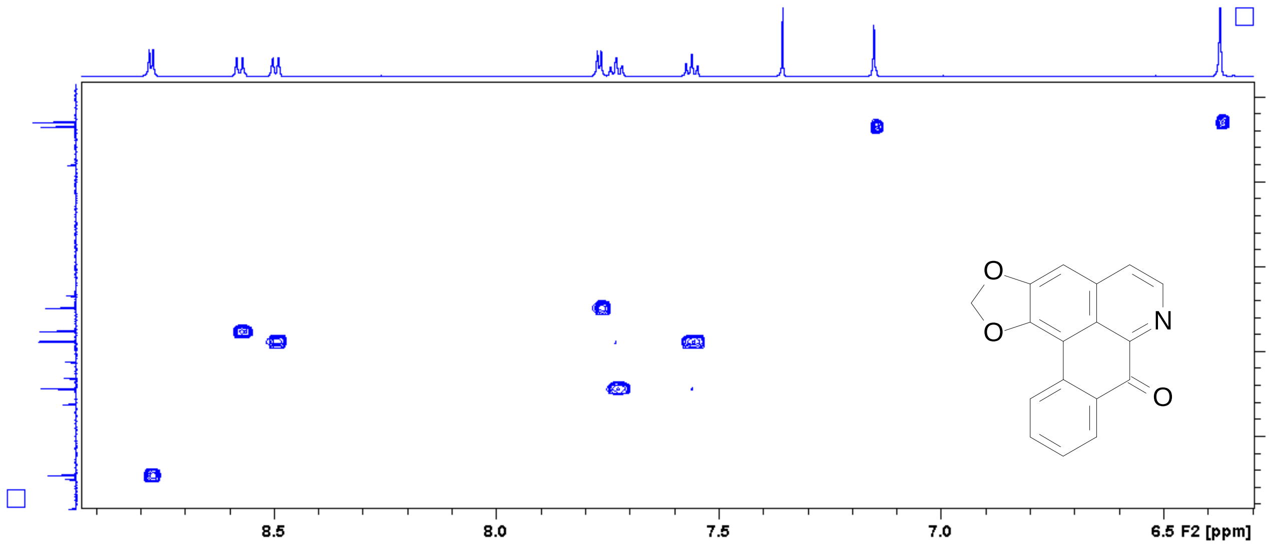

Figure S78. Expanded view 1 of the one-bond ^{1}H–^{13}C HSQC correlation map of **liriodenine** (**10**) recorded in $CDCl_3$ with a drop of CD_3OD at 600 MHz (^{1}H) and 150 MHz (^{13}C).

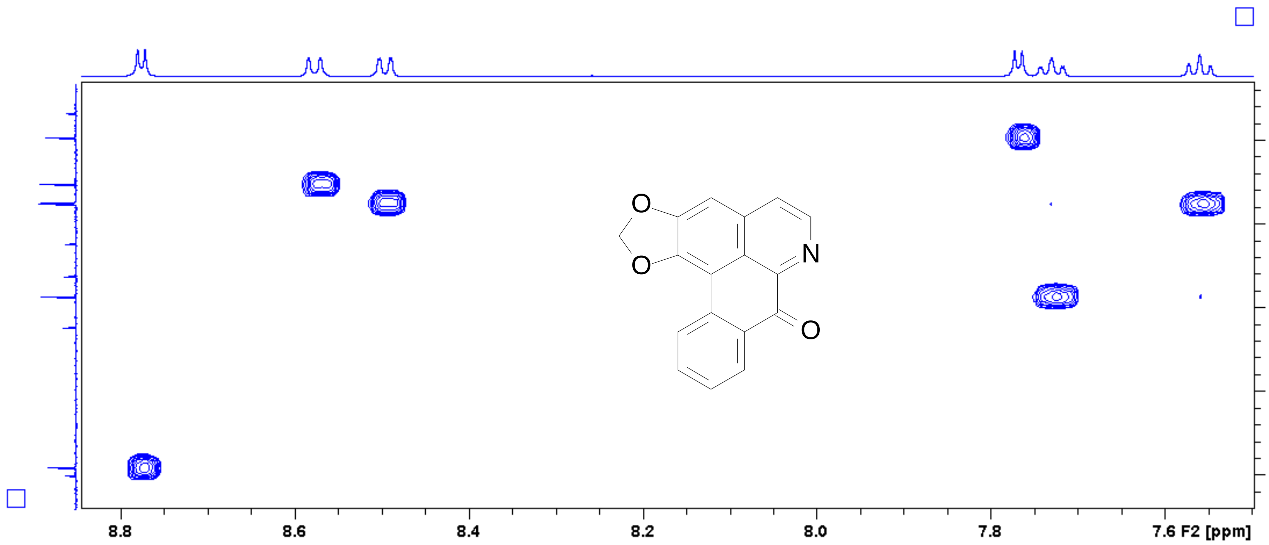

Figure S79. Expanded view 2 of the one-bond ^{1}H–^{13}C HSQC correlation of **liriodenine** (**10**) recorded in $CDCl_3$ with a drop of CD_3OD at 600 MHz (^{1}H) and 150 MHz (^{13}C).

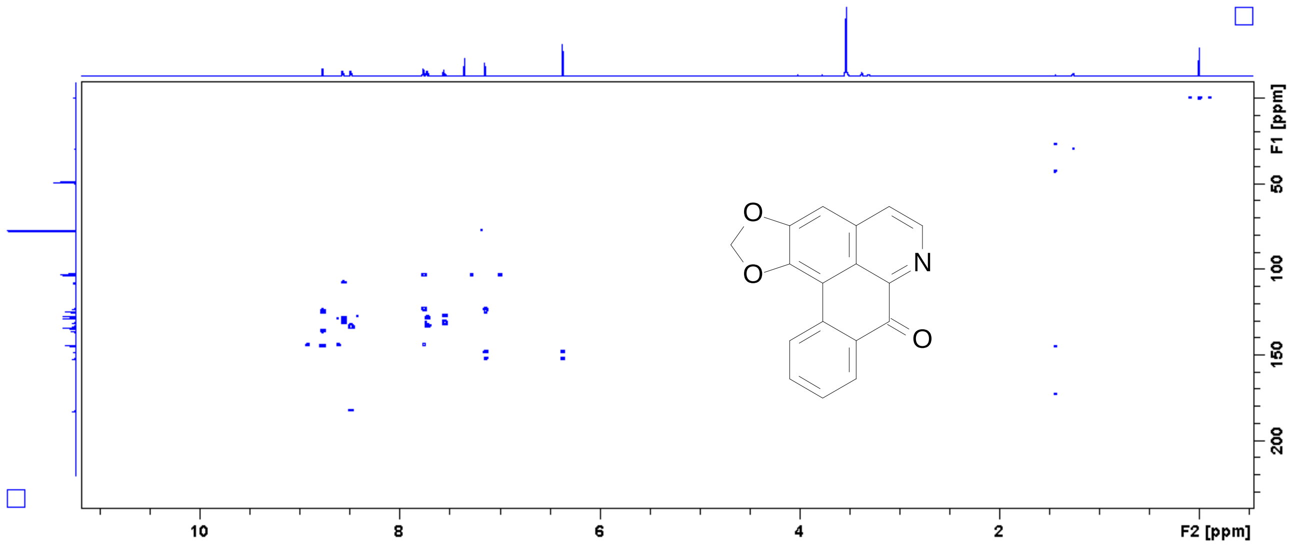

Figure S80. Long-range ^{1}H–^{13}C HMBC correlation map of **liriodenine** (**10**) recorded in $CDCl_3$ with a drop of CD_3OD at 600 MHz (^{1}H) and 150 MHz (^{13}C).

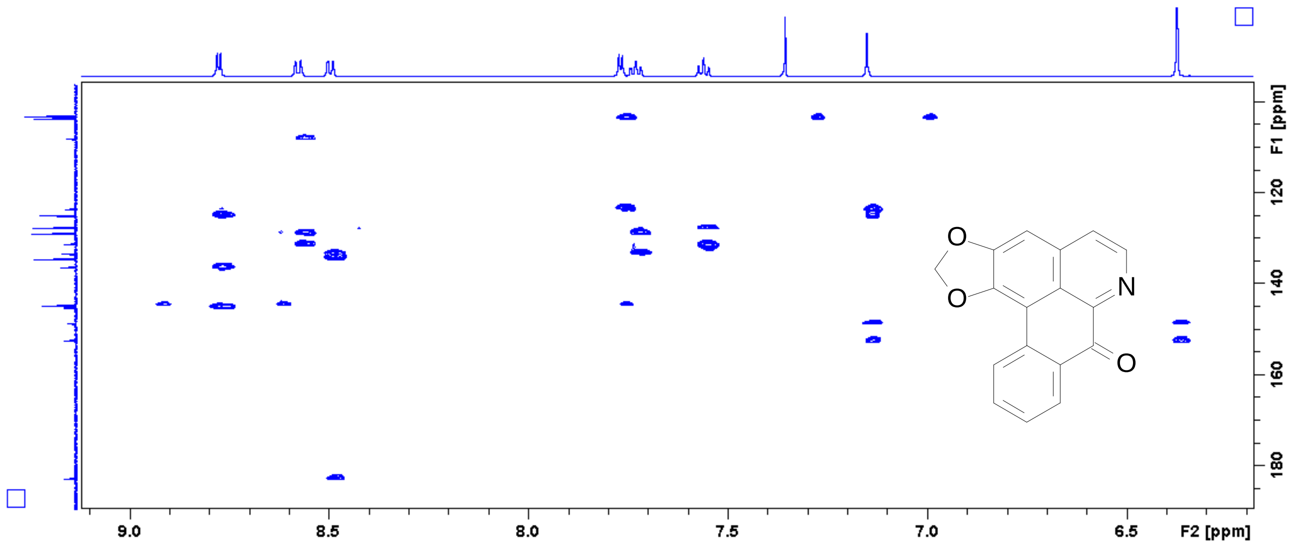

Figure S81. Expanded view 1 of the long-range ^{1}H–^{13}C HMBC correlation map of **liriodenine** (**10**) recorded in $CDCl_3$ with a drop of CD_3OD at 600 MHz (^{1}H) and 150 MHz (^{13}C).

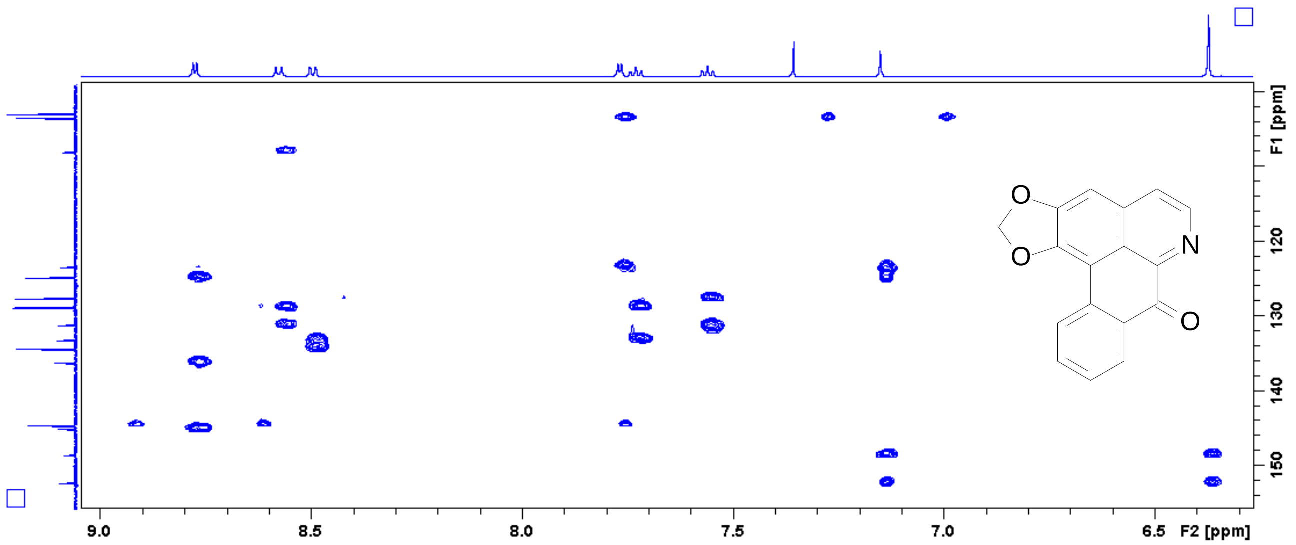

Figure S82. Expanded view 2 of the long-range ^{1}H–^{13}C HMBC correlation map of **liriodenine** (**10**) recorded in $CDCl_3$ with a drop of CD_3OD at 600 MHz (^{1}H) and 150 MHz (^{13}C).

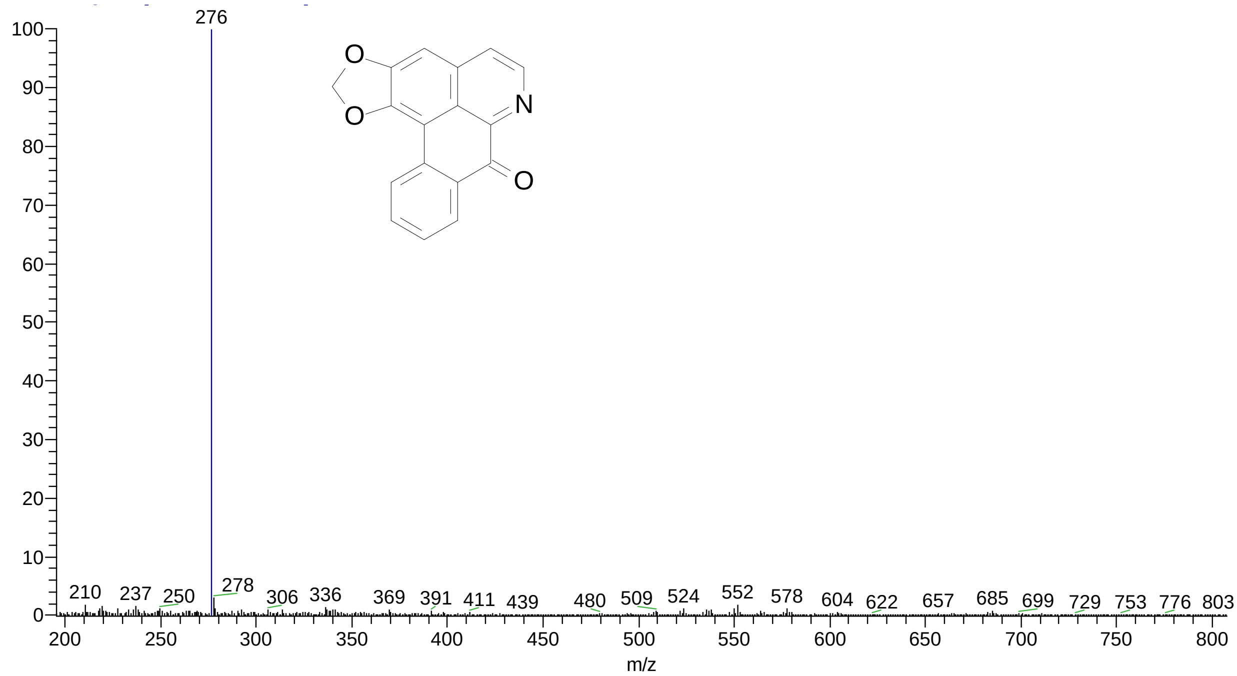

Figure S83. LR-APCI(+)-MS spectrum of **liriodenine** (**10**), showing the protonated molecular ion at *m/z* 276 $[M + H]^+$.

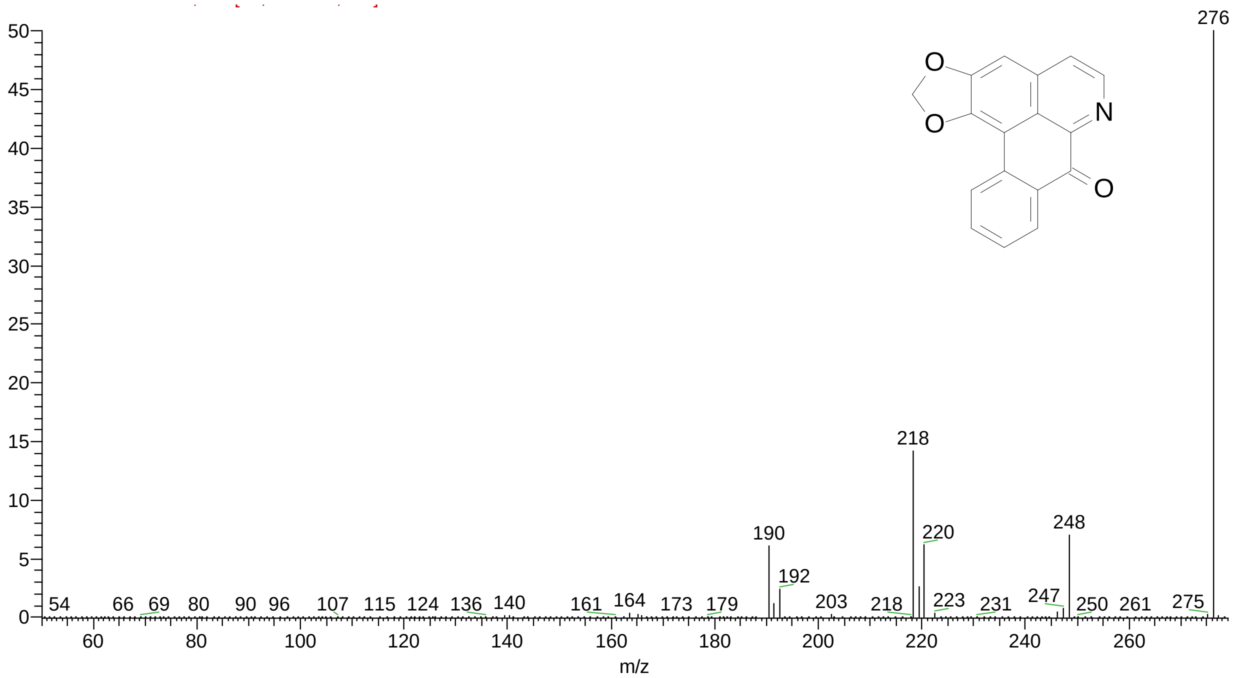

Figure S84. LR-APCI(+)-MS/MS spectrum of the protonated molecule of **liriodenine** (**10**) at *m/z* 276 $[M + H]^+$.

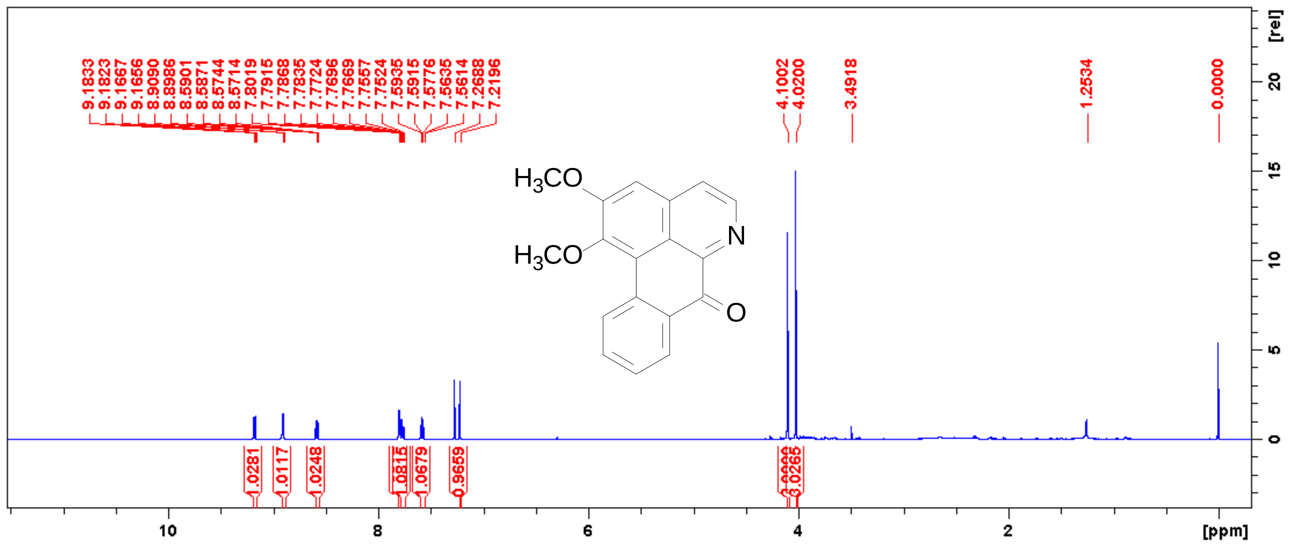

Figure S85. Full ^{1}H NMR spectrum of **lysicamine** (**11**) recorded in $CDCl_3$ at 500 MHz.

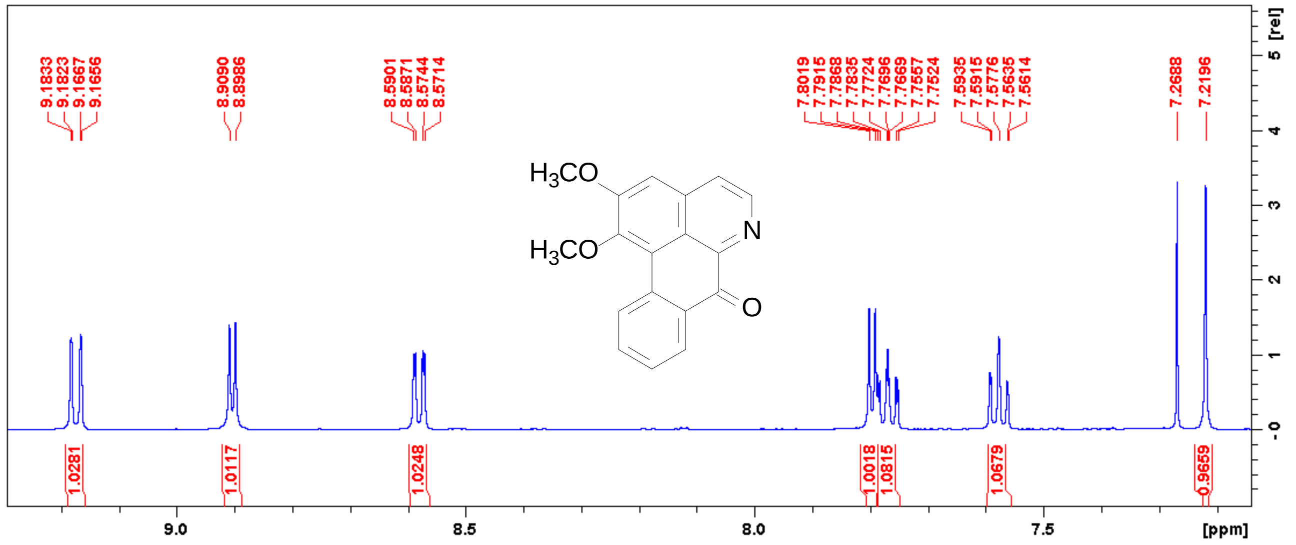

Figure S86. Expanded view 1 of the aromatic proton region in the ^{1}H NMR spectrum of **lysicamine** (**11**) recorded in $CDCl_3$ at 500 MHz.

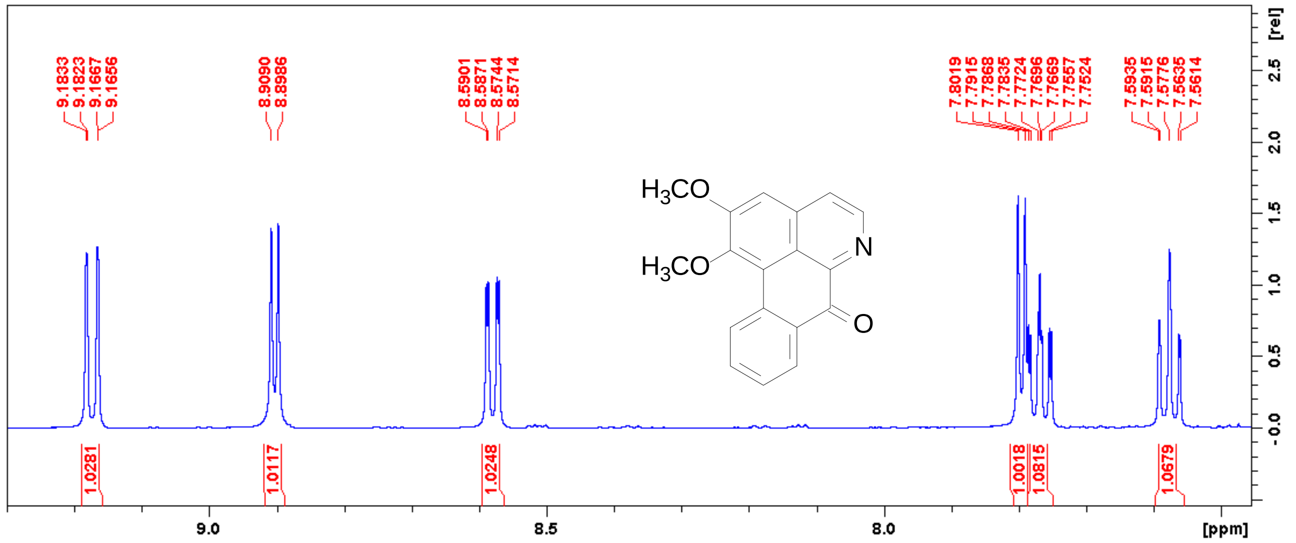

Figure S87. Expanded view 2 of the aromatic proton region in the ^{1}H NMR spectrum of **lysicamine** (**11**) recorded in $CDCl_3$ at 500 MHz.

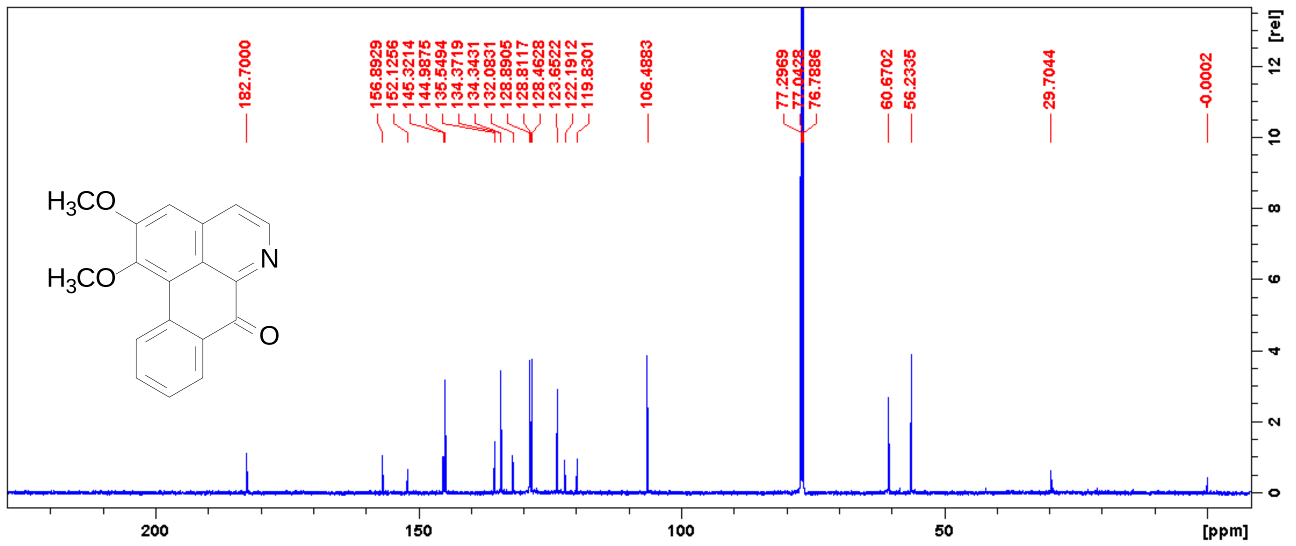

Figure S88. Full ^{13}C NMR spectrum of **lysicamine (11)** recorded in $CDCl_3$ at 125 MHz.

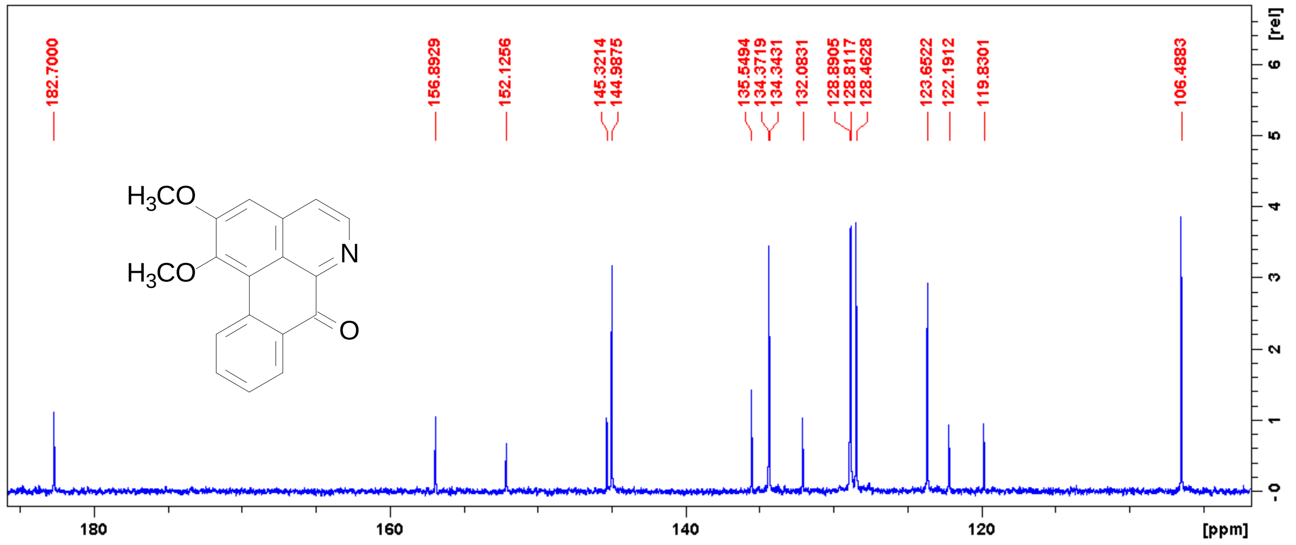

Figure S89. Expanded view of the carbon region (δ_C 186.0–102.6 ppm) in the ^{13}C NMR spectrum of **lysicamine (11)** recorded in $CDCl_3$ at 125 MHz.

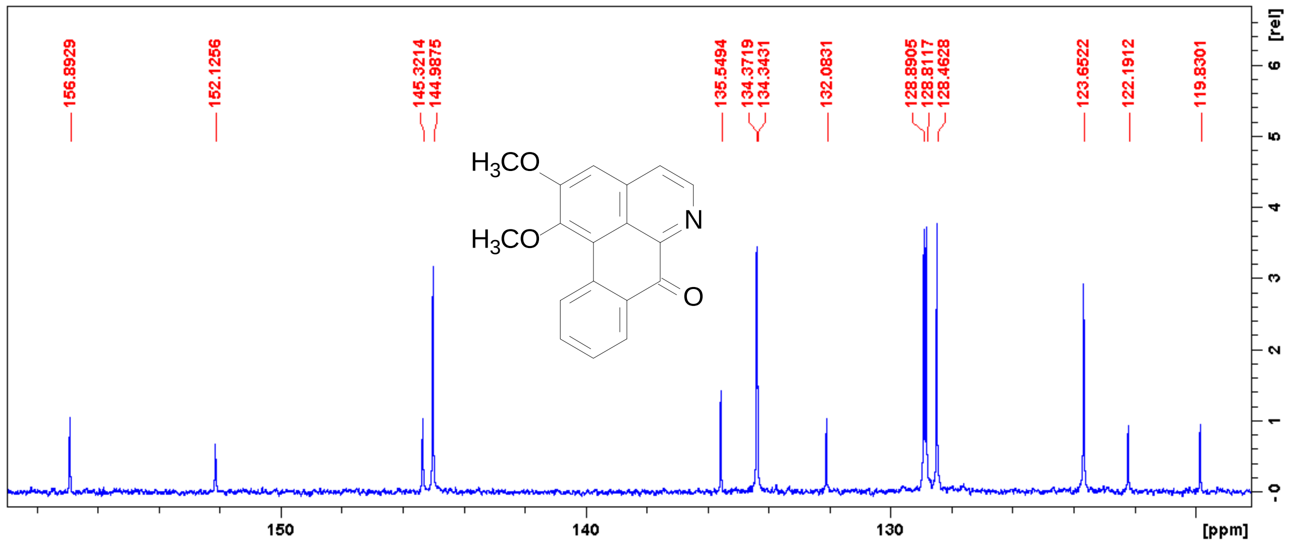

Figure S90. Expanded view of the carbon region (δ_C 158.8–118.2 ppm) in the ^{13}C NMR spectrum of **lysicamine (11)** recorded in $CDCl_3$ at 125 MHz.

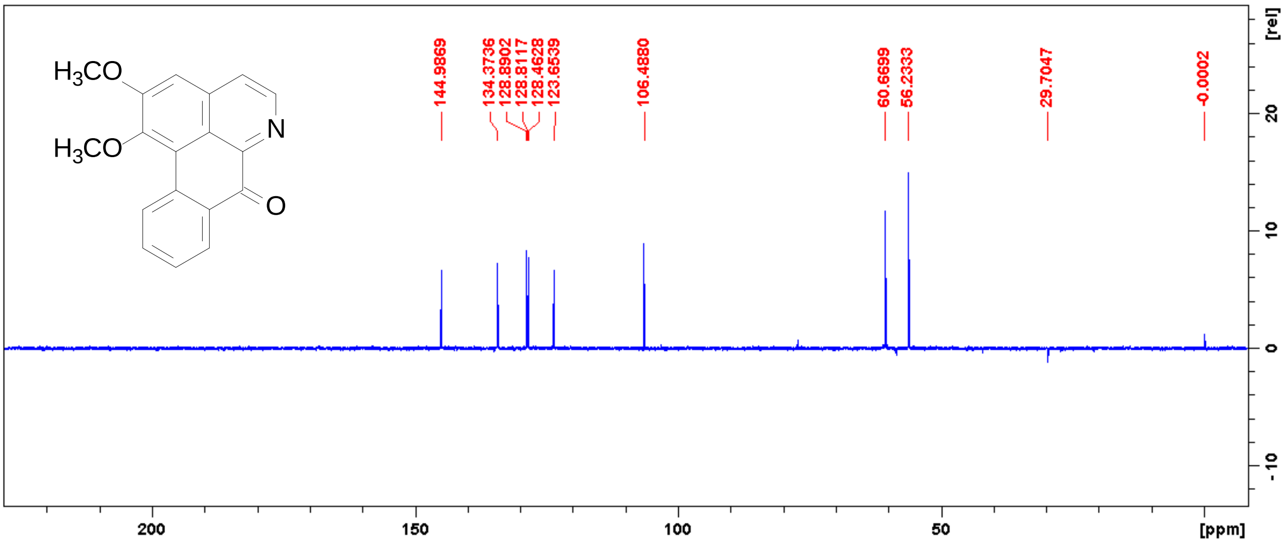

Figure S91. Full DEPT-135 ^{13}C NMR spectrum of **lysicamine** (**11**) recorded in $CDCl_3$ at 125 MHz.

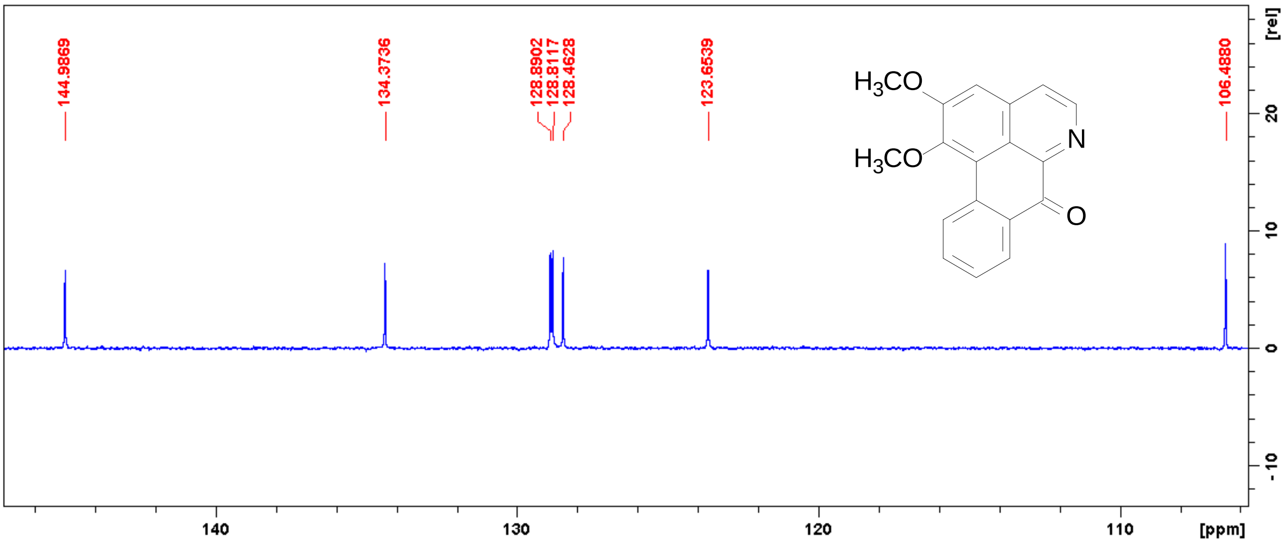

Figure S92. Expanded view of the carbon region (δ_C 146.9–105.9 ppm) in the DEPT-135 ^{13}C NMR spectrum of **lysicamine** (**11**) recorded in $CDCl_3$ at 125 MHz.

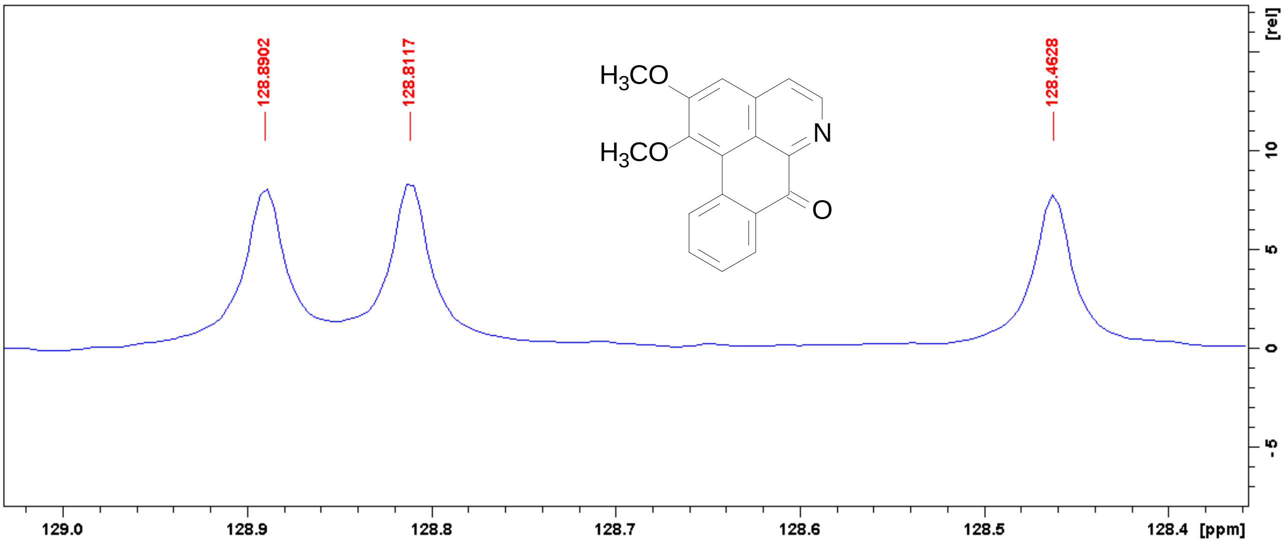

Figure S93. Expanded view of the carbon region (δ_C 129.0–128.3 ppm) in the DEPT-135 ^{13}C NMR spectrum of **lysicamine** (**11**) recorded in $CDCl_3$ at 125 MHz.

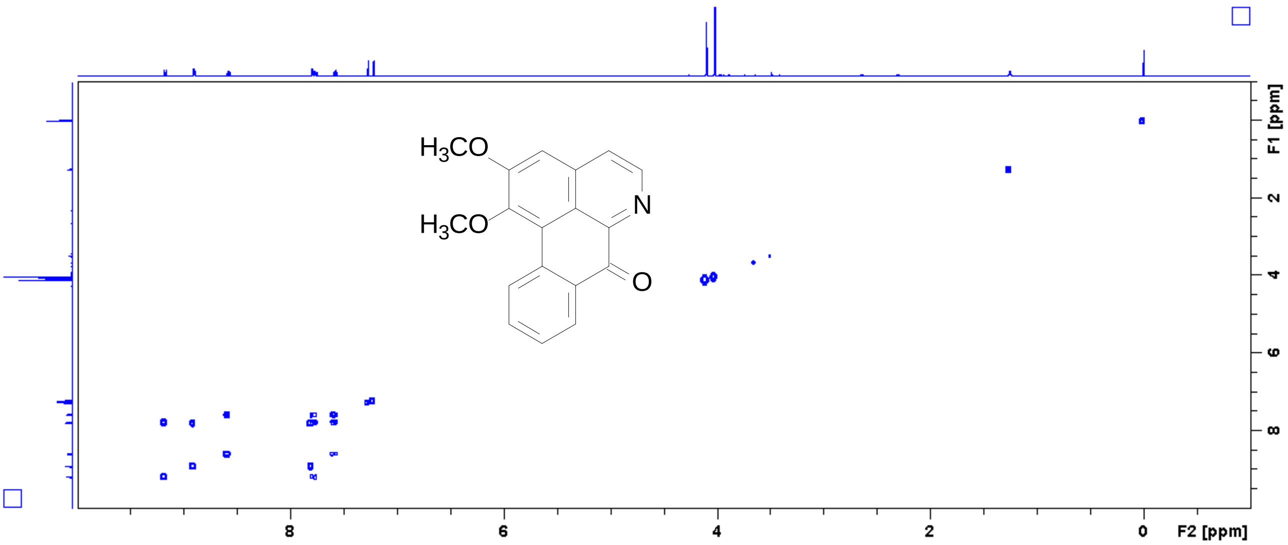

Figure S94. ^{1}H–^{1}H COSY correlation map of **lysicamine** (**11**) recorded in $CDCl_3$ at 500 MHz.

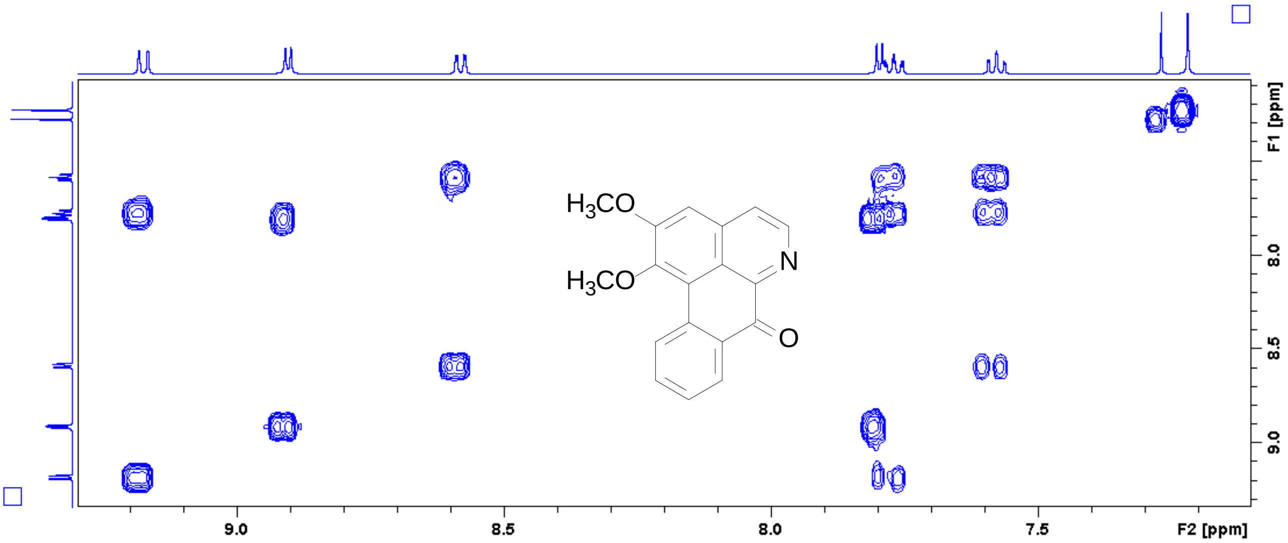

Figure S95. Expanded view 1 of the ^{1}H–^{1}H COSY correlation map of **lysicamine** (**11**) recorded in $CDCl_3$ at 500 MHz.

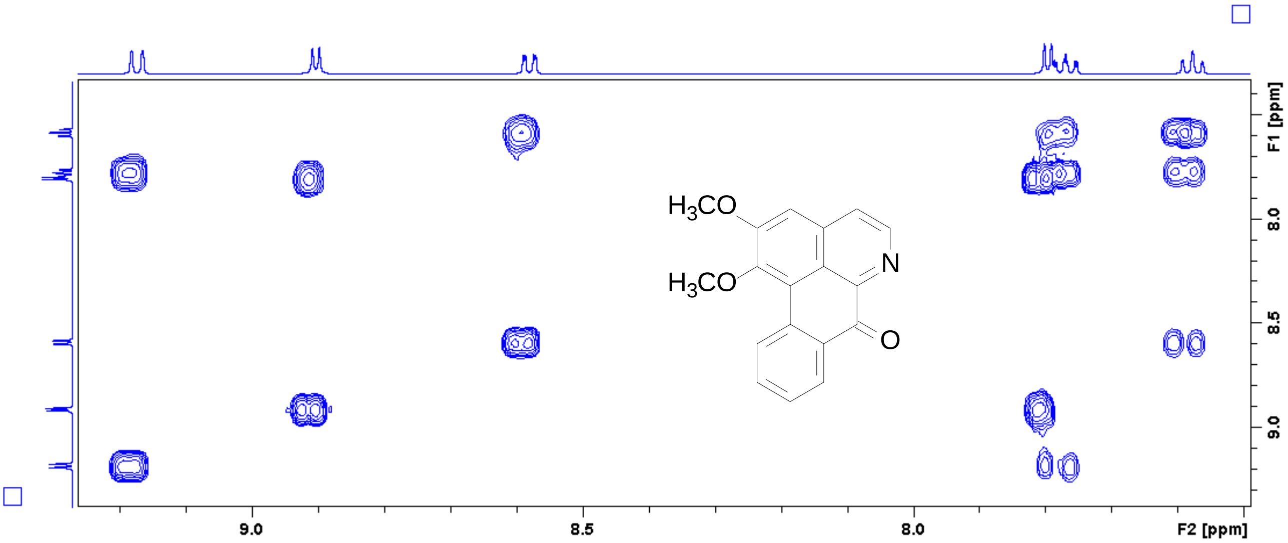

Figure S96. Expanded view 2 of the ^{1}H–^{1}H COSY correlation map of **lysicamine** (**11**) recorded in $CDCl_3$ at 500 MHz.

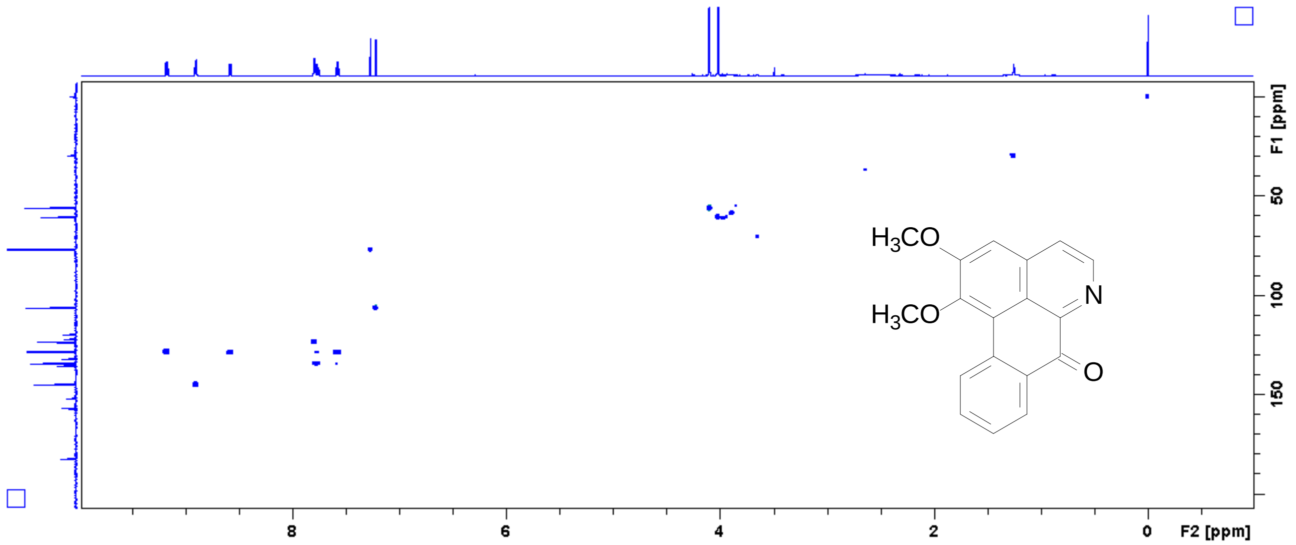

Figure S97. One-bond $^{1}H–^{13}C$ HSQC correlation map of **lysicamine** (**11**) recorded in $CDCl_3$ at 500 MHz (^{1}H) and 125 MHz (^{13}C).

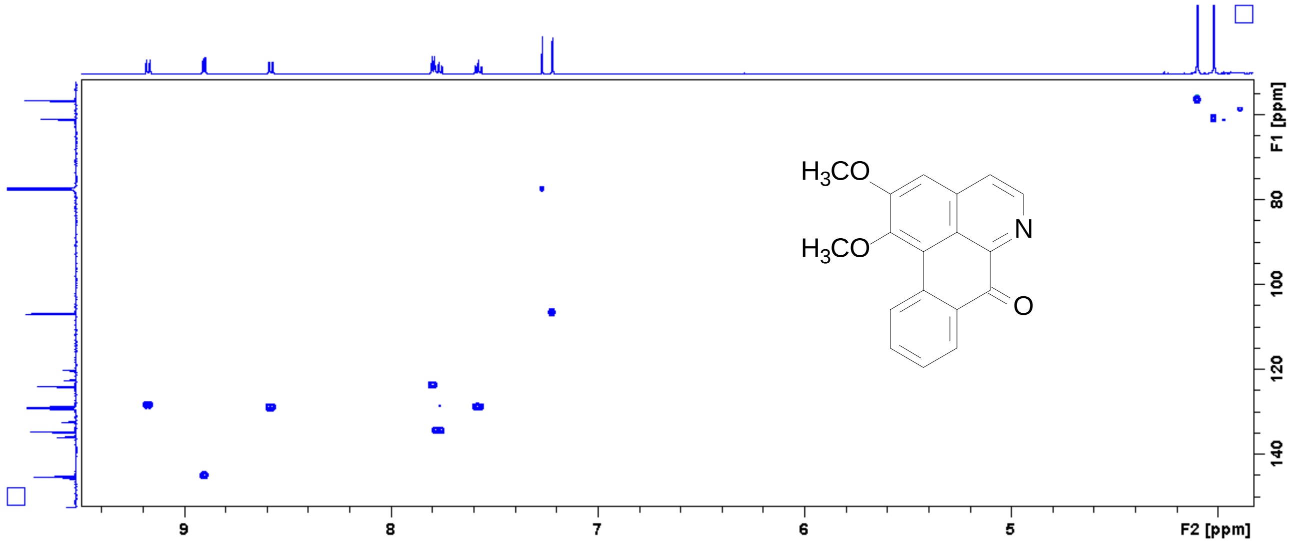

Figure S98. Expanded view 1 of the one-bond $^{1}H–^{13}C$ HSQC correlation map of **lysicamine** (**11**) recorded in $CDCl_3$ at 500 MHz (^{1}H) and 125 MHz (^{13}C).

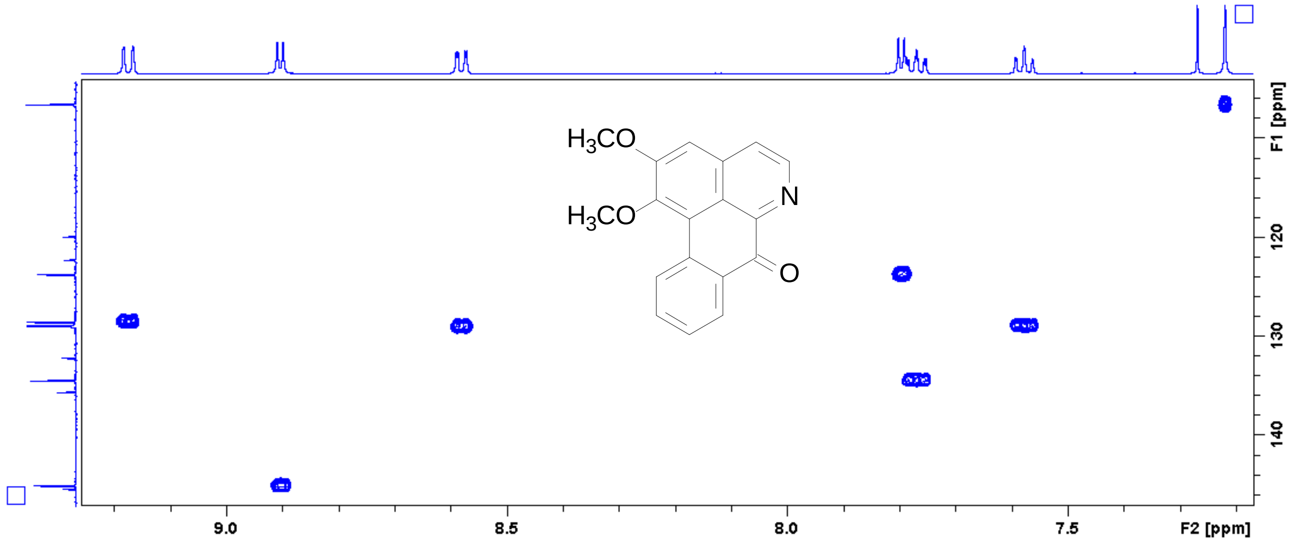

Figure S99. Expanded view 2 of the one-bond $^{1}H–^{13}C$ HSQC correlation of **lysicamine** (**11**) recorded in $CDCl_3$ at 500 MHz (^{1}H) and 125 MHz (^{13}C).

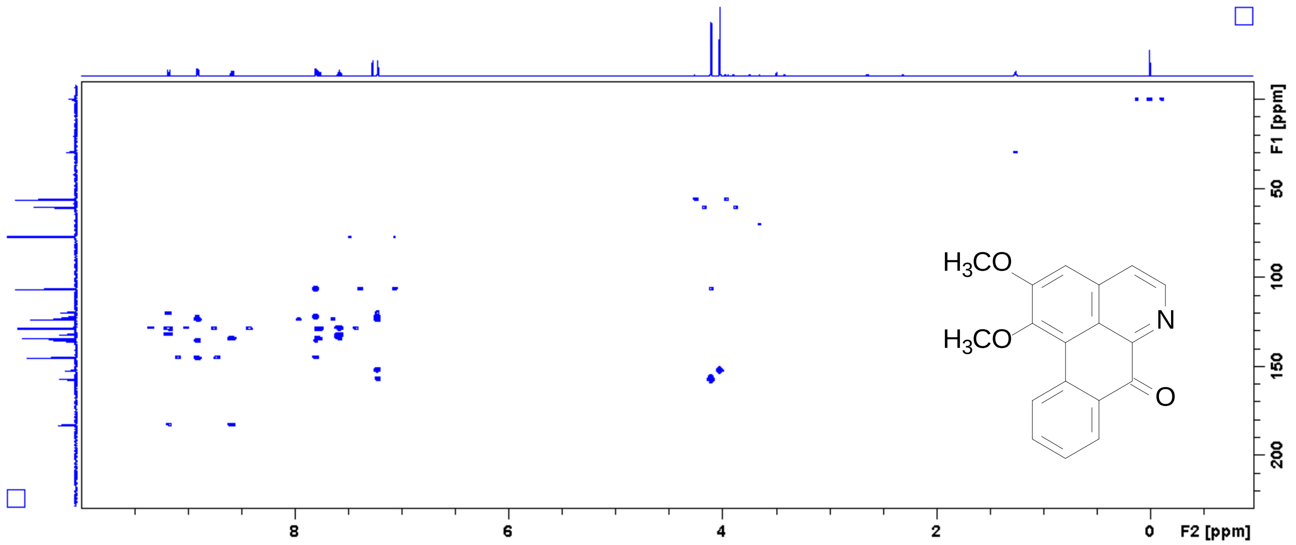

Figure S100. Long-range ^{1}H–^{13}C HMBC correlation map of **lysicamine** (**11**) recorded in $CDCl_3$ at 500 MHz (^{1}H) and 125 MHz (^{13}C).

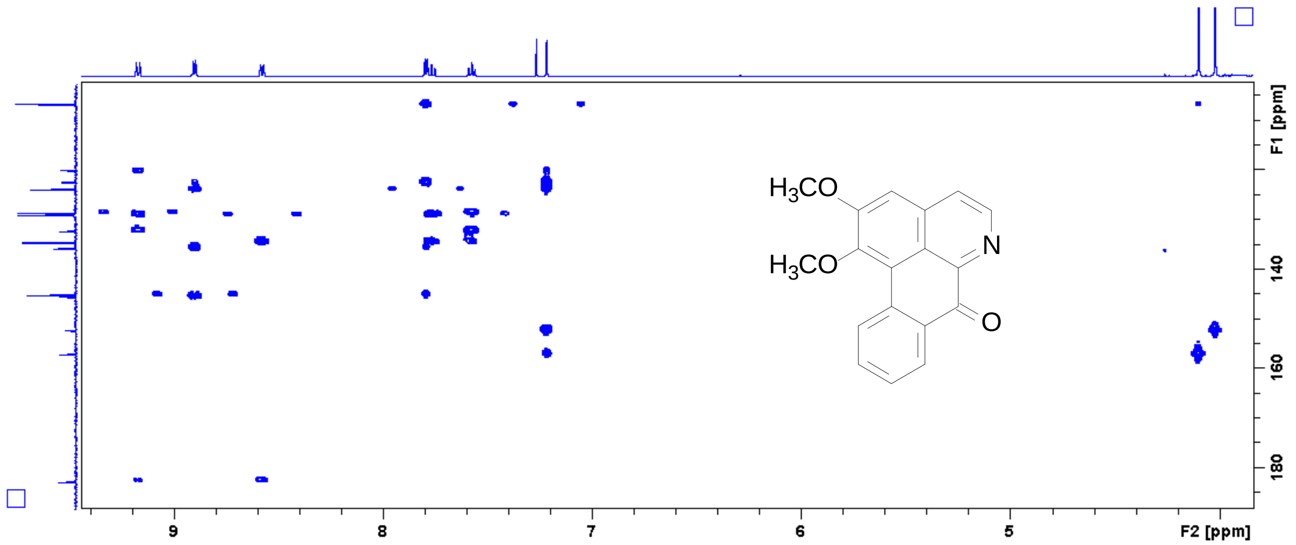

Figure S101. Expanded view 1 of the long-range ^{1}H–^{13}C HMBC correlation map of **lysicamine** (**11**) recorded in $CDCl_3$ at 500 MHz (^{1}H) and 125 MHz (^{13}C).

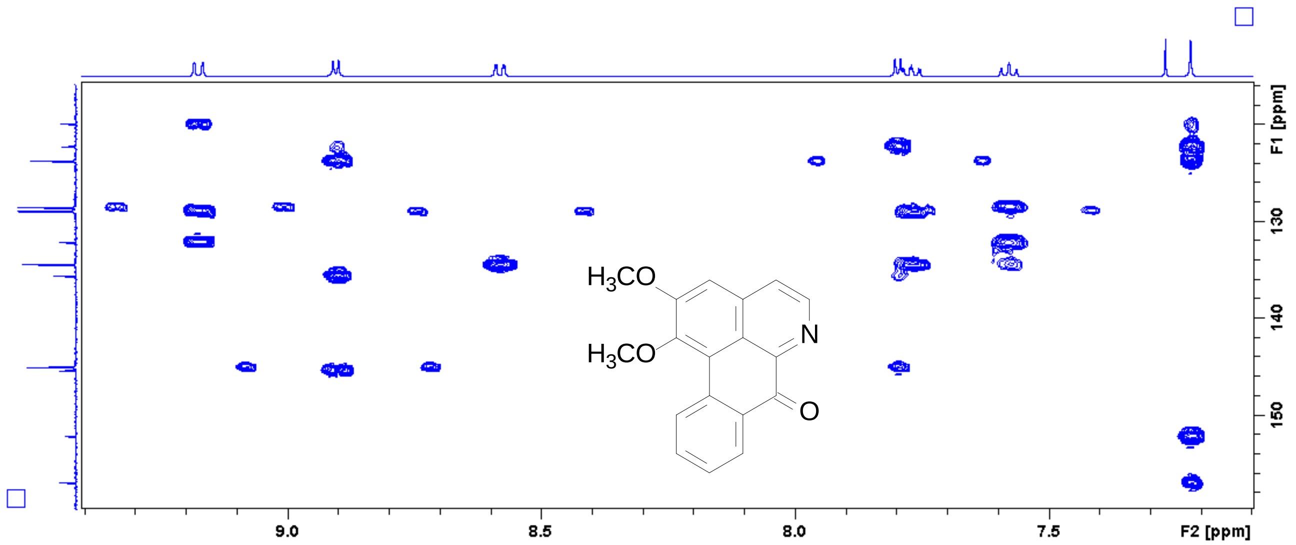

Figure S102. Expanded view 2 of the long-range ^{1}H–^{13}C HMBC correlation map of **lysicamine** (**11**) recorded in $CDCl_3$ at 500 MHz (^{1}H) and 125 MHz (^{13}C).

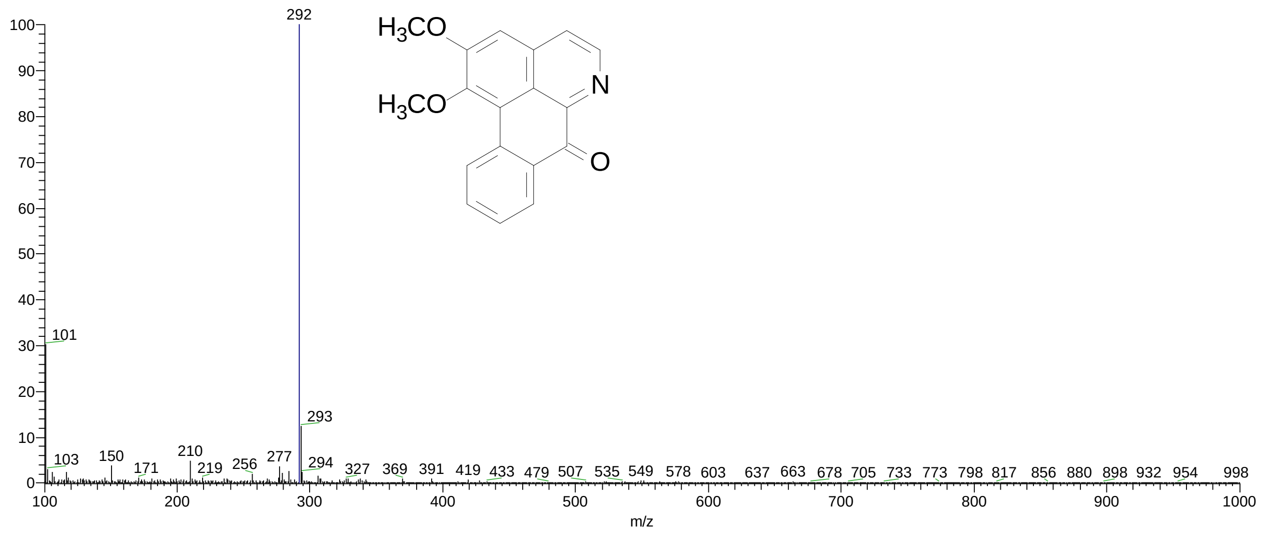

Figure S103. LR-APCI(+)-MS spectrum of **lysicamine** (**11**), showing the protonated molecular ion at *m/z* 292 $[M + H]^+$.

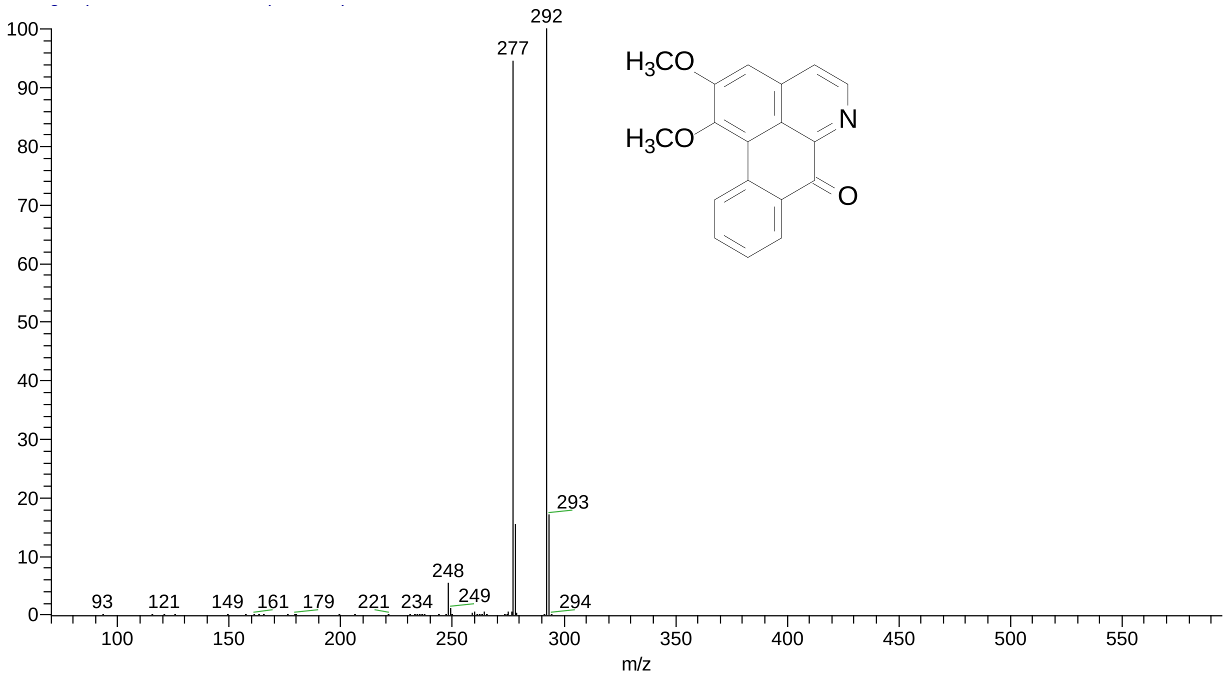

Figure S104. LR-APCI(+)-MS/MS spectrum of the protonated molecule of **lysicamine** (**11**) at *m/z* 292 $[M + H]^+$.

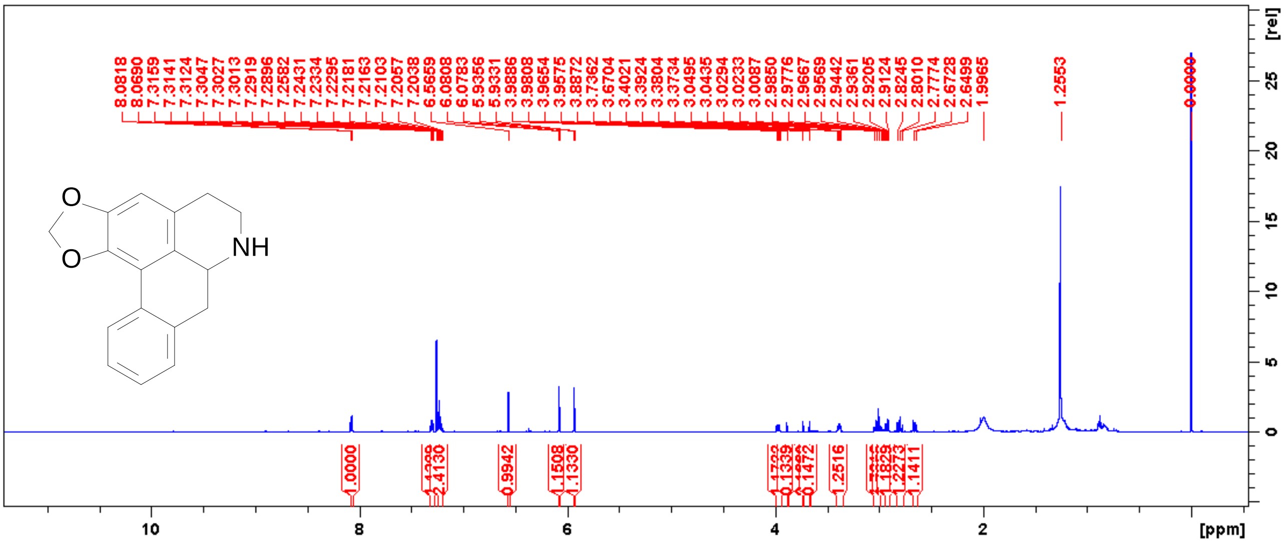

Figure S105. Full ^{1}H NMR spectrum of **anonaine** (**12**) recorded in $CDCl_3$ at 600 MHz.

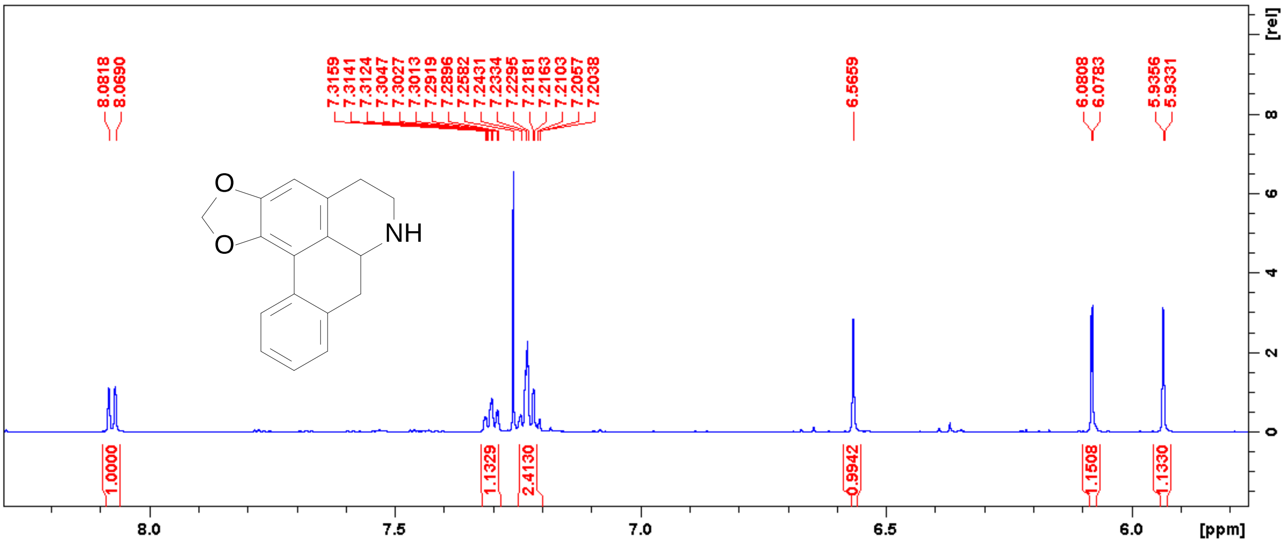

Figure S106. Expanded view 1 of the aromatic proton region in the ^{1}H NMR spectrum of **anonaine** (**12**) recorded in $CDCl_3$ at 600 MHz.

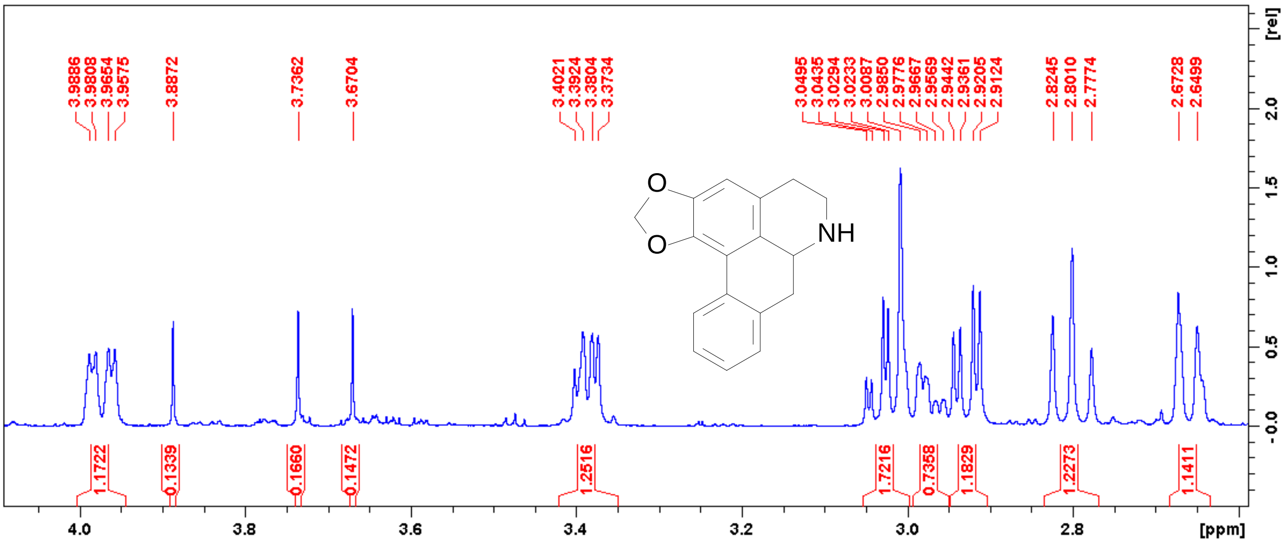

Figure S107. Expanded view 2 of the aliphatic proton region in the ^{1}H NMR spectrum **anonaine** (**12**) recorded in $CDCl_3$ at 600 MHz.

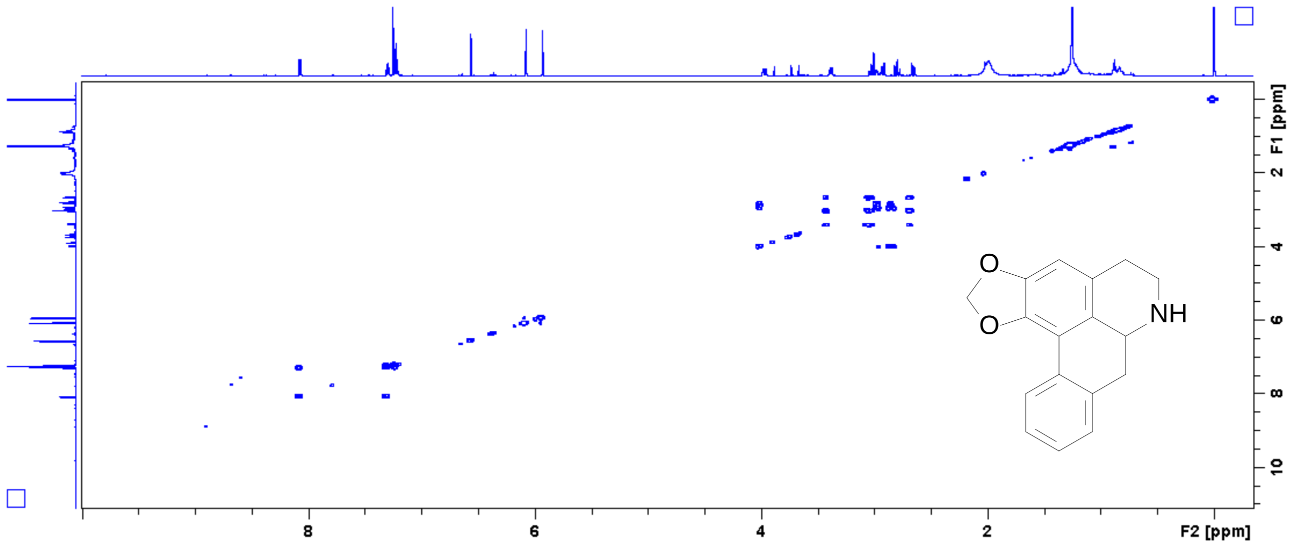

Figure S108. ^{1}H–^{1}H COSY correlation map of **anonaine** (**12**) recorded in $CDCl_3$ at 600 MHz.

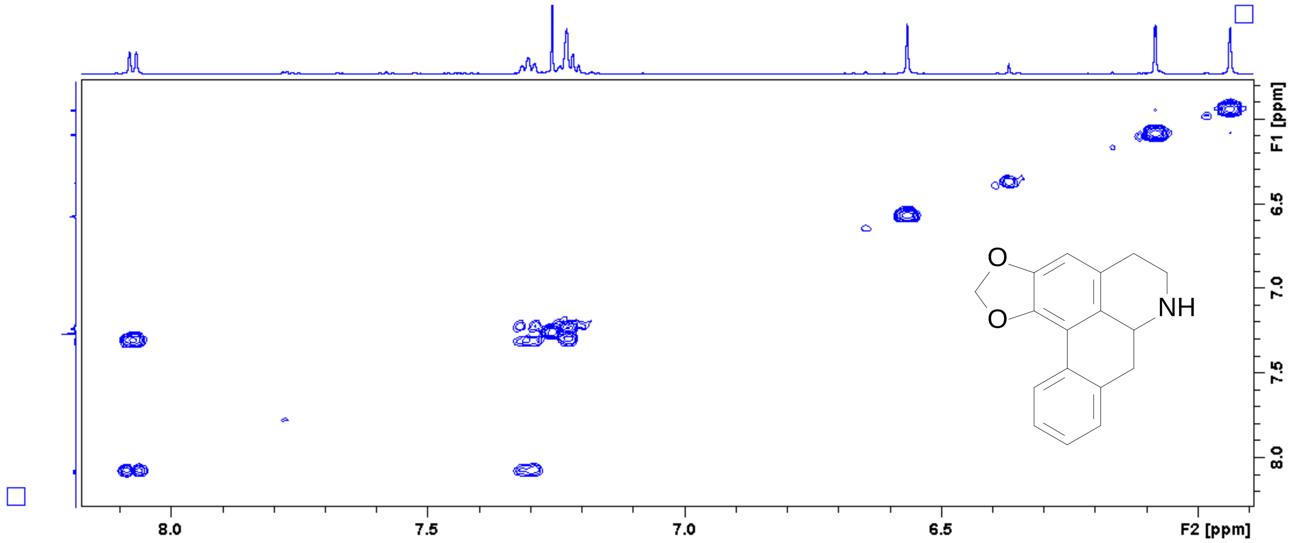

Figure S109. Expanded view 1 of the ^{1}H–^{1}H COSY correlation map of **anonaine** (**12**) recorded in $CDCl_3$ at 600 MHz.

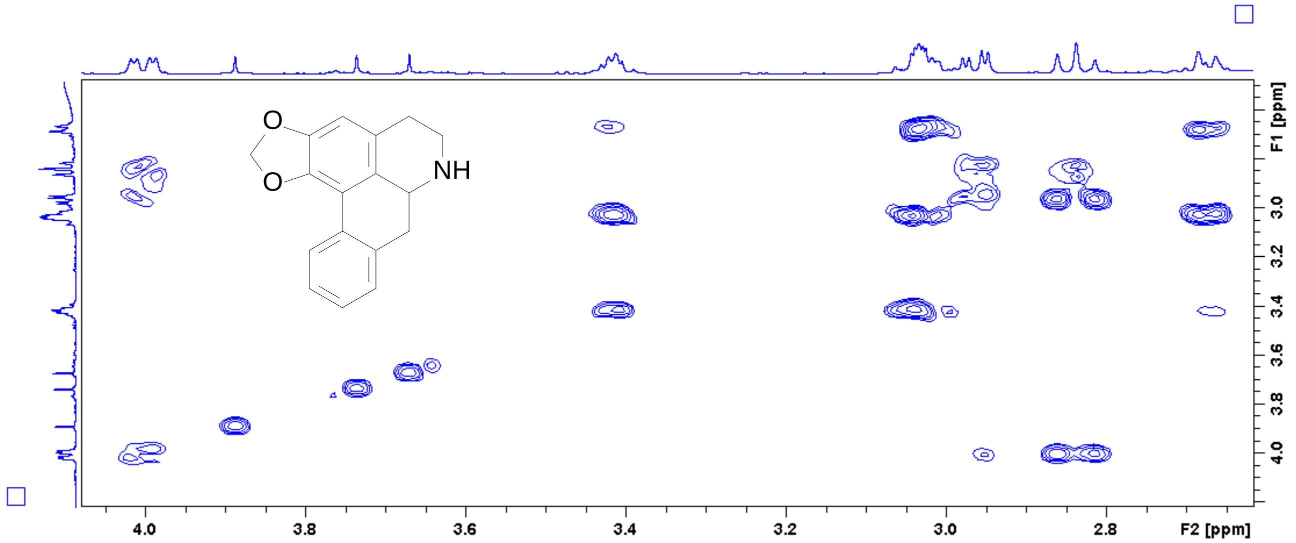

Figure S110. Expanded view 2 of the ^{1}H–^{1}H COSY correlation map of **anonaine** (**12**) recorded in $CDCl_3$ at 600 MHz.

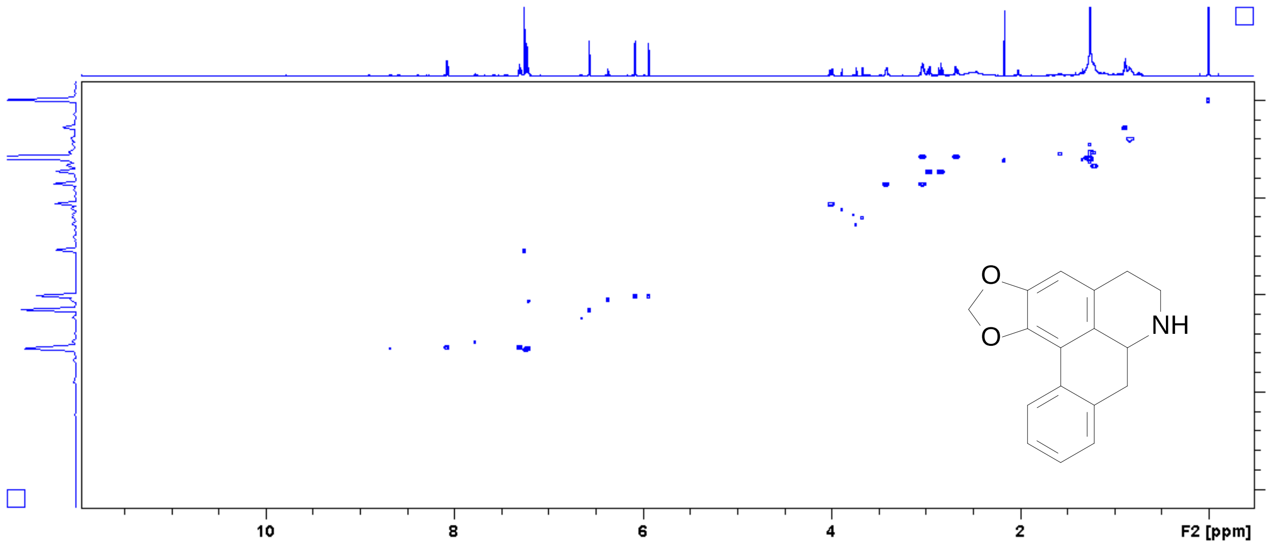

Figure S111. One-bond ^{1}H–^{13}C HSQC correlation map of **anonaine** (**12**) recorded in $CDCl_3$ with a drop of CD_3OD at 600 MHz (^{1}H) and 150 MHz (^{13}C).

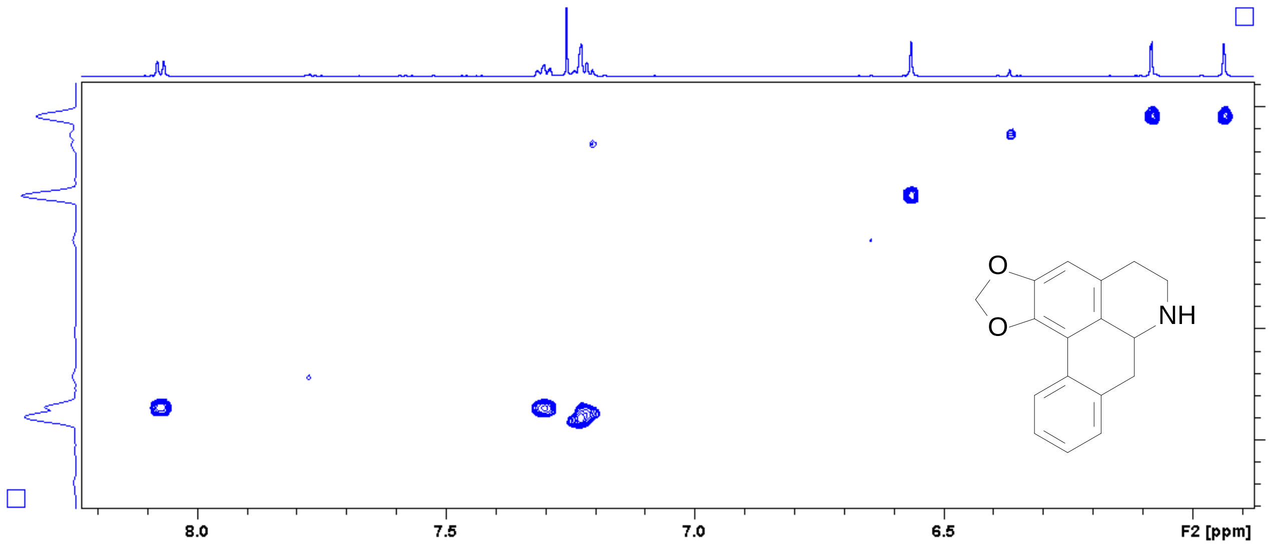

Figure S112. Expanded view 1 of the one-bond ^{1}H–^{13}C HSQC correlation map of **anonaine** (**12**) recorded in $CDCl_3$ at 600 MHz (^{1}H) and 150 MHz (^{13}C).

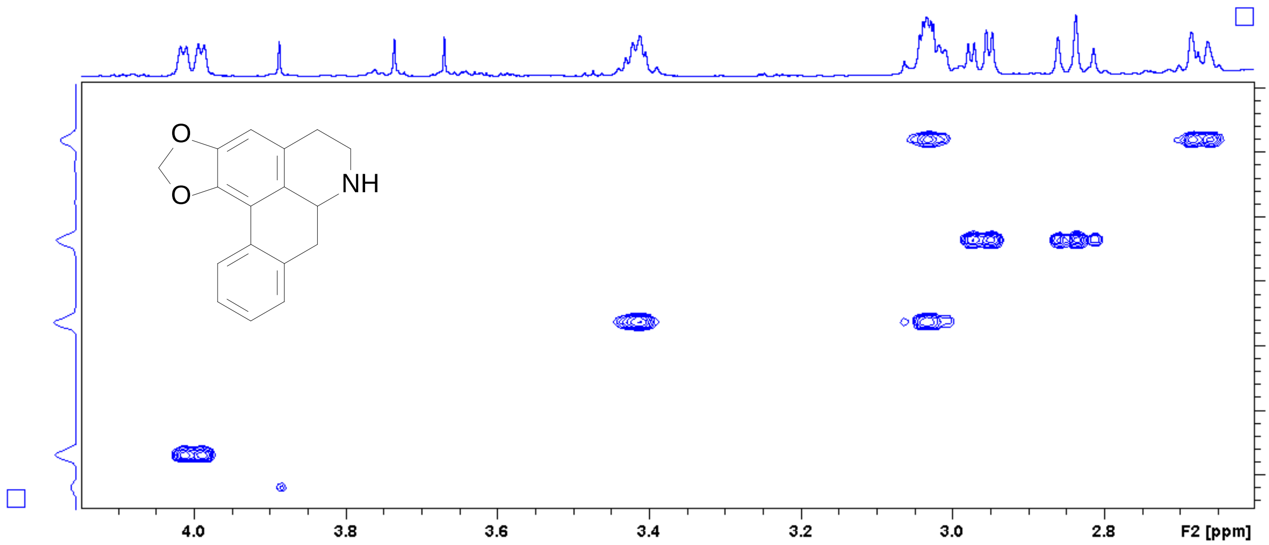

Figure S113. Expanded view 2 of the one-bond ^{1}H–^{13}C HSQC correlation of **anonaine** (**12**) recorded in $CDCl_3$ at 600 MHz (^{1}H) and 150 MHz (^{13}C).

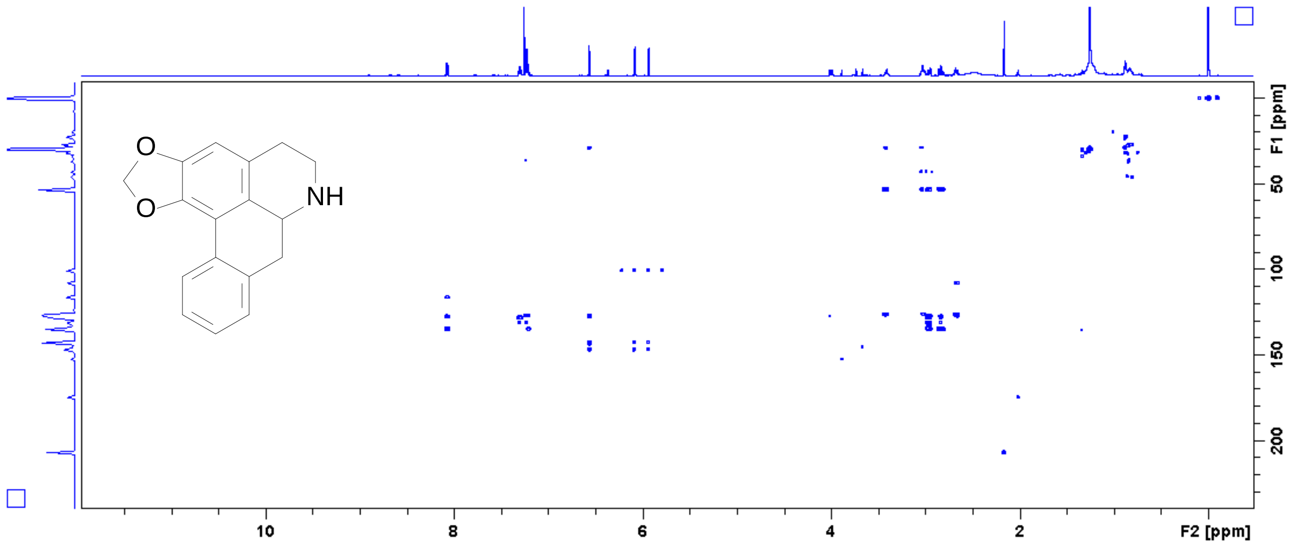

Figure S114. Long-range ^{1}H–^{13}C HMBC correlation map of **anonaine** (**12**) recorded in $CDCl_3$ at 600 MHz (^{1}H) and 150 MHz (^{13}C).

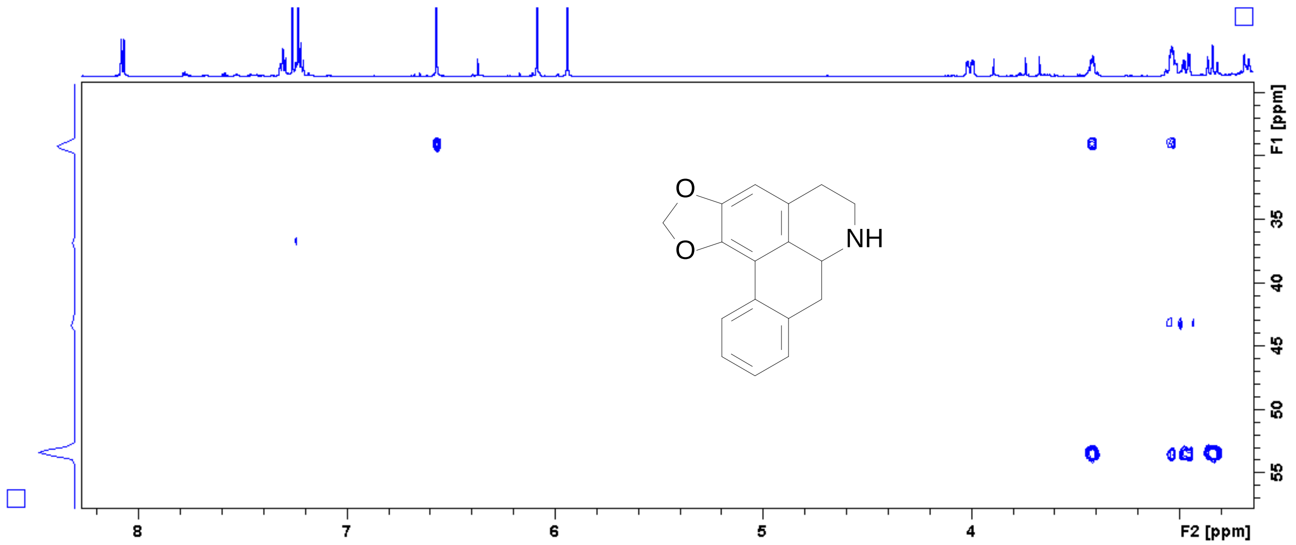

Figure S115. Expanded view 1 of the long-range ^{1}H–^{13}C HMBC correlation map of **anonaine** (**12**) recorded in $CDCl_3$ at 600 MHz (^{1}H) and 150 MHz (^{13}C).

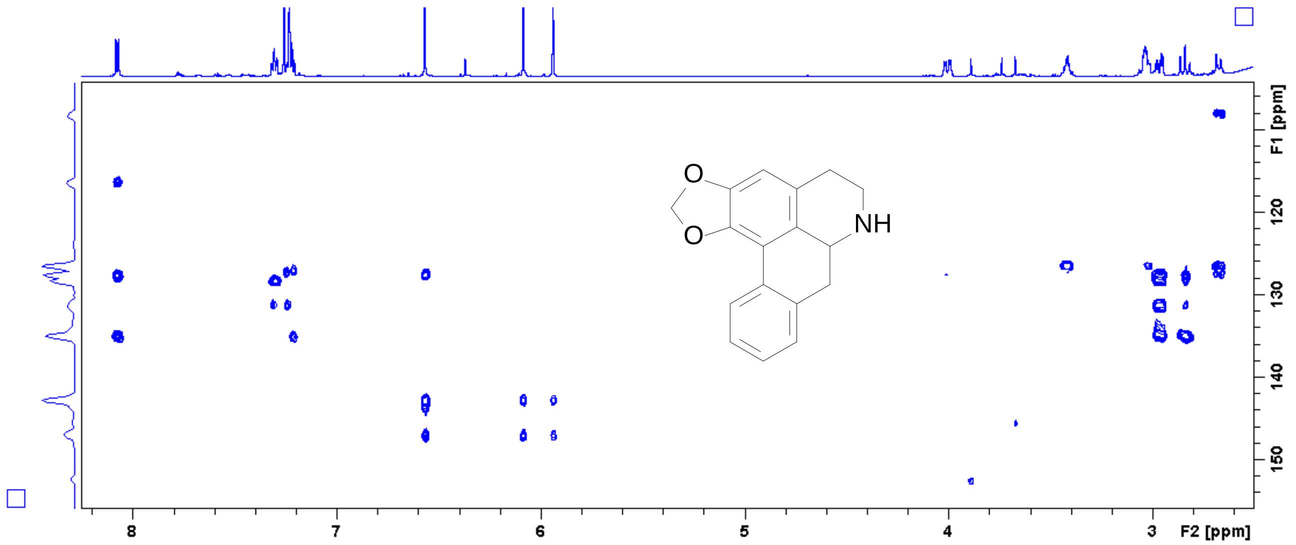

Figure S116. Expanded view 2 of the long-range ^{1}H–^{13}C HMBC correlation map of **anonaine** (**12**) recorded in $CDCl_3$ at 600 MHz (^{1}H) and 150 MHz (^{13}C).

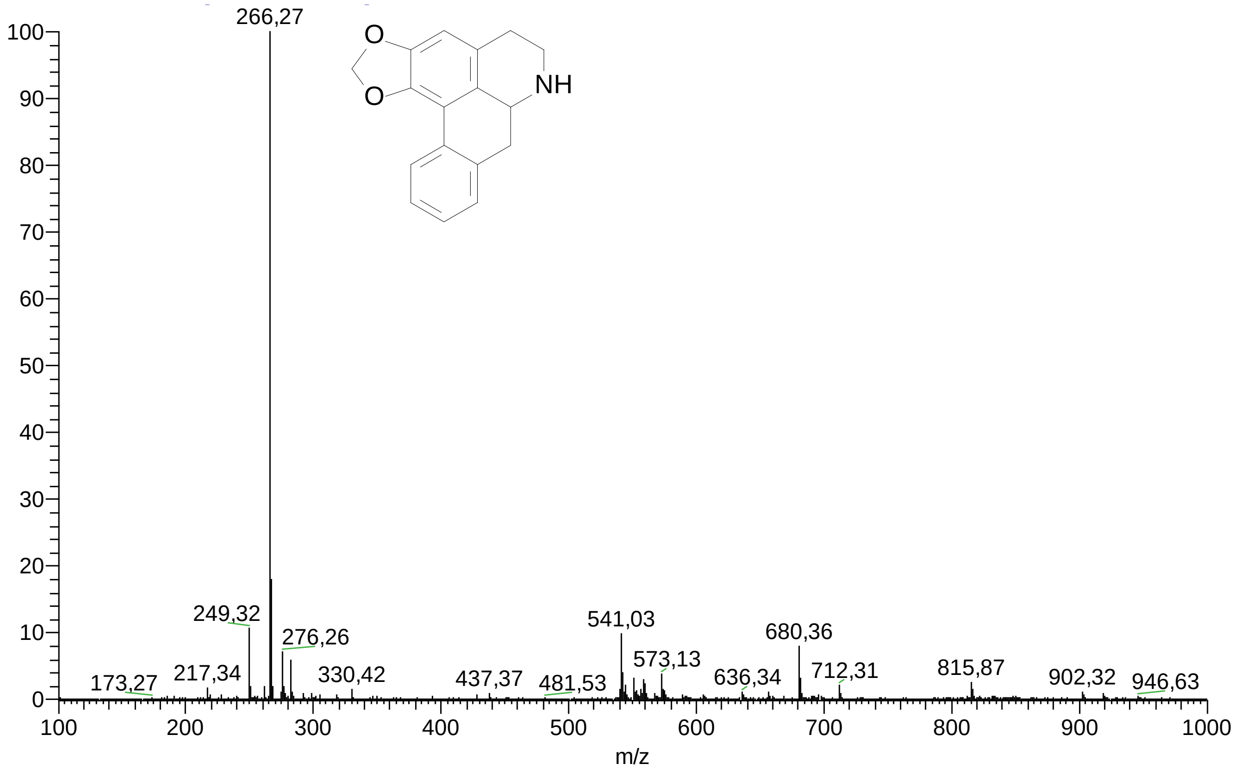

Figure S117. LR-APCI(+)-MS spectrum of **anonaine** (**12**), showing the protonated molecular ion at *m/z* 266 $[M + H]^+$.

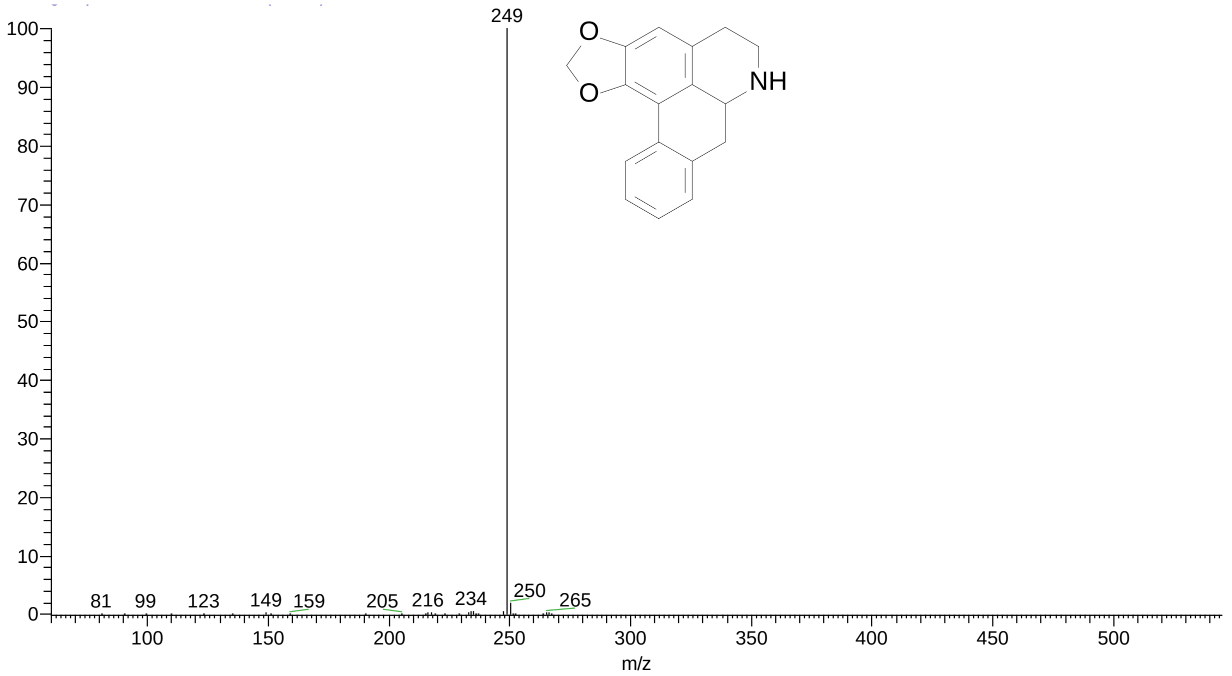

Figure S118. LR-APCI(+)-MS/MS spectrum of the protonated molecule of **anonaine** (**12**) at *m/z* 266 $[M + H]^+$.

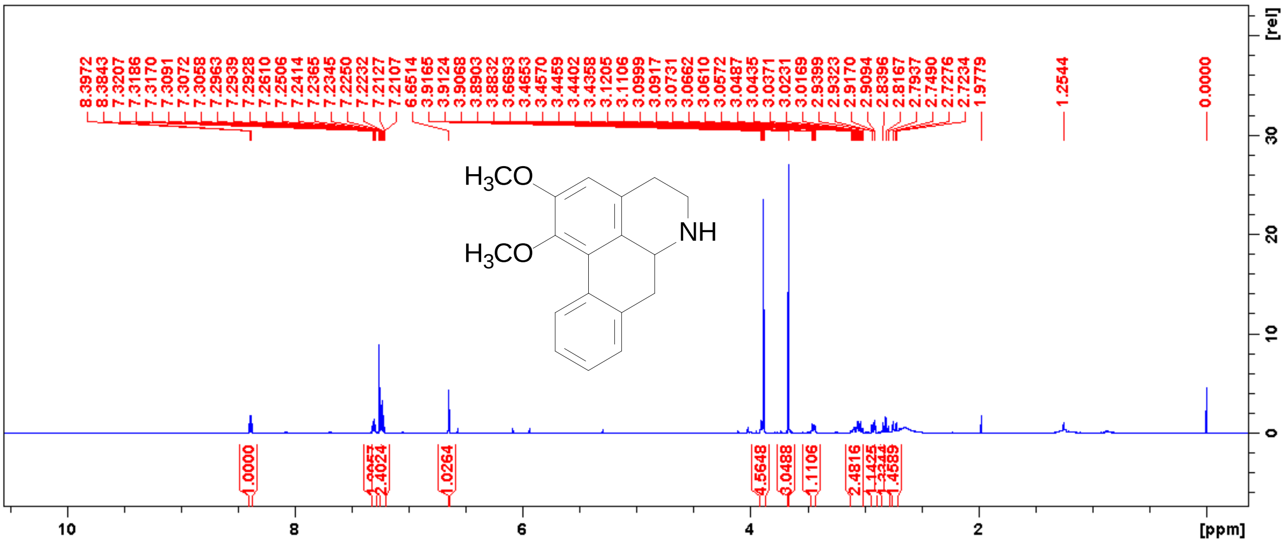

Figure S119. Full ^{1}H NMR spectrum of **nornuciferine** (**13**) recorded in $CDCl_3$ at 600 MHz.

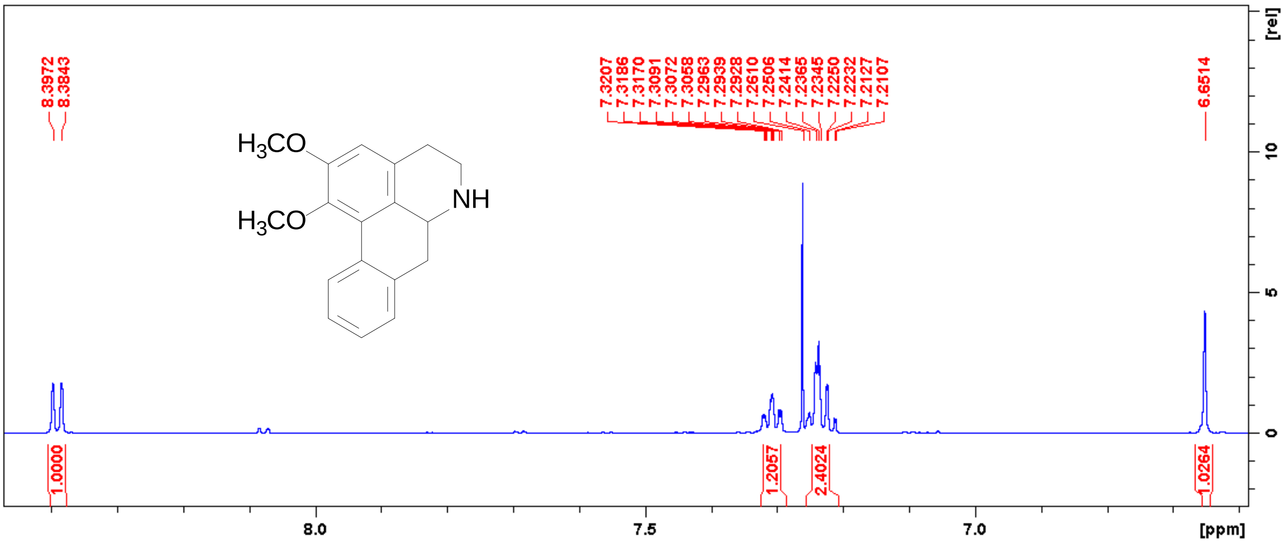

Figure S120. Expanded view 1 of the aromatic proton region in the ^{1}H NMR spectrum of **nornuciferine** (**13**) recorded in $CDCl_3$ at 600 MHz.

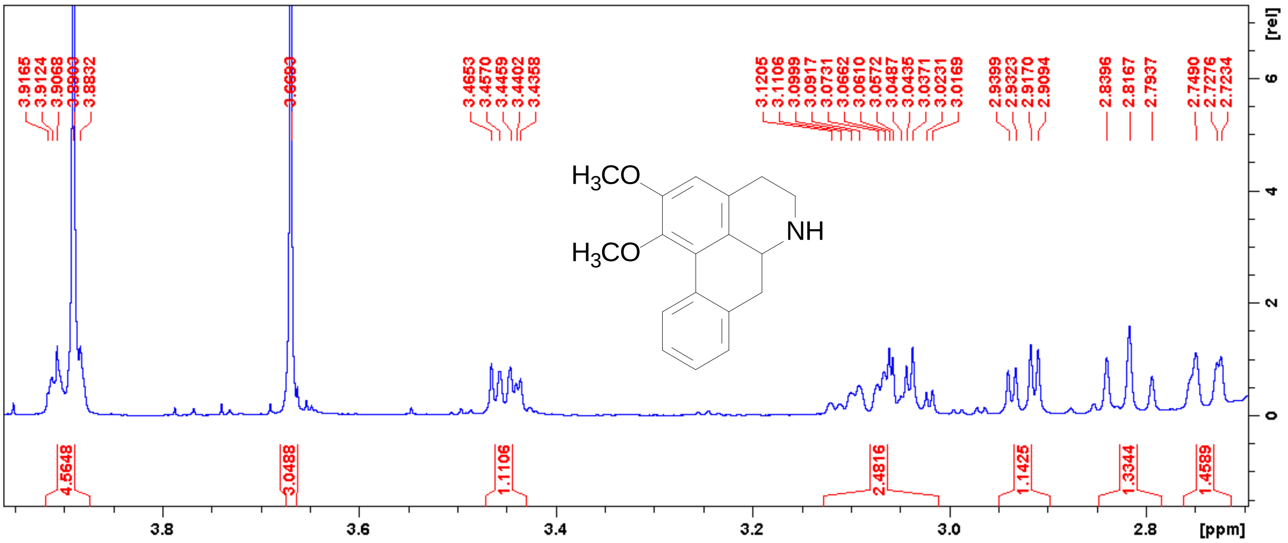

Figure S121. Expanded view 2 of the aliphatic proton region in the ^{1}H NMR spectrum **nornuciferine** (13) recorded in $CDCl_3$ at 600 MHz.

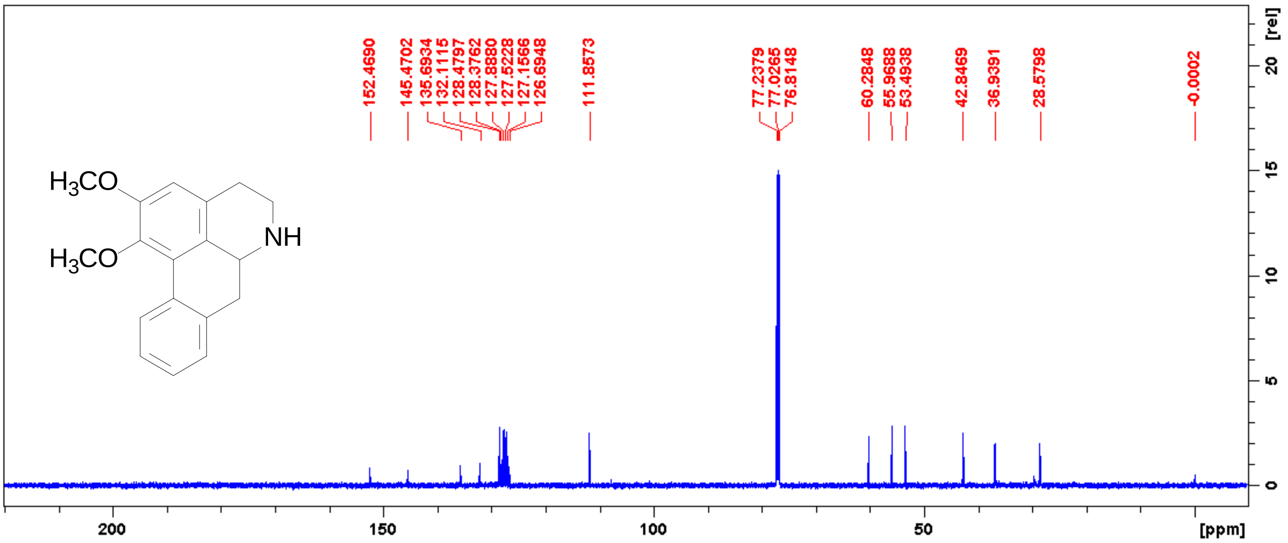

Figure S122. Full ^{13}C NMR spectrum of **nornuciferine** (**13**) recorded in $CDCl_3$ at 150 MHz.

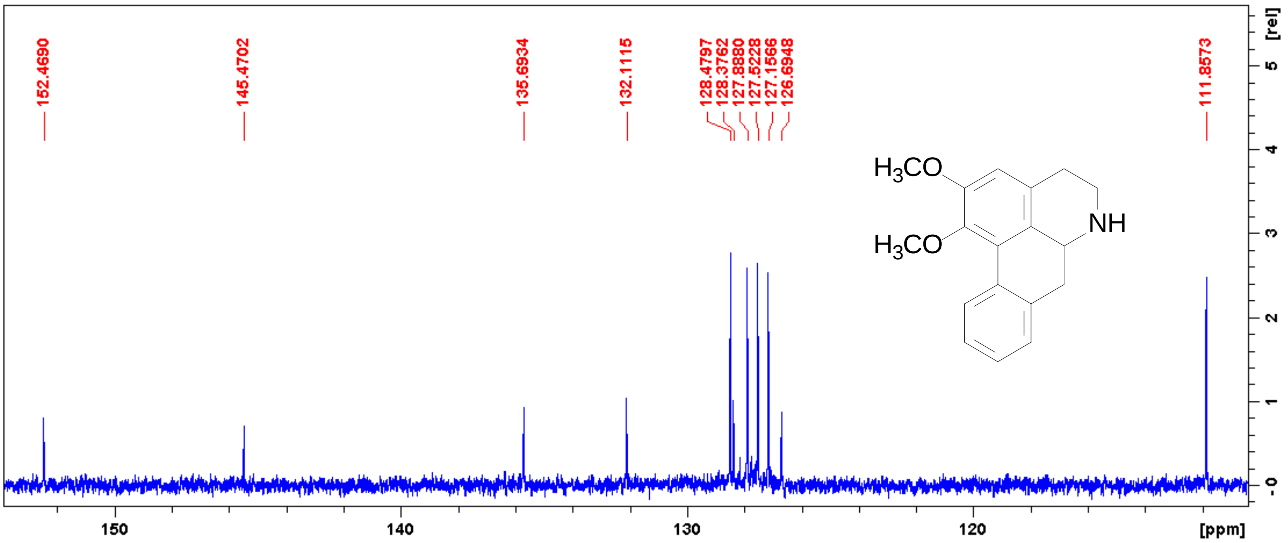

Figure S123. Expanded view of the carbon region (δ_C 153.7–110.4 ppm) in the ^{13}C NMR spectrum of **nornuciferine** (**13**) recorded in $CDCl_3$ at 150 MHz.

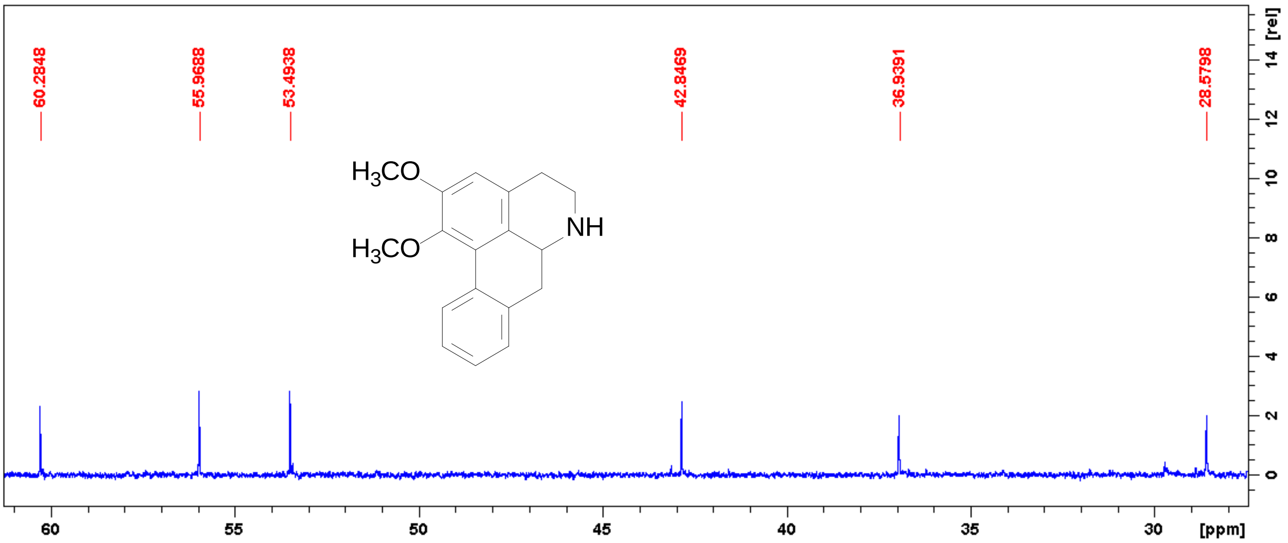

Figure S124. Expanded view of the carbon region (δ_C 61.1–27.5 ppm) in the ^{13}C NMR spectrum of **nornuciferine** (**13**) recorded in $CDCl_3$ at 150 MHz.

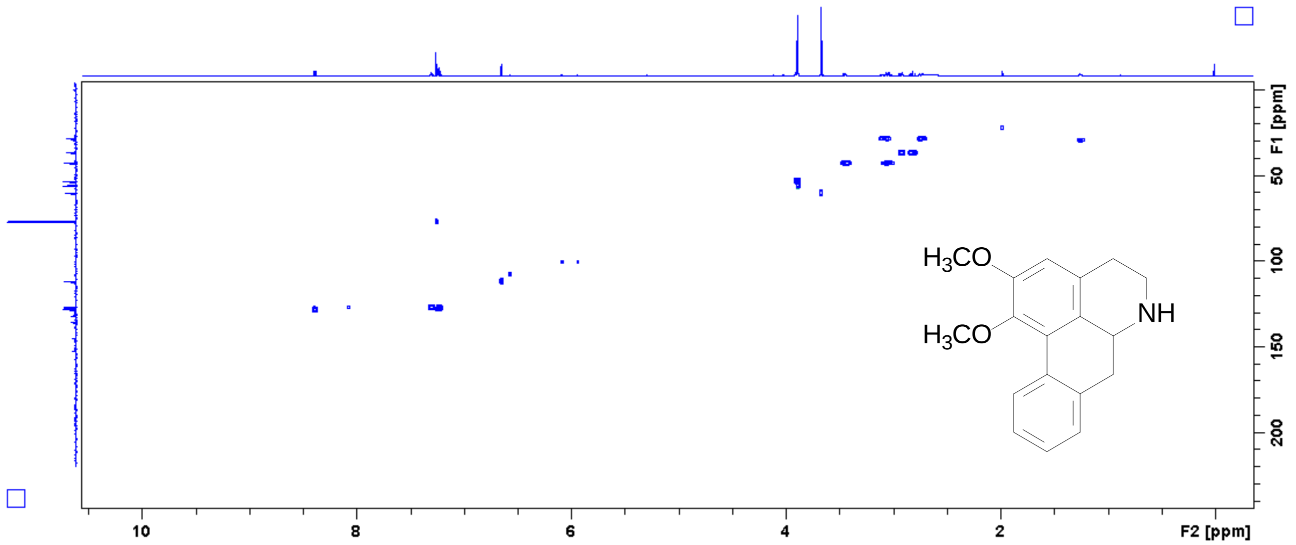

Figure S125. One-bond ^{1}H–^{13}C HSQC correlation map of **nornuciferine** (**13**) recorded in $CDCl_3$ at 600 MHz (^{1}H) and 150 MHz (^{13}C).

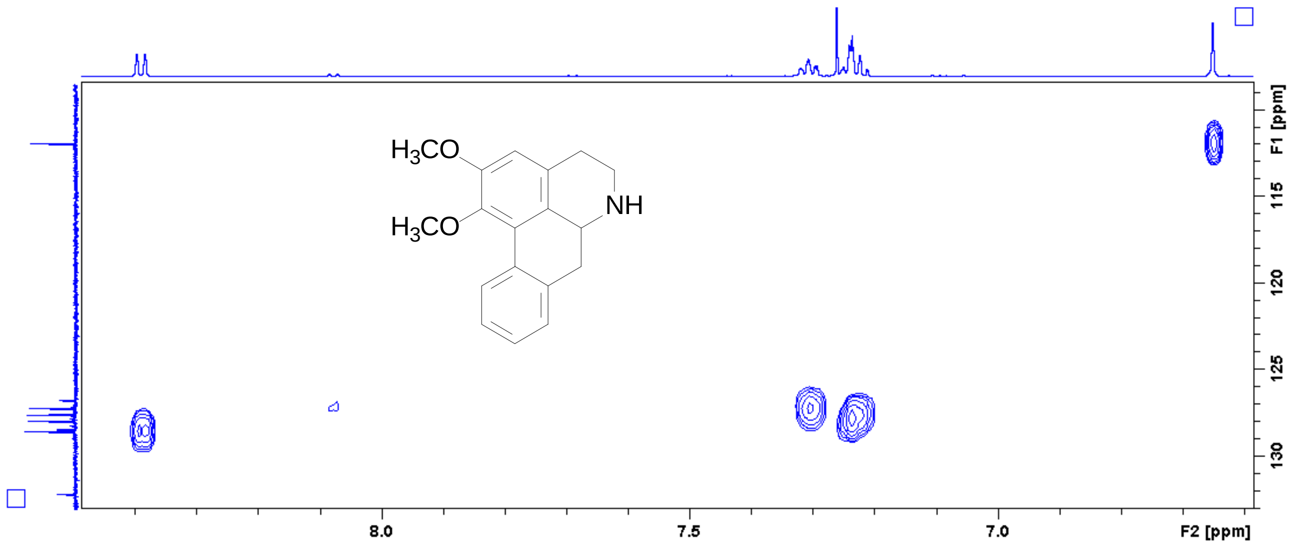

Figure S126. Expanded view 1 of the one-bond ^{1}H–^{13}C HSQC correlation map of **nornuciferine** (**13**) recorded in $CDCl_3$ at 600 MHz (^{1}H) and 150 MHz (^{13}C).

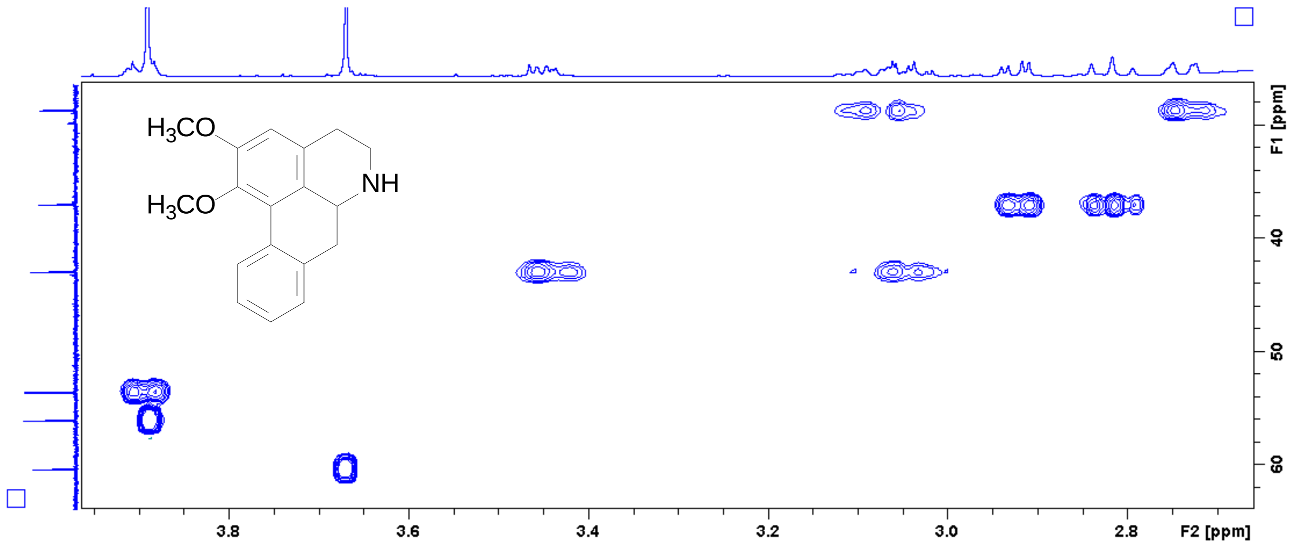

Figure S127. Expanded view 2 of the one-bond ^{1}H–^{13}C HSQC correlation of **nornuciferine** (**13**) recorded in $CDCl_3$ at 600 MHz (^{1}H) and 150 MHz (^{13}C).

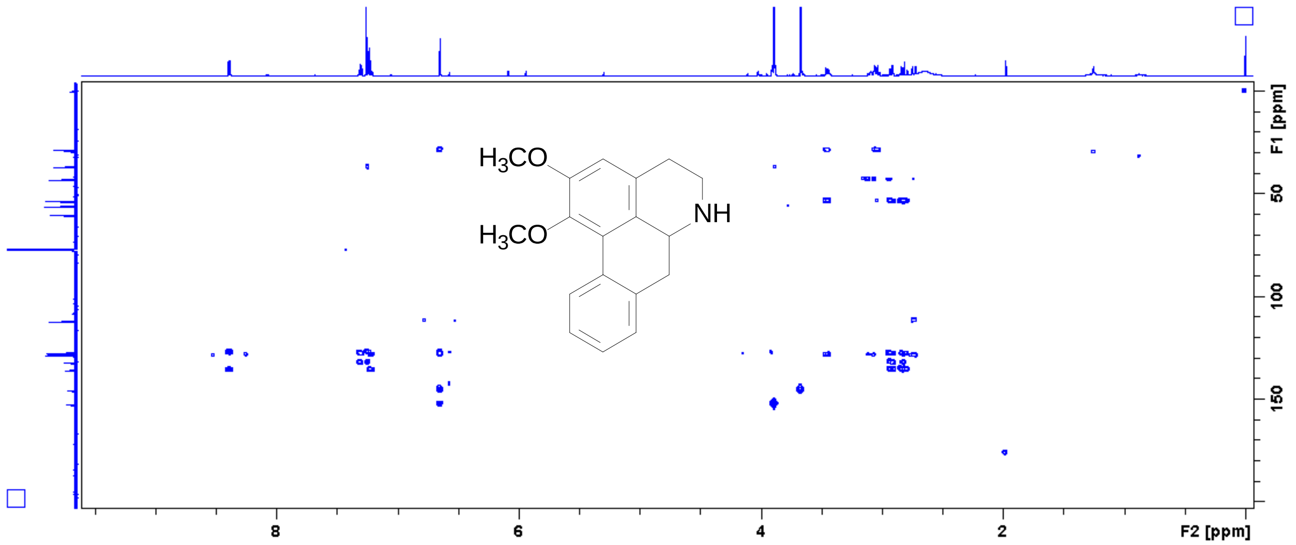

Figure S128. Long-range ^{1}H–^{13}C HMBC correlation map of **nornuciferine** (**13**) recorded in $CDCl_3$ at 600 MHz (^{1}H) and 150 MHz (^{13}C).

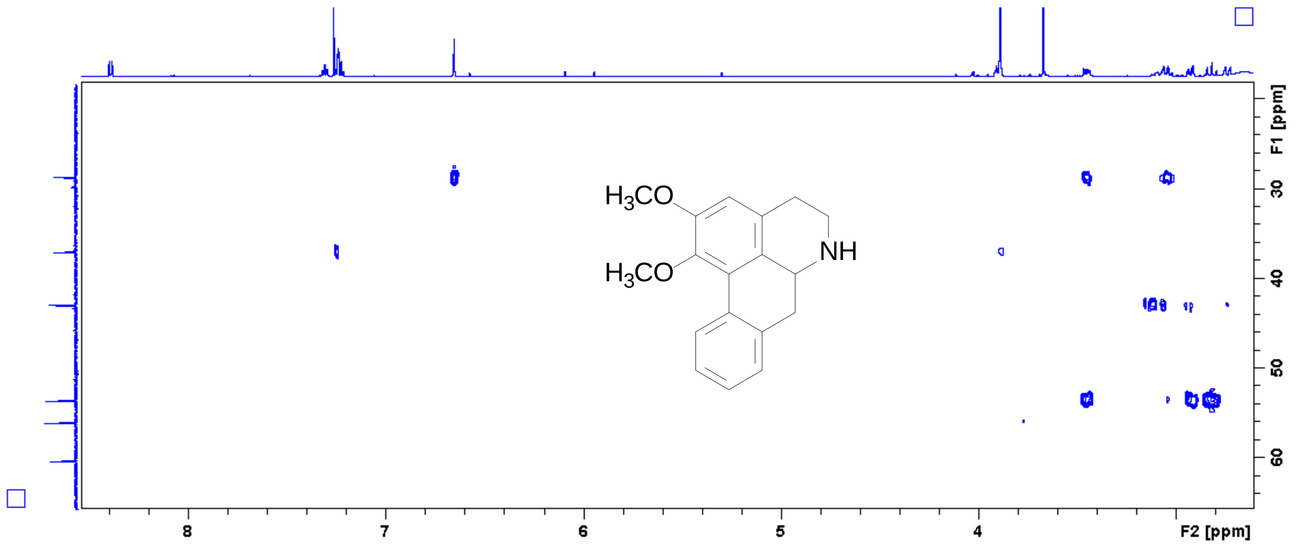

Figure S129. Expanded view 1 of the long-range ^{1}H–^{13}C HMBC correlation map of **nornuciferine** (**13**) recorded in $CDCl_3$ at 600 MHz (^{1}H) and 150 MHz (^{13}C).

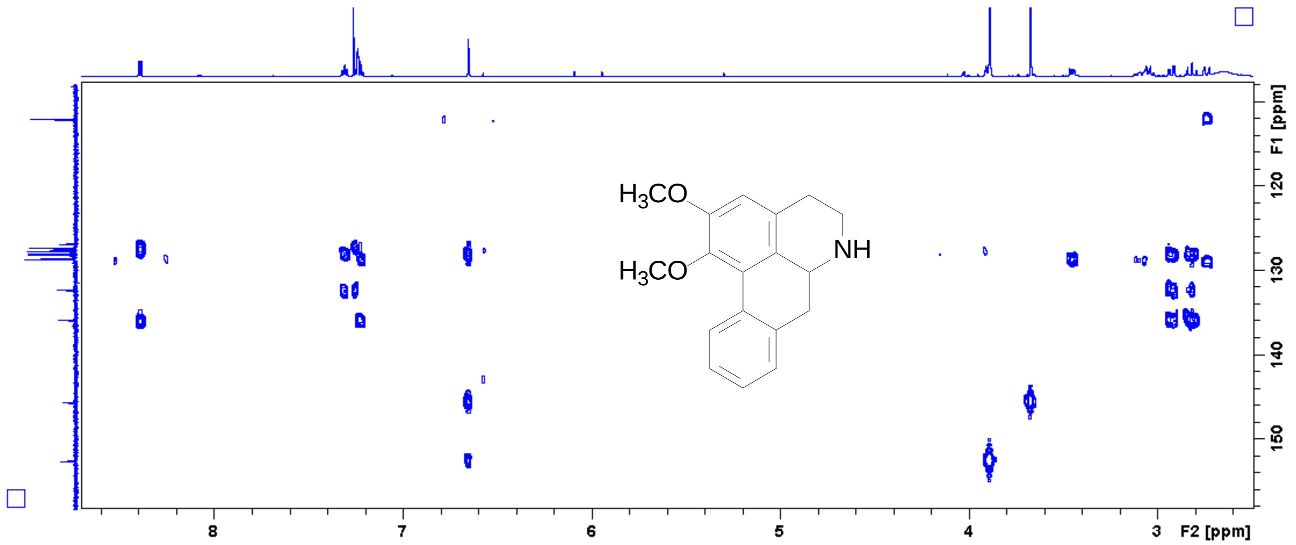

Figure S130. Expanded view 2 of the long-range ^{1}H–^{13}C HMBC correlation map of **nornuciferine** (**13**) recorded in $CDCl_3$ at 600 MHz (^{1}H) and 150 MHz (^{13}C).

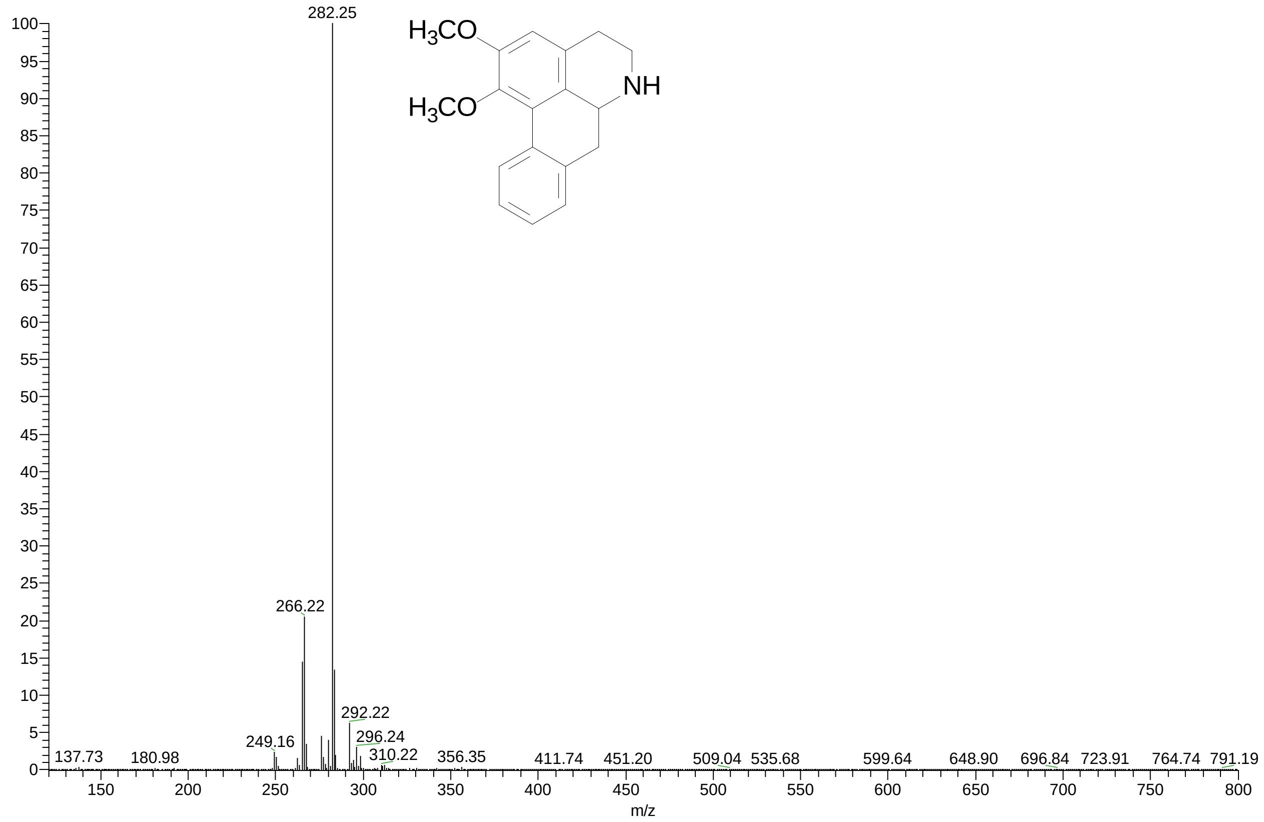

Figure S131. LR-APCI(+)-MS spectrum of **nornuciferine** (**13**), showing the protonated molecular ion at *m/z* 282 $[M + H]^+$.

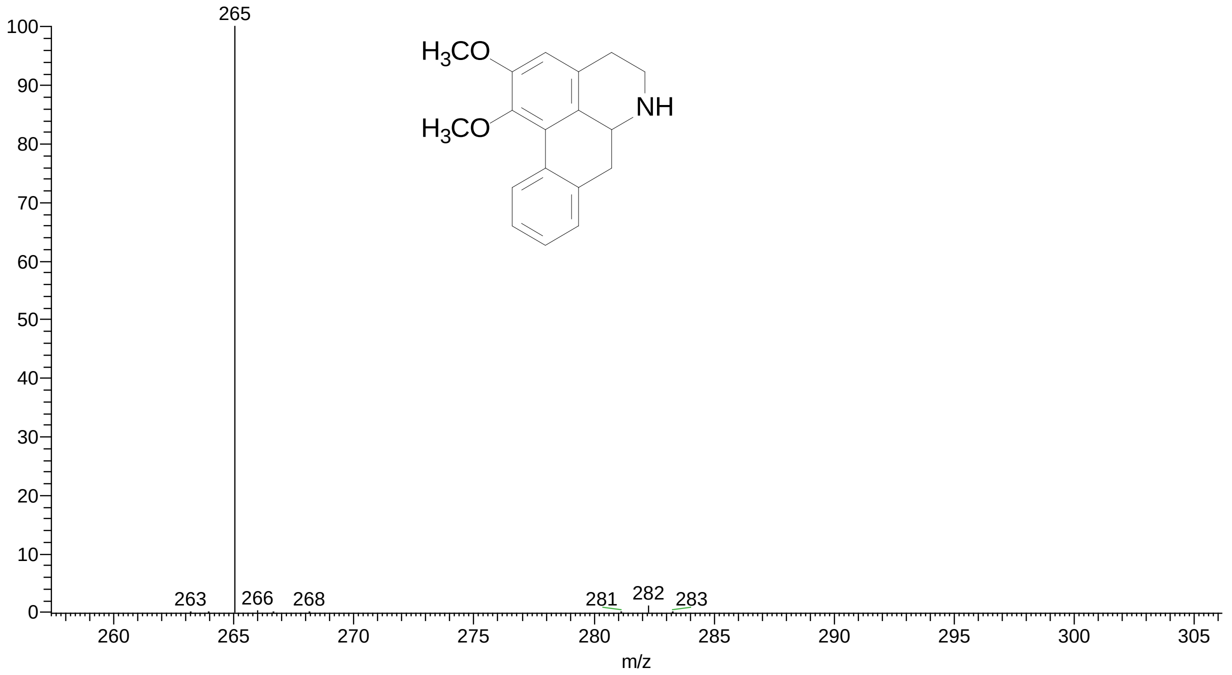

Figure S132. LR-APCI(+)-MS/MS spectrum of the protonated molecule of **nornuciferine** (**13**) at *m/z* 282 $[M + H]^+$.

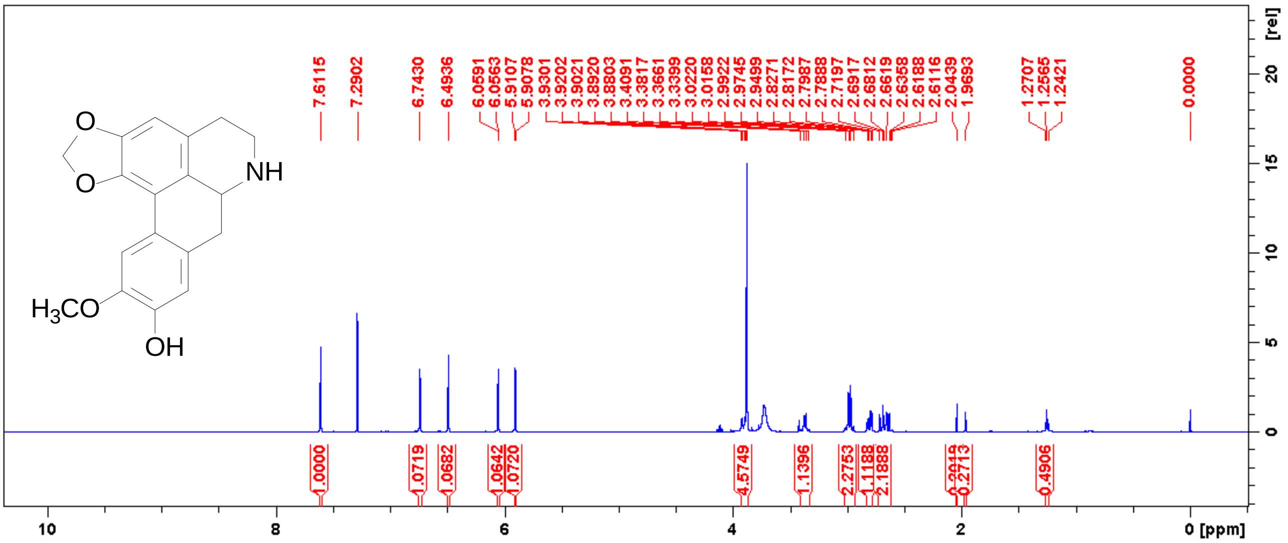

Figure S133. Full ^{1}H NMR spectrum of **actinodaphnine** (**14**) recorded in $CDCl_3$ with a drop of CD_3OD at 500 MHz.

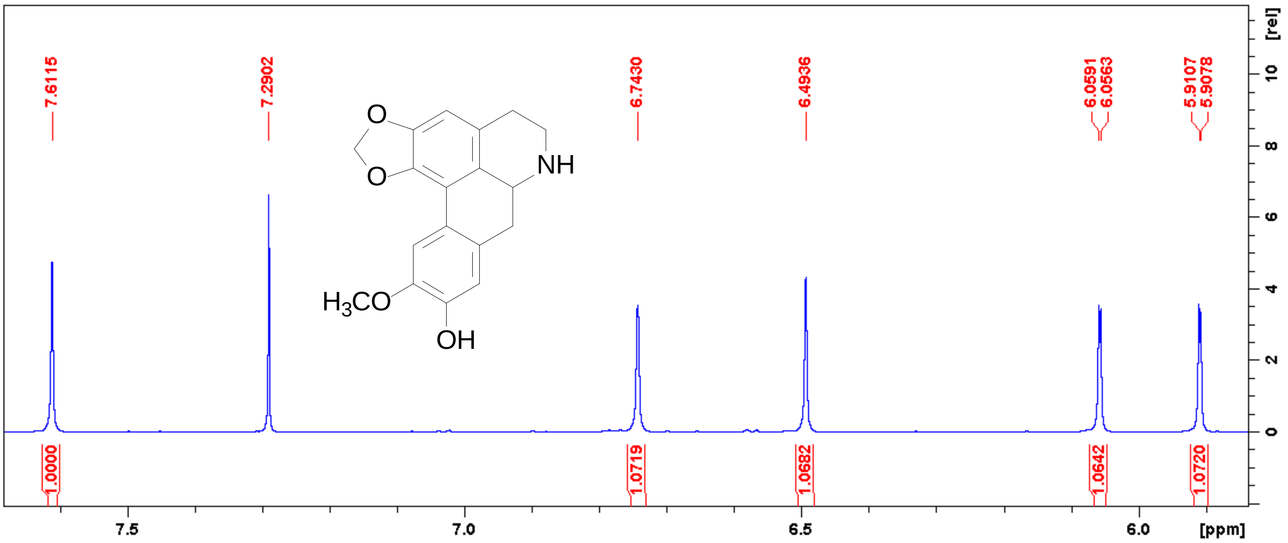

Figure S134. Expanded view 1 of the aromatic proton region in the ^{1}H NMR spectrum of **actinodaphnine** (**14**) recorded in $CDCl_3$ with a drop of CD_3OD at 500 MHz.

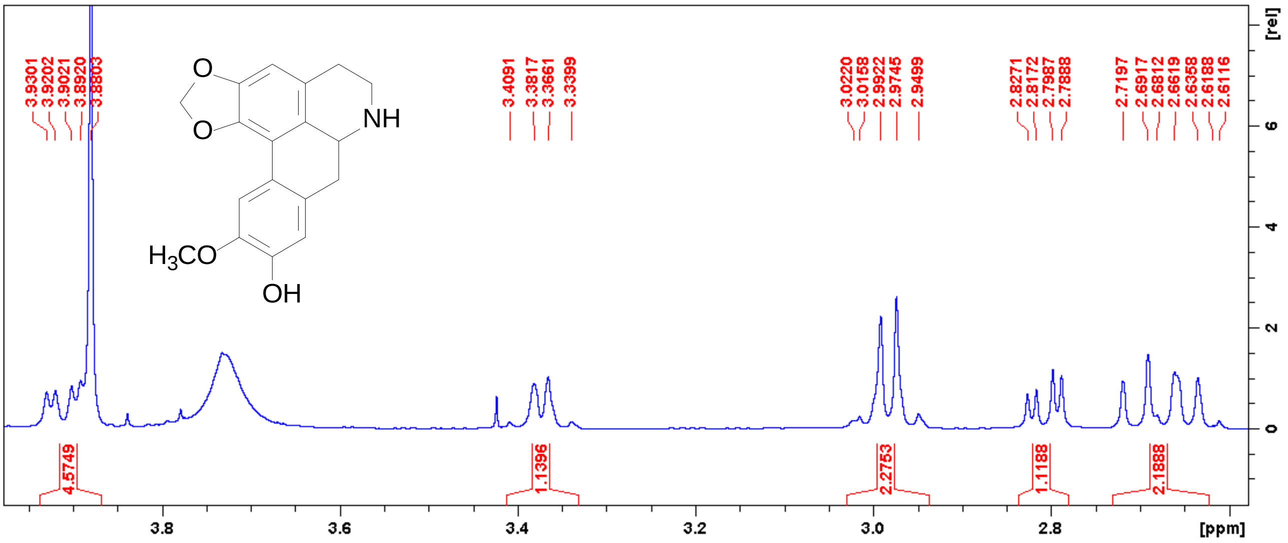

Figure S135. Expanded view 2 of the aliphatic proton region in the ^{1}H NMR spectrum **actinodaphnine** (**14**) recorded in $CDCl_3$ with a drop of CD_3OD at 500 MHz.

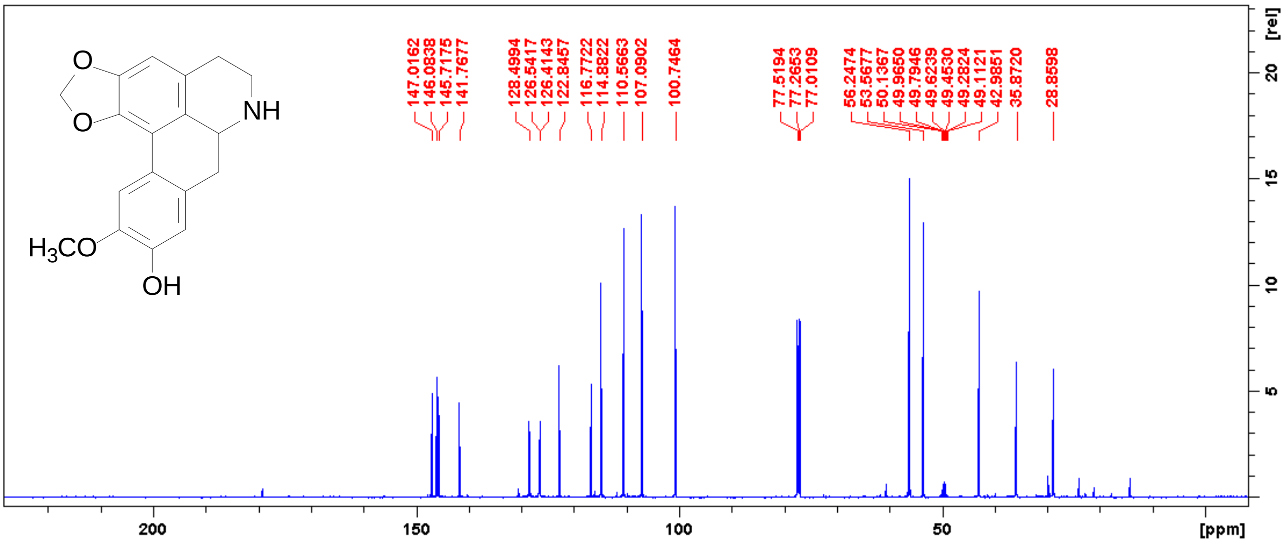

Figure S136. Full ^{13}C NMR spectrum of **actinodaphnine** (**14**) recorded in $CDCl_3$ with a drop of CD_3OD at 125 MHz.

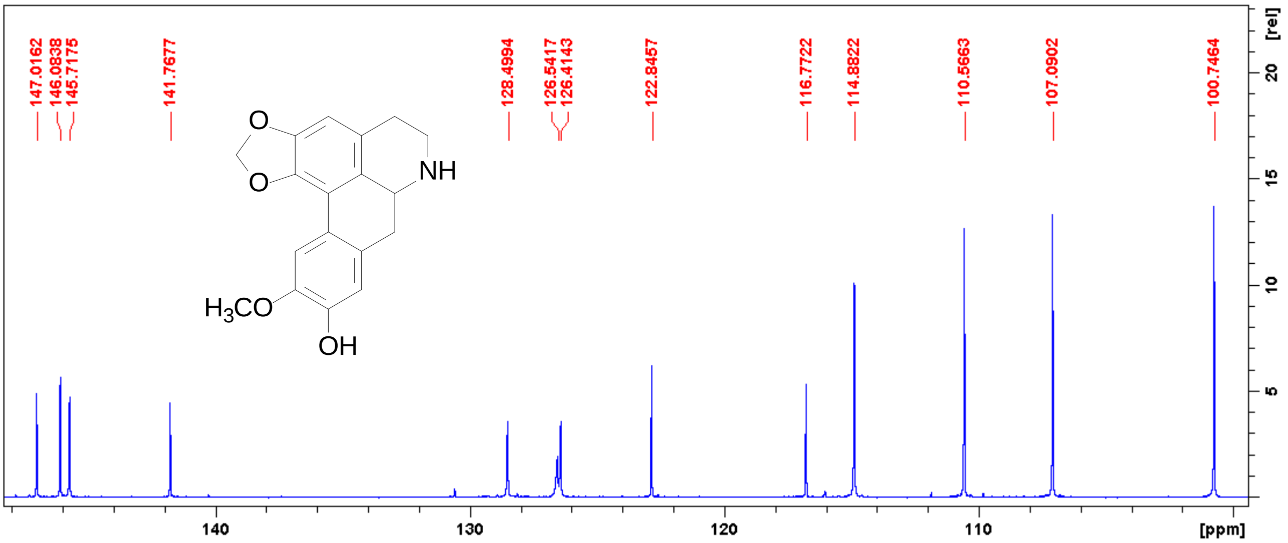

Figure S137. Expanded view of the carbon region (δ_C 148.2–99.5 ppm) in the ^{13}C NMR spectrum of **actinodaphnine** (**14**) recorded in $CDCl_3$ with a drop of CD_3OD at 125 MHz.

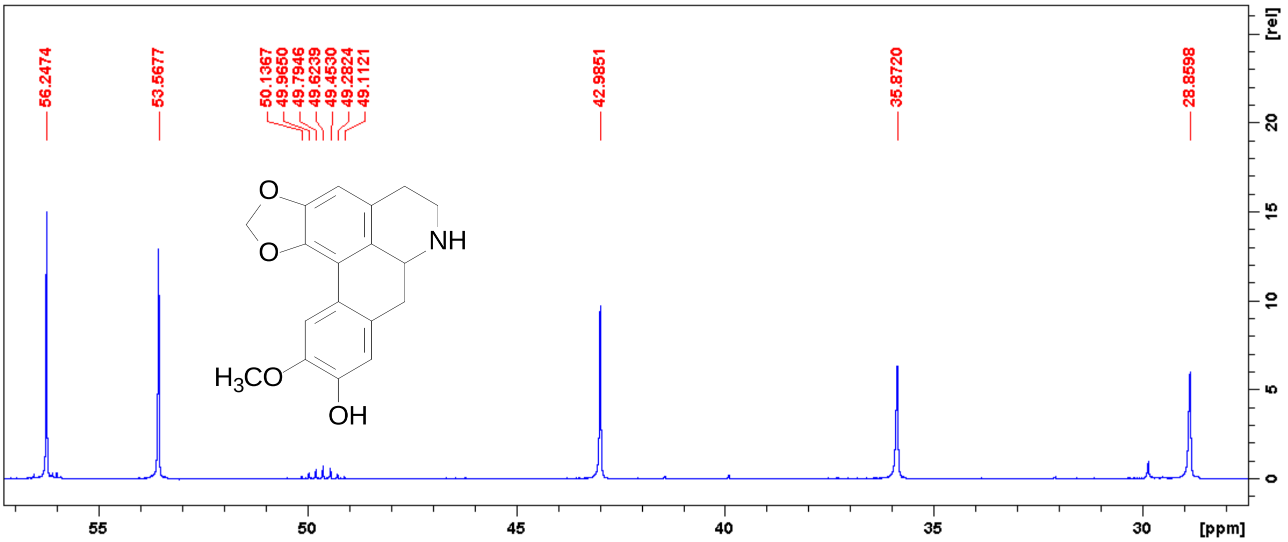

Figure S138. Expanded view of the carbon region (δ_C 57.1–27.5 ppm) in the ^{13}C NMR spectrum of **actinodaphnine** (**14**) recorded in $CDCl_3$ with a drop of CD_3OD at 125 MHz.

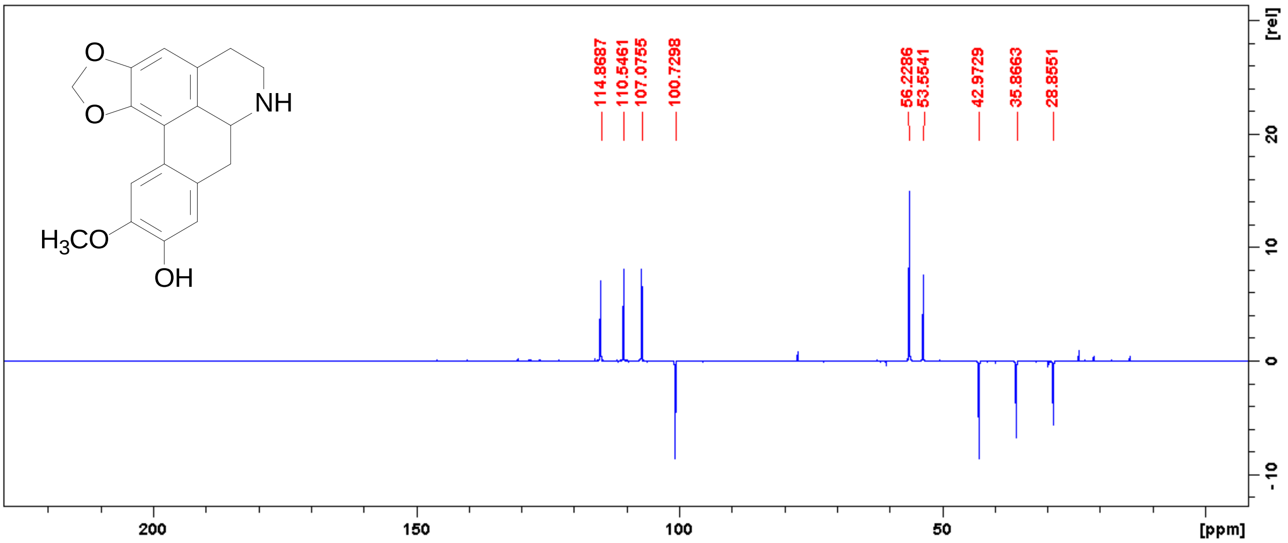

Figure S139. Full DEPT-135 ^{13}C NMR spectrum of **actinodaphnine** (**14**) recorded in $CDCl_3$ with a drop of CD_3OD at 125 MHz.

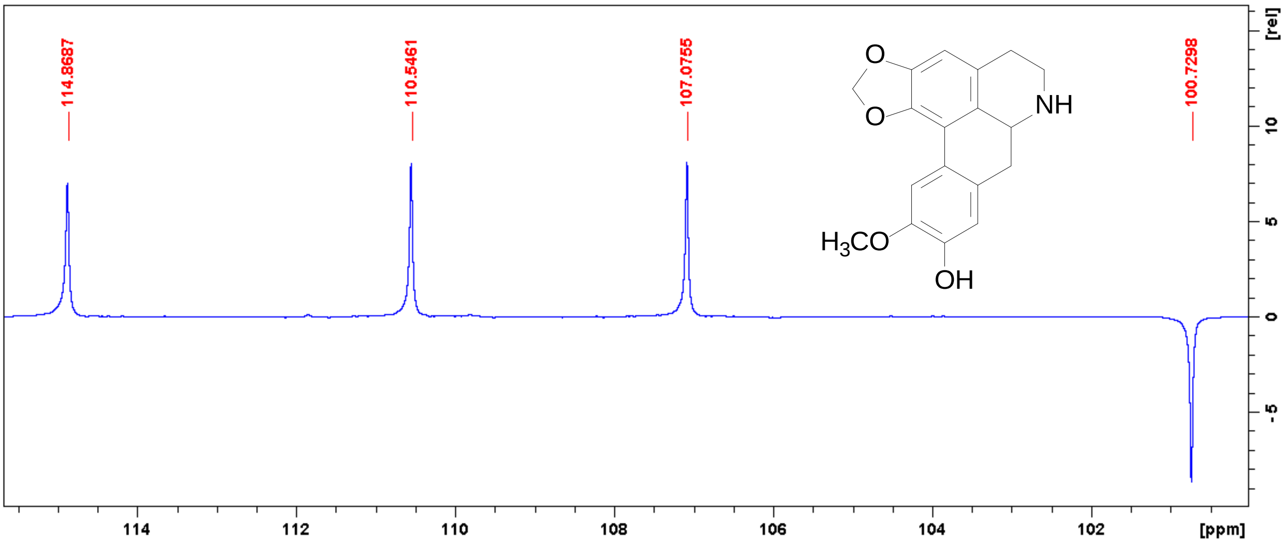

Figure S140. Expanded view of the carbon region (δ_C 115.6–100.0 ppm) in the DEPT-135 ^{13}C NMR spectrum of **actinodaphnine** (**14**) recorded in $CDCl_3$ with a drop of CD_3OD at 125 MHz.

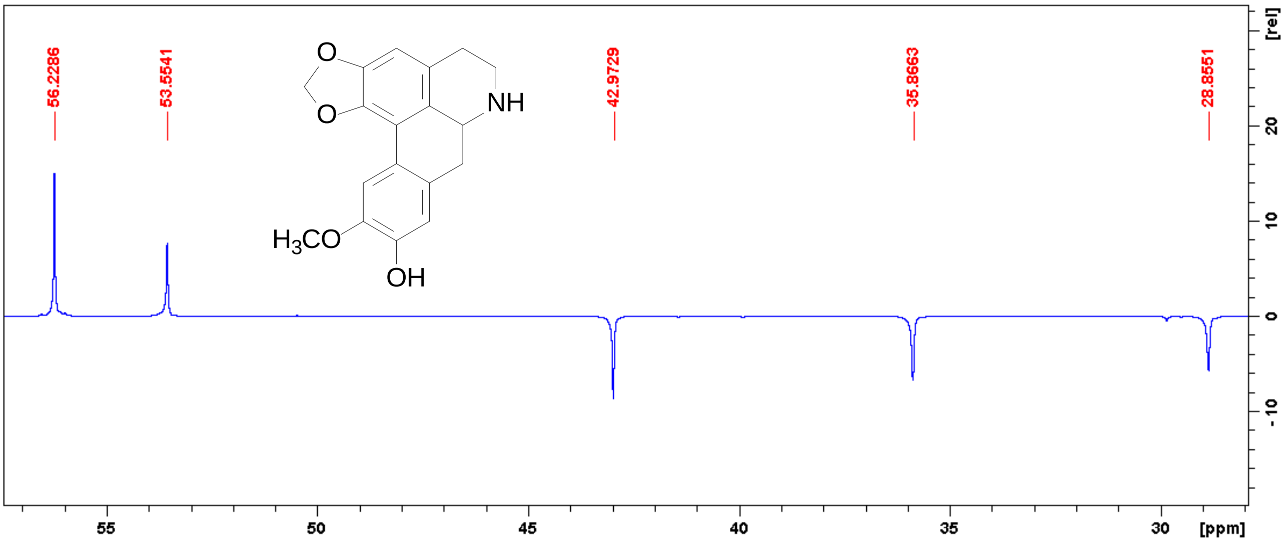

Figure S141. Expanded view of the carbon region (δ_C 57.2–28.4 ppm) in the DEPT-135 ^{13}C NMR spectrum of **actinodaphnine** (**14**) recorded in $CDCl_3$ with a drop of CD_3OD at 125 MHz.

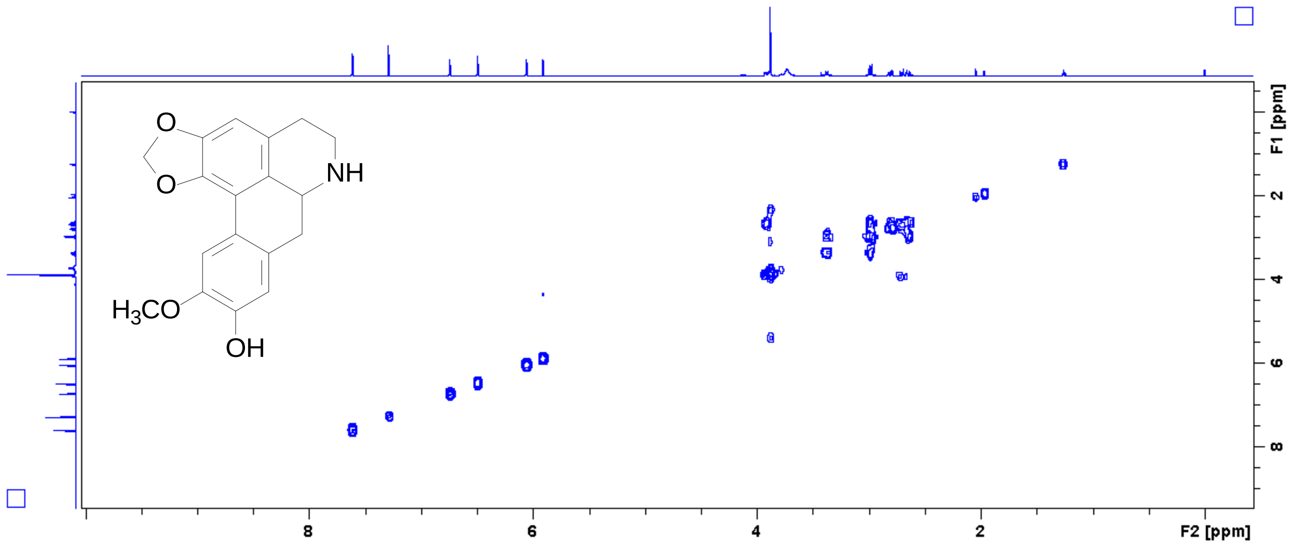

Figure S142. ^{1}H–^{1}H COSY correlation map of **actinodaphnine** (**14**) recorded in $CDCl_3$ with a drop of CD_3OD at 500 MHz.

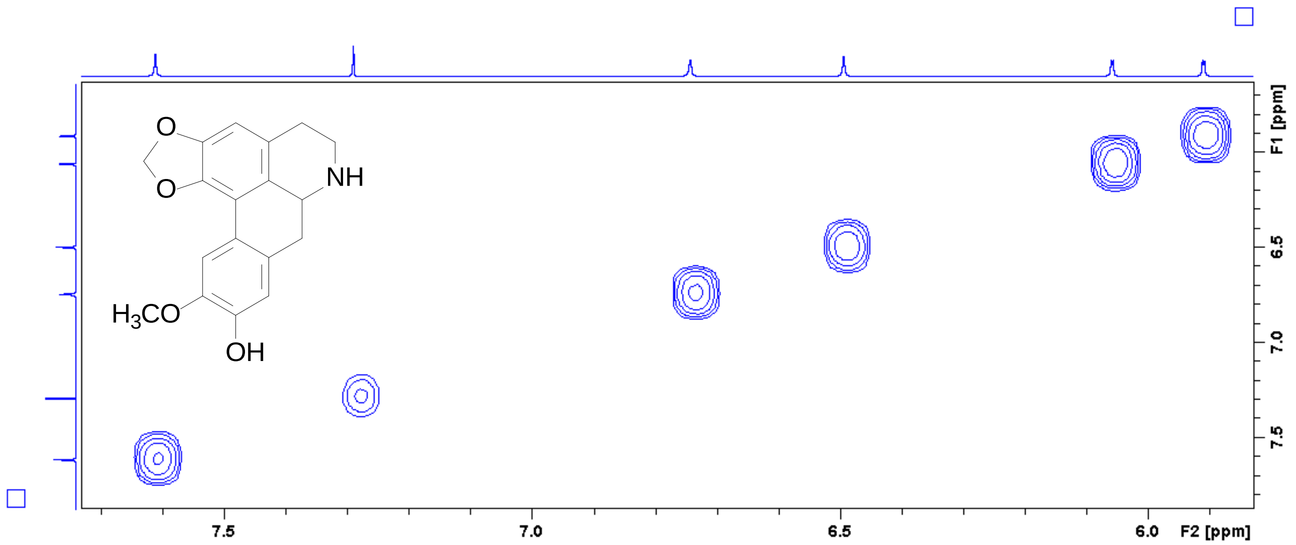

Figure S143. Expanded view 1 of the ^{1}H–^{1}H COSY correlation map of **actinodaphnine** (**14**) recorded in $CDCl_3$ with a drop of CD_3OD at 500 MHz.

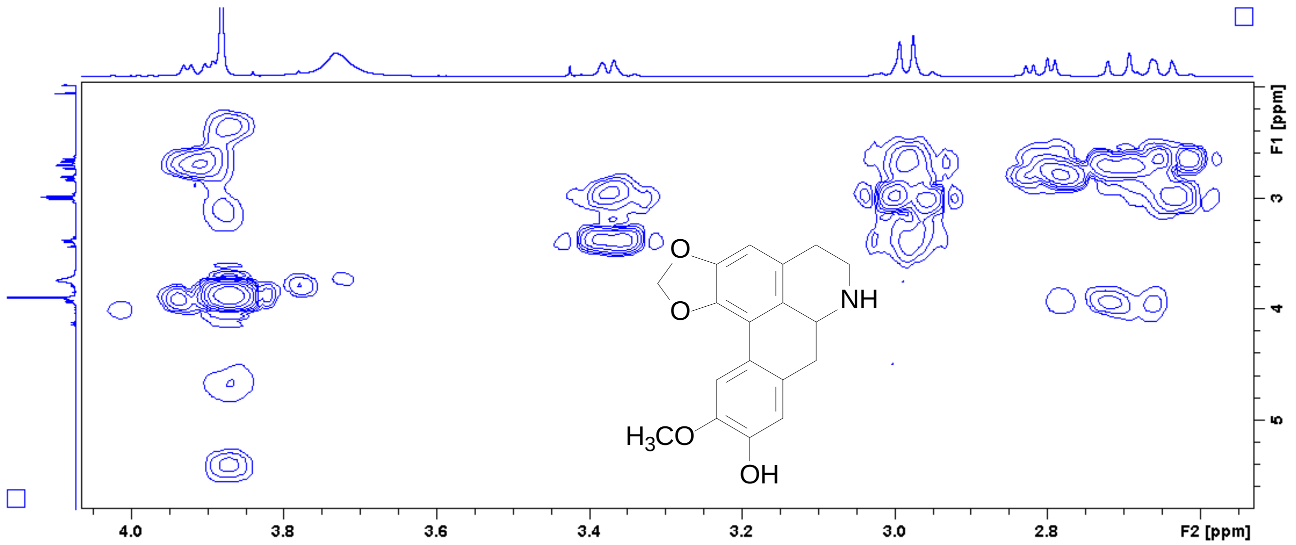

Figure S144. Expanded view 2 of the ^{1}H–^{1}H COSY correlation map of **actinodaphnine** (**14**) recorded in $CDCl_3$ with a drop of CD_3OD at 500 MHz.

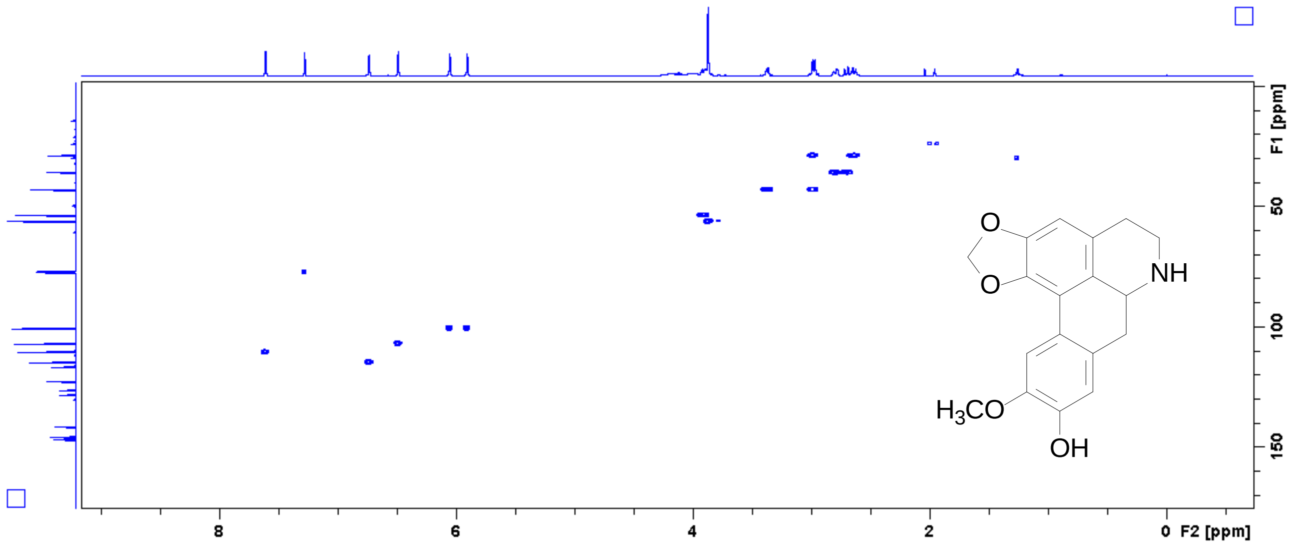

Figure S145. One-bond ^{1}H–^{13}C HSQC correlation map of **actinodaphnine** (**14**) recorded in $CDCl_3$ with a drop of CD_3OD at 500 MHz (^{1}H) and 125 MHz (^{13}C).

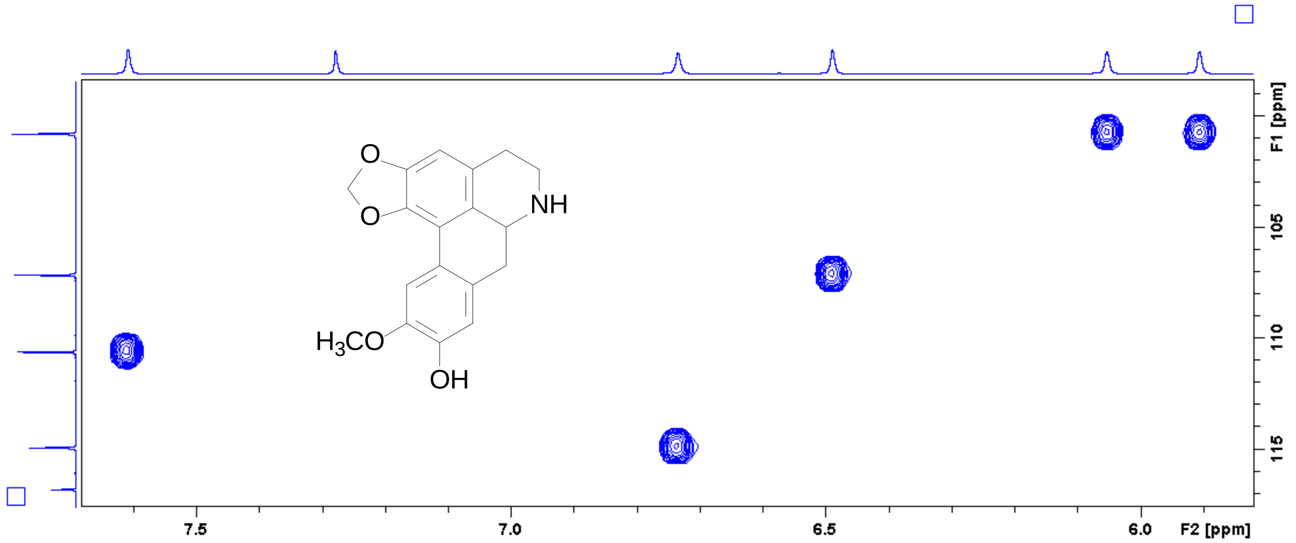

Figure S146. Expanded view 1 of the one-bond ^{1}H–^{13}C HSQC correlation map of **actinodaphnine** (**14**) recorded in $CDCl_3$ with a drop of CD_3OD at 500 MHz (^{1}H) and 125 MHz (^{13}C).

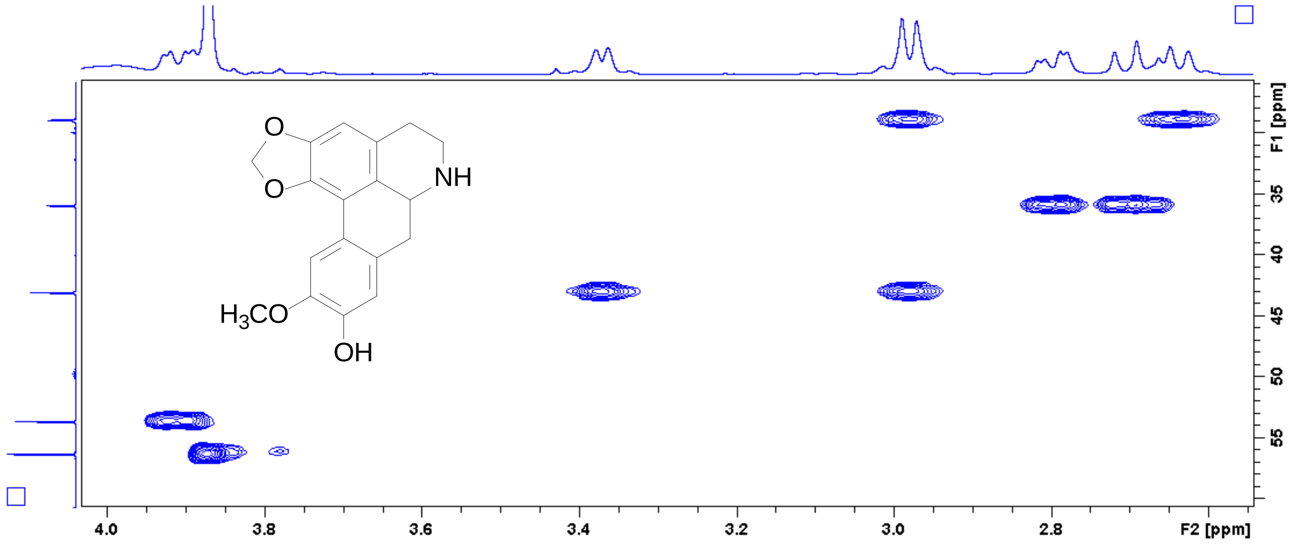

Figure S147. Expanded view 2 of the one-bond ^{1}H–^{13}C HSQC correlation of **actinodaphnine** (**14**) recorded in $CDCl_3$ with a drop of CD_3OD at 500 MHz (^{1}H) and 125 MHz (^{13}C).

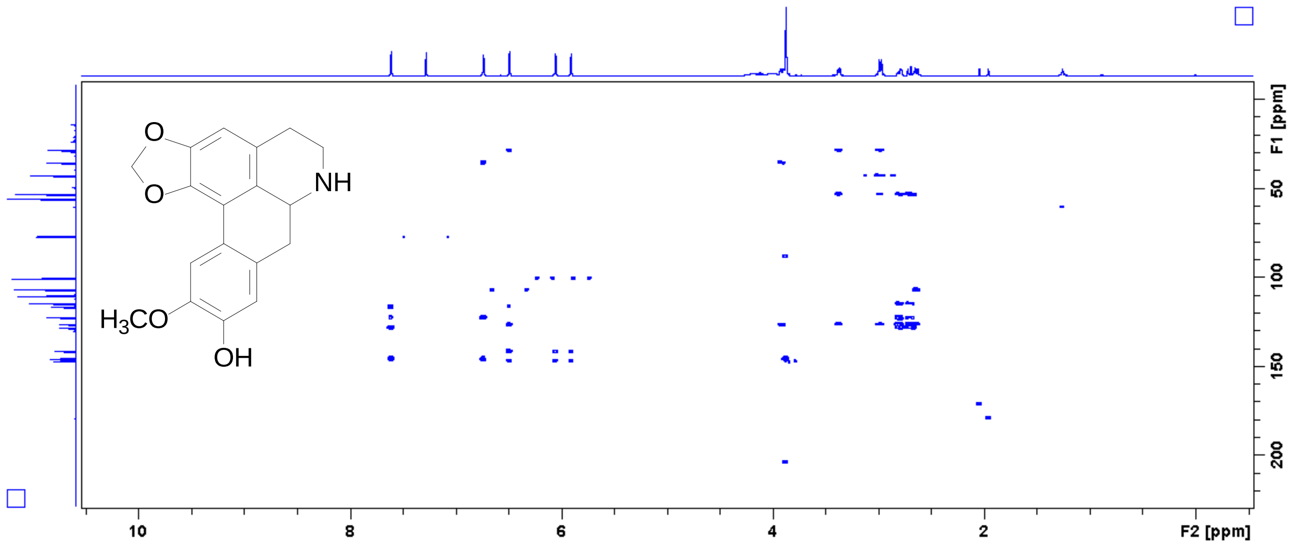

Figure S148. Long-range ^{1}H–^{13}C HMBC correlation map of **actinodaphnine** (**14**) recorded in $CDCl_3$ with a drop of CD_3OD at 500 MHz (^{1}H) and 125 MHz (^{13}C).

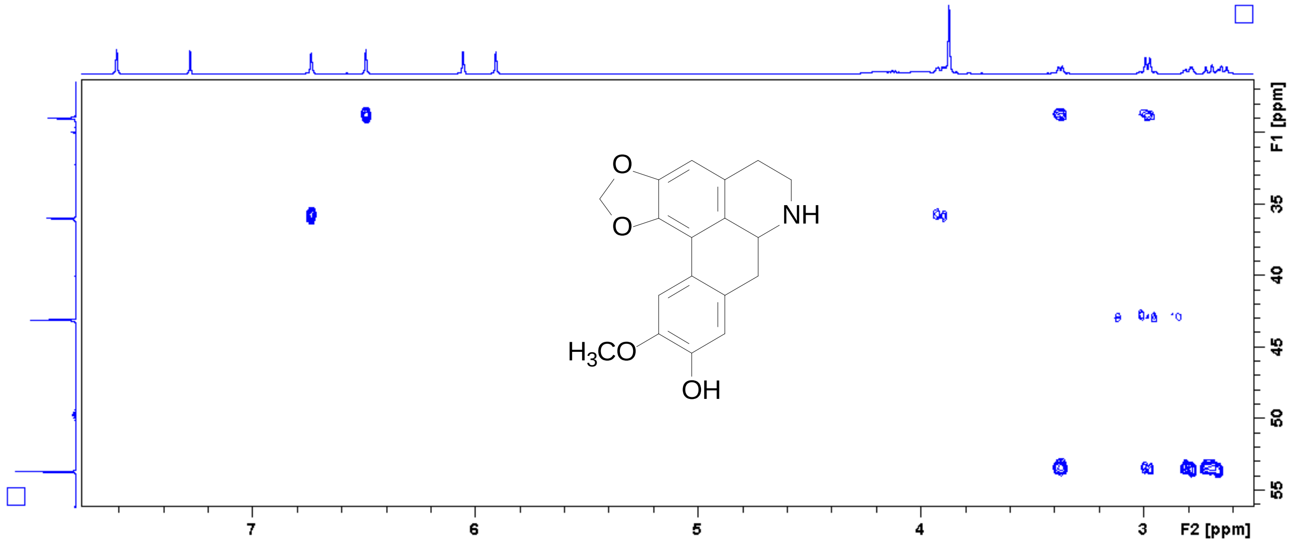

Figure S149. Expanded view 1 of the long-range ^{1}H–^{13}C HMBC correlation map of **actinodaphnine** (**14**) recorded in $CDCl_3$ with a drop of CD_3OD at 500 MHz (^{1}H) and 125 MHz (^{13}C).

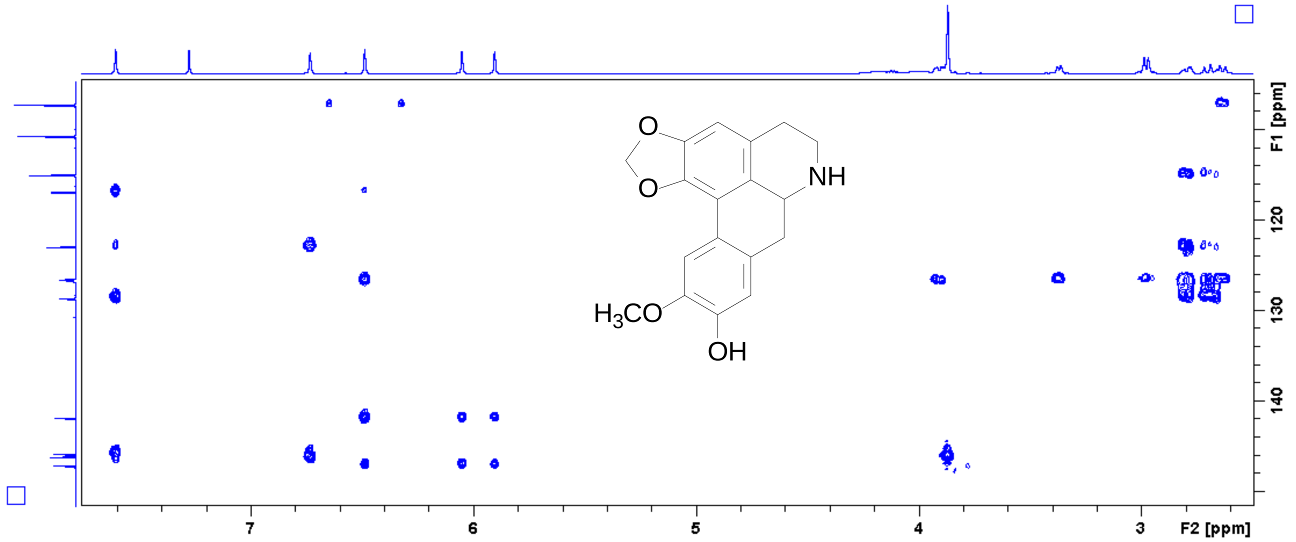

Figure S150. Expanded view 2 of the long-range ^{1}H–^{13}C HMBC correlation map of **actinodaphnine** (**14**) recorded in $CDCl_3$ with a drop of CD_3OD at 600 MHz (^{1}H) and 150 MHz (^{13}C).

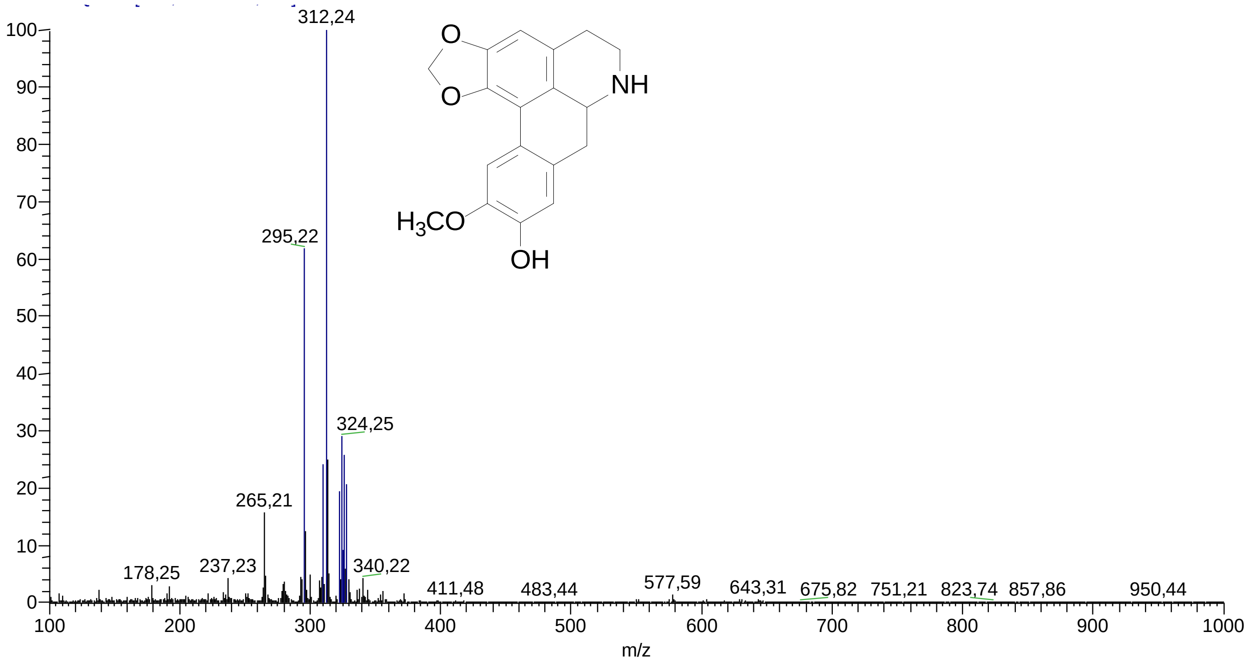

Figure S151. LR-APCI(+)-MS spectrum of **actinodaphnine** (**14**), showing the protonated molecular ion at *m/z* 312 $[M + H]^+$.

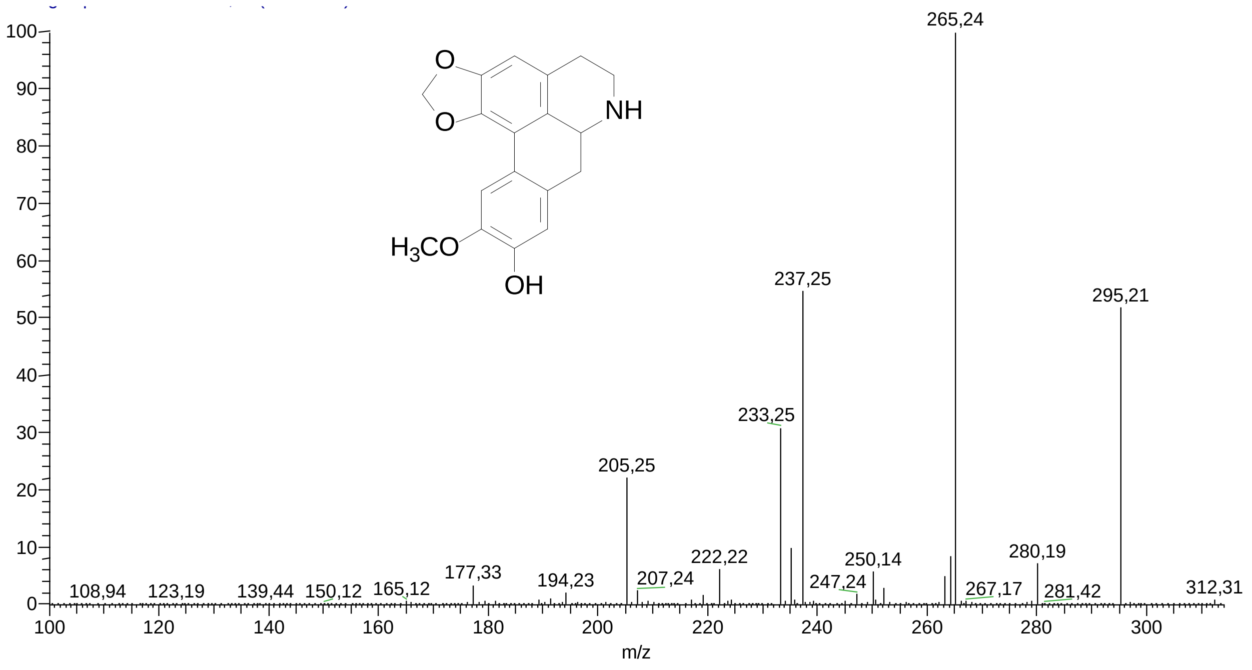

Figure S152. LR-APCI(+)-MS/MS spectrum of the protonated molecule of **actinodaphnine** (**14**) at *m/z* 312 $[M + H]^+$.

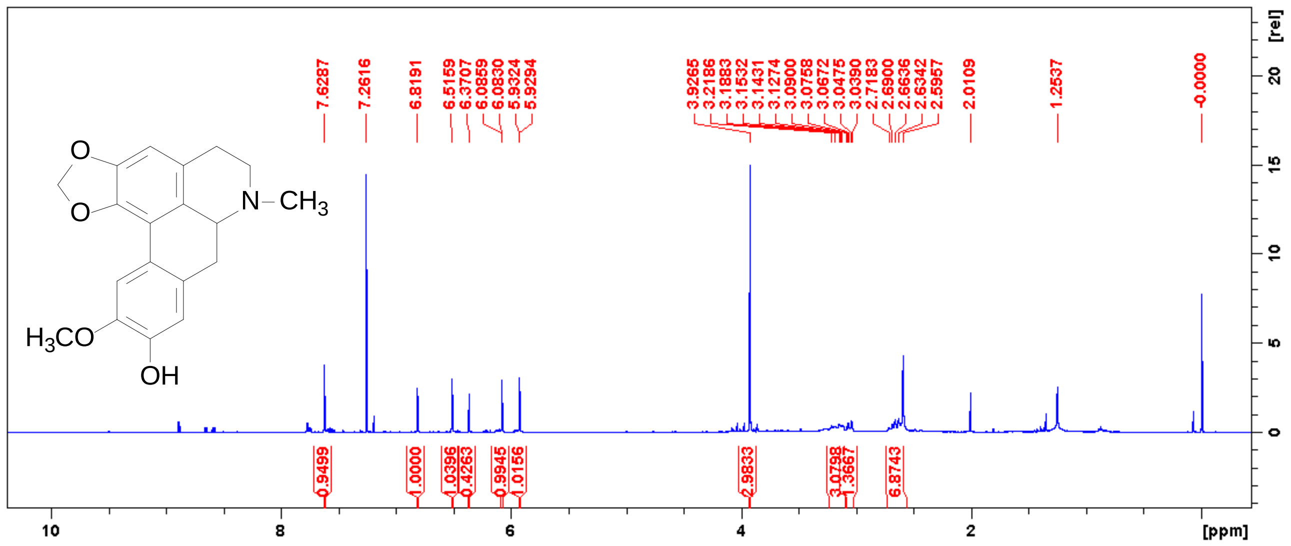

Figure S153. Full ^{1}H NMR spectrum of ***N*-methylactinodaphnine** (**15**) recorded in $CDCl_3$ at 500 MHz.

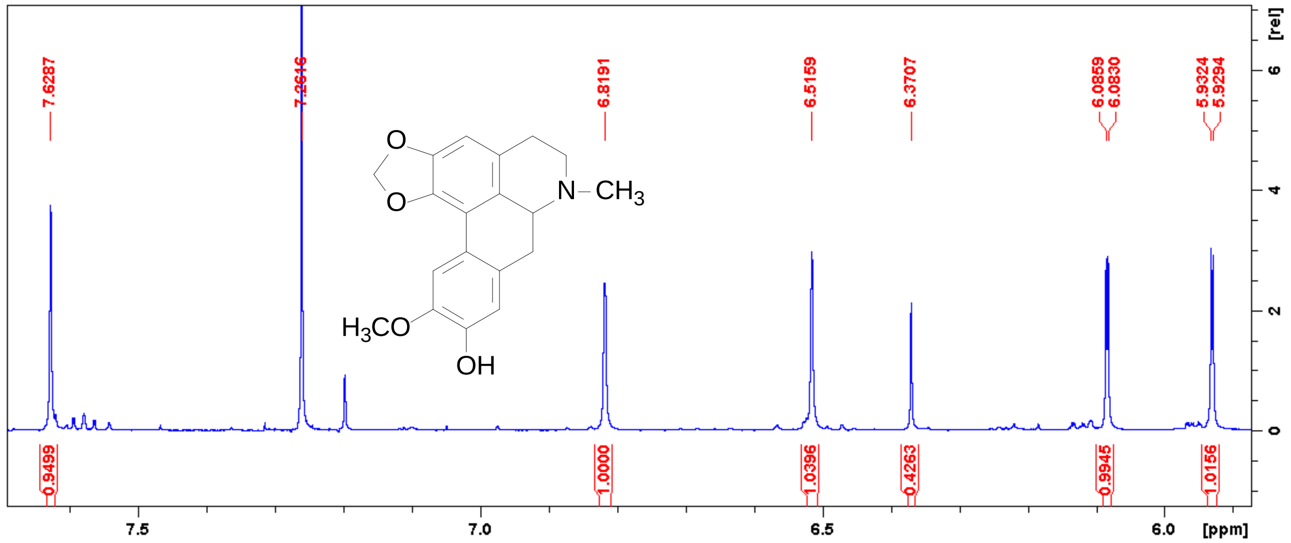

Figure S154. Expanded view 1 of the aromatic proton region in the ^{1}H NMR spectrum of ***N*-methylactinodaphnine** (**15**) recorded in $CDCl_3$ at 500 MHz.

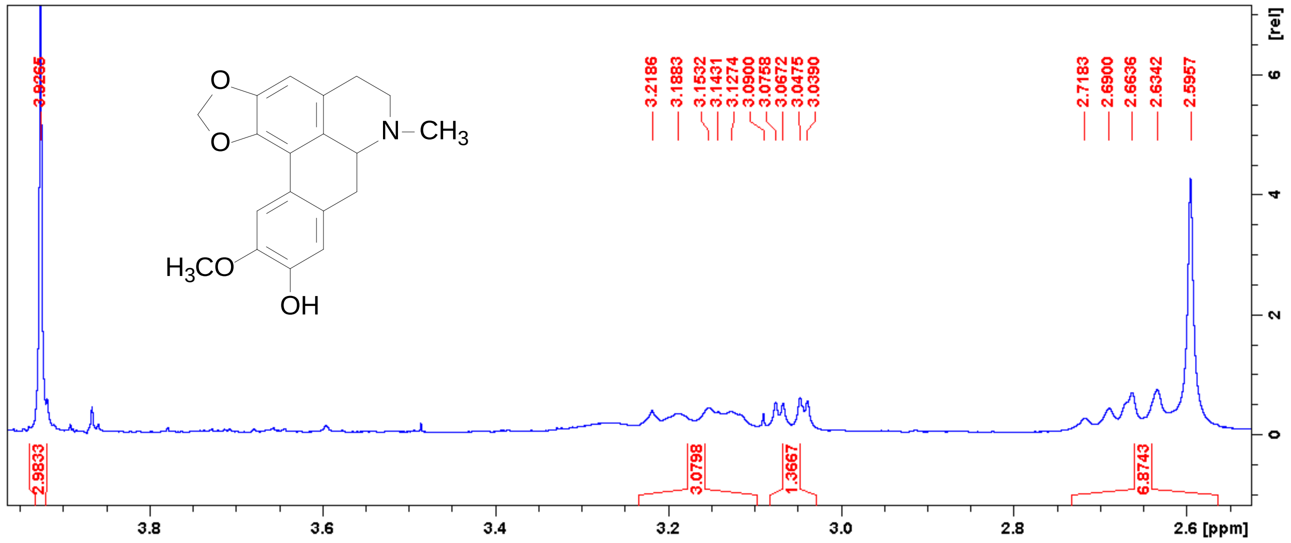

Figure S155. Expanded view 2 of the aliphatic proton region in the ^{1}H NMR spectrum ***N*-methylactinodaphnine** (**15**) recorded in $CDCl_3$ at 500 MHz.

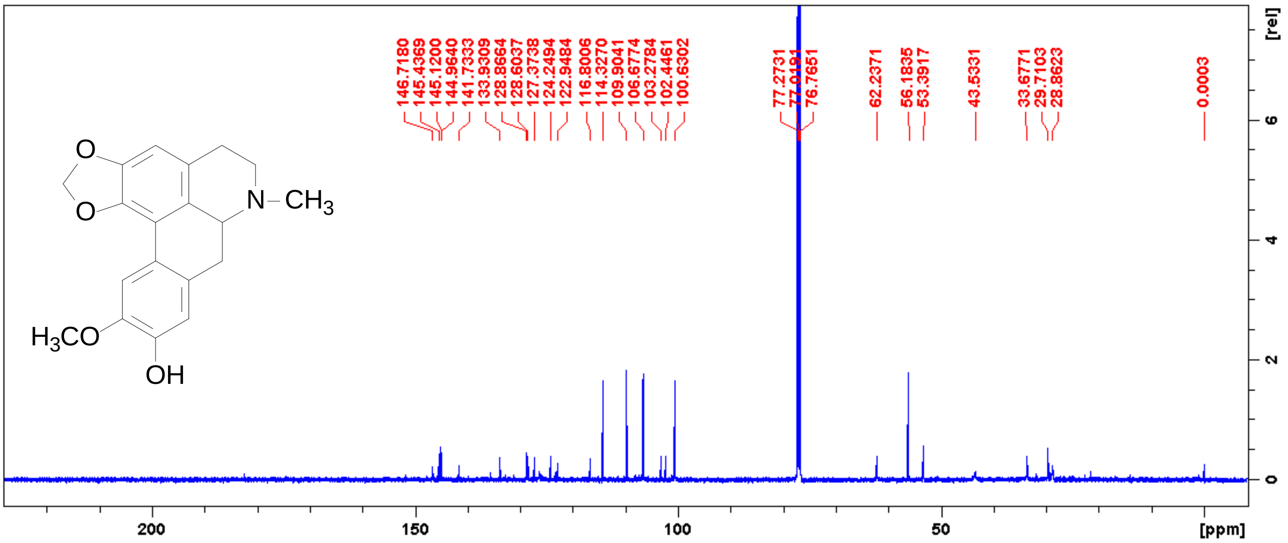

Figure S156. Full ^{13}C NMR spectrum of ***N*-methylactinodaphnine** (**15**) recorded in $CDCl_3$ at 125 MHz.

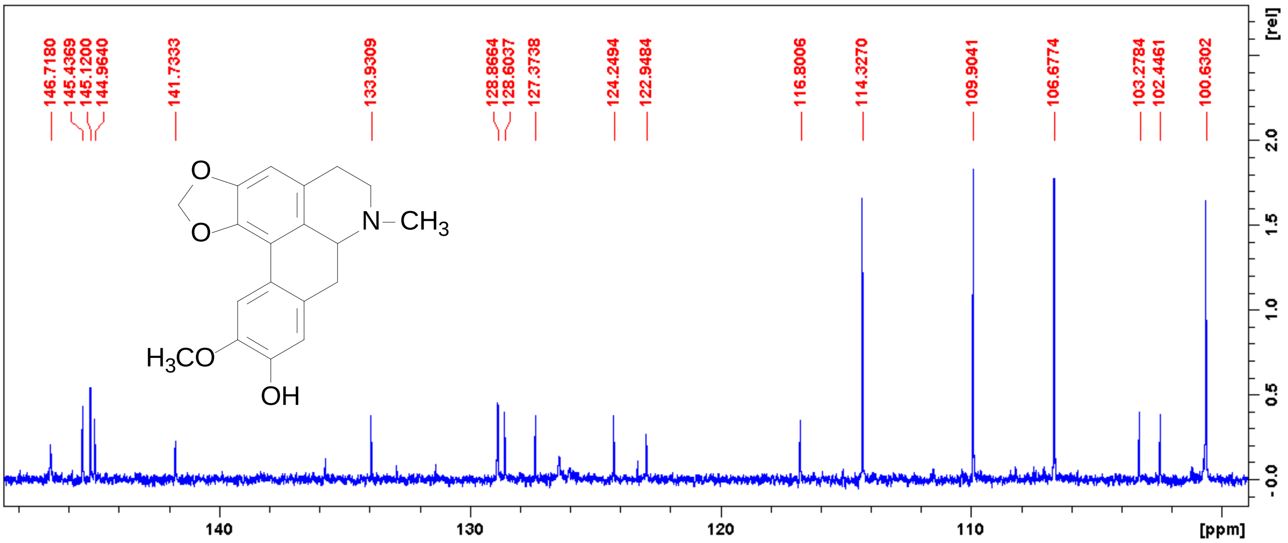

Figure S157. Expanded view of the carbon region (δ_C 148.5–99.0 ppm) in the ^{13}C NMR spectrum of ***N*-methylactinodaphnine** (**15**) recorded in $CDCl_3$ at 125 MHz.

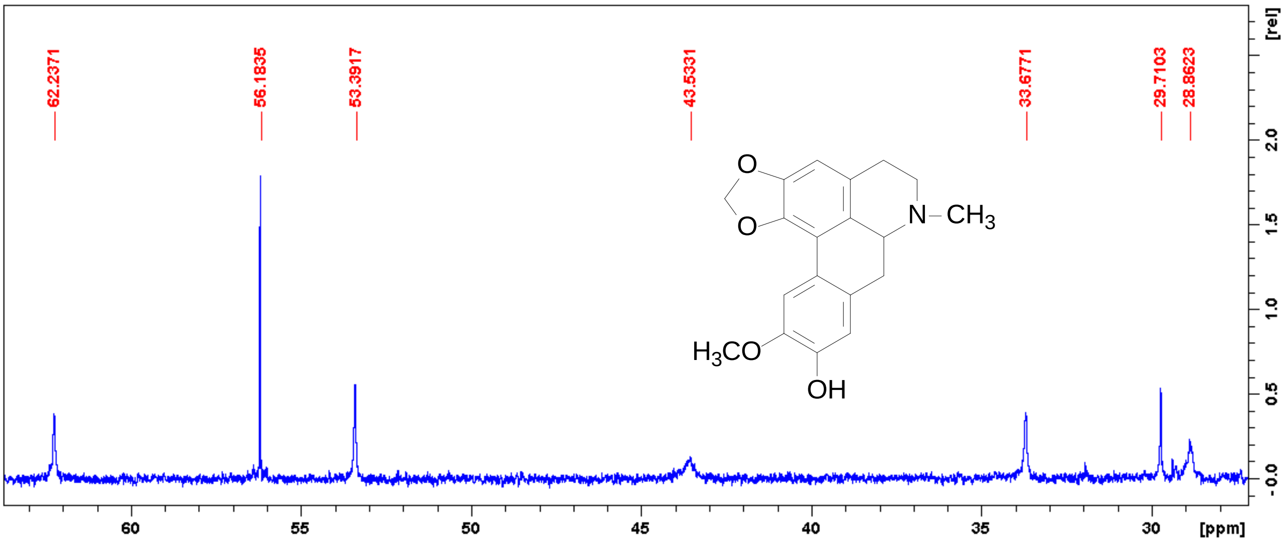

Figure S158. Expanded view of the carbon region (δ_C 63.6–27.2 ppm) in the ^{13}C NMR spectrum of ***N*-methylactinodaphnine** (**15**) recorded in $CDCl_3$ at 125 MHz.

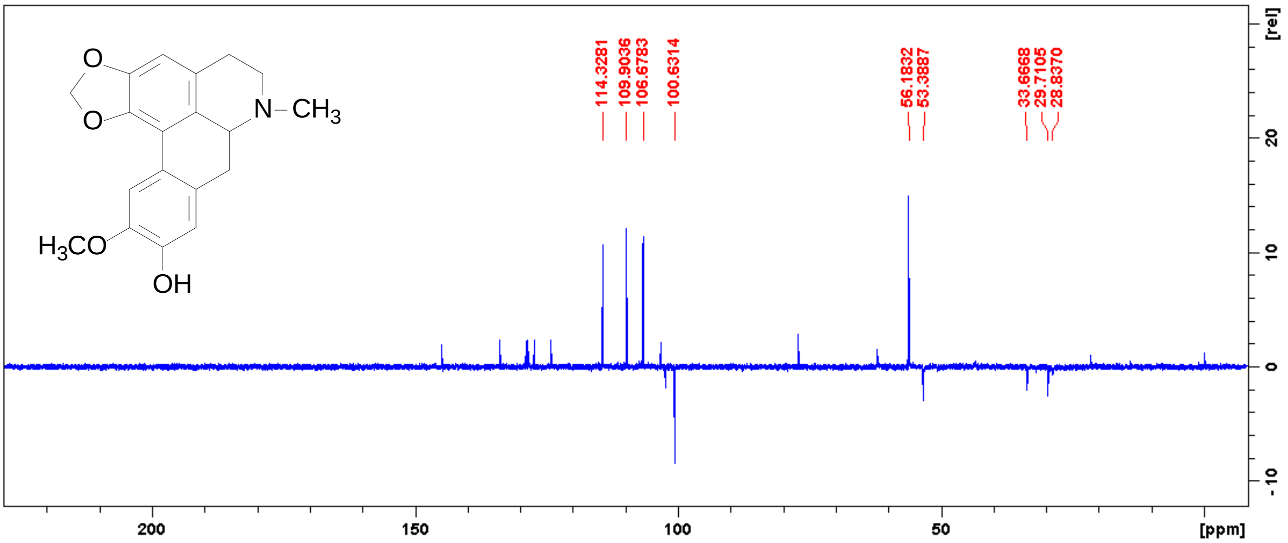

Figure S159. Full DEPT-135 ^{13}C NMR spectrum of ***N*-methylactinodaphnine** (**15**) recorded in $CDCl_3$ at 125 MHz.

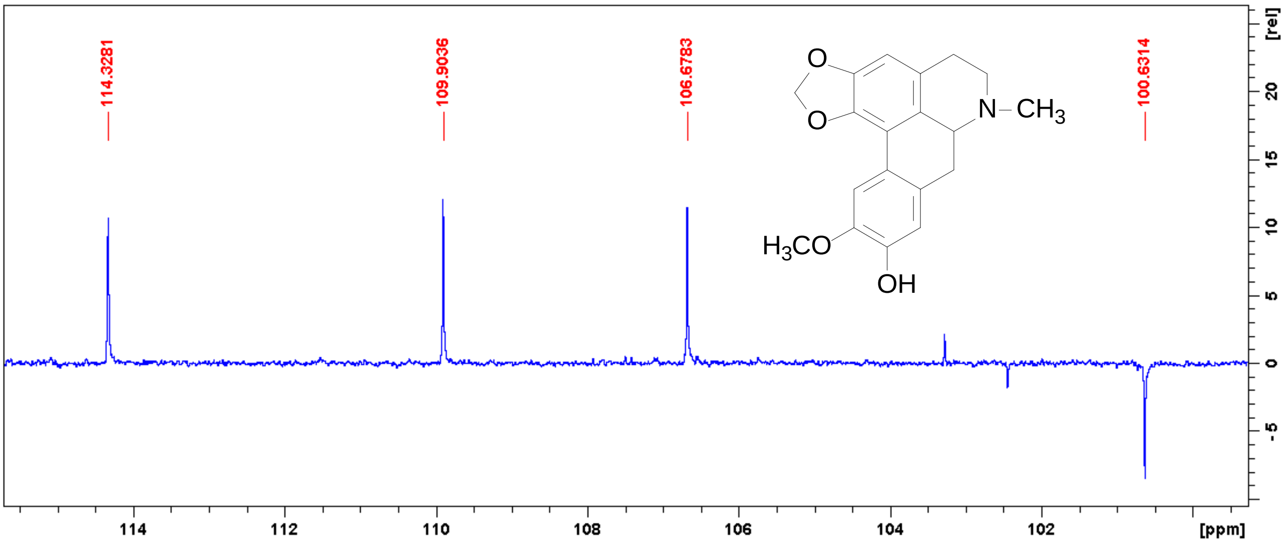

Figure S160. Expanded view of the carbon region (δ_C 115.6–99.3 ppm) in the DEPT-135 ^{13}C NMR spectrum of ***N*-methylactinodaphnine** (**15**) recorded in $CDCl_3$ at 125 MHz.

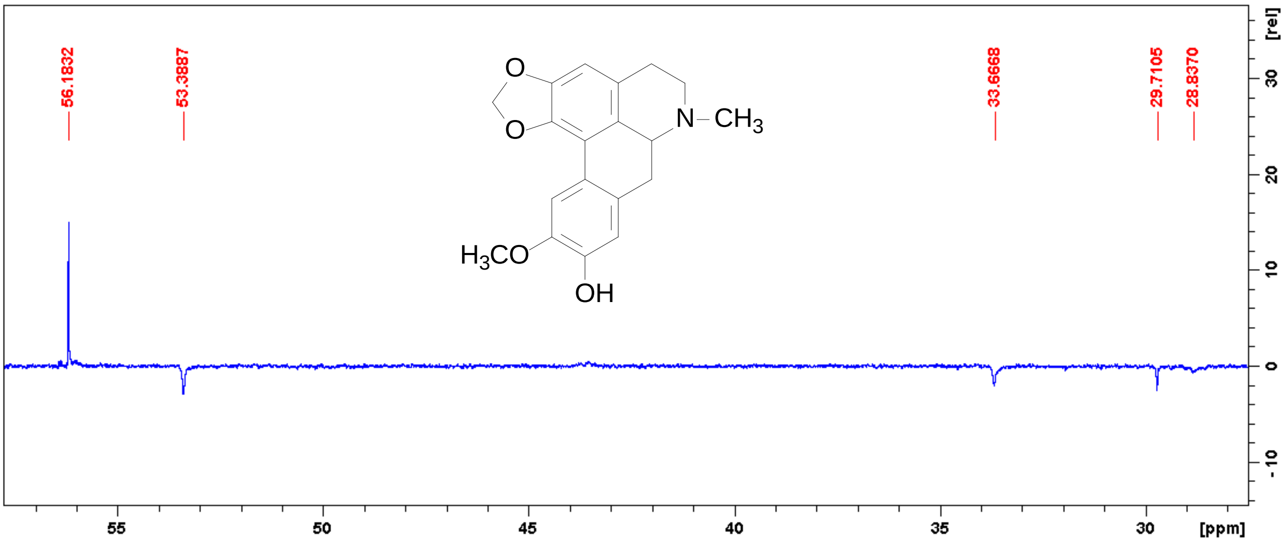

Figure S161. Expanded view of the carbon region (δ_C 57.2–28.4 ppm) in the DEPT-135 ^{13}C NMR spectrum of ***N*-methylactinodaphnine** (**15**) recorded in $CDCl_3$ at 125 MHz.

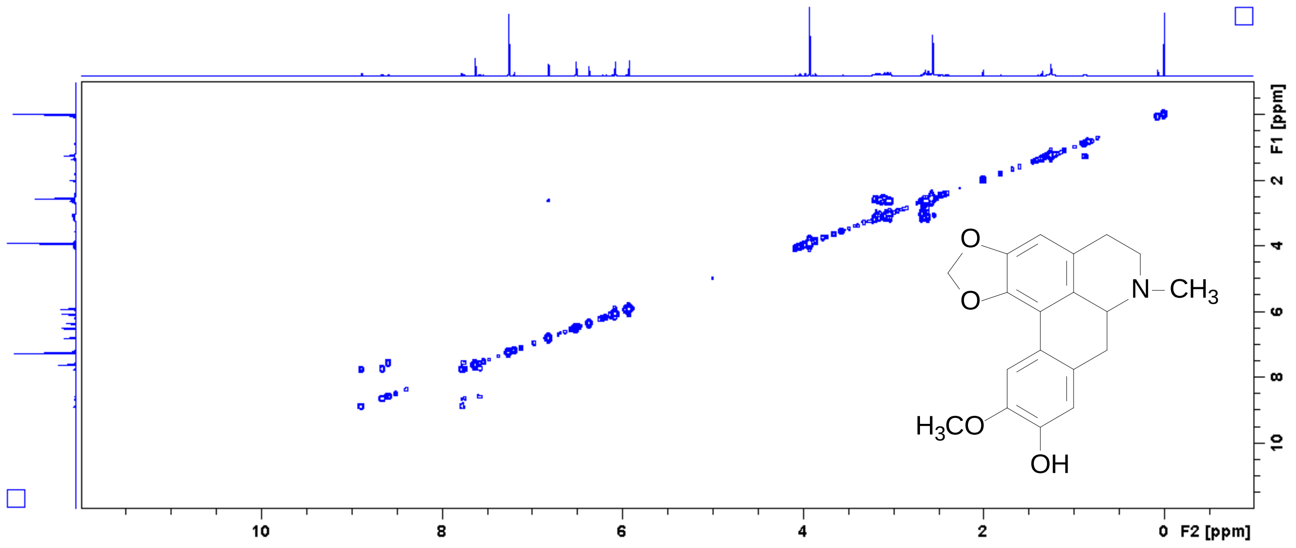

Figure S162. ^1H–^1H COSY correlation map ***N*-methylactinodaphnine** (**15**) recorded in $CDCl_3$ at 500 MHz.

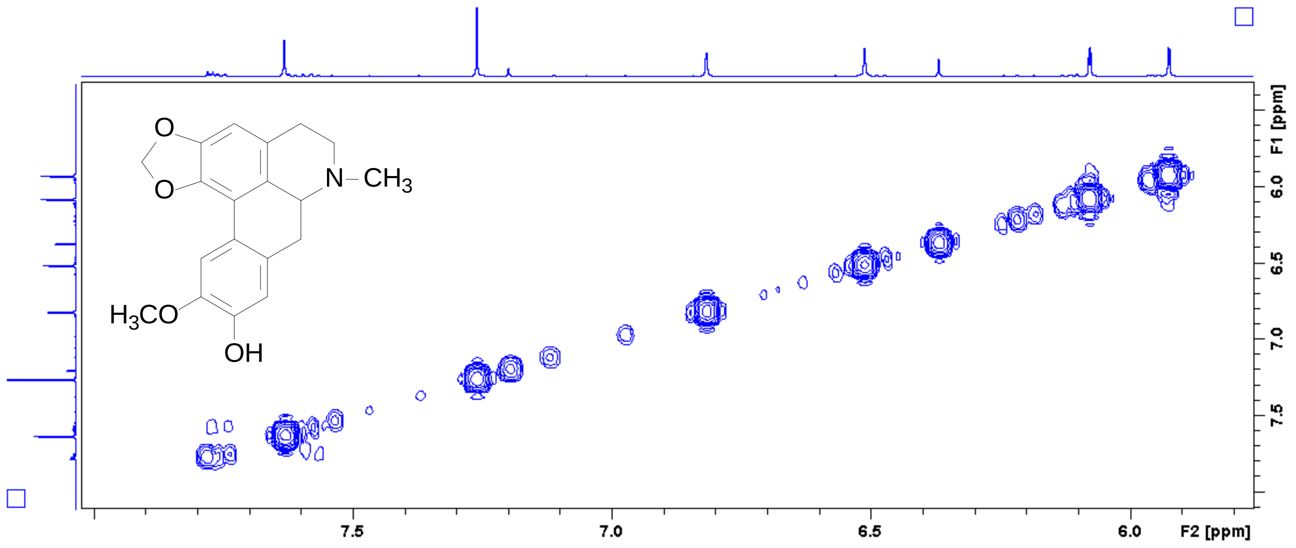

Figure S163. Expanded view 1 of the ^1H–^1H COSY correlation map of ***N*-methylactinodaphnine** (**15**) recorded in $CDCl_3$ at 500 MHz.

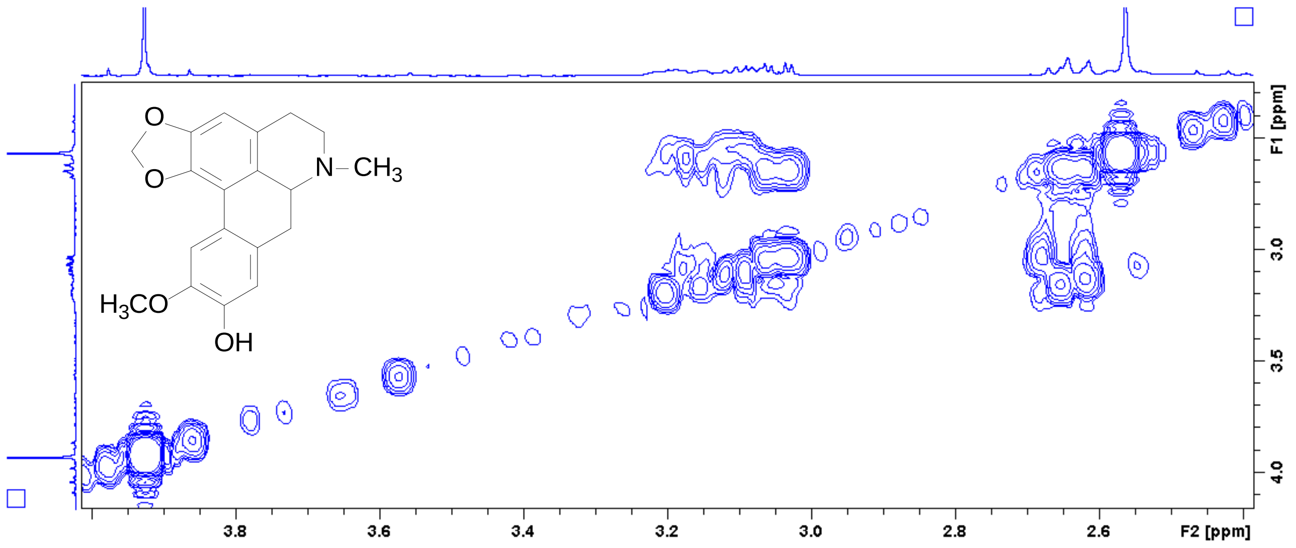

Figure S164. Expanded view 2 of the ^1H–^1H COSY correlation map of ***N*-methylactinodaphnine** (**15**) recorded in $CDCl_3$ at 500 MHz.

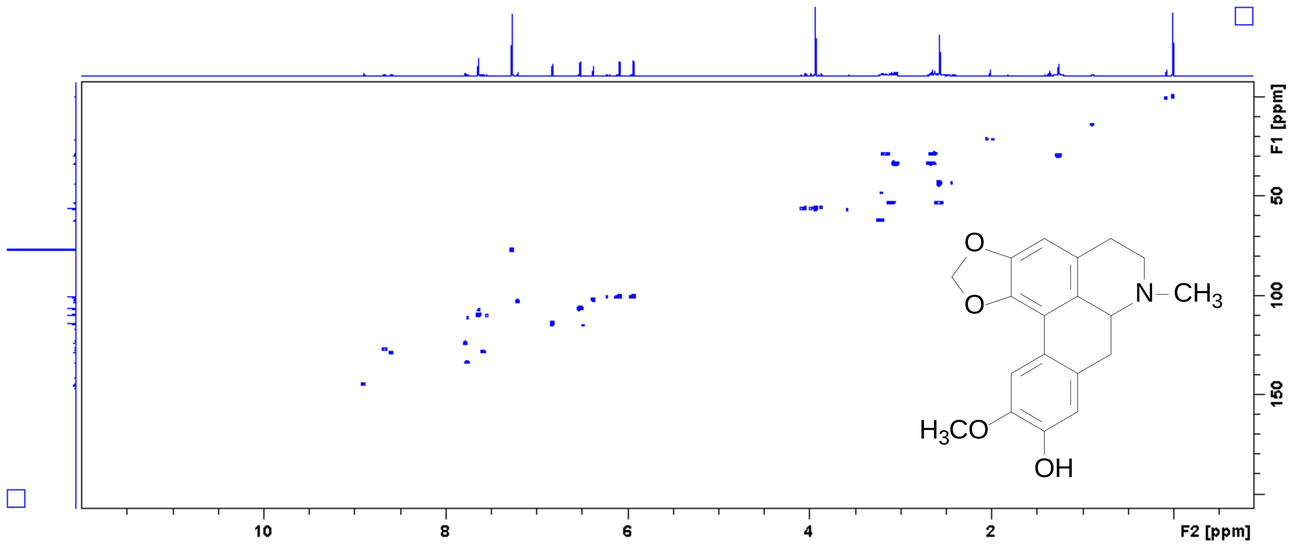

Figure S165. One-bond ^{1}H–^{13}C HSQC correlation map of ***N*-methylactinodaphnine** (**15**) recorded in $CDCl_3$ at 500 MHz (^{1}H) and 125 MHz (^{13}C).

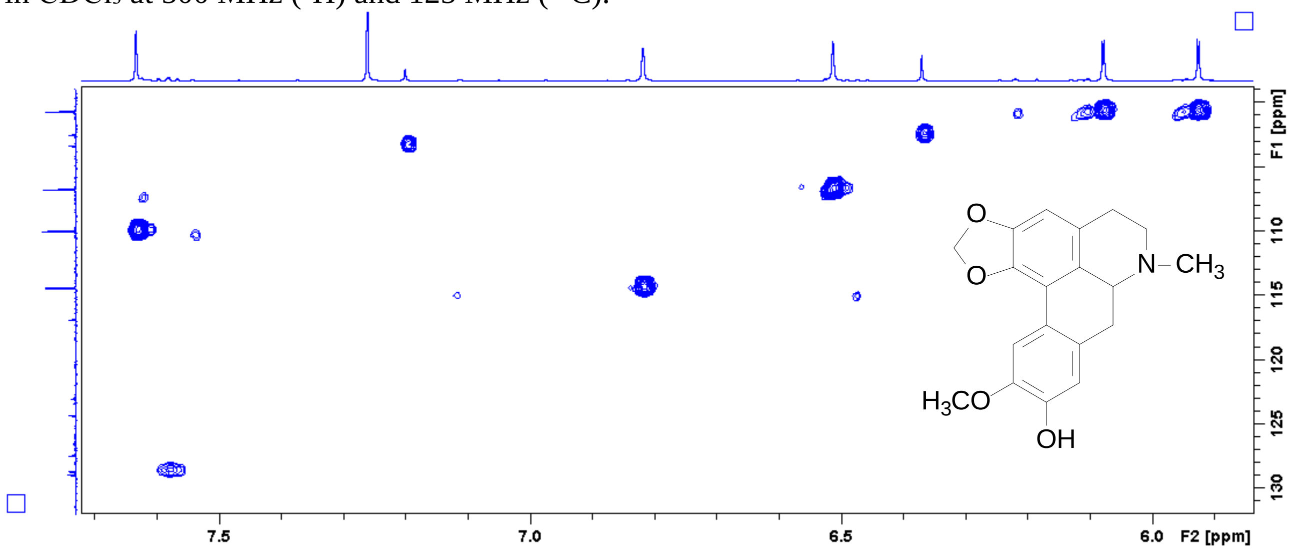

Figure S166. Expanded view 1 of the one-bond ^{1}H–^{13}C HSQC correlation map of ***N*-methylactinodaphnine** (**15**) recorded in $CDCl_3$ at 500 MHz (^{1}H) and 125 MHz (^{13}C).

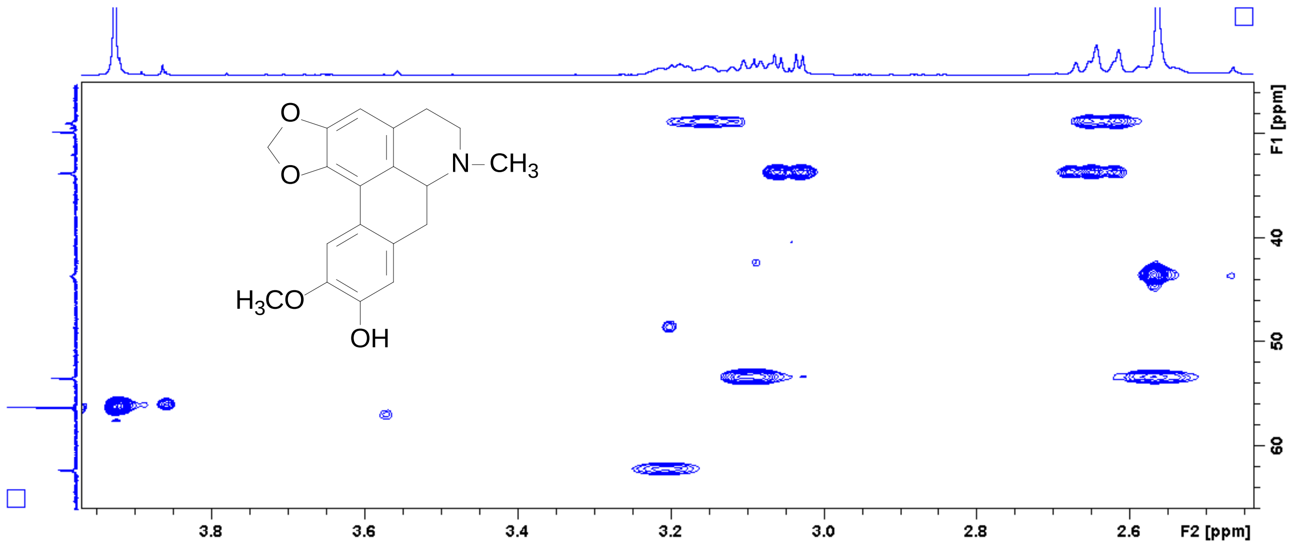

Figure S167. Expanded view 2 of the one-bond ^{1}H–^{13}C HSQC correlation map of ***N*-methylactinodaphnine** (**15**) recorded in $CDCl_3$ at 500 MHz (^{1}H) and 125 MHz (^{13}C).

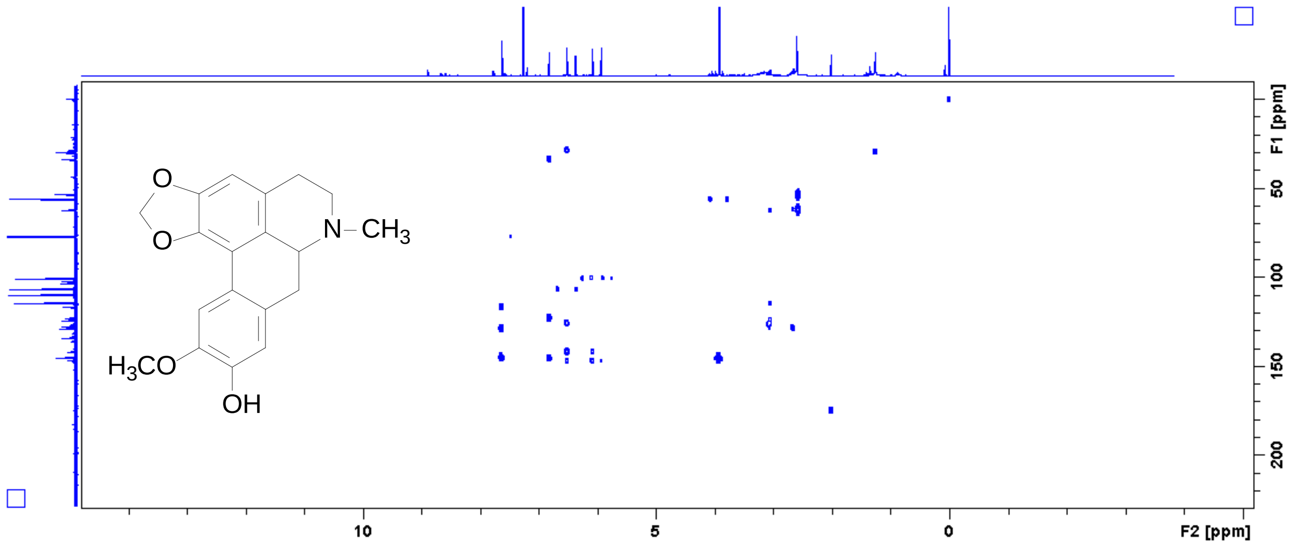

Figure S168. Long-range ^{1}H–^{13}C HMBC correlation map of ***N*-methylactinodaphnine** (**15**) recorded in $CDCl_3$ at 500 MHz (^{1}H) and 125 MHz (^{13}C).

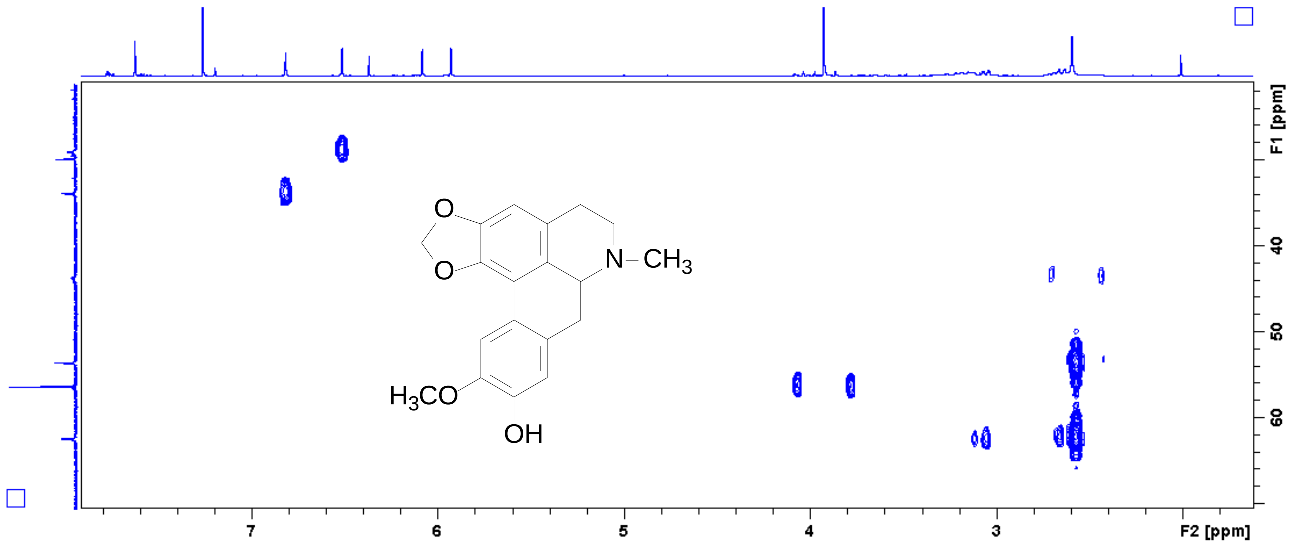

Figure S169. Expanded view 1 of the long-range ^{1}H–^{13}C HMBC correlation map of ***N*-methylactinodaphnine** (**15**) recorded in $CDCl_3$ at 500 MHz (^{1}H) and 125 MHz (^{13}C).

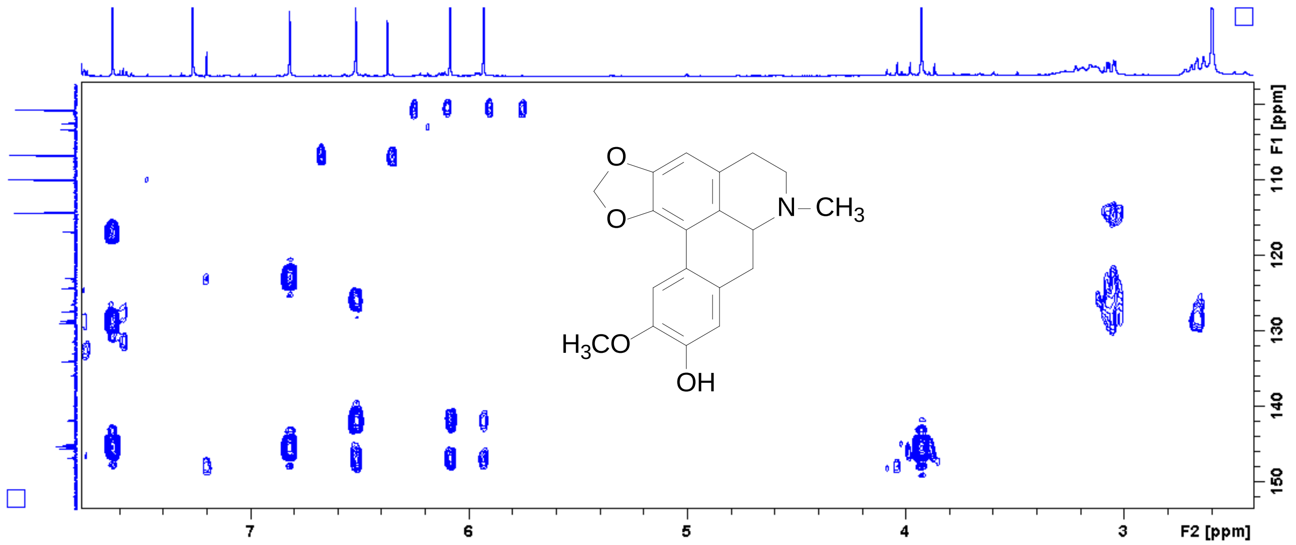

Figure S170. Expanded view 2 of the long-range ^{1}H–^{13}C HMBC correlation map of ***N*-methylactinodaphnine** (**15**) recorded in $CDCl_3$ at 500 MHz (^{1}H) and 125 MHz (^{13}C).

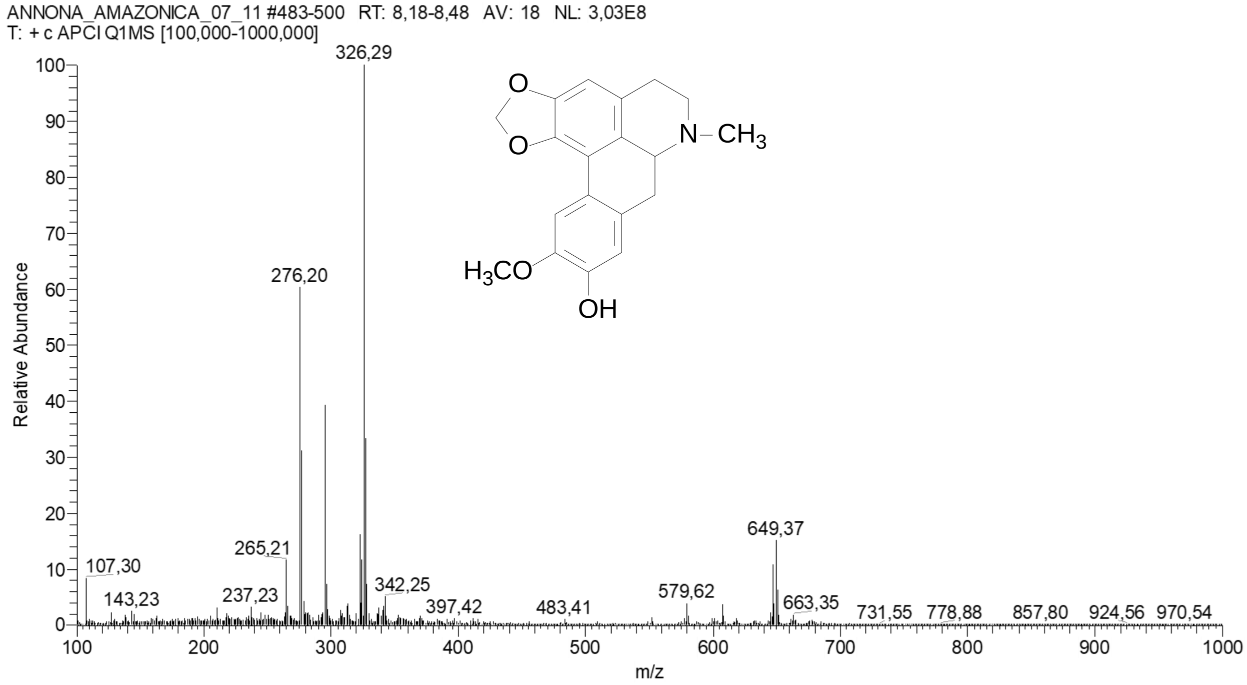

Figure S171. LR-APCI(+)-MS spectrum of ***N*-methylactinodaphnine** (**15**), showing the protonated molecular ion at *m/z* 326 $[M + H]^+$.

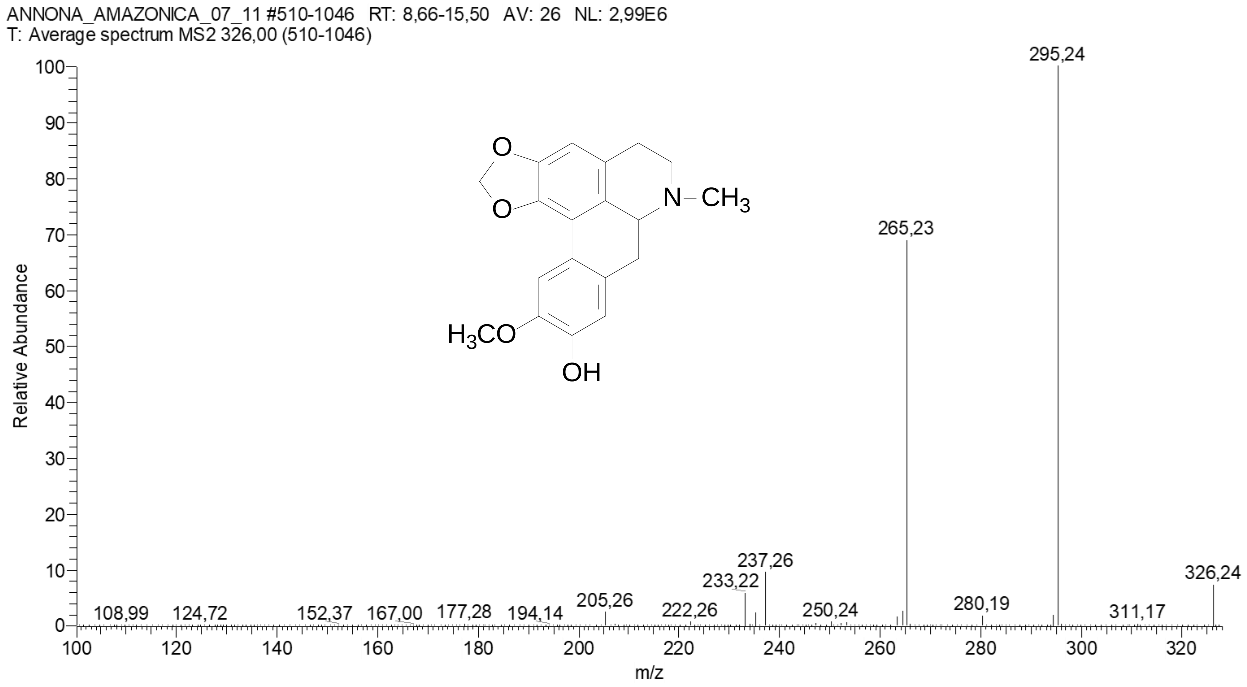

Figure S172. LR-APCI(+)-MS/MS spectrum of the protonated molecule of ***N*-methylactinodaphnine** (**15**) at *m/z* 326 $[M + H]^+$.

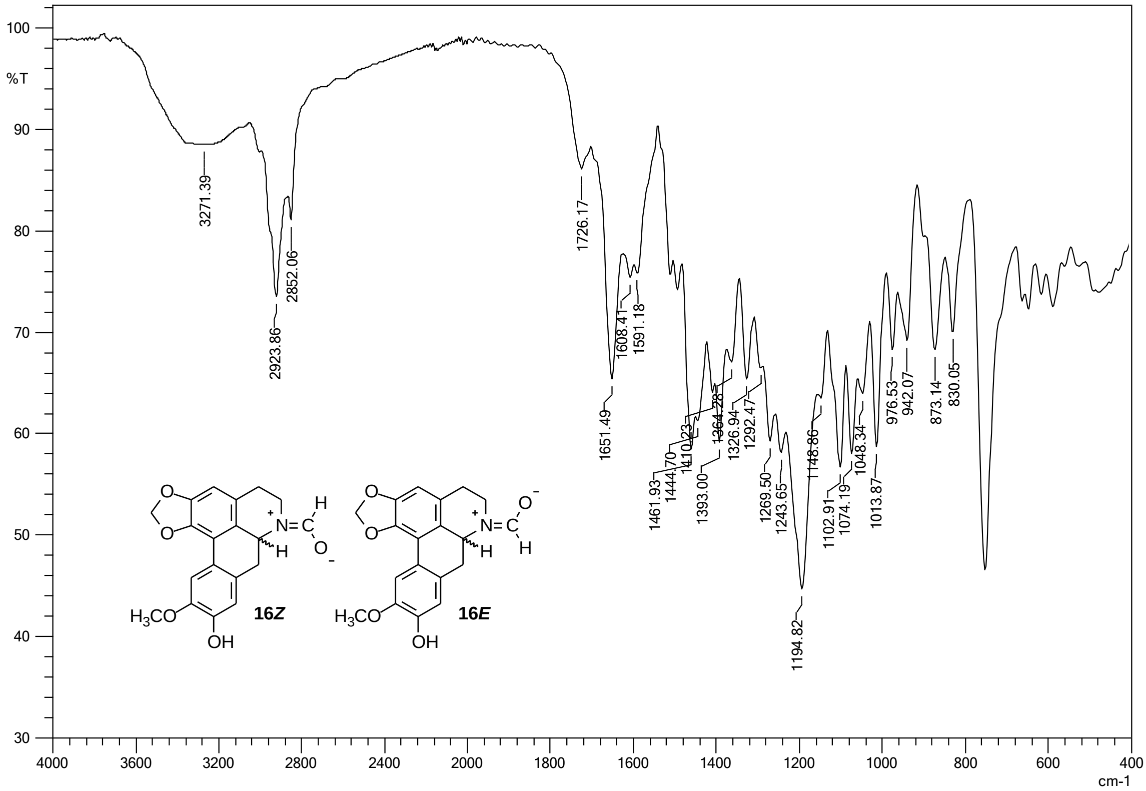

Figure S173. FTIR spectrum (KBr) of ***(Z/E)-N*-formylactinodaphnine** (**16**).

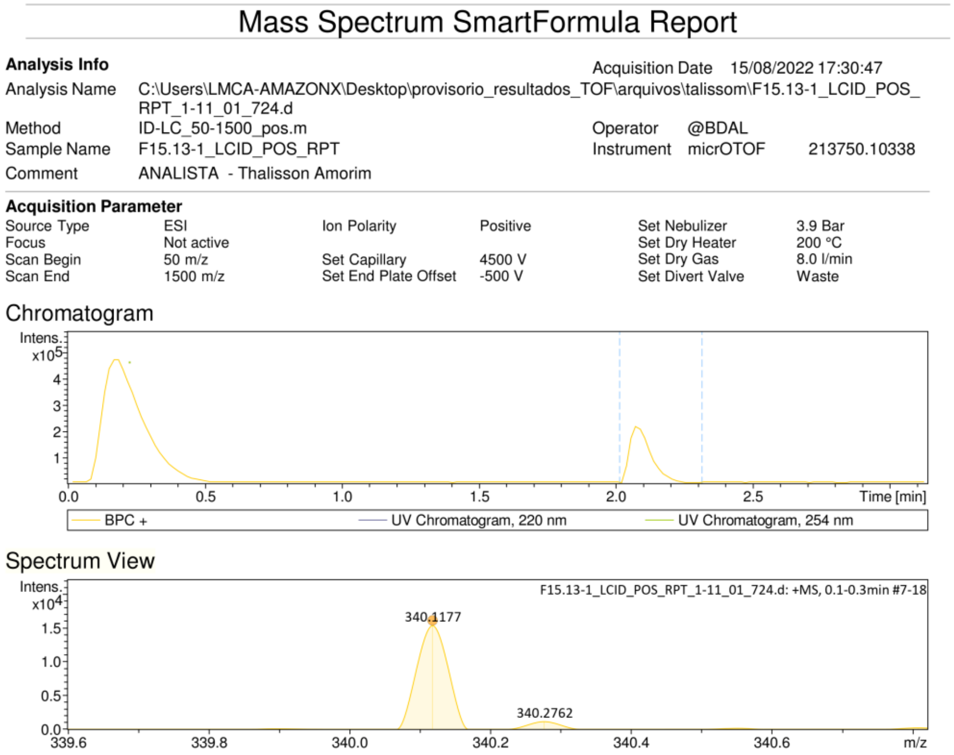

Figure S174. HRESIMS spectrum of ***(Z/E)-N*-formylactinodaphnine** (**16**), recorded in positive electrospray ionization mode, showing the protonated molecular ion at *m/z* 340.1177 $[M + H]^+$. The observed accurate mass is consistent with the molecular formula $C_{19}H_{17}NO_5$ (calcd for $C_{19}H_{18}NO_5^+$, m/z 340.1185; $\Delta = -2.3$ ppm). The upper panel shows the corresponding base peak chromatogram (BPC), while the lower panel presents the accurate-mass spectrum of compound **16**.

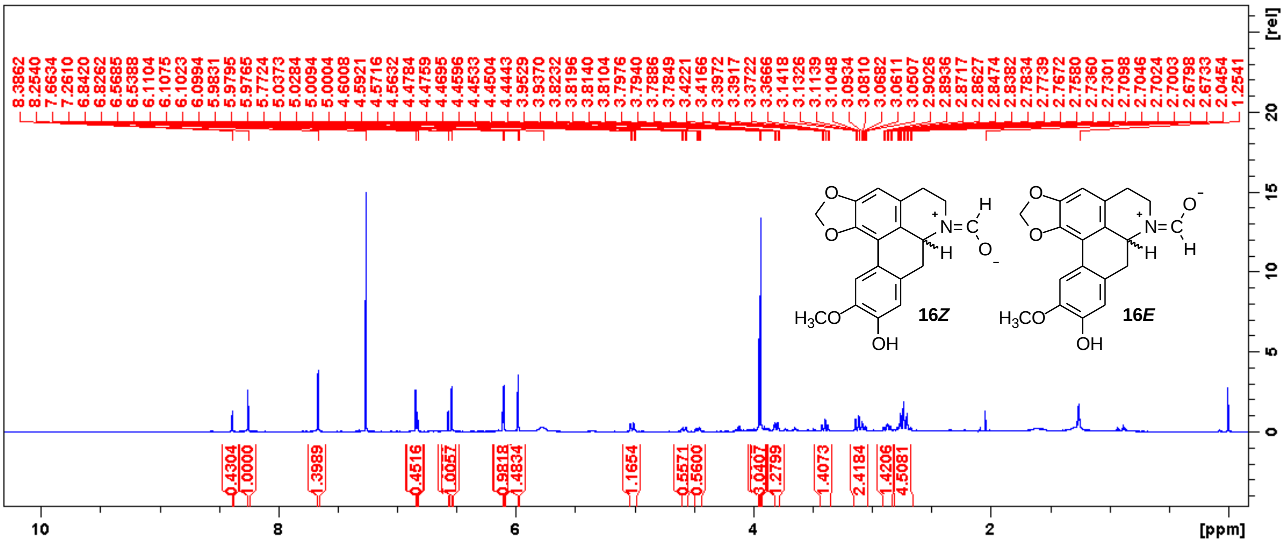

Figure S175. Full ^{1}H NMR spectrum of **(*Z*/*E*)-*N*-formylactinodaphnine** (**16**) recorded in $CDCl_3$ at 500 MHz. Direct integration of the resolved formyl singlets at δH 8.25 and 8.38 gave normalized values of 1.000 and 0.436, respectively, corresponding to an approximate major:minor ratio of 69.6:30.4 (reported as ca. 70:30). No formal statistical uncertainty was assigned because the estimate derives from a single spectrum rather than replicate quantitative NMR measurements.

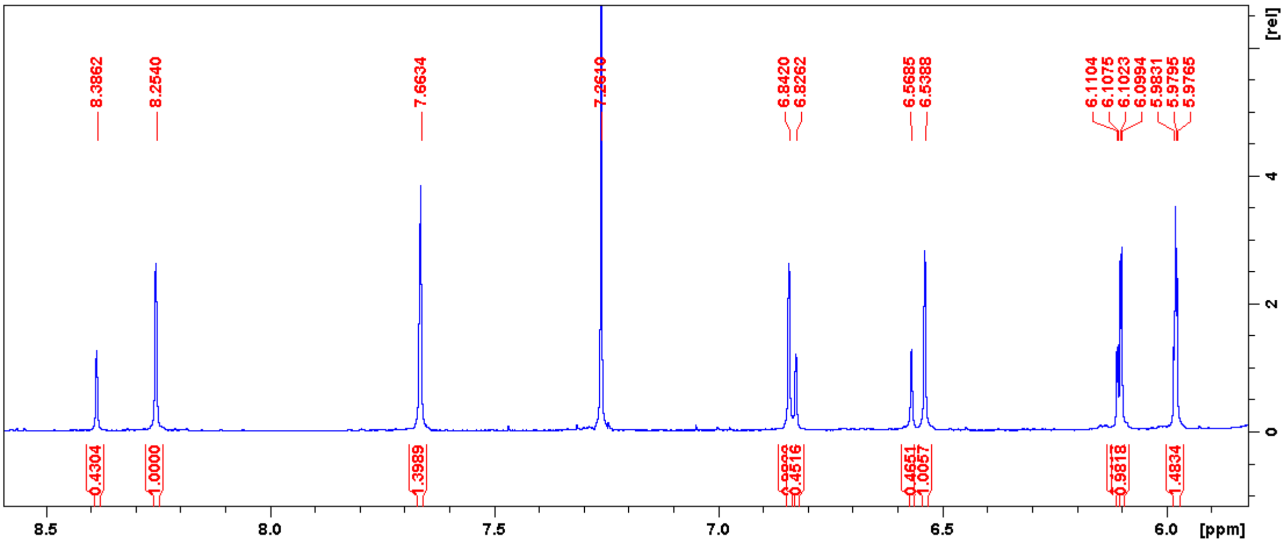

Figure S176. Expanded view 1 of the aromatic proton region in the ^{1}H NMR spectrum of **(*Z*/*E*)-*N*-formylactinodaphnine** (**16**) recorded in $CDCl_3$ at 500 MHz. Where duplicated resonances are resolved, the signal sets should be interpreted together with the major:minor assignment defined from the formyl signals in Figure S175.

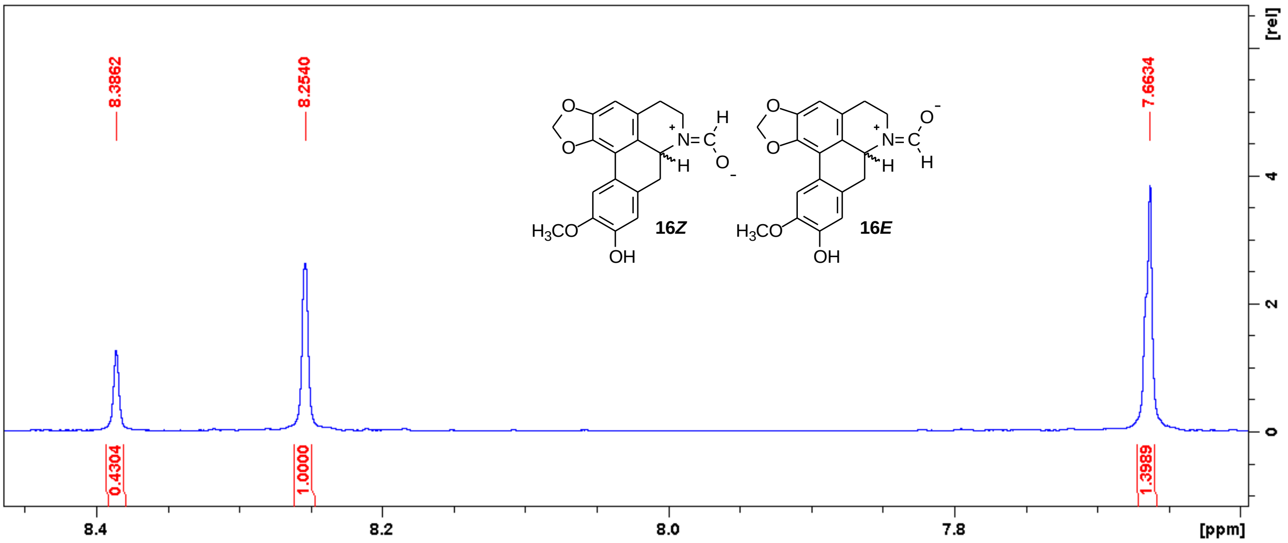

Figure S177. Expanded view 2 of the aromatic proton region in the ^{1}H NMR spectrum of **(*Z*/*E*)-*N*-formylactinodaphnine** (**16**) recorded in $CDCl_3$ at 500 MHz. Where duplicated resonances are resolved, the signal sets should be interpreted together with the major:minor assignment defined from the formyl signals in Figure S175.

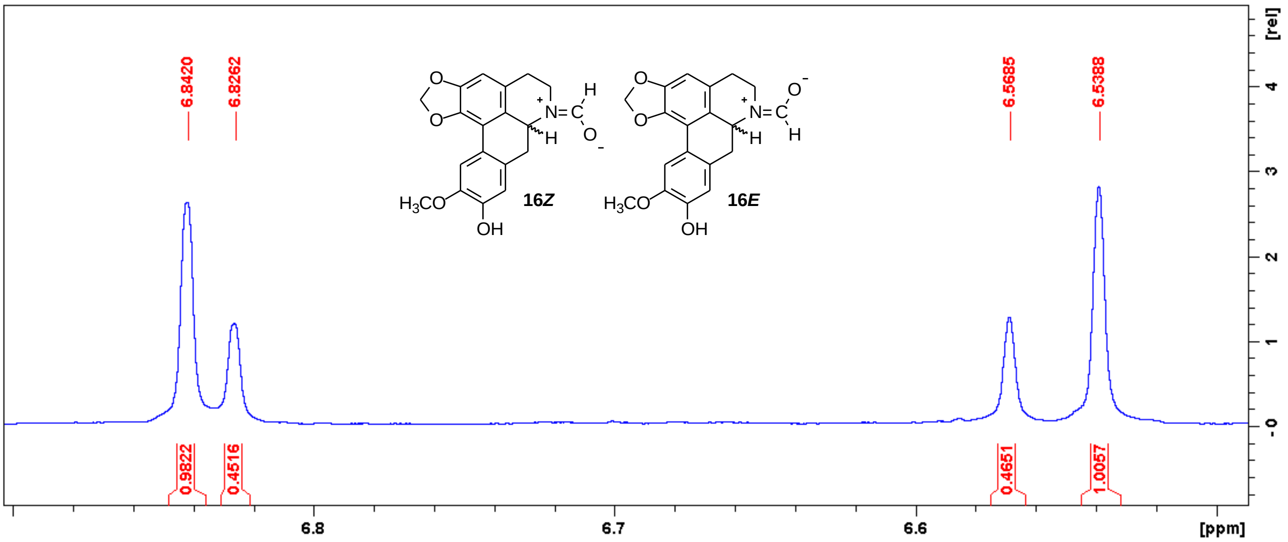

Figure S178. Expanded view 3 of the aromatic proton region in the ^{1}H NMR spectrum of **(*Z*/*E*)-*N*-formylactinodaphnine** (**16**) recorded in $CDCl_3$ at 500 MHz. Where duplicated resonances are resolved, the signal sets should be interpreted together with the major:minor assignment defined from the formyl signals in Figure S175.

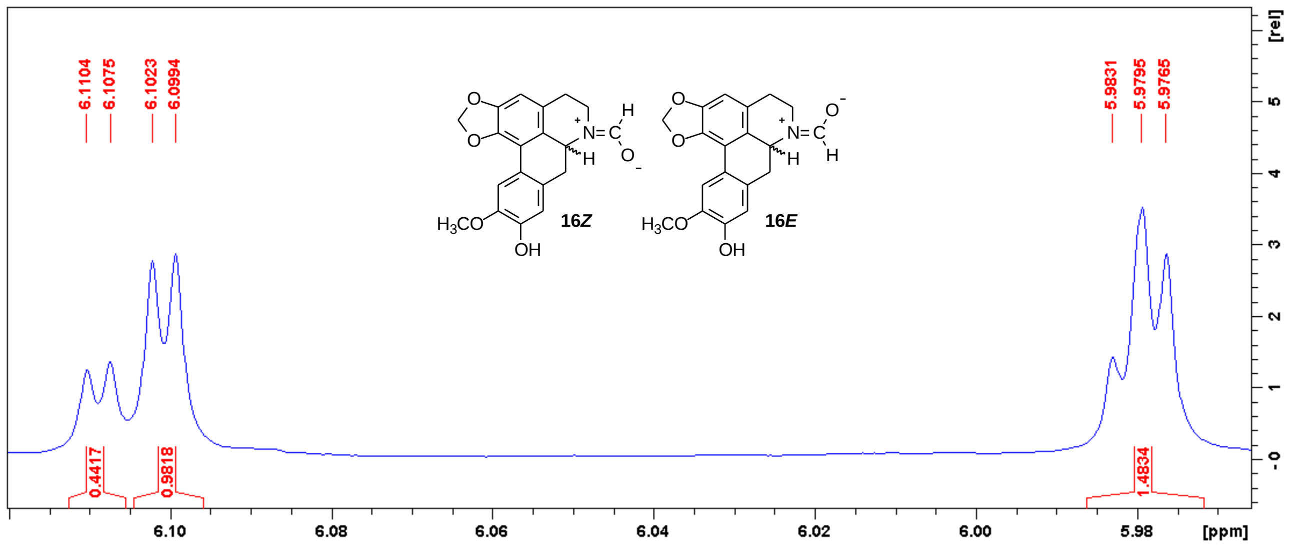

Figure S179. Expanded view 4 of the aromatic proton region in the ^{1}H NMR spectrum of **(*Z*/*E*)-*N*-formylactinodaphnine** (**16**) recorded in CDCl₃ at 500 MHz. Where duplicated resonances are resolved, the signal sets should be interpreted together with the major:minor assignment defined from the formyl signals in Figure S175.

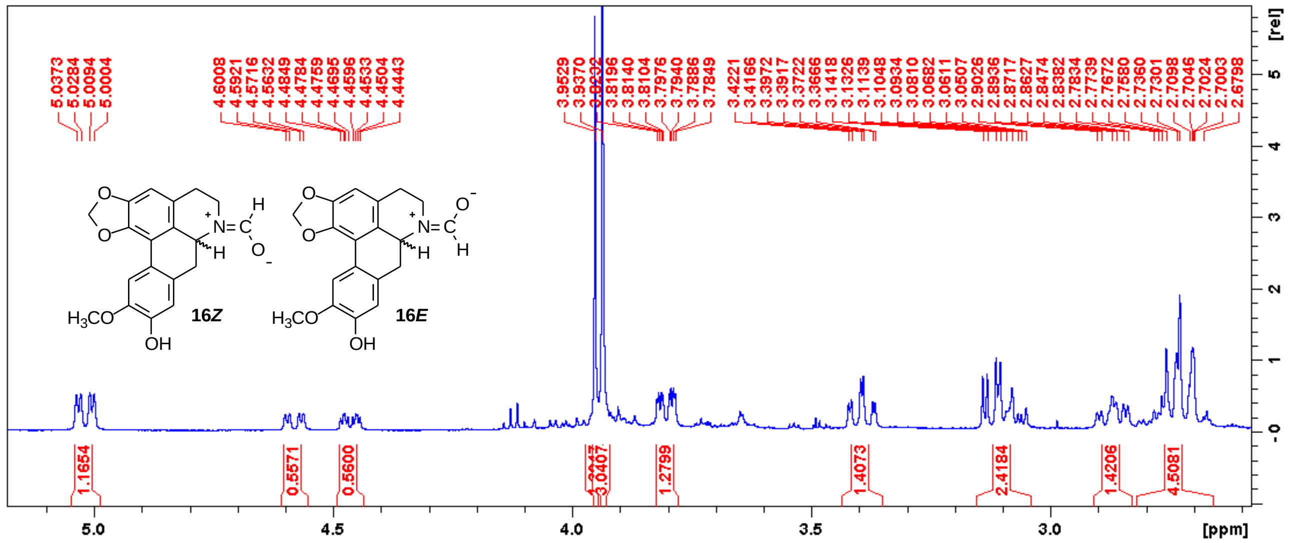

Figure S180. Expanded view 5 of the aliphatic proton region in the ^{1}H NMR spectrum of **(*Z*/*E*)-*N*-formylactinodaphnine** (**16**) recorded in CDCl₃ at 500 MHz. Where duplicated resonances are resolved, the signal sets should be interpreted together with the major:minor assignment defined from the formyl signals in Figure S175.

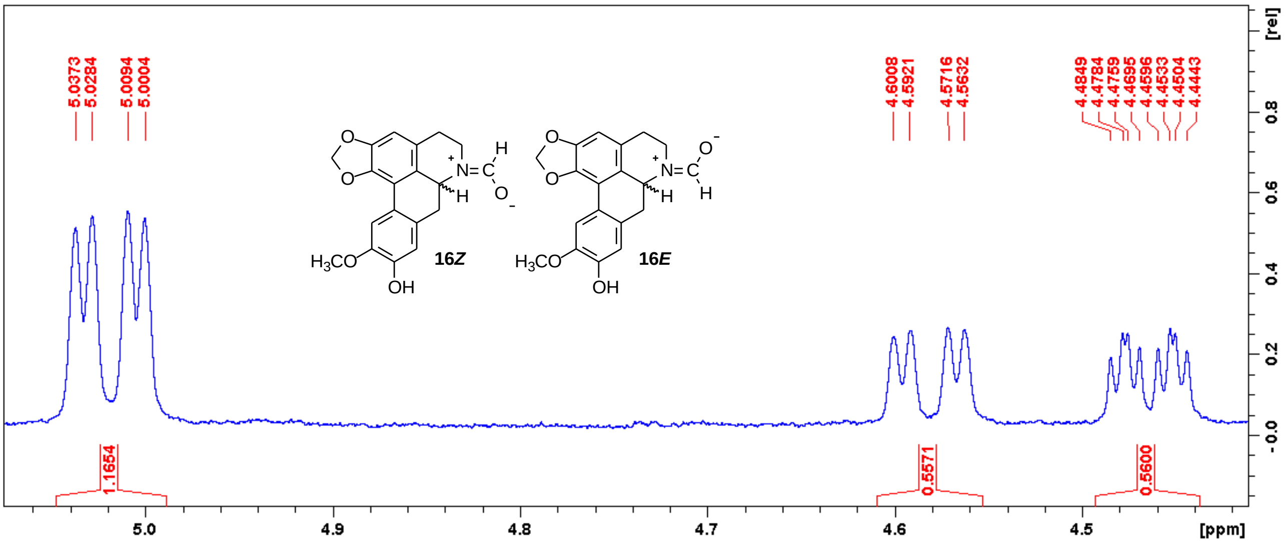

Figure S181. Expanded view 6 of the aliphatic proton region in the ^{1}H NMR spectrum of **(*Z*/*E*)-*N*-formylactinodaphnine** (**16**) recorded in $CDCl_3$ at 500 MHz. Where duplicated resonances are resolved, the signal sets should be interpreted together with the major:minor assignment defined from the formyl signals in Figure S175.

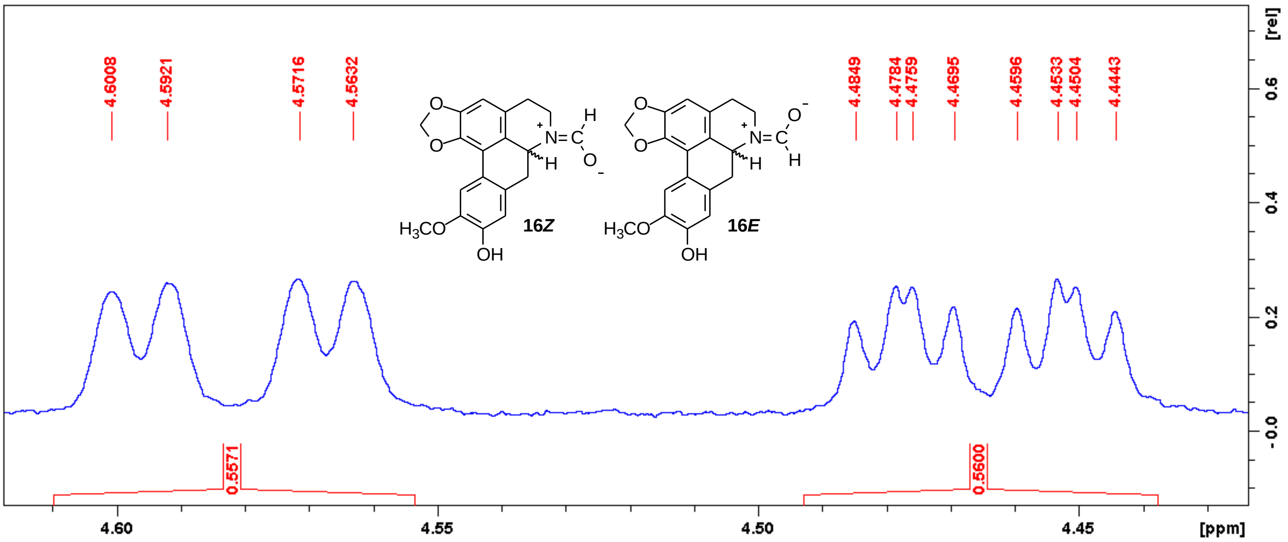

Figure S182. Expanded view 7 of the aliphatic proton region in the ^{1}H NMR spectrum of **(*Z*/*E*)-*N*-formylactinodaphnine** (**16**) recorded in $CDCl_3$ at 500 MHz. Where duplicated resonances are resolved, the signal sets should be interpreted together with the major:minor assignment defined from the formyl signals in Figure S175.

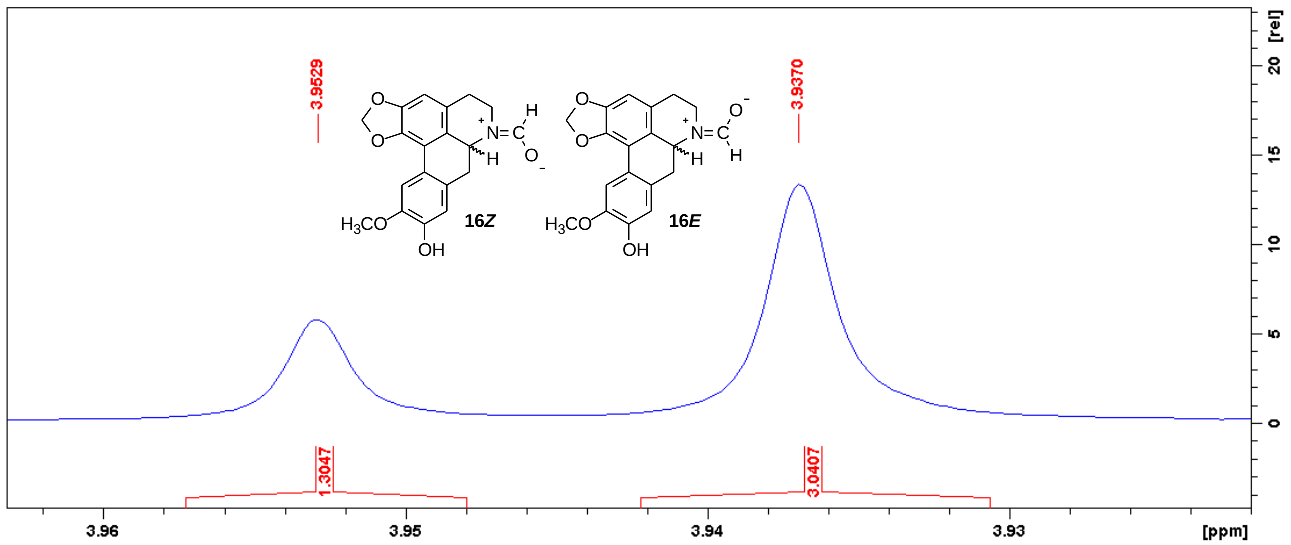

Figure S183. Expanded view 8 of the aliphatic proton region in the ^{1}H NMR spectrum of **(*Z*/*E*)-*N*-formylactinodaphnine** (**16**) recorded in $CDCl_3$ at 500 MHz. Where duplicated resonances are resolved, the signal sets should be interpreted together with the major:minor assignment defined from the formyl signals in Figure S175.

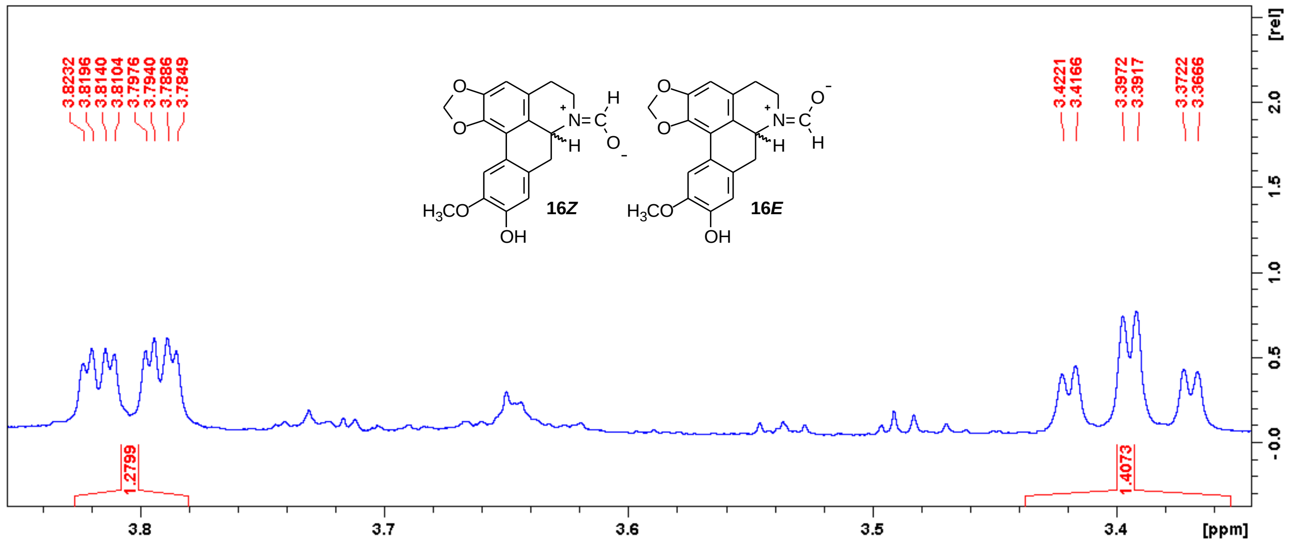

Figure S184. Expanded view 9 of the aliphatic proton region in the ^{1}H NMR spectrum of **(*Z*/*E*)-*N*-formylactinodaphnine** (**16**) recorded in $CDCl_3$ at 500 MHz. Where duplicated resonances are resolved, the signal sets should be interpreted together with the major:minor assignment defined from the formyl signals in Figure S175.

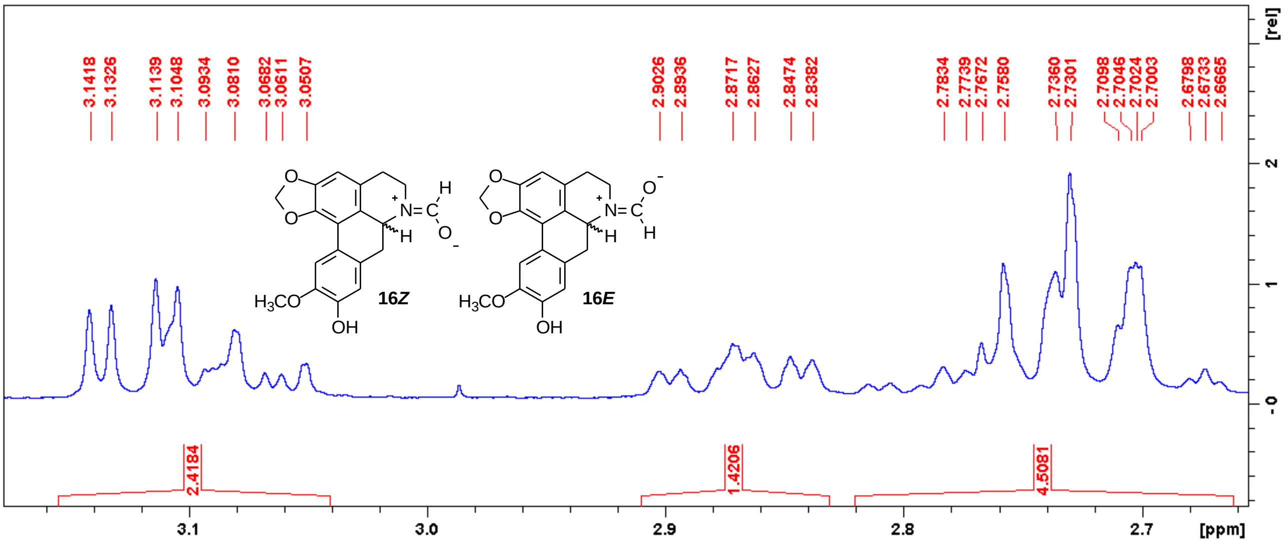

Figure S185. Expanded view 10 of the aliphatic proton region in the ^{1}H NMR spectrum of **(*Z*/*E*)-*N*-formylactinodaphnine** (**16**) recorded in $CDCl_3$ at 500 MHz. Where duplicated resonances are resolved, the signal sets should be interpreted together with the major:minor assignment defined from the formyl signals in Figure S175.

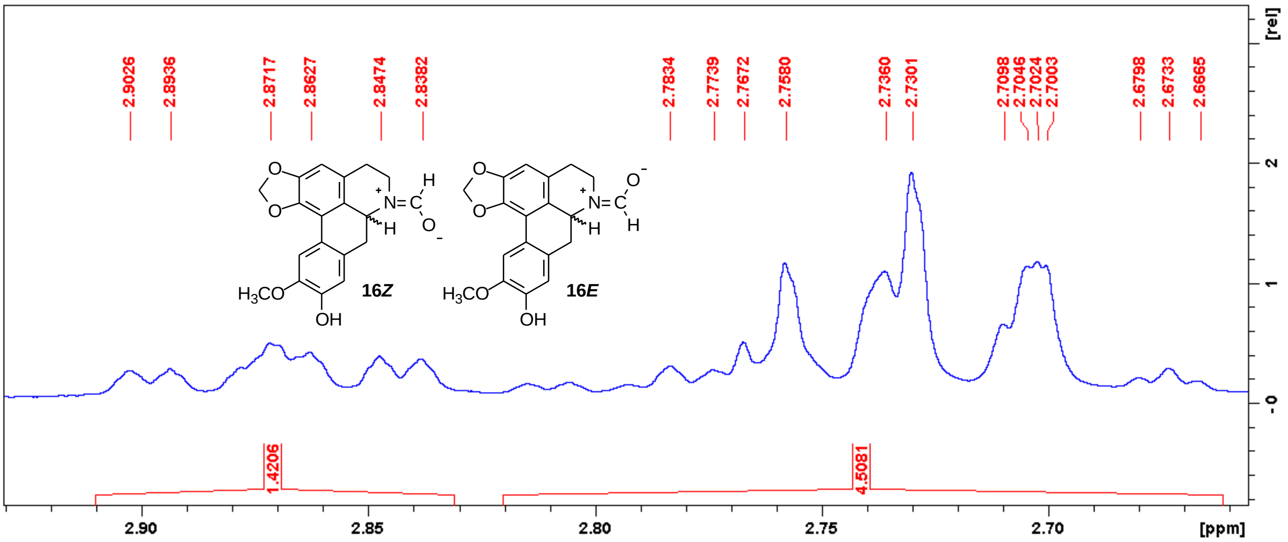

Figure S186. Expanded view 11 of the aliphatic proton region in the 1H NMR spectrum of **(*Z*/*E*)-*N*-formylactinodaphnine** (**16**) recorded in $CDCl_3$ at 500 MHz. Where duplicated resonances are resolved, the signal sets should be interpreted together with the major:minor assignment defined from the formyl signals in Figure S175.

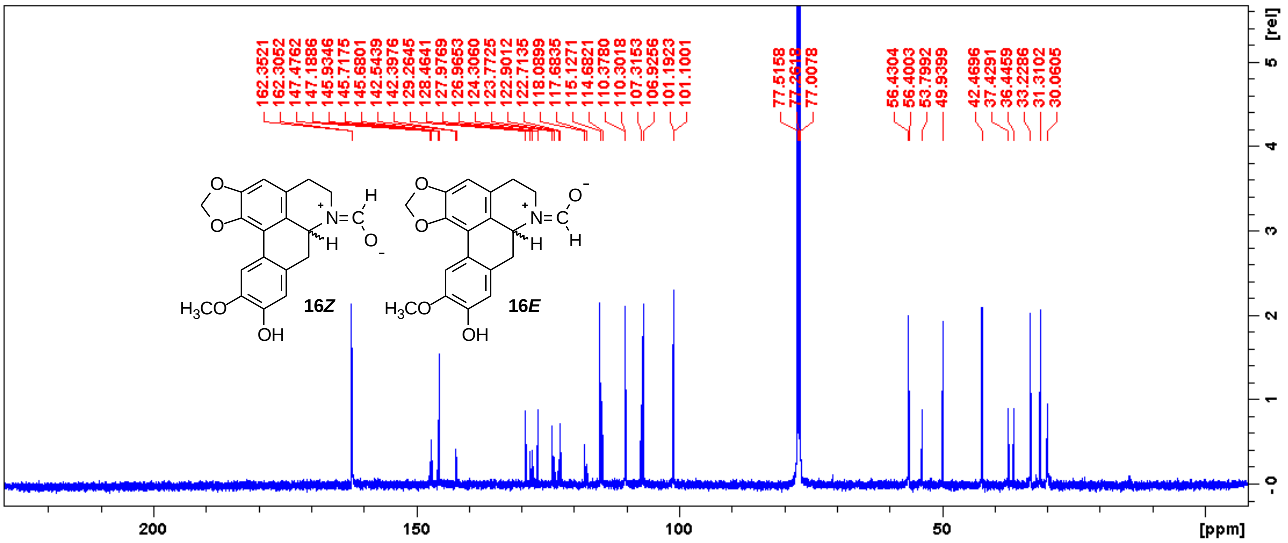

Figure S187. Full ^{13}C NMR spectrum of **(*Z*/*E*)-*N*-formylactinodaphnine** (**16**) recorded in $CDCl_3$ at 125 MHz.

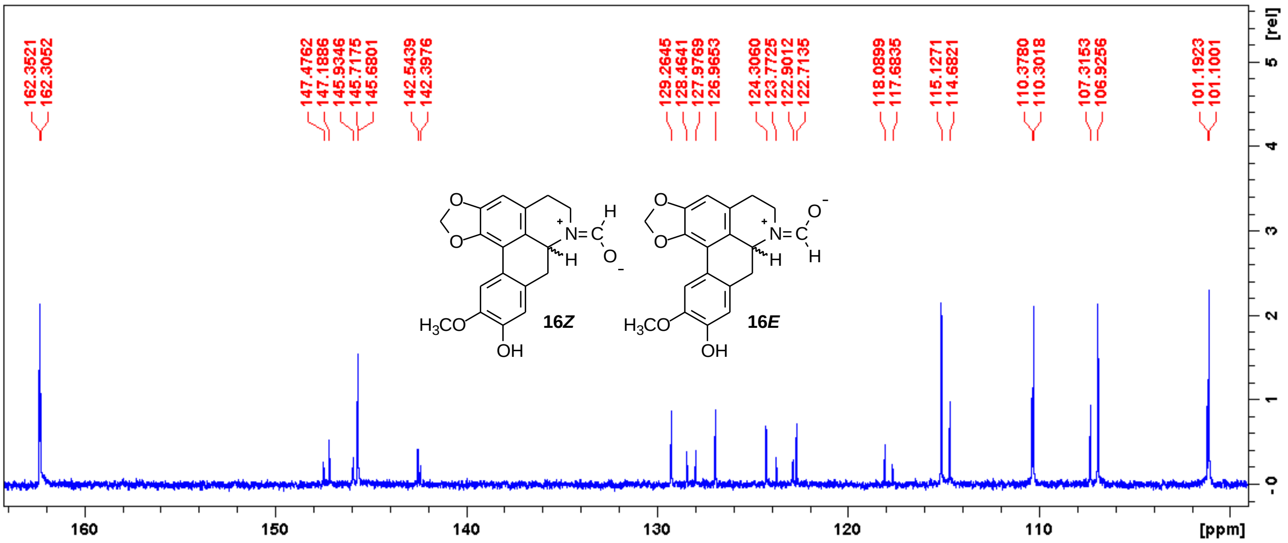

Figure S188. Expanded view of the carbon region (δ_C 164.1–99.2 ppm) in the ^{13}C NMR spectrum of **(*Z*/*E*)-*N*-formylactinodaphnine** (**16**) recorded in $CDCl_3$ at 125 MHz.

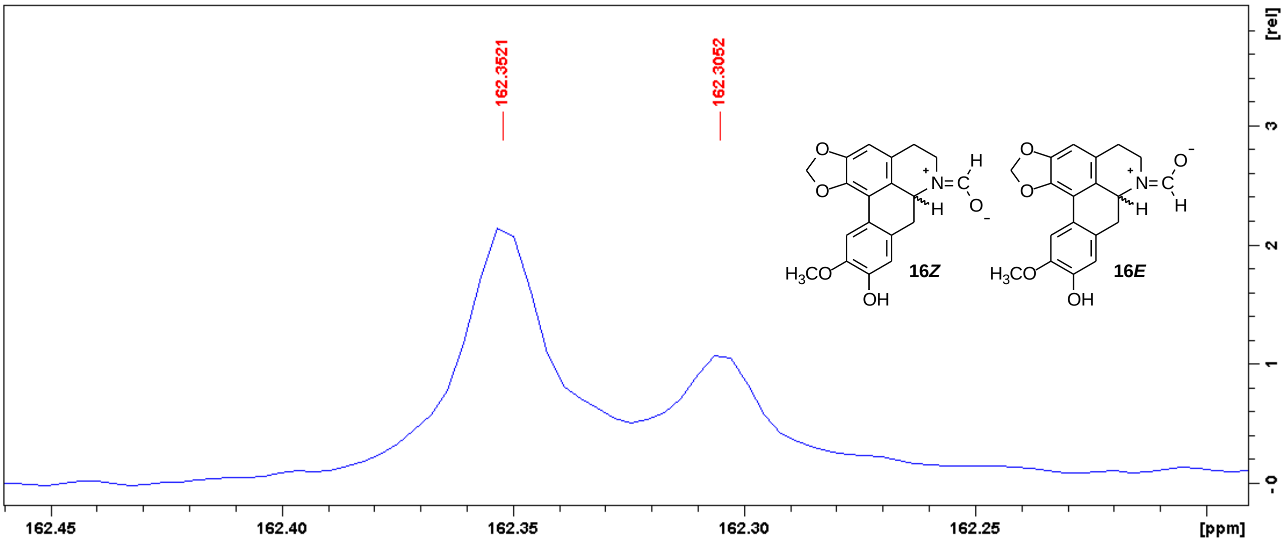

Figure S189. Expanded view of the carbon region (δ_C 164.4–162.1 ppm) in the ^{13}C NMR spectrum of **(*Z*/*E*)-*N*-formylactinodaphnine** (**16**) recorded in $CDCl_3$ at 125 MHz.

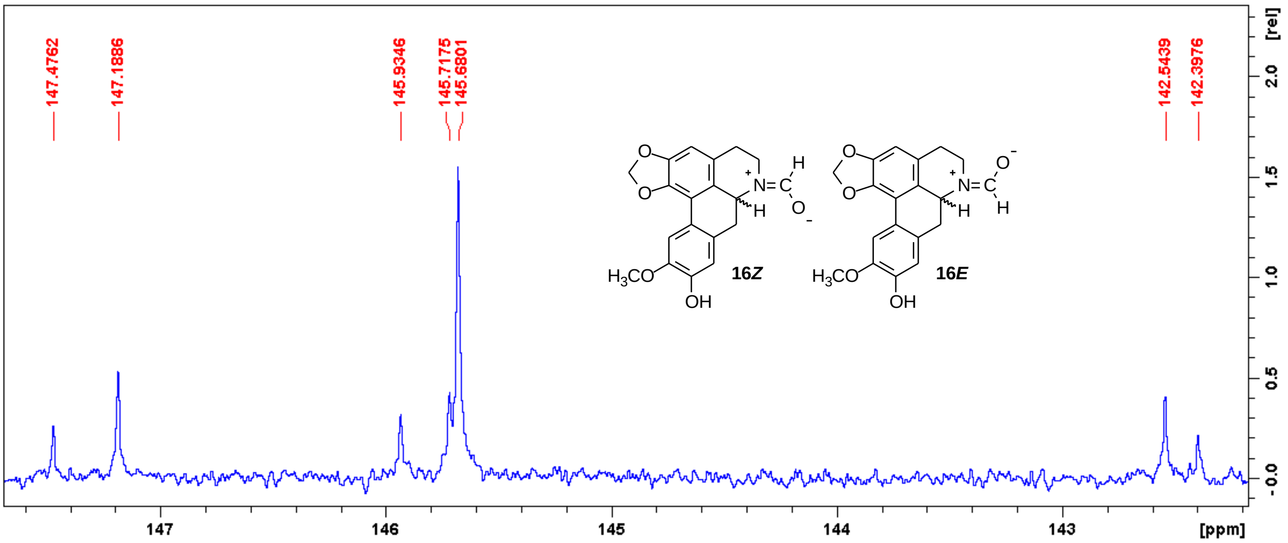

Figure S190. Expanded view of the carbon region (δ_C 147.6–142.2 ppm) in the ^{13}C NMR spectrum of ***(Z*/*E)*-*N*-formylactinodaphnine (16)** recorded in $CDCl_3$ at 125 MHz.

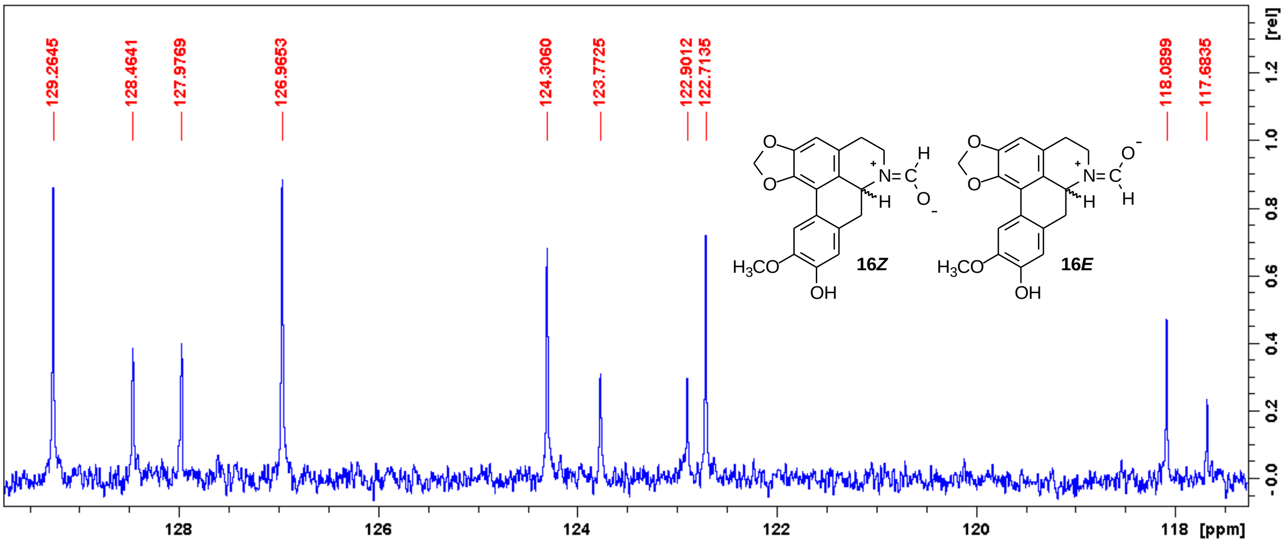

Figure S191. Expanded view of the carbon region (δ_C 129.7–117.3 ppm) in the ^{13}C NMR spectrum of ***(Z*/*E)*-*N*-formylactinodaphnine (16)** recorded in $CDCl_3$ at 125 MHz.

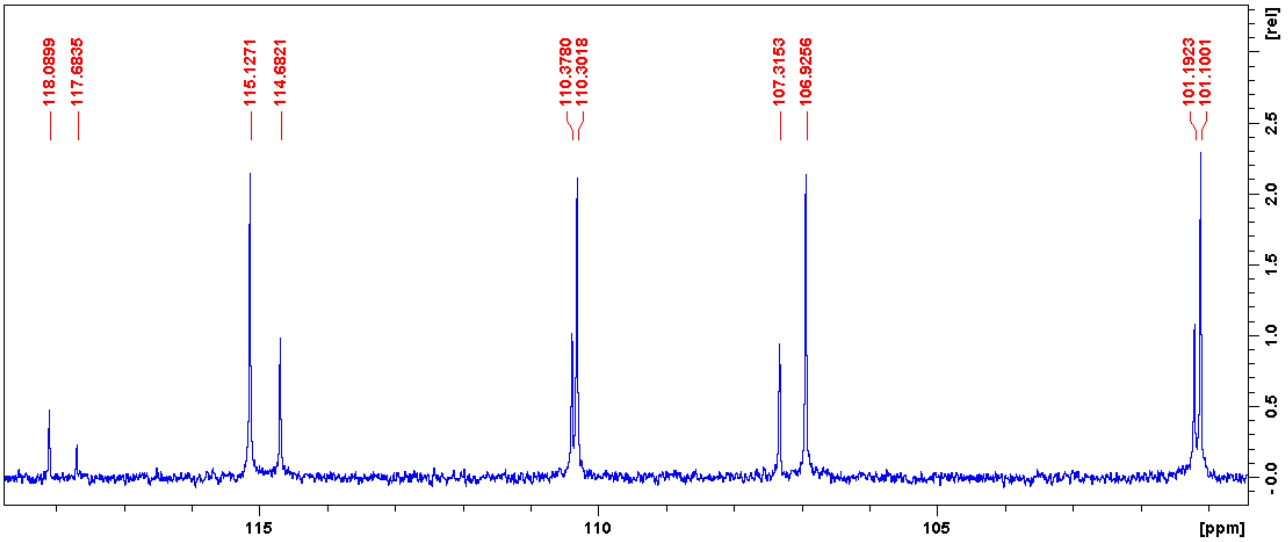

Figure S192. Expanded view of the carbon region (δ_C 118.7–100.5 ppm) in the ^{13}C NMR spectrum of ***(Z*/*E)*-*N*-formylactinodaphnine (16)** recorded in $CDCl_3$ at 125 MHz.

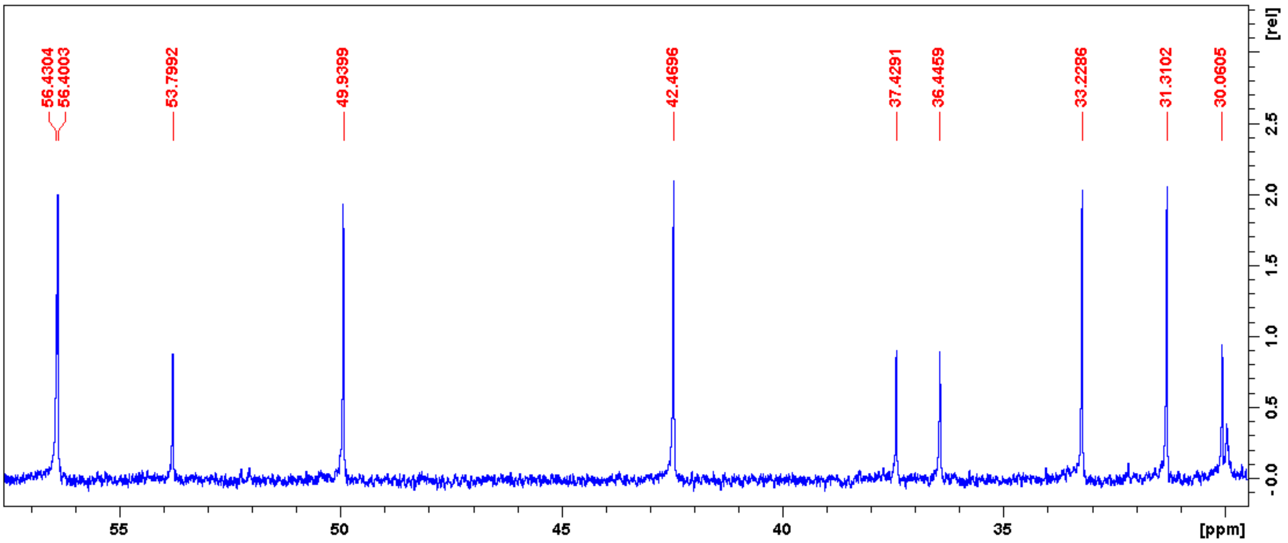

Figure S193. Expanded view of the carbon region (δ_C 57.4–30.0 ppm) in the ^{13}C NMR spectrum of **(*Z*/*E*)-*N*-formylactinodaphnine** (**16**) recorded in $CDCl_3$ at 125 MHz.

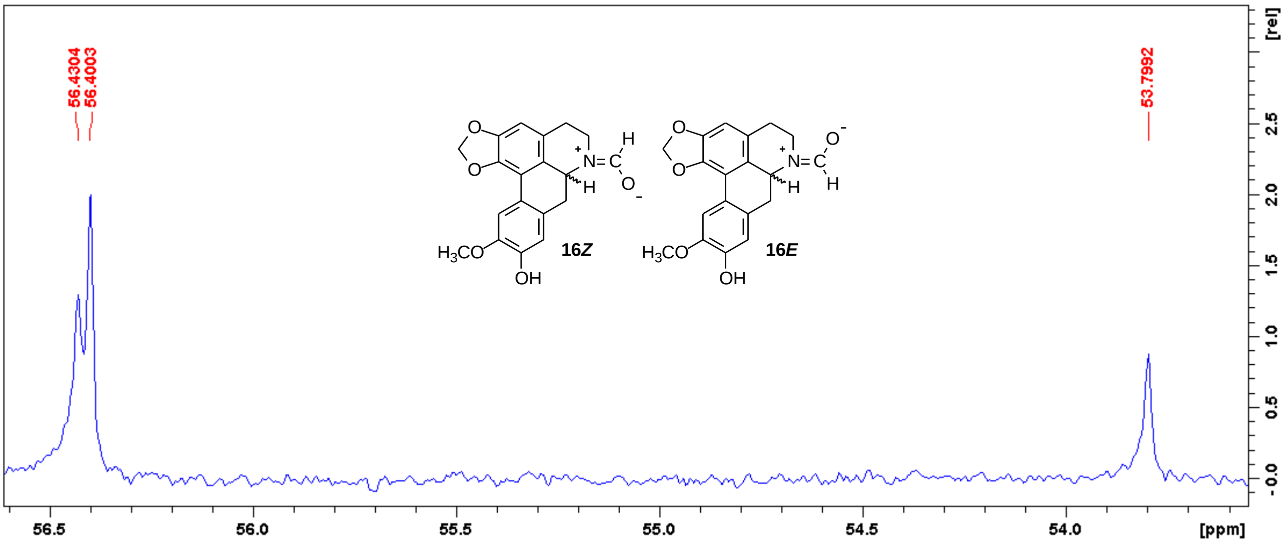

Figure S194. Expanded view of the carbon region (δ_C 56.6–53.5 ppm) in the ^{13}C NMR spectrum of **(*Z*/*E*)-*N*-formylactinodaphnine** (**16**) recorded in $CDCl_3$ at 125 MHz.

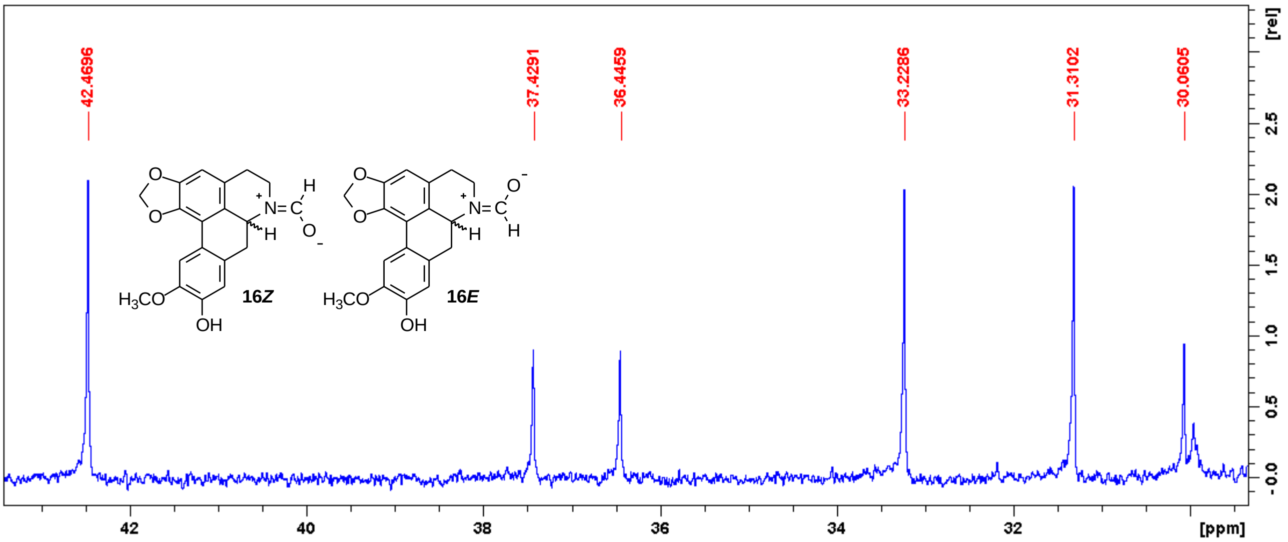

Figure S195. Expanded view of the carbon region (δ_C 43.4–29.4 ppm) in the ^{13}C NMR spectrum of **(*Z*/*E*)-*N*-formylactinodaphnine** (**16**) recorded in $CDCl_3$ at 125 MHz.

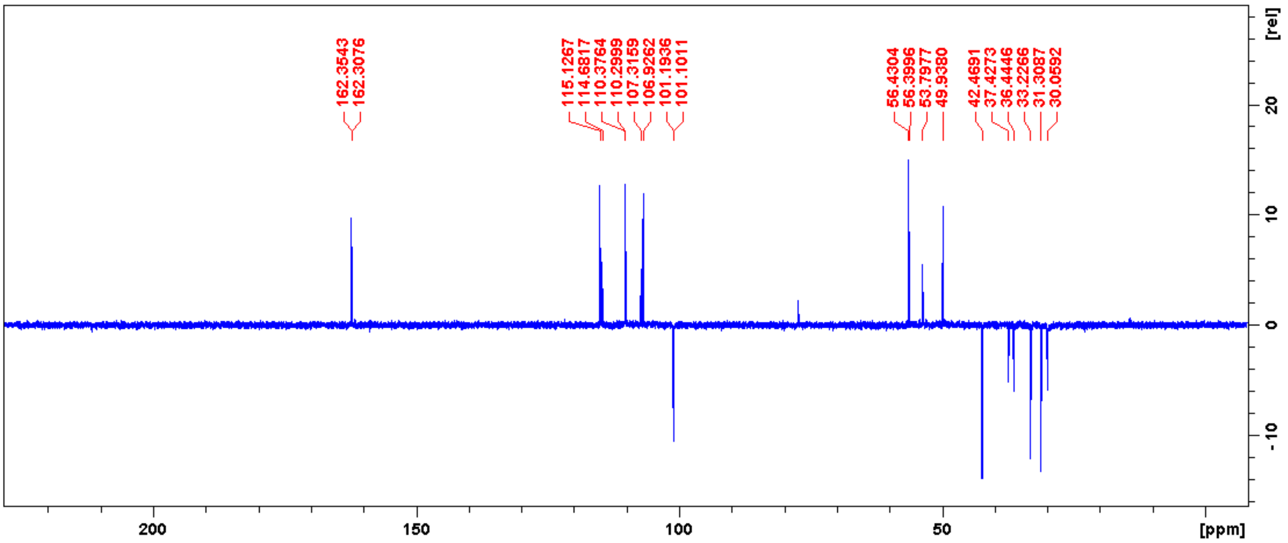

Figure S196. Full DEPT-135 ^{13}C NMR spectrum of **(*Z*/*E*)-*N*-formylactinodaphnine** (**16**) recorded in $CDCl_3$ at 125 MHz.

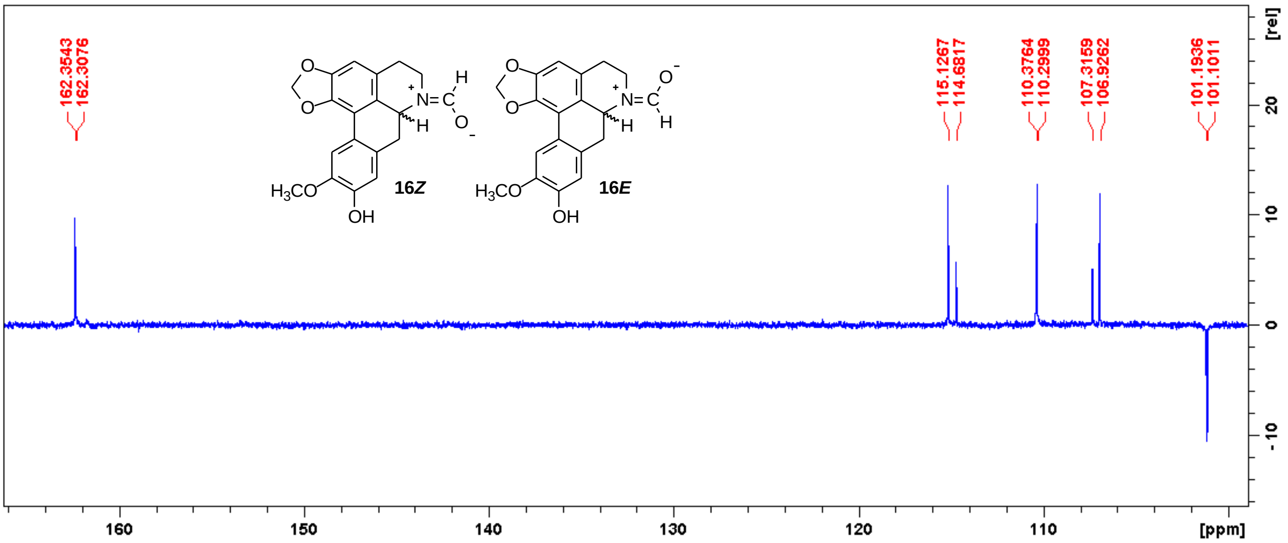

Figure S197. Expanded view of the carbon region (δ_C 166.2–99.3 ppm) in the DEPT-135 ^{13}C NMR spectrum of **(*Z*/*E*)-*N*-formylactinodaphnine** (**16**) recorded in $CDCl_3$ at 125 MHz.

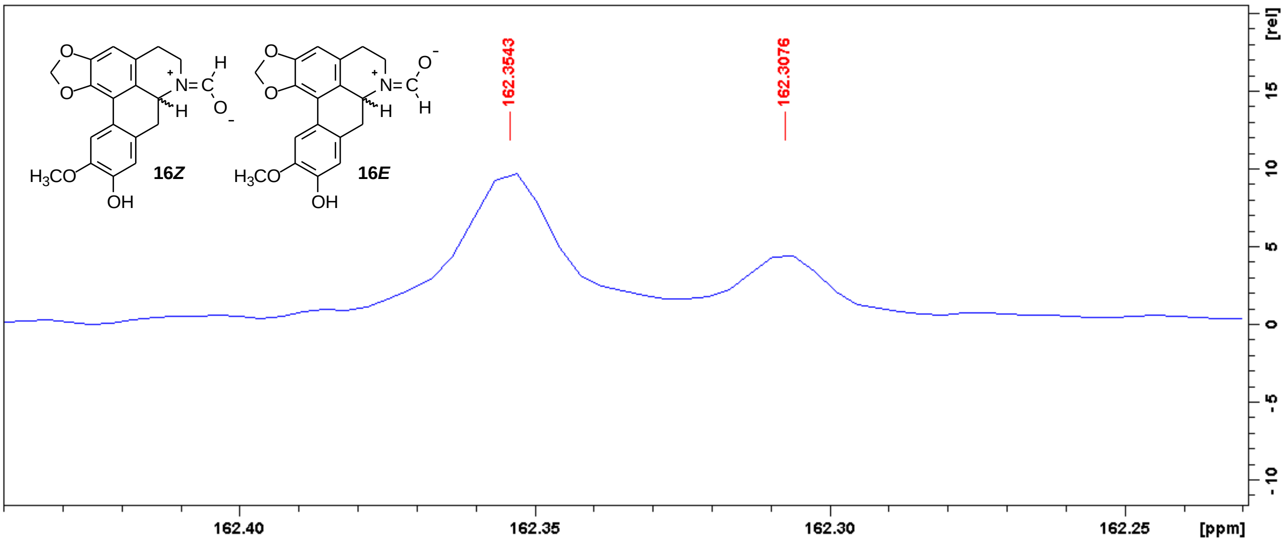

Figure S198. Expanded view of the carbon region (δ_C 166.4–162.2 ppm) in the DEPT-135 ^{13}C NMR spectrum of **(*Z*/*E*)-*N*-formylactinodaphnine** (**16**) recorded in $CDCl_3$ at 125 MHz.

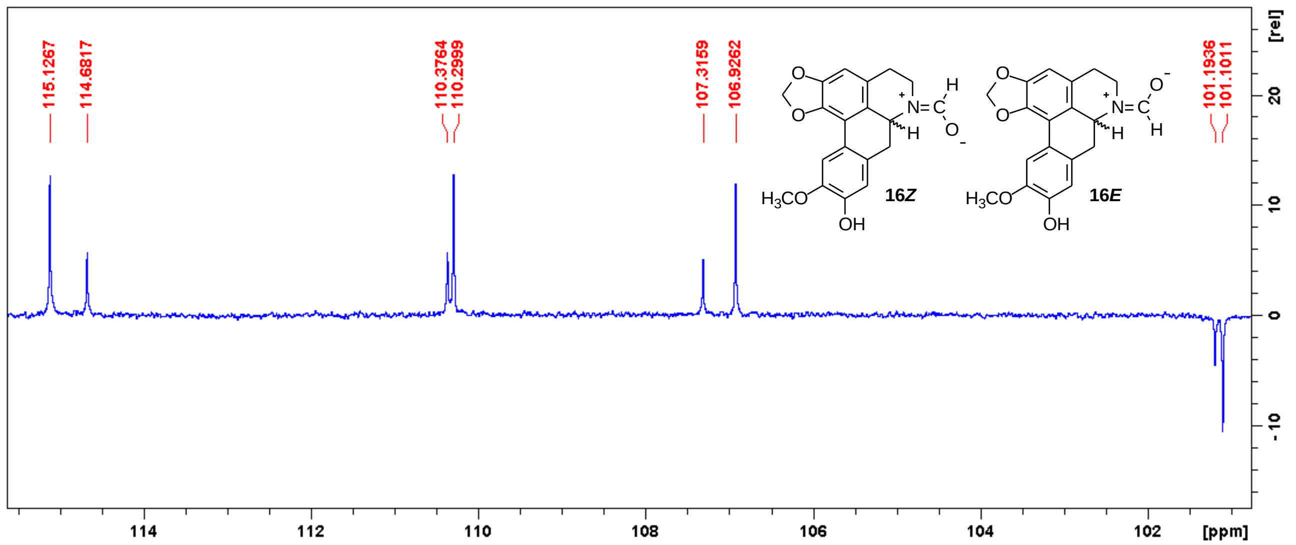

Figure S199. Expanded view of the carbon region (δ_C 115.6–100.8 ppm) in the DEPT-135 ^{13}C NMR spectrum of ***(Z/E)-N*-formylactinodaphnine** (**16**) recorded in $CDCl_3$ at 125 MHz.

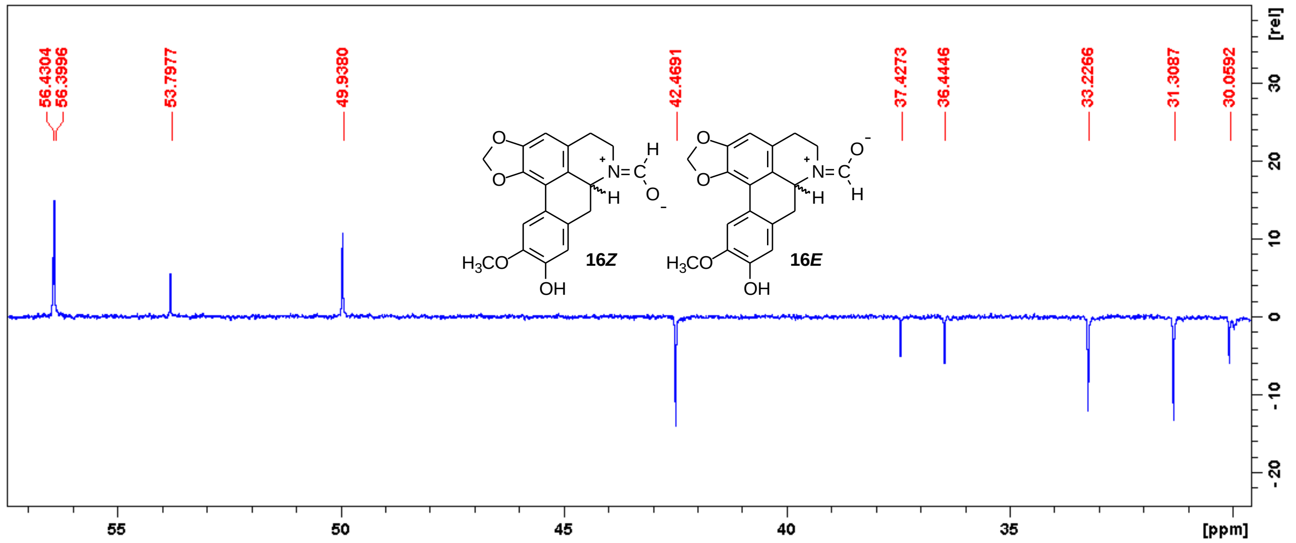

Figure S200. Expanded view of the carbon region (δ_C 57.4–29.6 ppm) in the DEPT-135 ^{13}C NMR spectrum of ***(Z/E)-N*-formylactinodaphnine** (**16**) recorded in $CDCl_3$ at 125 MHz.

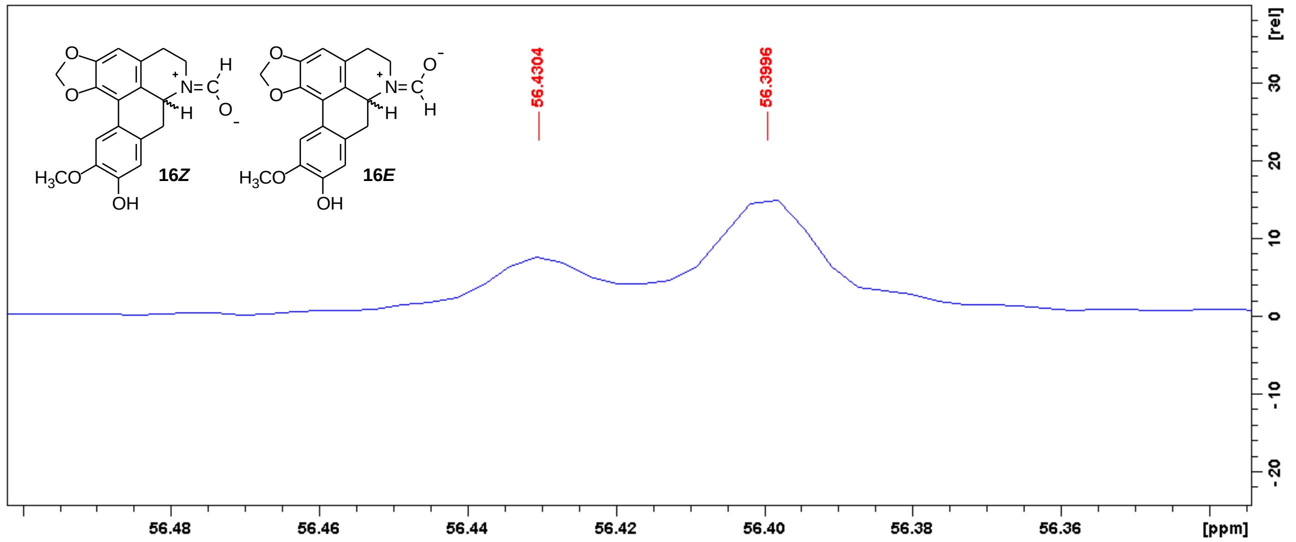

Figure S201. Expanded view of the carbon region (δ_C 56.5–53.3 ppm) in the DEPT-135 ^{13}C NMR spectrum of ***(Z/E)-N*-formylactinodaphnine** (**16**) recorded in $CDCl_3$ at 125 MHz.

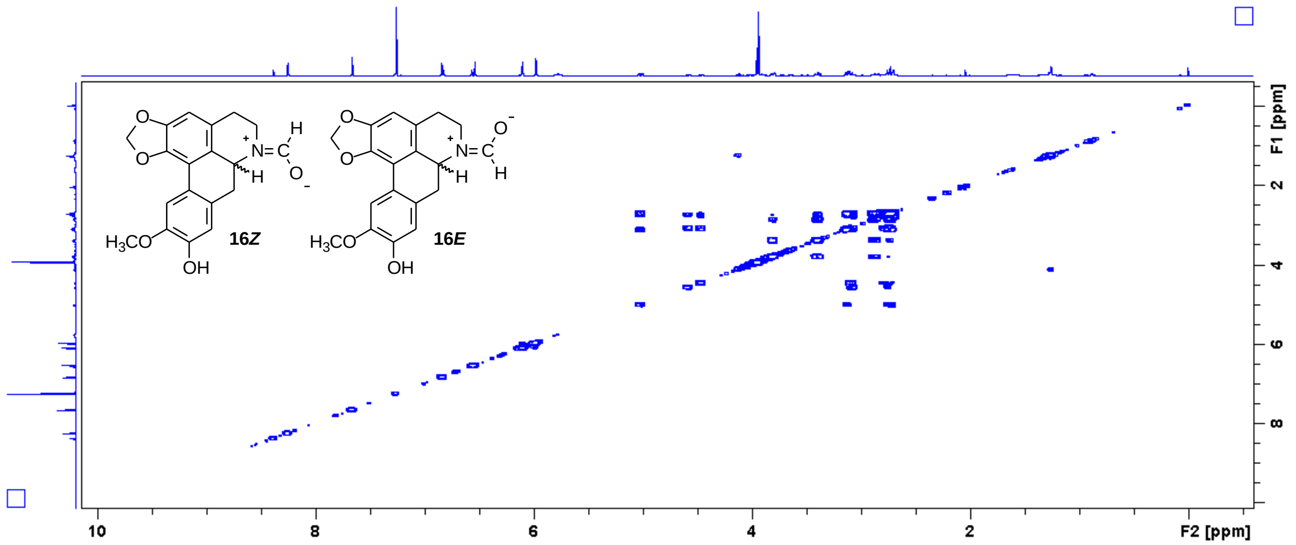

Figure S202. ^{1}H–^{1}H COSY correlation map **(*Z*/*E*)-*N*-formylactinodaphnine** (**16**) recorded in $CDCl_3$ at 500 MHz.

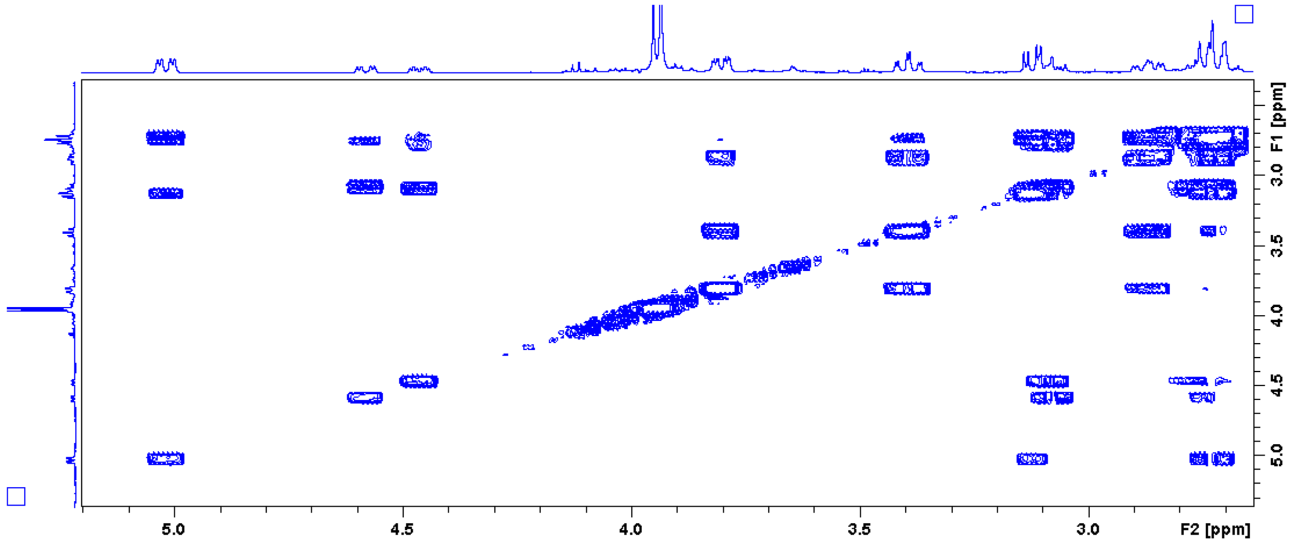

Figure S203. Expanded view 1 of the ^{1}H–^{1}H COSY correlation map of **(*Z*/*E*)-*N*-formylactinodaphnine** (**16**) recorded in $CDCl_3$ at 500 MHz.

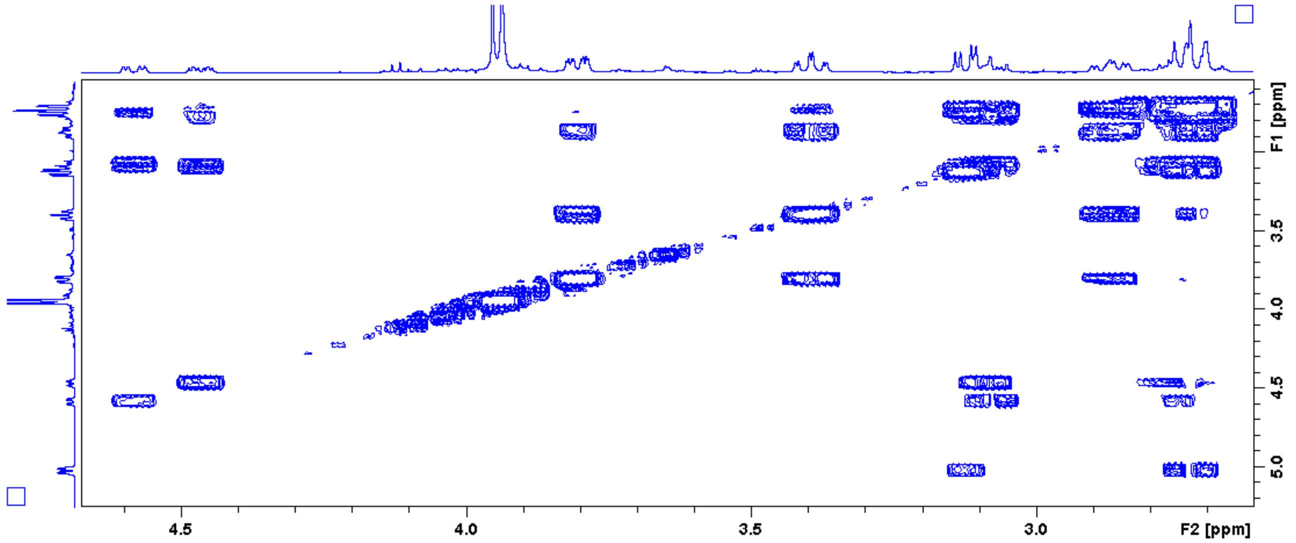

Figure S204. Expanded view 2 of the ^{1}H–^{1}H COSY correlation map of **(*Z*/*E*)-*N*-formylactinodaphnine** (**16**) recorded in $CDCl_3$ at 500 MHz.

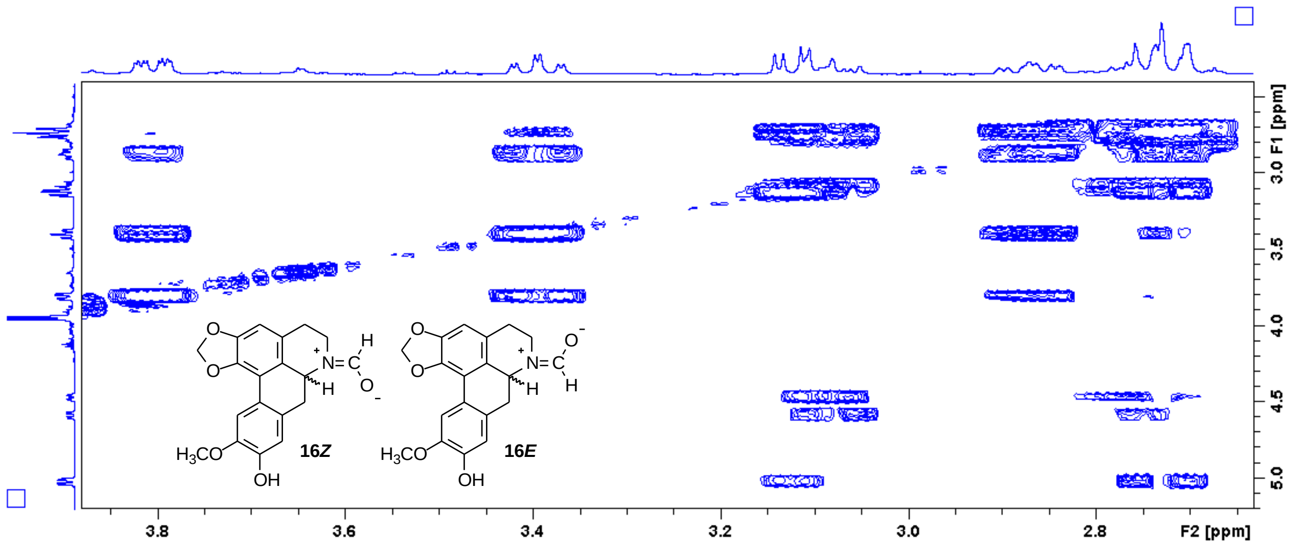

Figure S205. Expanded view 3 of the ^{1}H–^{1}H COSY correlation map of ***(Z/E)-N*-formylactinodaphnine (16)** recorded in $CDCl_3$ at 500 MHz.

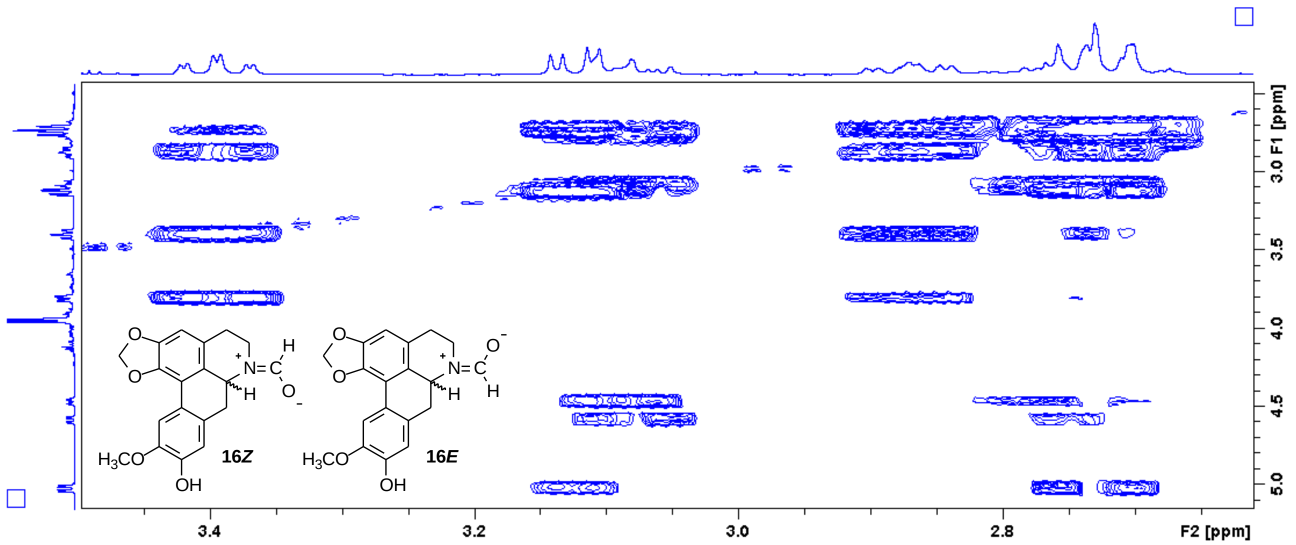

Figure S206. Expanded view 4 of the ^{1}H–^{1}H COSY correlation map of ***(Z/E)-N*-formylactinodaphnine (16)** recorded in $CDCl_3$ at 500 MHz.

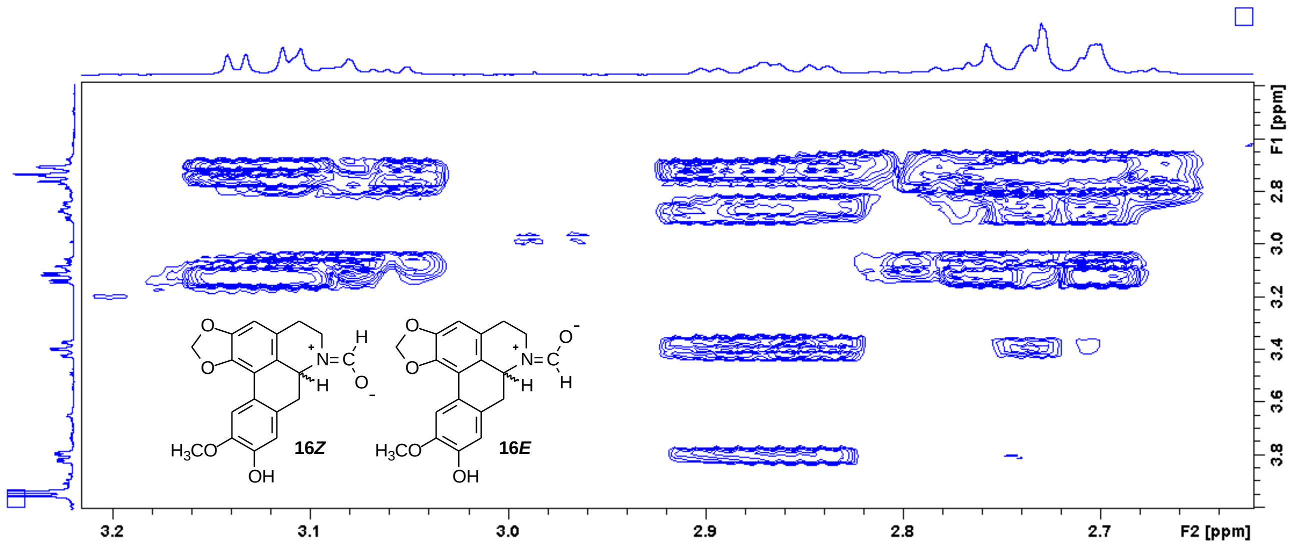

Figure S207. Expanded view 5 of the ^{1}H–^{1}H COSY correlation map of ***(Z/E)-N*-formylactinodaphnine (16)** recorded in $CDCl_3$ at 500 MHz.

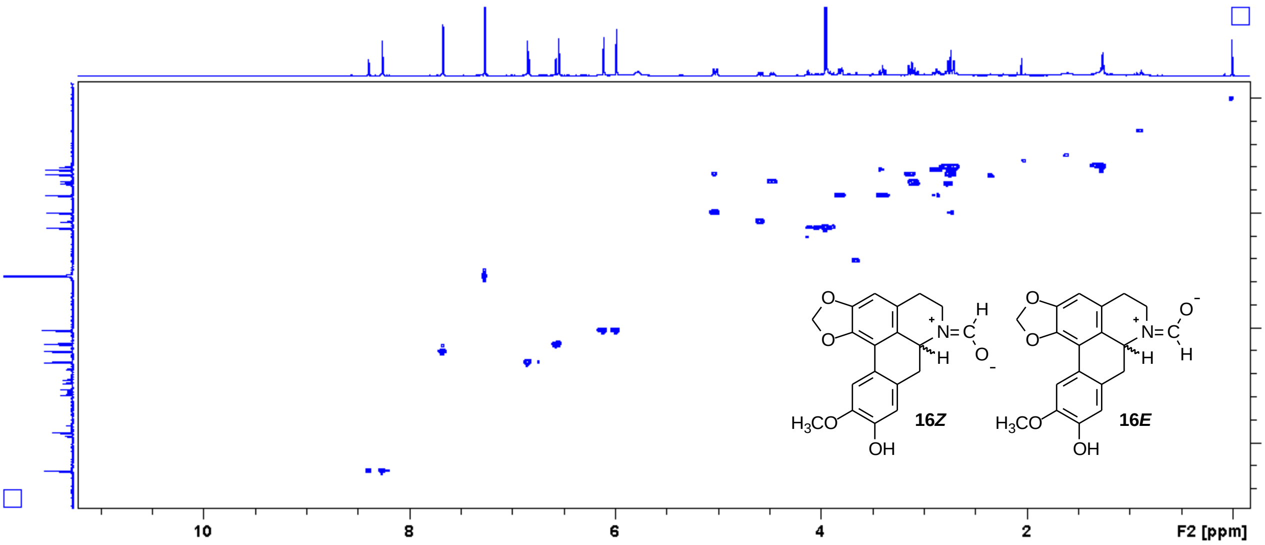

Figure S208. One-bond ^{1}H–^{13}C HSQC correlation map of **(*Z*/*E*)-*N*-formylactinodaphnine** (**16**) recorded in CDCl at 500 MHz (^{1}H) and 125 MHz (^{13}C).

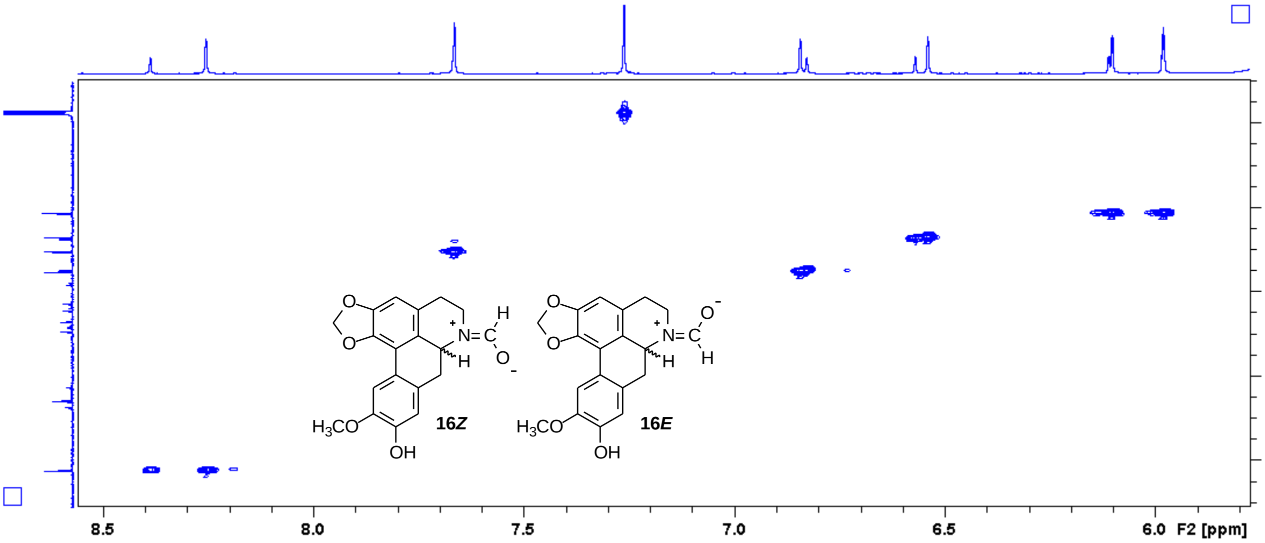

Figure S209. Expanded view 1 of the one-bond ^{1}H–^{13}C HSQC correlation map of **(*Z*/*E*)-*N*-formylactinodaphnine** (**16**) recorded in $CDCl_3$ at 500 MHz (^{1}H) and 125 MHz (^{13}C).

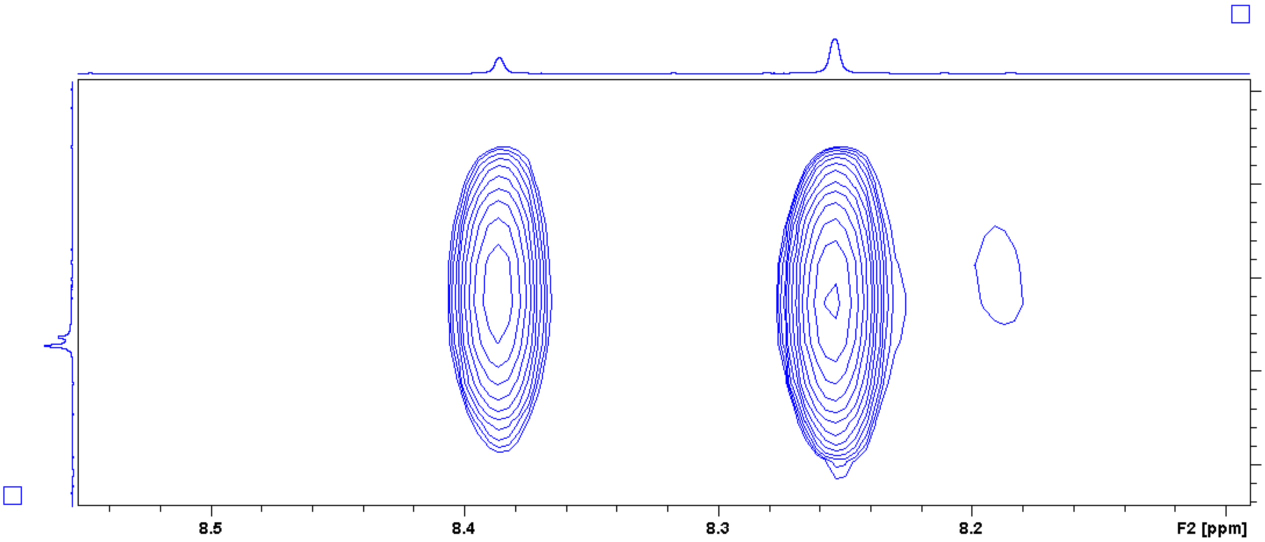

Figure S210. Expanded view 2 of the one-bond ^{1}H–^{13}C HSQC correlation map of **(*Z*/*E*)-*N*-formylactinodaphnine** (**16**) recorded in $CDCl_3$ at 500 MHz (^{1}H) and 125 MHz (^{13}C).

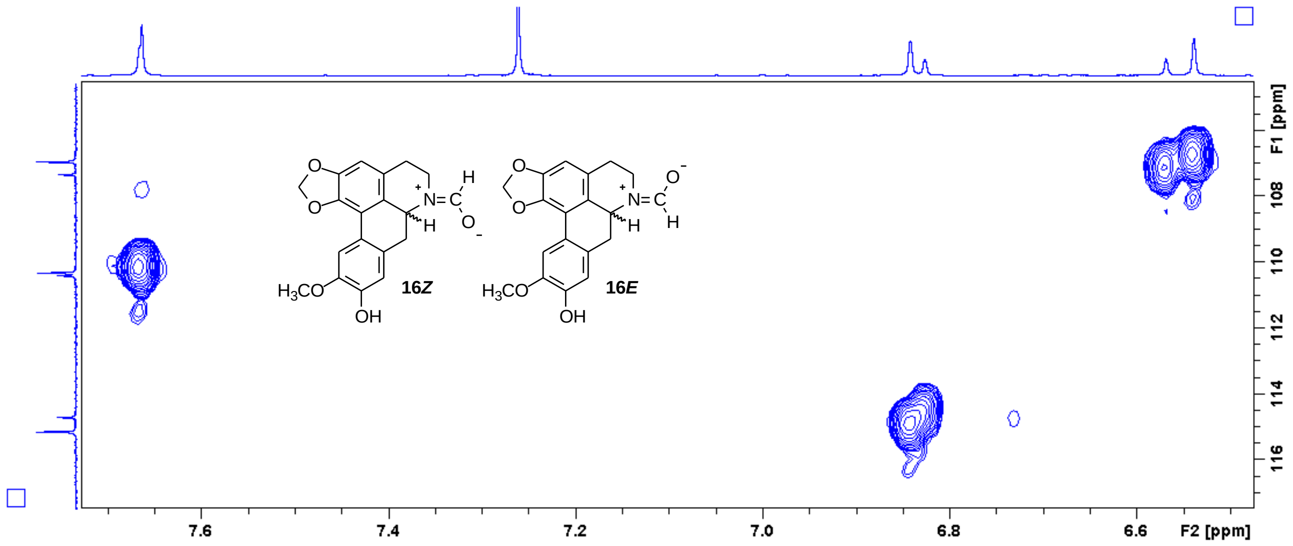

Figure S211. Expanded view 3 of the one-bond ^{1}H–^{13}C HSQC correlation map of **(*Z*/*E*)-*N*-formylactinodaphnine** (**16**) recorded in $CDCl_3$ at 500 MHz (^{1}H) and 125 MHz (^{13}C).

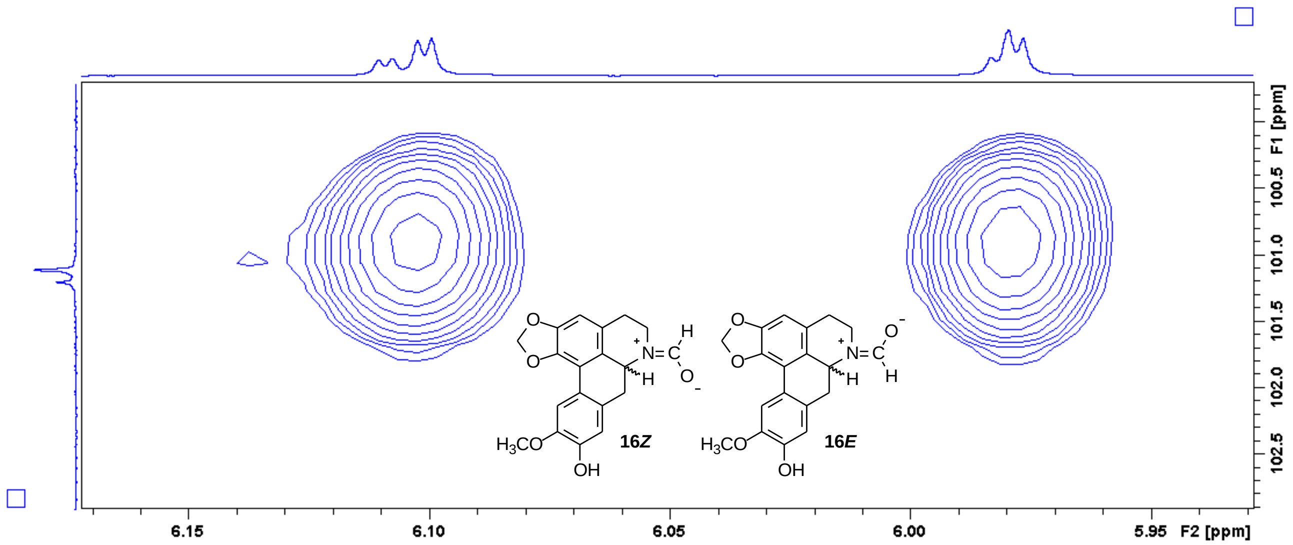

Figure S212. Expanded view 4 of the one-bond ^{1}H–^{13}C HSQC correlation map of **(*Z*/*E*)-*N*-formylactinodaphnine** (**16**) recorded in $CDCl_3$ at 500 MHz (^{1}H) and 125 MHz (^{13}C).

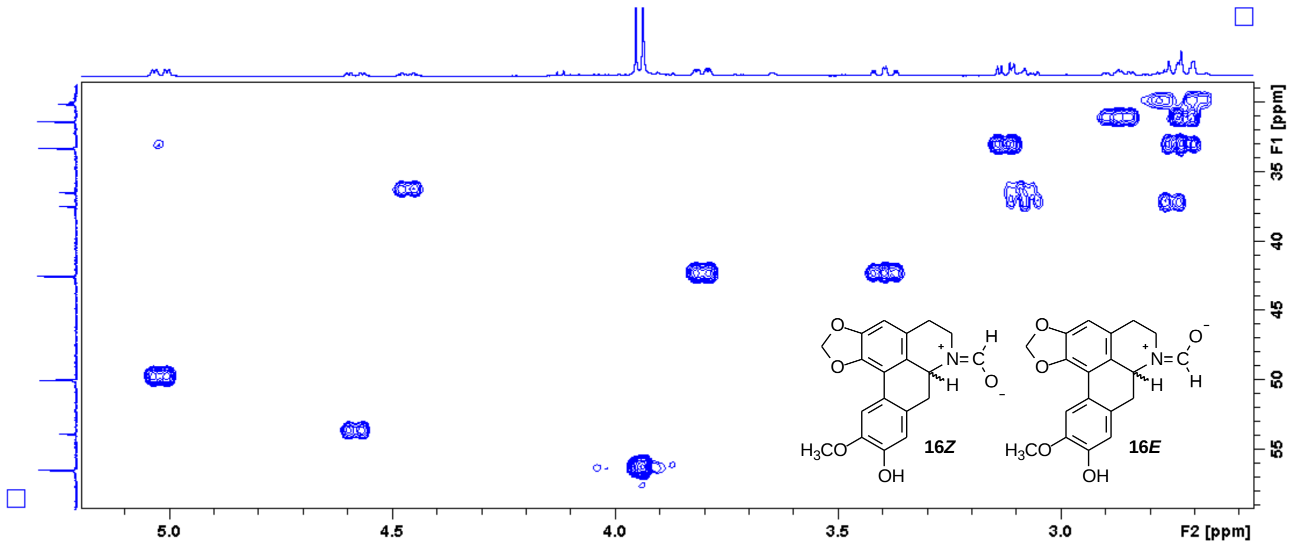

Figure S213. Expanded view 5 of the one-bond ^{1}H–^{13}C HSQC correlation map of **(*Z*/*E*)-*N*-formylactinodaphnine** (**16**) recorded in $CDCl_3$ at 500 MHz (^{1}H) and 125 MHz (^{13}C).

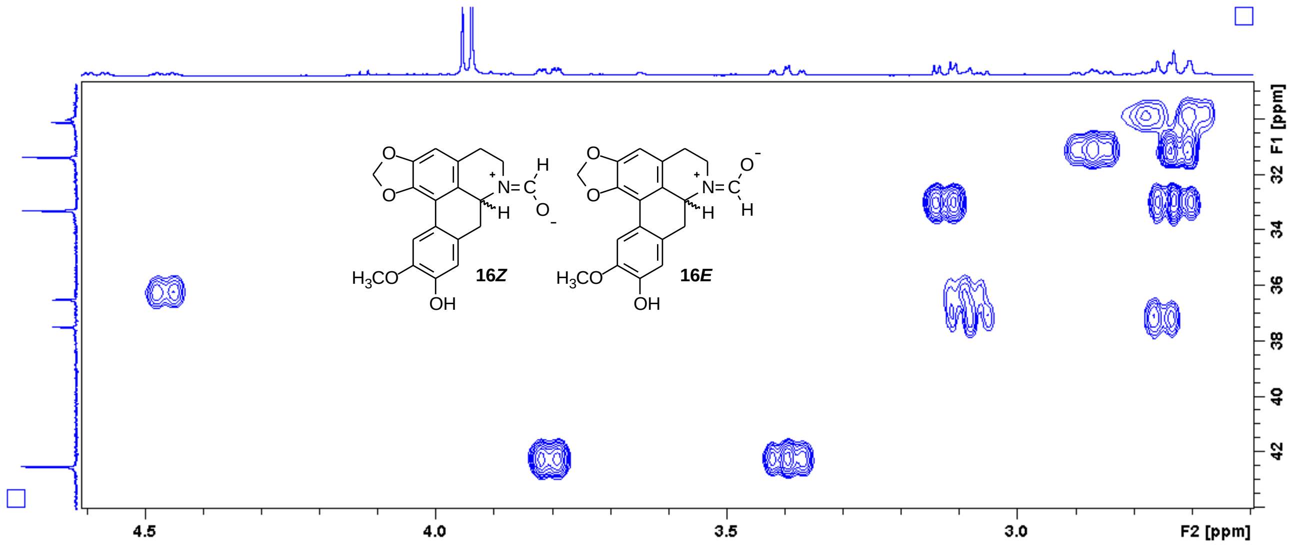

Figure S214. Expanded view 6 of the one-bond ^{1}H–^{13}C HSQC correlation map of **(*Z*/*E*)-*N*-formylactinodaphnine** (**16**) recorded in $CDCl_3$ at 500 MHz (^{1}H) and 125 MHz (^{13}C).

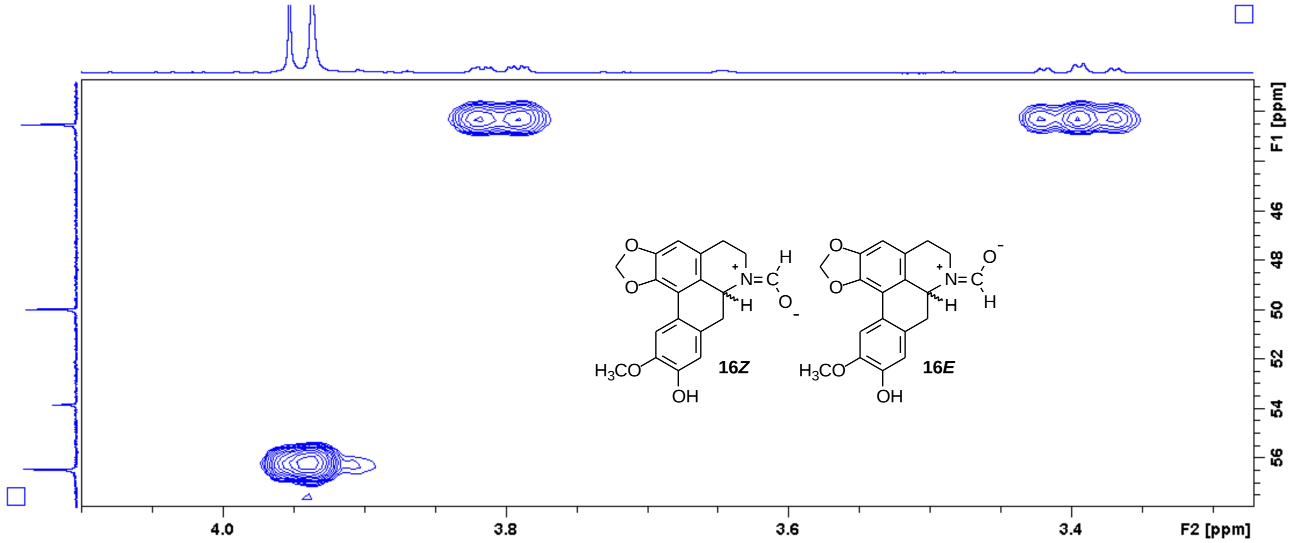

Figure S215. Expanded view 7 of the one-bond ^{1}H–^{13}C HSQC correlation map of **(*Z*/*E*)-*N*-formylactinodaphnine** (**16**) recorded in $CDCl_3$ at 500 MHz (^{1}H) and 125 MHz (^{13}C).

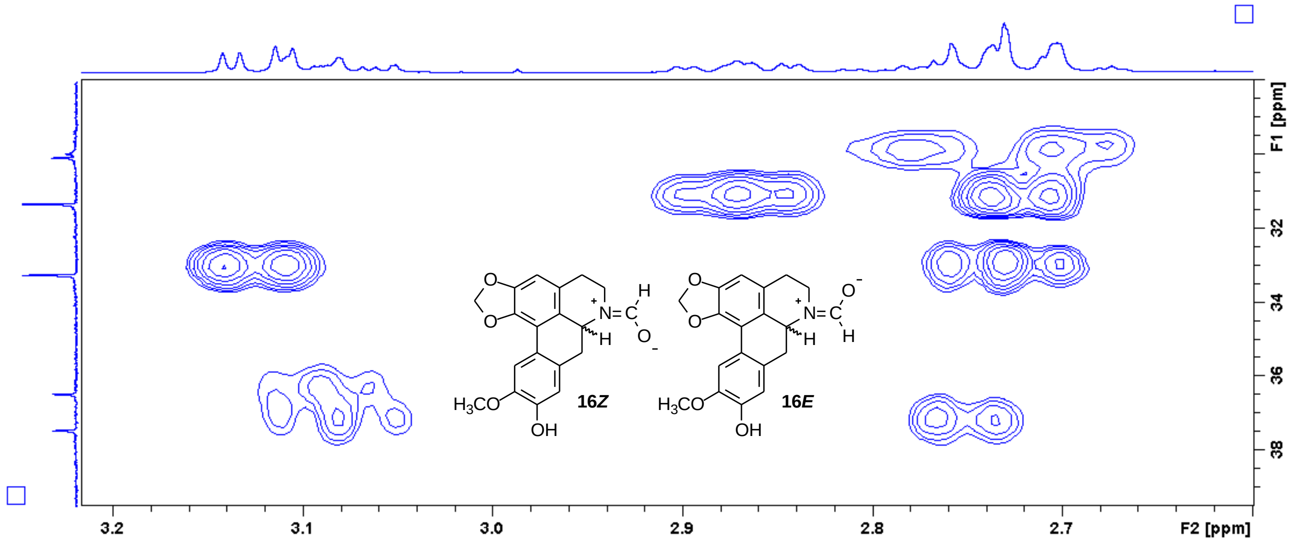

Figure S216. Expanded view 8 of the one-bond ^{1}H–^{13}C HSQC correlation map of **(*Z*/*E*)-*N*-formylactinodaphnine** (**16**) recorded in $CDCl_3$ at 500 MHz (^{1}H) and 125 MHz (^{13}C).

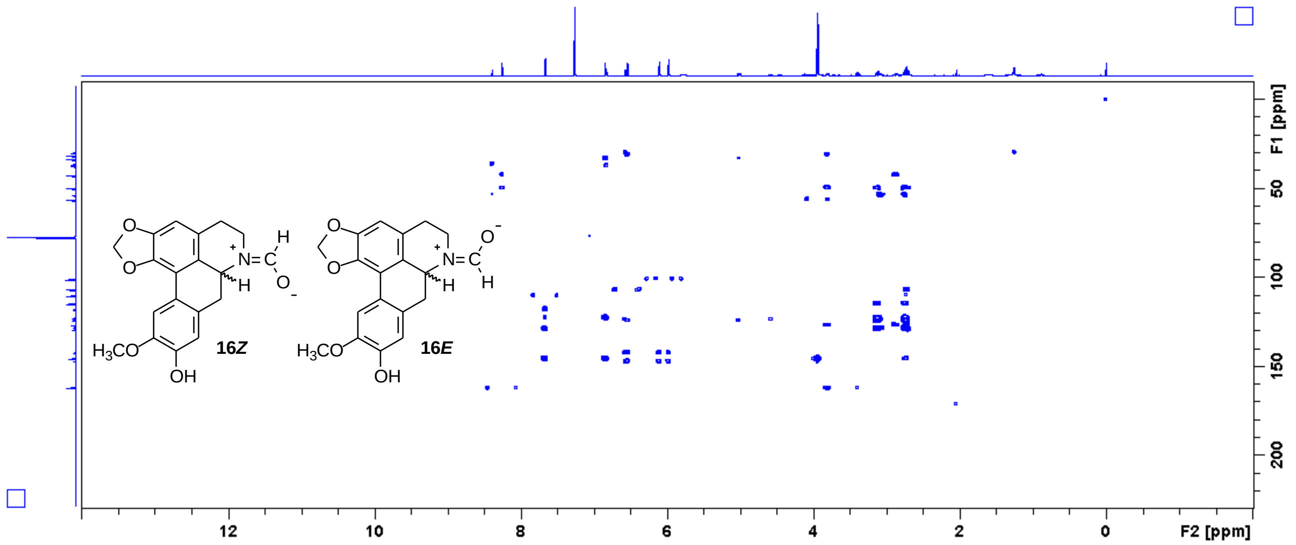

Figure S217. Long-range ^{1}H–^{13}C HMBC correlation map of **(*Z*/*E*)-*N*-formylactinodaphnine** (**16**) recorded in $CDCl_3$ at 500 MHz (^{1}H) and 125 MHz (^{13}C).

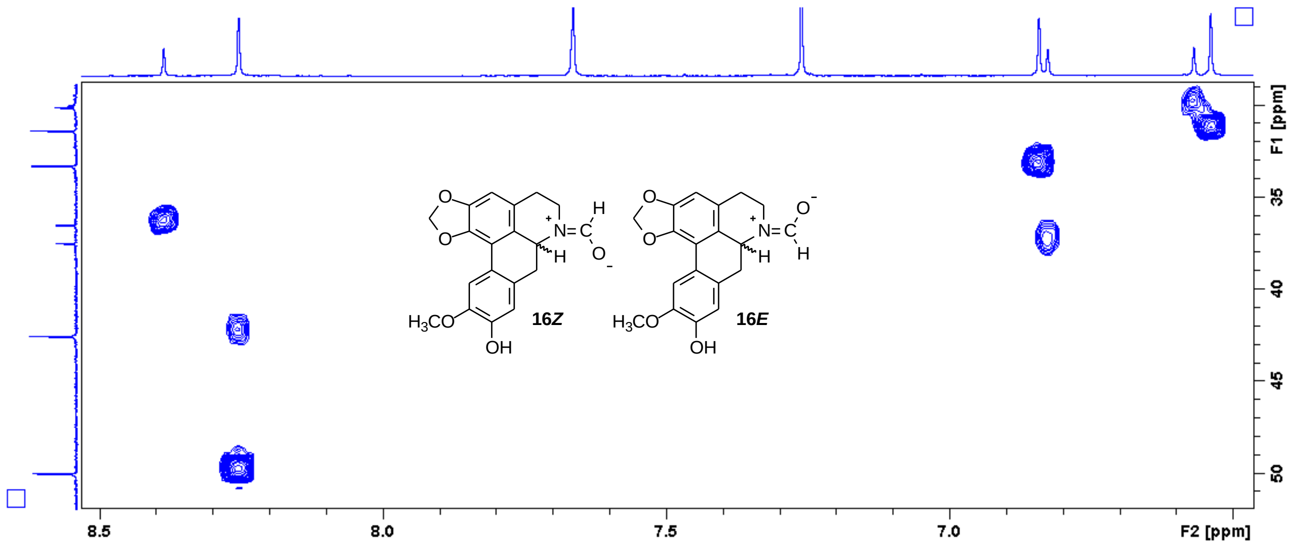

Figure S218. Expanded view 1 of the long-range ^{1}H–^{13}C HMBC correlation map of **(*Z*/*E*)-*N*-formylactinodaphnine** (**16**) recorded in $CDCl_3$ at 500 MHz (^{1}H) and 125 MHz (^{13}C).

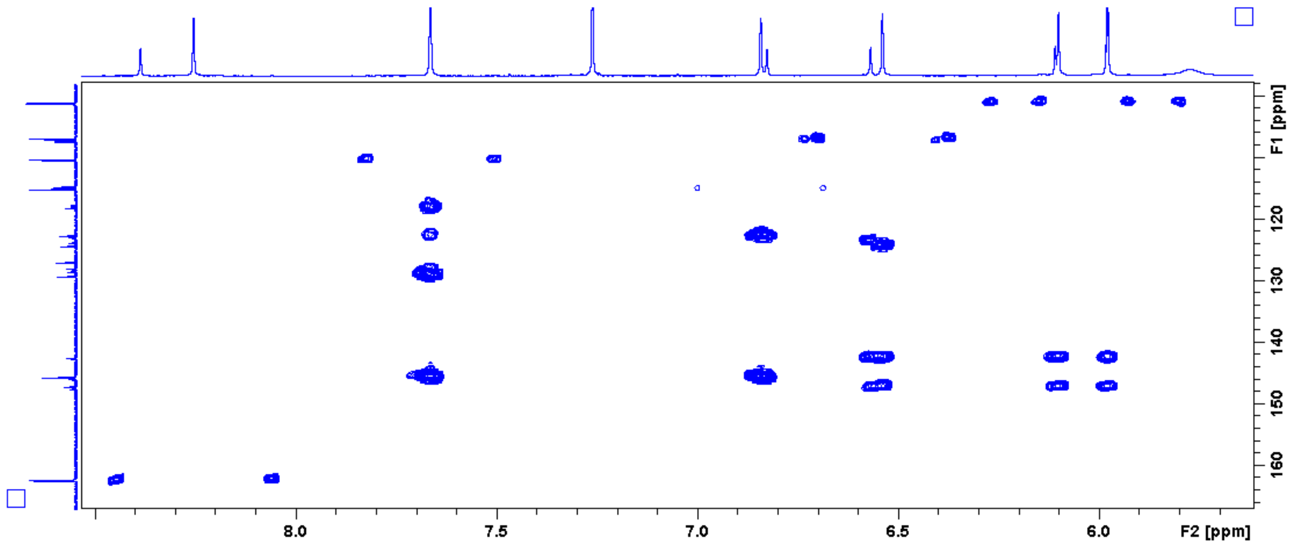

Figure S219. Expanded view 2 of the long-range ^{1}H–^{13}C HMBC correlation map of **(*Z*/*E*)-*N*-formylactinodaphnine** (**16**) recorded in $CDCl_3$ at 500 MHz (^{1}H) and 125 MHz (^{13}C).

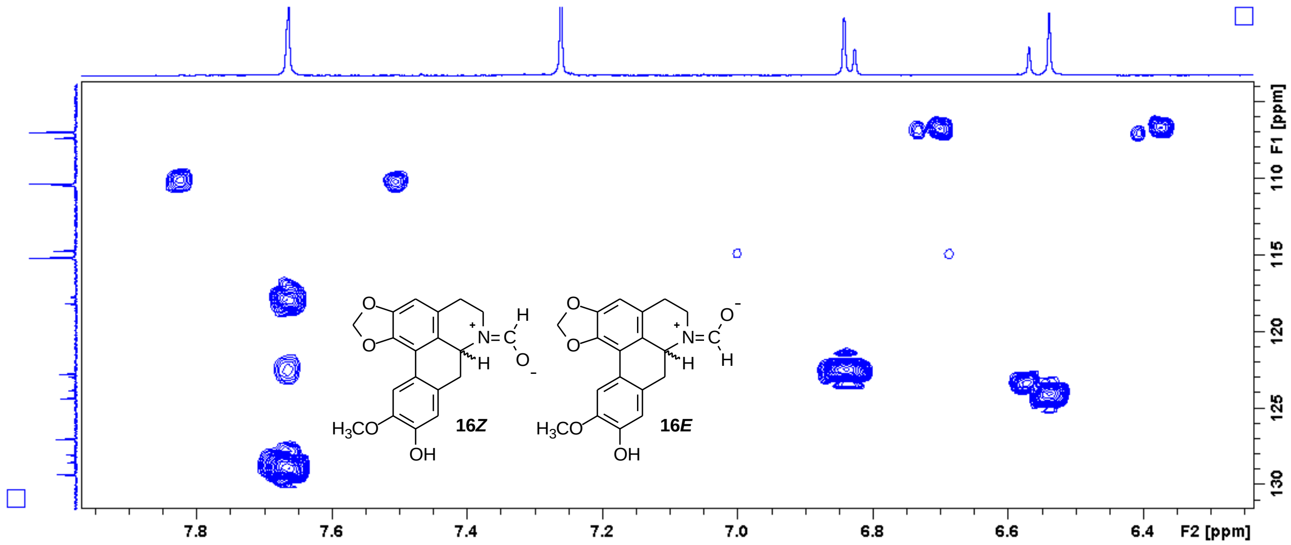

Figure S220. Expanded view 3 of the long-range ^{1}H–^{13}C HMBC correlation map of ***(Z/E)-N*-formylactinodaphnine** (**16**) recorded in $CDCl_3$ at 500 MHz (^{1}H) and 125 MHz (^{13}C).

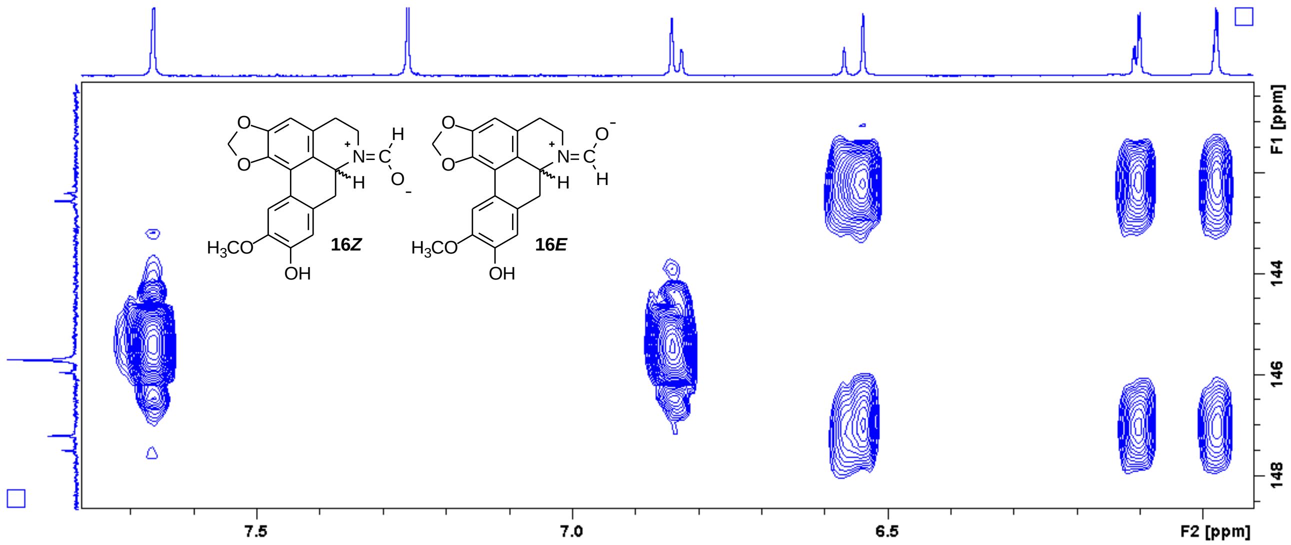

Figure S221. Expanded view 4 of the long-range ^{1}H–^{13}C HMBC correlation map of ***(Z/E)-N*-formylactinodaphnine** (**16**) recorded in $CDCl_3$ at 500 MHz (^{1}H) and 125 MHz (^{13}C).

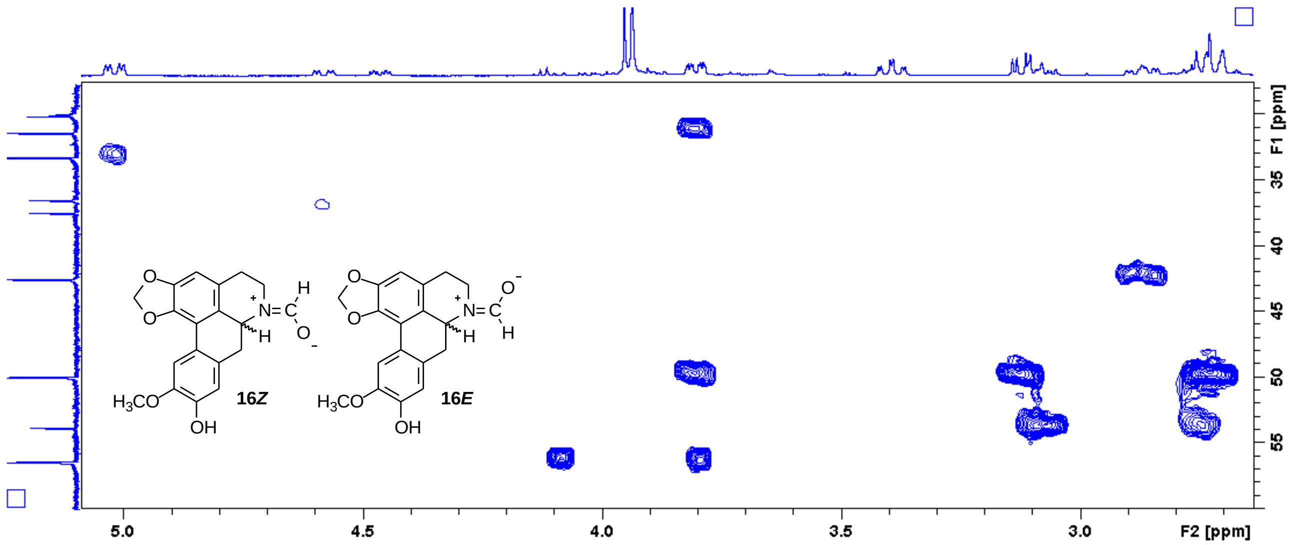

Figure S222. Expanded view 5 of the long-range ^{1}H–^{13}C HMBC correlation map of ***(Z/E)-N*-formylactinodaphnine** (**16**) recorded in $CDCl_3$ at 500 MHz (^{1}H) and 125 MHz (^{13}C).

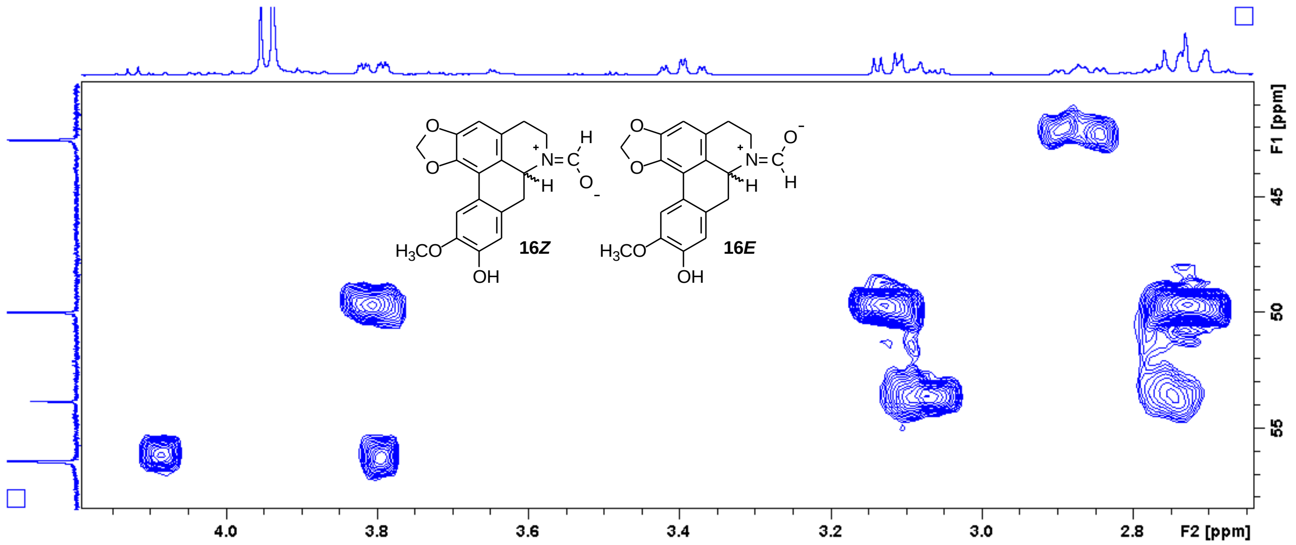

Figure S223. Expanded view 6 of the long-range ^{1}H–^{13}C HMBC correlation map of **(*Z*/*E*)-*N*-formylactinodaphnine** (**16**) recorded in $CDCl_3$ at 500 MHz (^{1}H) and 125 MHz (^{13}C).

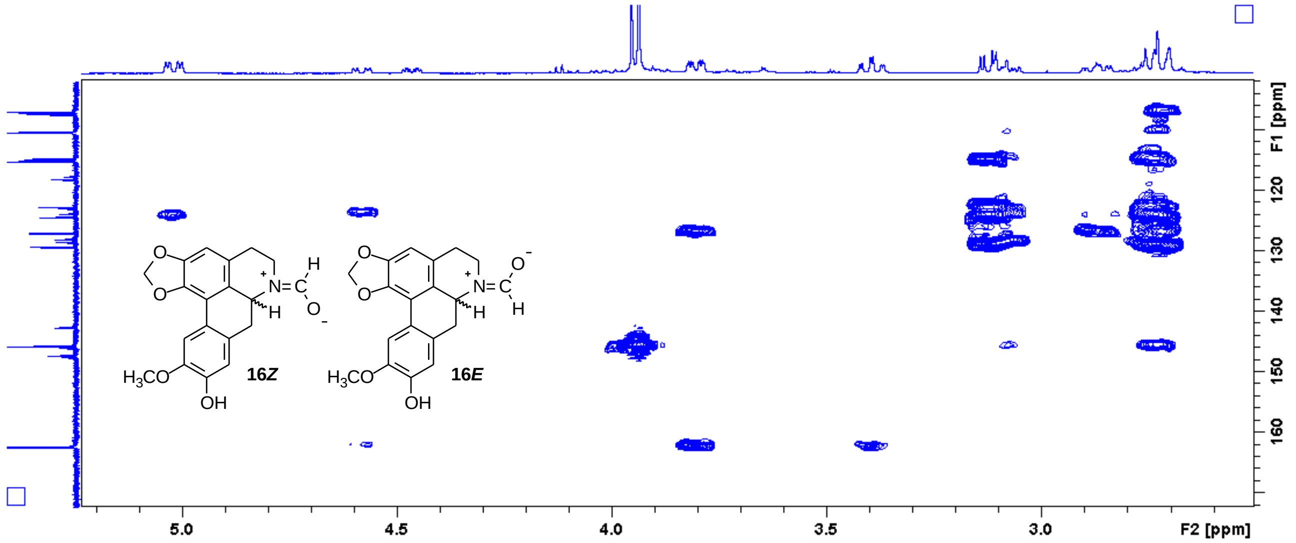

Figure S224. Expanded view 7 of the long-range ^{1}H–^{13}C HMBC correlation map of **(*Z*/*E*)-*N*-formylactinodaphnine** (**16**) recorded in $CDCl_3$ at 500 MHz (^{1}H) and 125 MHz (^{13}C).

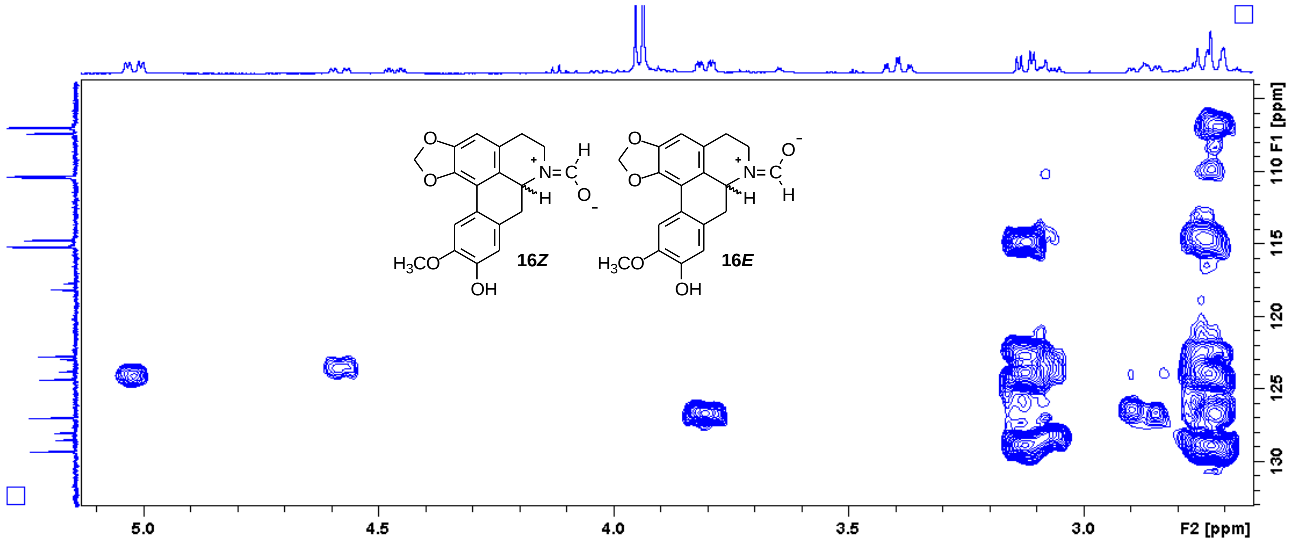

Figure S225. Expanded view 8 of the long-range ^{1}H–^{13}C HMBC correlation map of **(*Z*/*E*)-*N*-formylactinodaphnine** (**16**) recorded in $CDCl_3$ at 500 MHz (^{1}H) and 125 MHz (^{13}C).

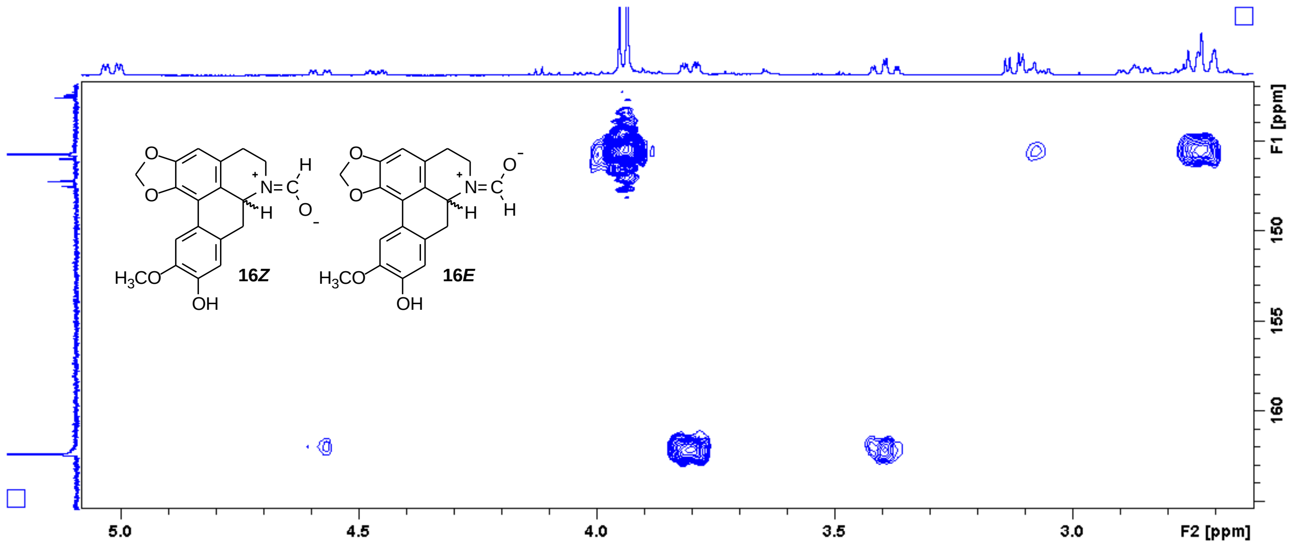

Figure S226. Expanded view 9 of the long-range ^{1}H–^{13}C HMBC correlation map of ***(Z/E)-N*-formylactinodaphnine** (**16**) recorded in $CDCl_3$ at 500 MHz (^{1}H) and 125 MHz (^{13}C).

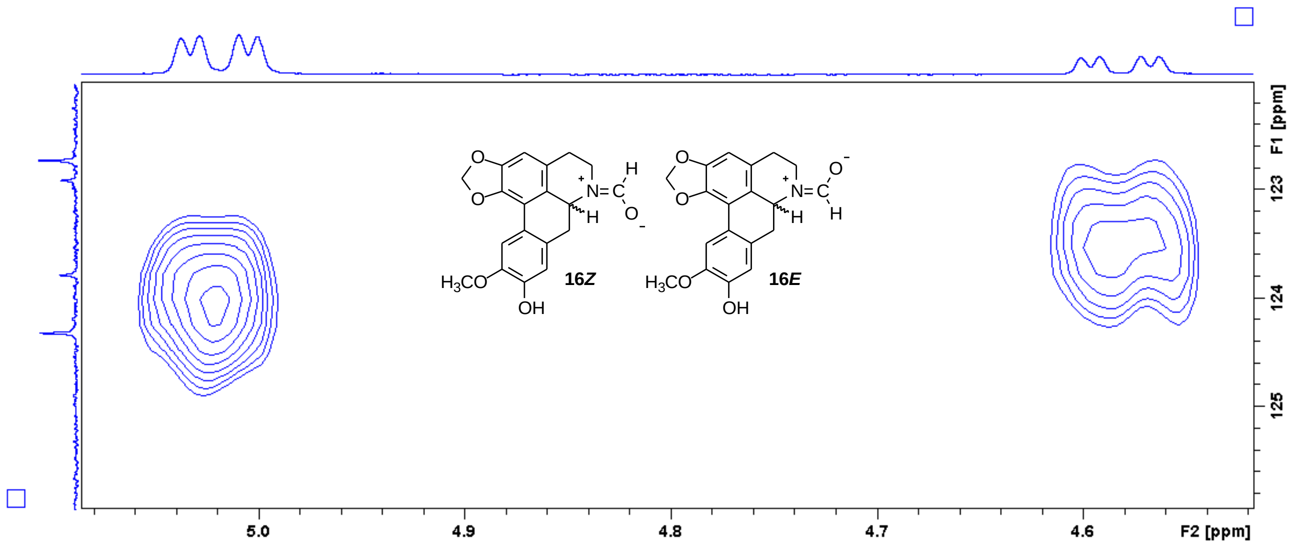

Figure S227. Expanded view 10 of the long-range ^{1}H–^{13}C HMBC correlation map of ***(Z/E)-N*-formylactinodaphnine** (**16**) recorded in $CDCl_3$ at 500 MHz (^{1}H) and 125 MHz (^{13}C).

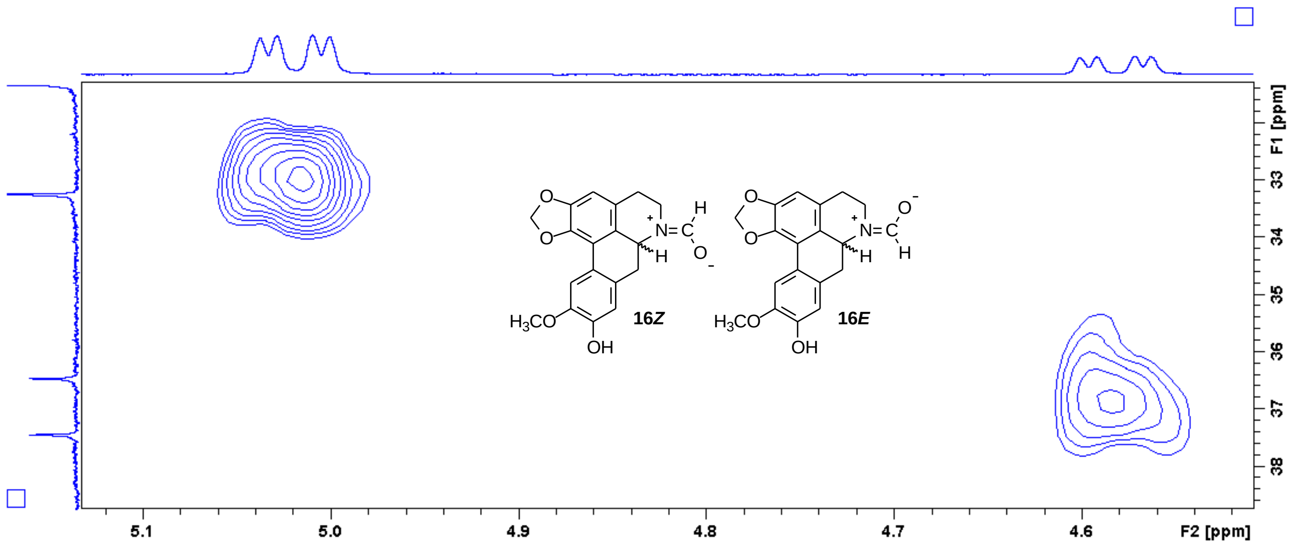

Figure S228. Expanded view 11 of the long-range ^{1}H–^{13}C HMBC correlation map of ***(Z/E)-N*-formylactinodaphnine** (**16**) recorded in $CDCl_3$ at 500 MHz (^{1}H) and 125 MHz (^{13}C).

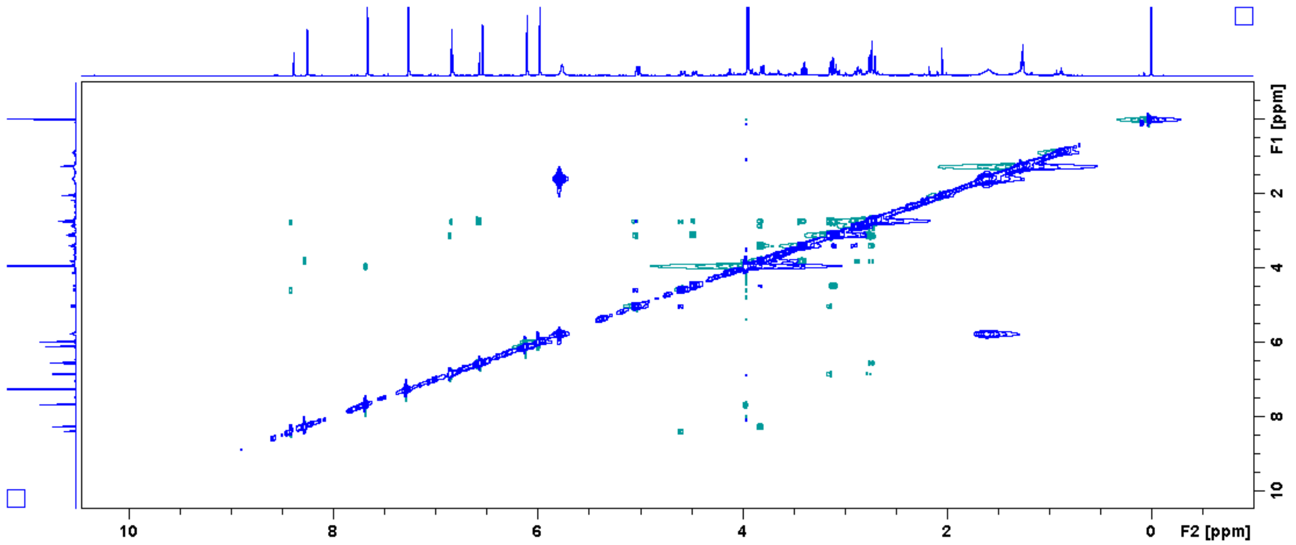

Figure S229. Two-dimensional ^{1}H–^{1}H NOESY spectrum of **(*Z*/*E*)-*N*-formylactinodaphnine** (**16**) recorded in $CDCl_3$ at 500 MHz. Diagnostic regions are expanded and annotated in Figures S230 and S231.

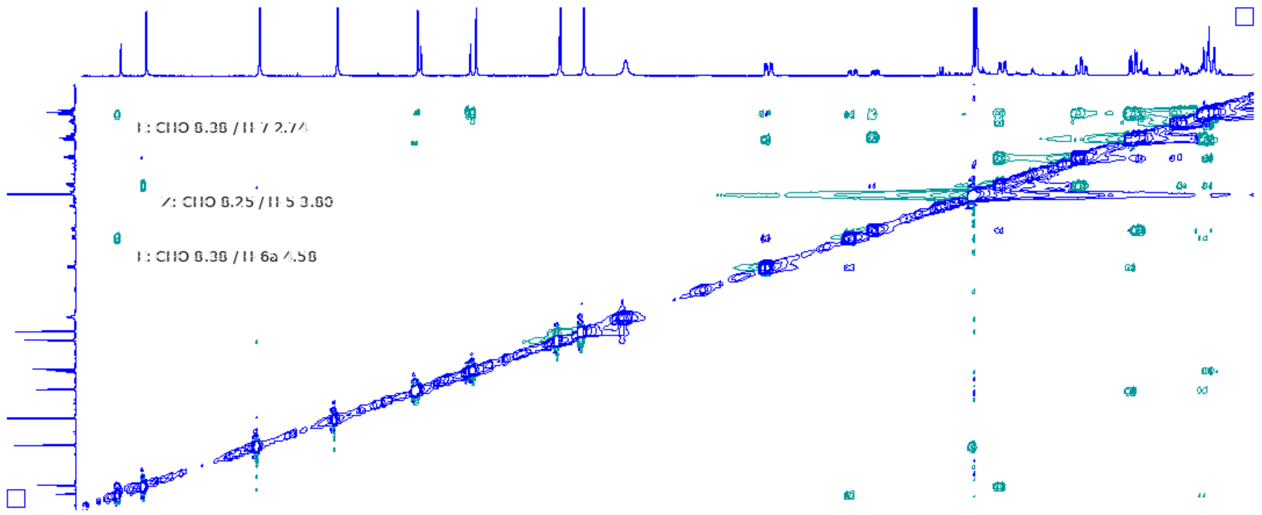

Figure S230. Expanded and annotated region of the two-dimensional ^{1}H–^{1}H NOESY spectrum of **(*Z*/*E*)-*N*-formylactinodaphnine** (**16**) recorded in $CDCl_3$ at 500 MHz. Diagnostic spatial correlations used for the tentative conformational assignments are δ_H 8.25 (CHO)/3.80 (H-5) for the major *Z*-assigned state and δ_H 8.38 (CHO)/4.58 (H-6a) and δH 8.38 (CHO)/2.74 (H-7) for the minor *E*-assigned state.

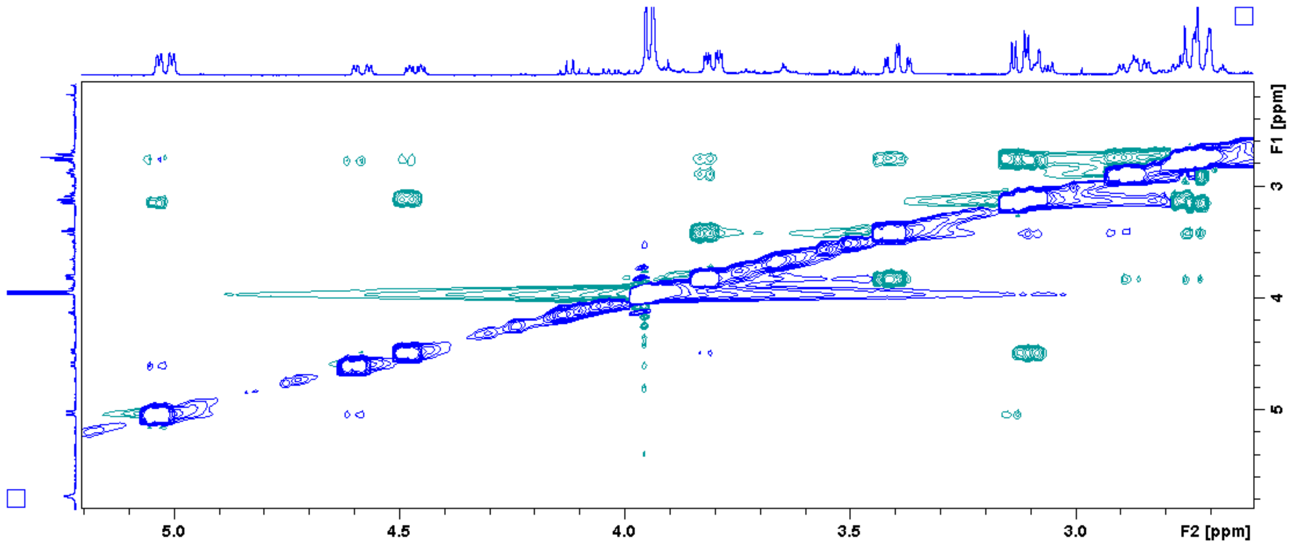

Figure S231. Additional expanded NOESY region of **(*Z*/*E*)-*N*-formylactinodaphnine** (**16**) recorded in $CDCl_3$ at 500 MHz. The NOESY correlations support spatial assignments only and do not constitute direct evidence of chemical exchange between the two NMR states.

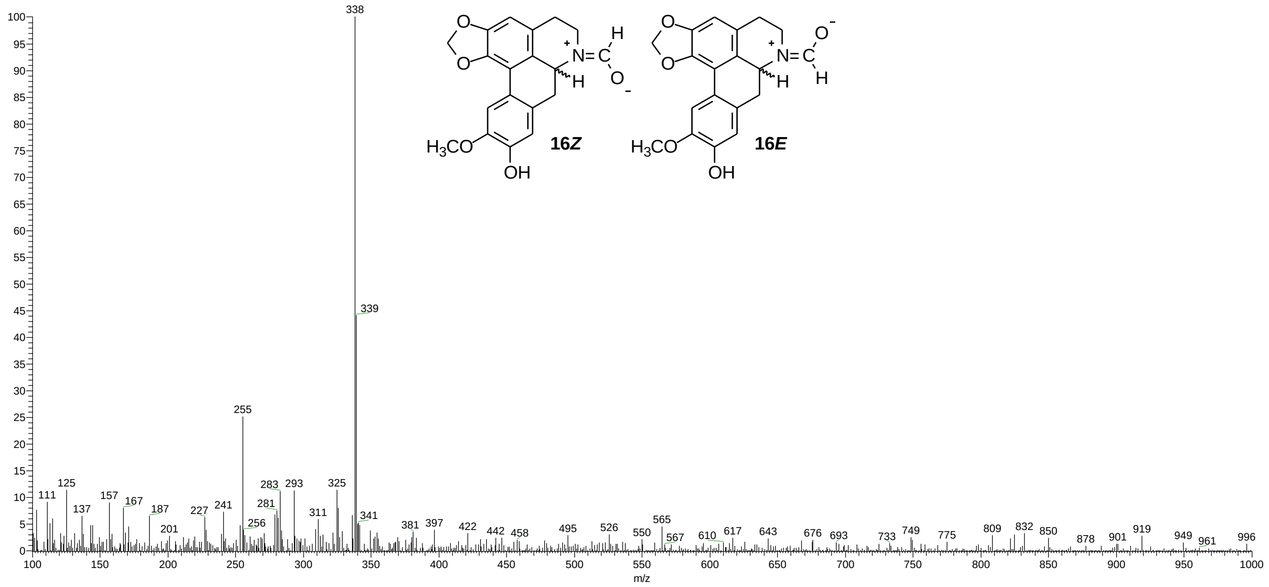

Figure S232. LR-APCI(−)-MS spectrum of **(*Z*/*E*)-*N*-formylactinodaphnine** (**16**), showing the deprotonated molecular ion at *m/z* 338 $[M-H]^-$.

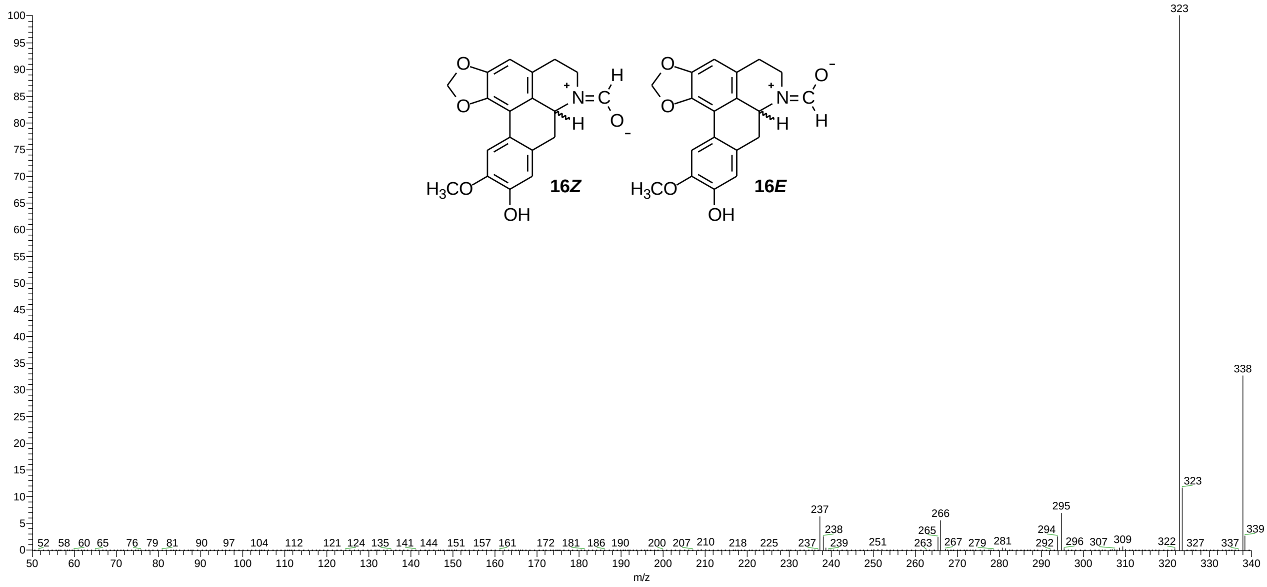

Figure S233. LR-APCI(−)-MS/MS spectrum of the deprotonated molecule of **(*Z*/*E*)-*N*-formylactinodaphnine** (**16**) at *m/z* 338 $[M-H]^-$.

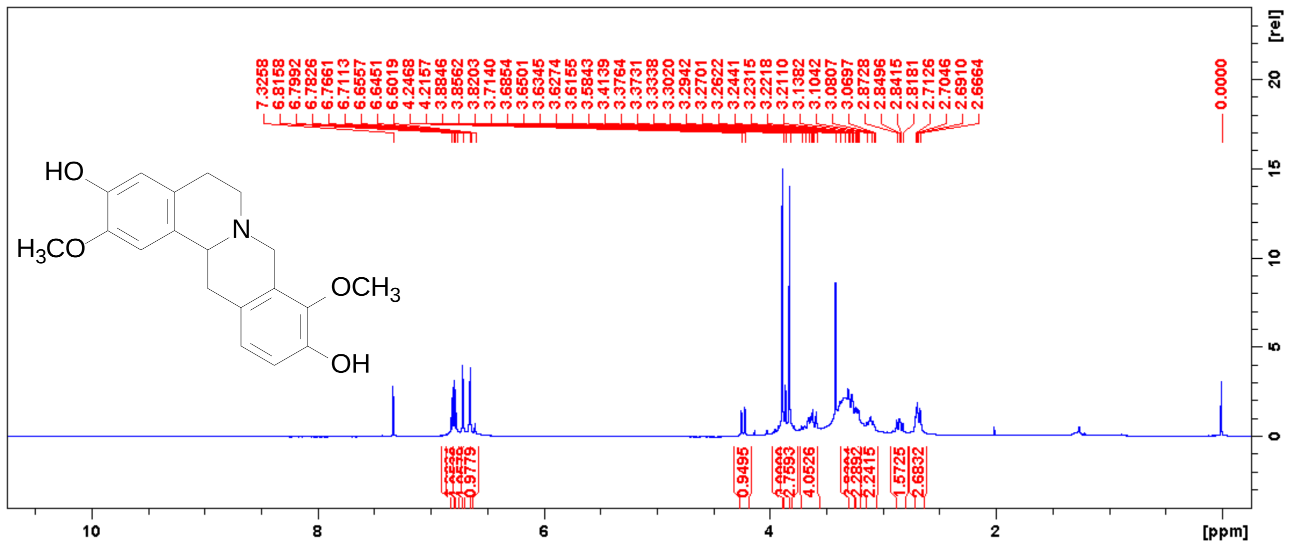

Figure S234. Full ^{1}H NMR spectrum of **discretamine** (**17**) recorded in $CDCl_3$ with a drop of CD_3OD at 500 MHz.

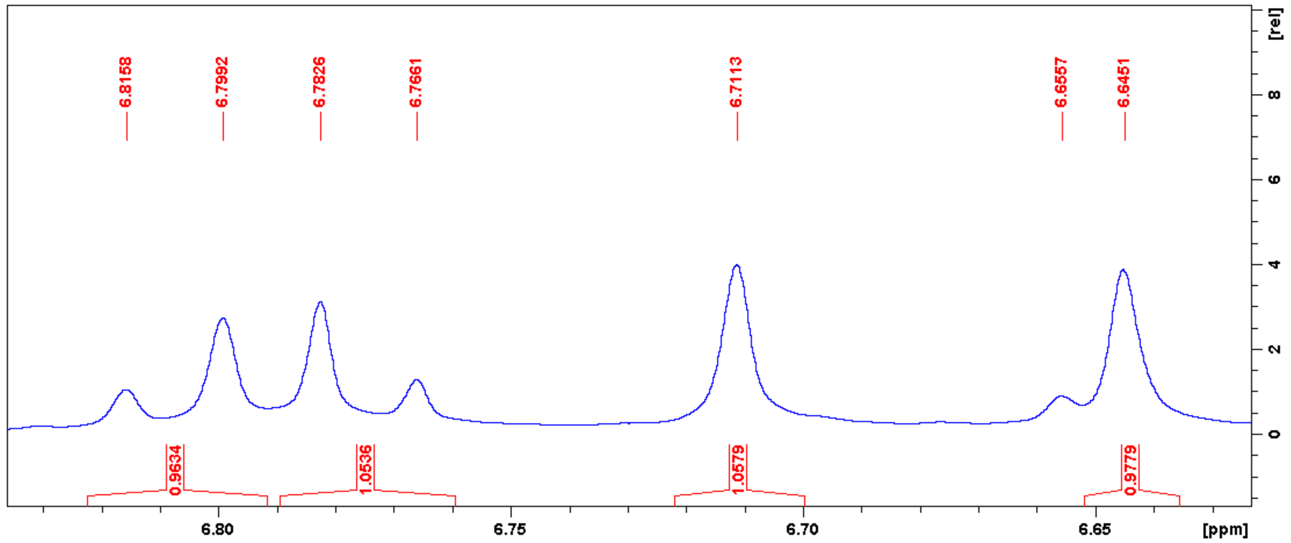

Figure S235. Expanded view 1 of the aromatic proton region in the ^{1}H NMR spectrum of **discretamine** (**17**) recorded in $CDCl_3$ with a drop of CD_3OD at 500 MHz.

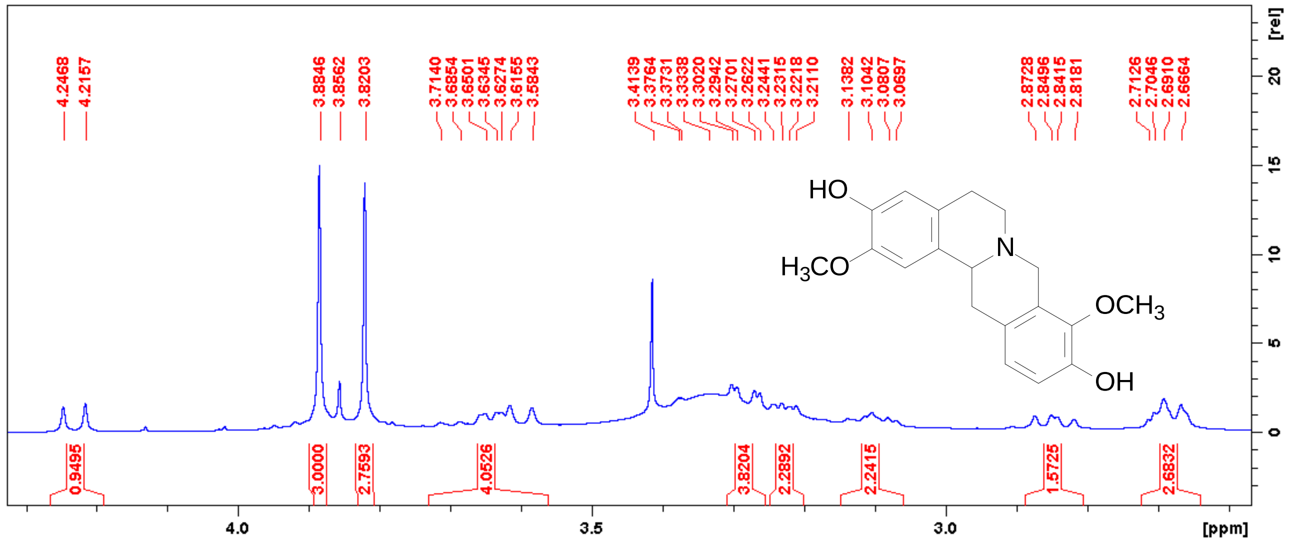

Figure S236. Expanded view 2 of the aliphatic proton region in the ^{1}H NMR spectrum of **discretamine** (**17**) recorded in $CDCl_3$ with a drop of CD_3OD at 500 MHz.

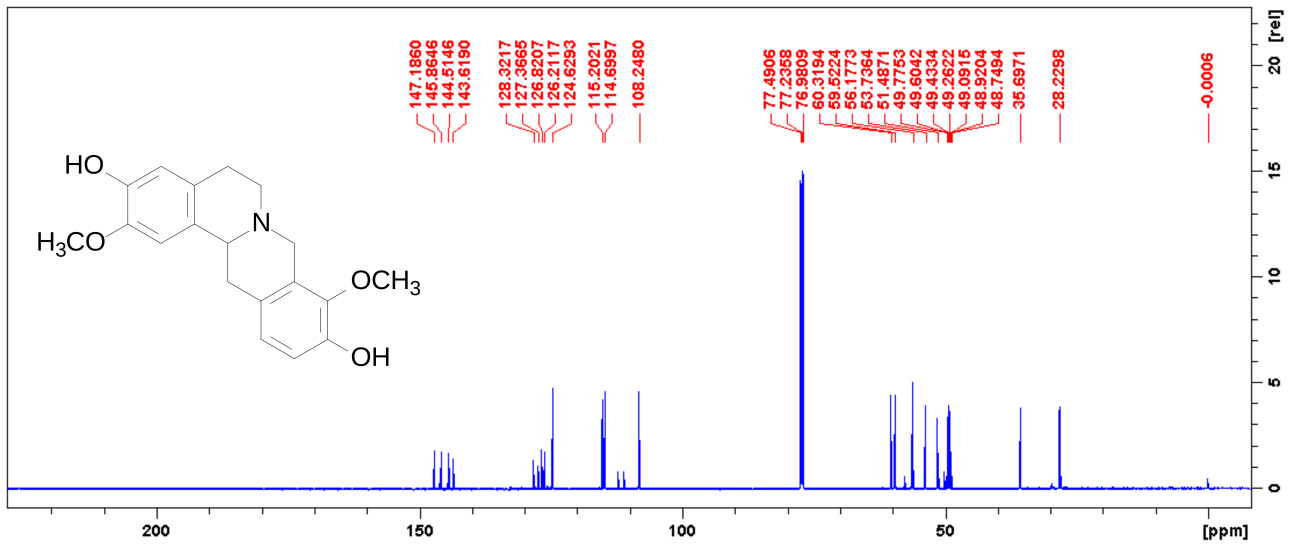

Figure S237. Full ^{13}C NMR spectrum of **discretamine** (**17**) recorded in $CDCl_3$ with a drop of CD_3OD at 125 MHz.

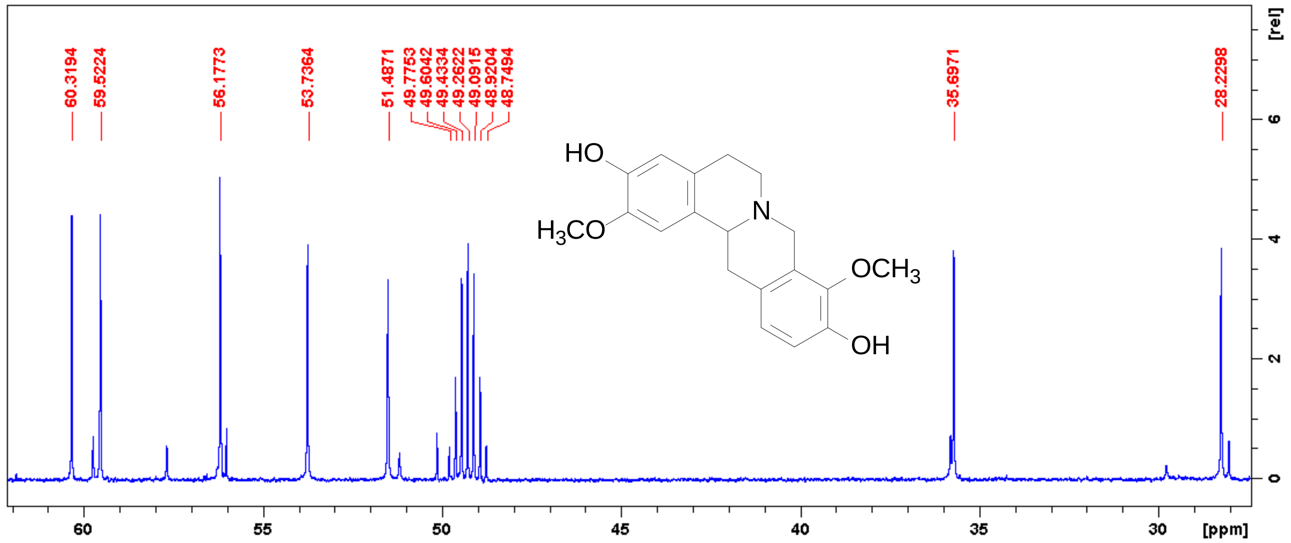

Figure S238. Expanded view of the carbon region (δ_C 148.2–106.7 ppm) in the ^{13}C NMR spectrum of **discretamine** (**17**) recorded in $CDCl_3$ with a drop of CD_3OD at 125 MHz.

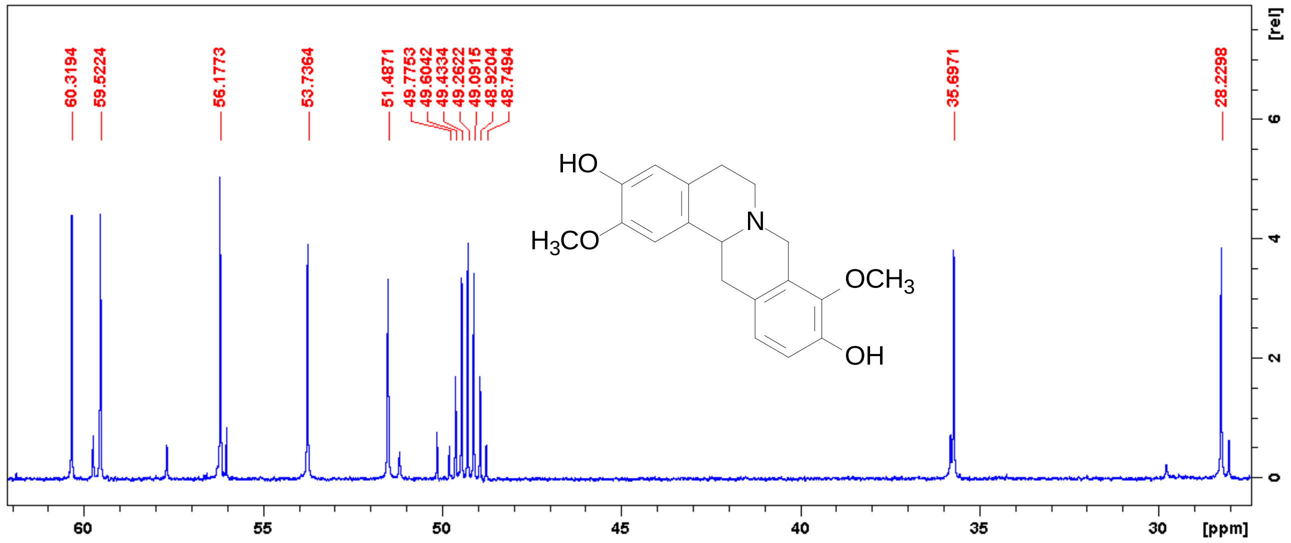

Figure S239. Expanded view of the carbon region (δ_C 62.0–27.5 ppm) in the ^{13}C NMR spectrum of **discretamine** (**17**) recorded in $CDCl_3$ with a drop of CD_3OD at 125 MHz.

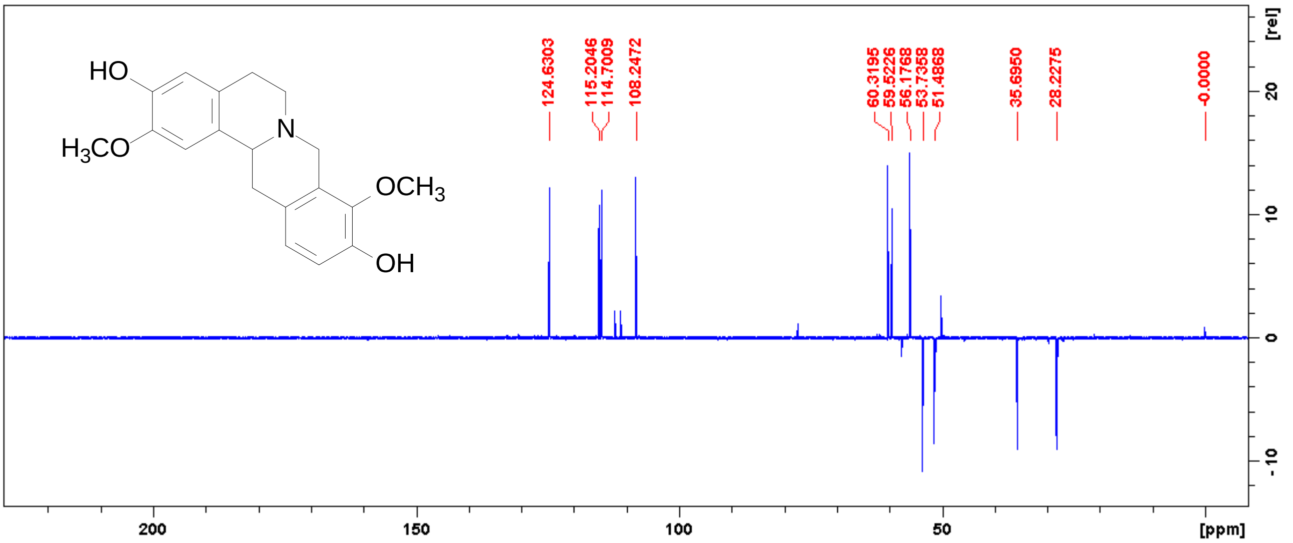

Figure S240. Full DEPT-135 ^{13}C NMR spectrum of **discretamine** (**17**) recorded in $CDCl_3$ with a drop of CD_3OD at 125 MHz.

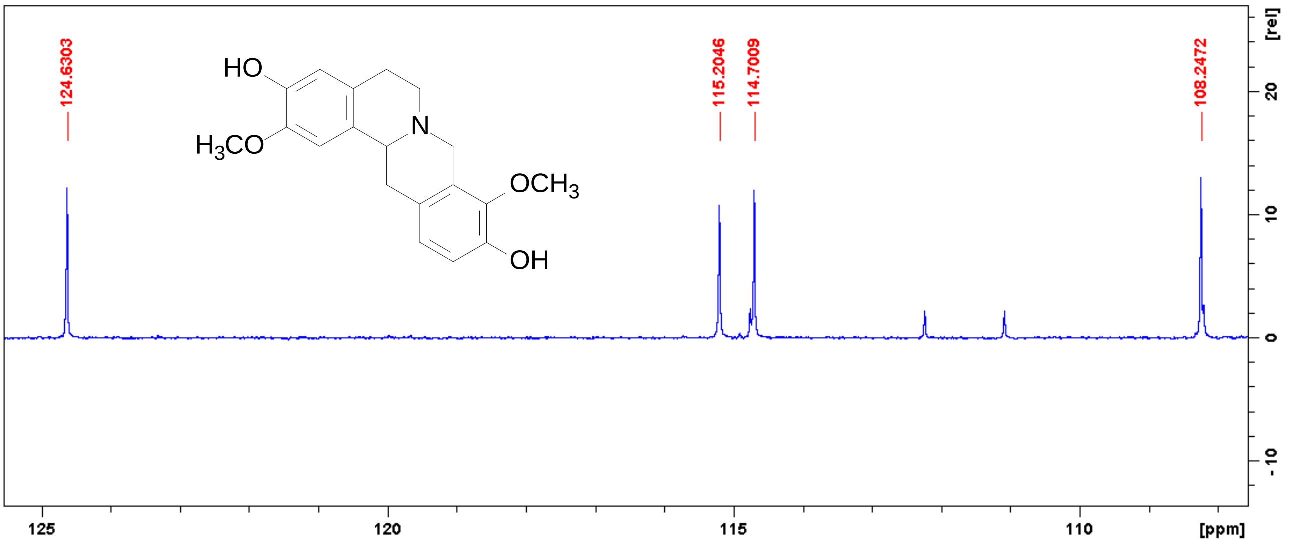

Figure S241. Expanded view of the carbon region (δ_C 125.5–107.6 ppm) in the DEPT-135 ^{13}C NMR spectrum of **discretamine** (**17**) recorded in $CDCl_3$ with a drop of CD_3OD at 125 MHz.

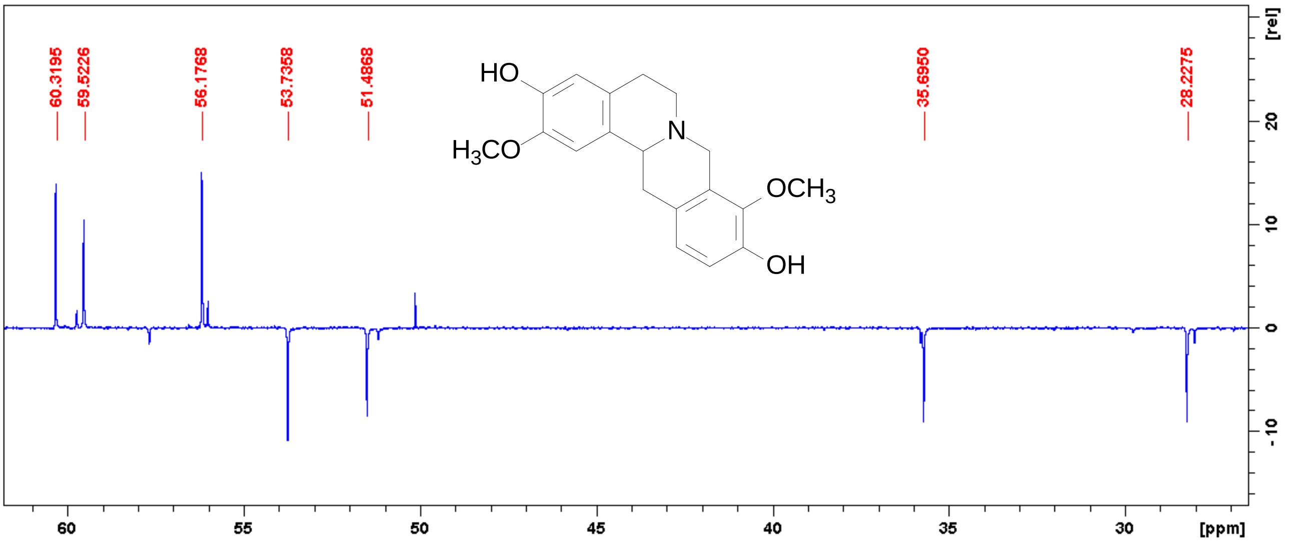

Figure S242. Expanded view of the carbon region (δ_C 61.7–26.7 ppm) in the DEPT-135 ^{13}C NMR spectrum of **discretamine** (**17**) recorded in $CDCl_3$ with a drop of CD_3OD at 125 MHz.

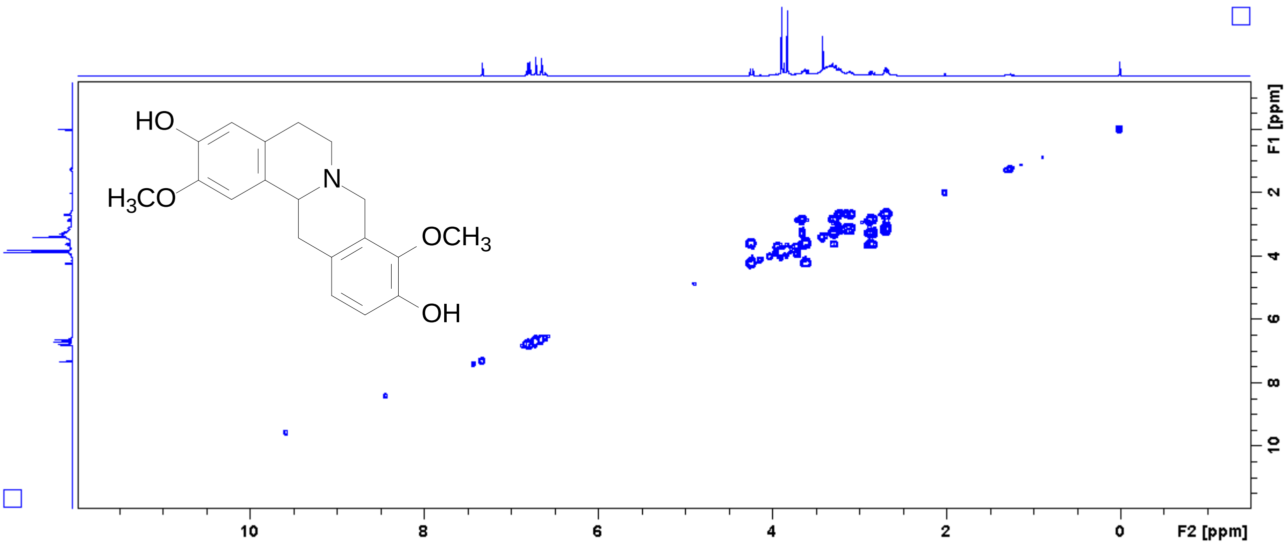

Figure S243. ^1H–^1H COSY correlation map of **discretamine** (**17**) recorded in $CDCl_3$ with a drop of CD_3OD at 500 MHz.

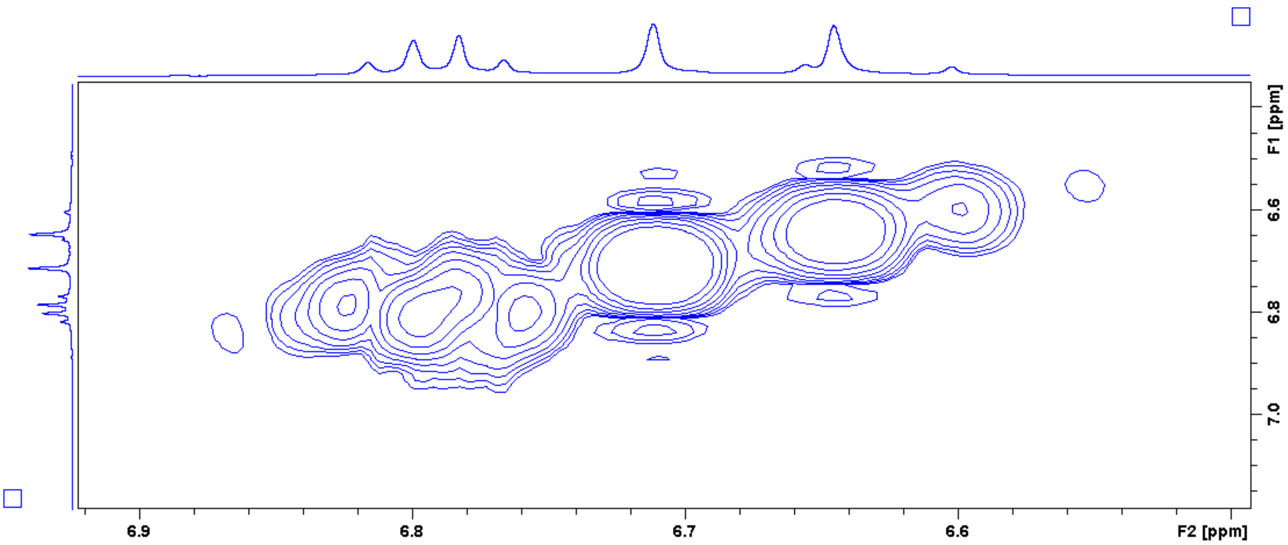

Figure S244. Expanded view 1 of the ^1H–^1H COSY correlation map of **discretamine** (**17**) recorded in $CDCl_3$ with a drop of CD_3OD at 500 MHz.

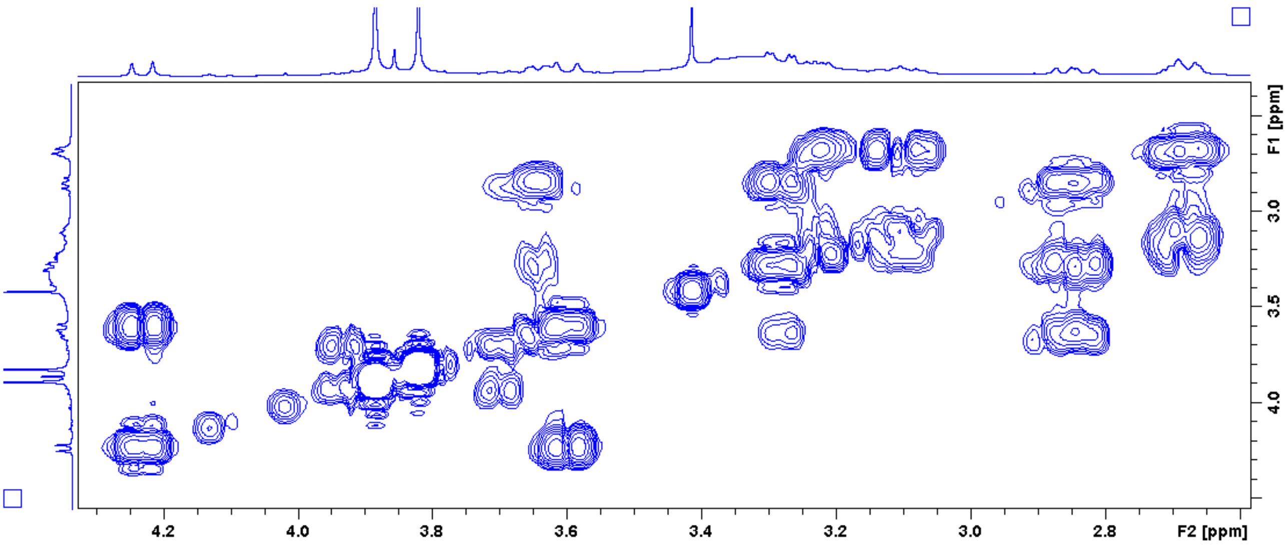

Figure S245. Expanded view 2 of the ^1H–^1H COSY correlation map of **discretamine** (**17**) recorded in $CDCl_3$ with a drop of CD_3OD at 500 MHz.

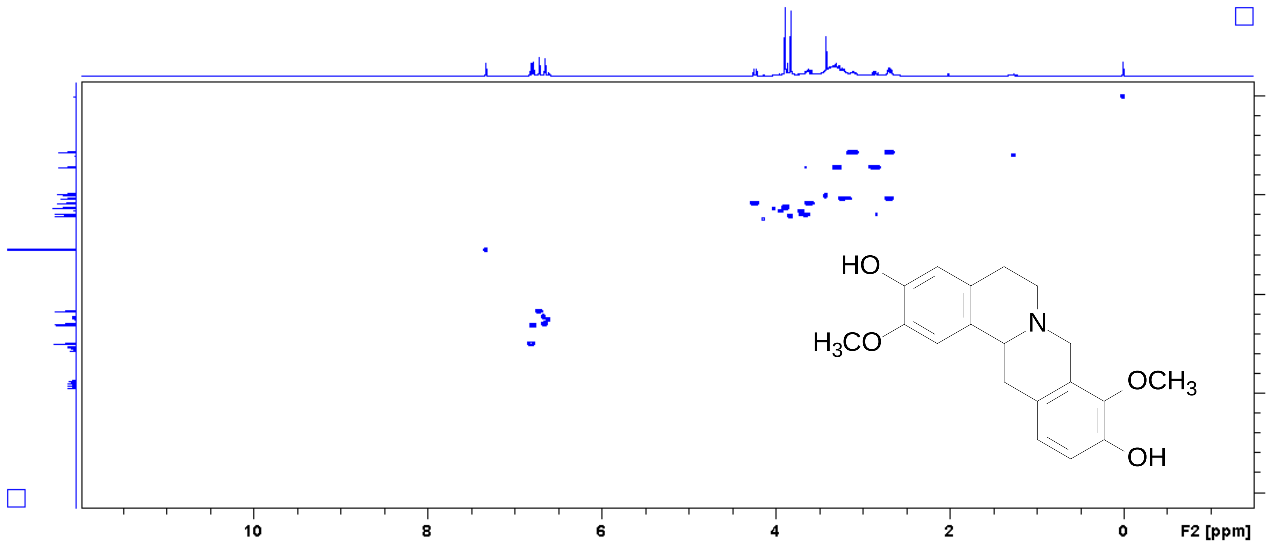

Figure S246. One-bond ^{1}H–^{13}C HSQC correlation map of **discretamine** (**17**) recorded in $CDCl_3$ with a drop of CD_3OD at 500 MHz (^{1}H) and 125 MHz (^{13}C).

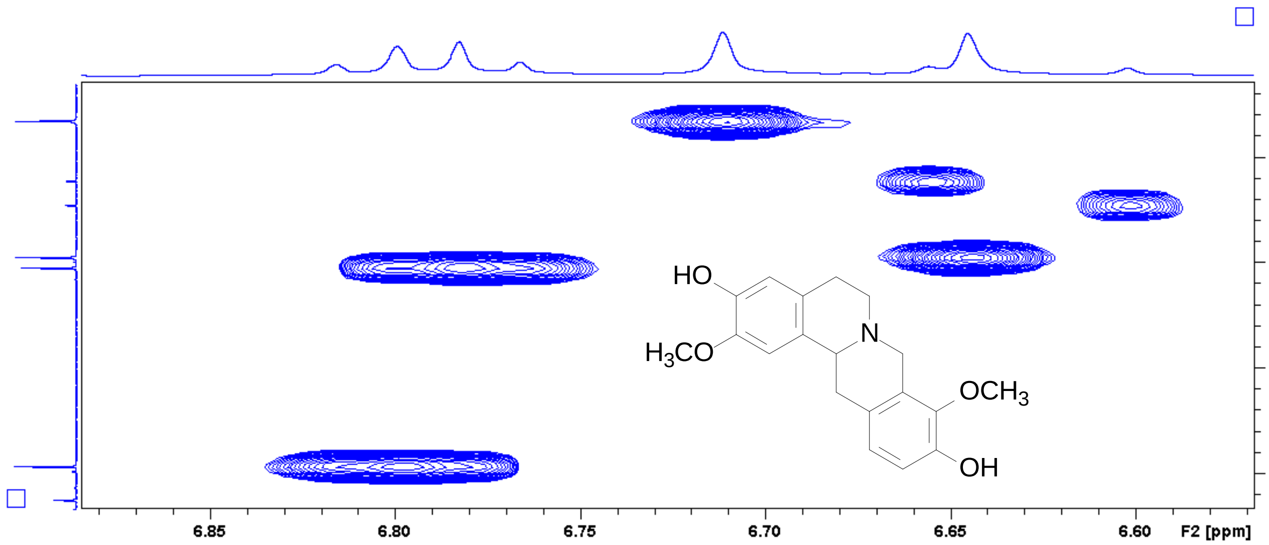

Figure S247. Expanded view 1 of the one-bond ^{1}H–^{13}C HSQC correlation map of **discretamine** (**17**) recorded in $CDCl_3$ with a drop of CD_3OD at 500 MHz (^{1}H) and 125 MHz (^{13}C).

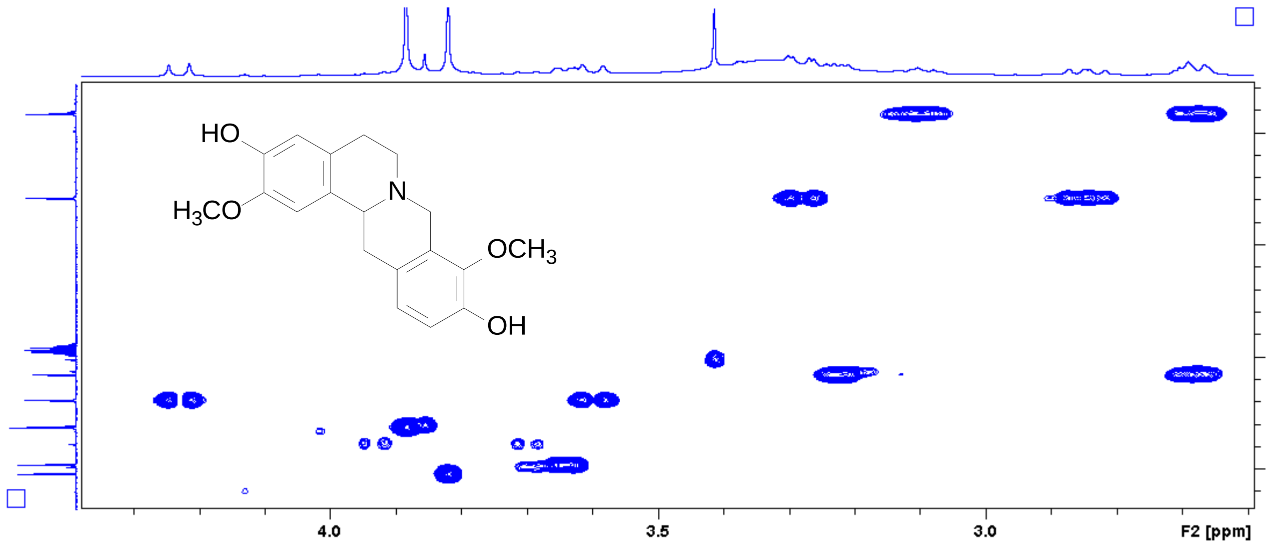

Figure S248. Expanded view 2 of the one-bond ^{1}H–^{13}C HSQC correlation of **discretamine** (**17**) recorded in $CDCl_3$ with a drop of CD_3OD at 500 MHz (^{1}H) and 125 MHz (^{13}C).

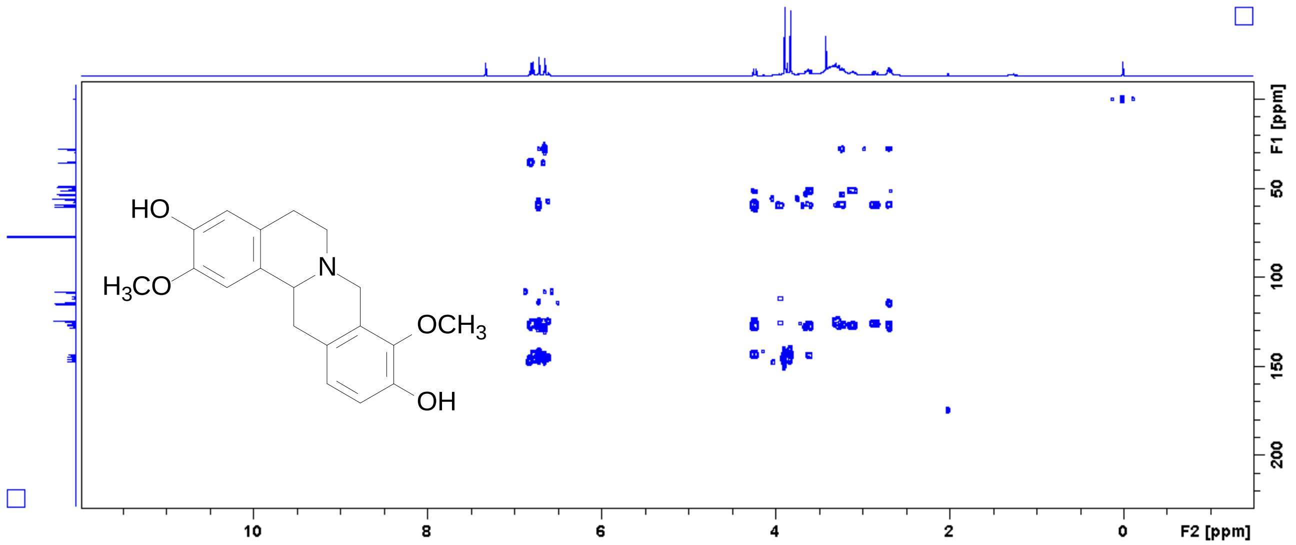

Figure S249. Long-range ^{1}H–^{13}C HMBC correlation map of **discretamine** (**17**) recorded in $CDCl_3$ with a drop of CD_3OD at 500 MHz (^{1}H) and 125 MHz (^{13}C).

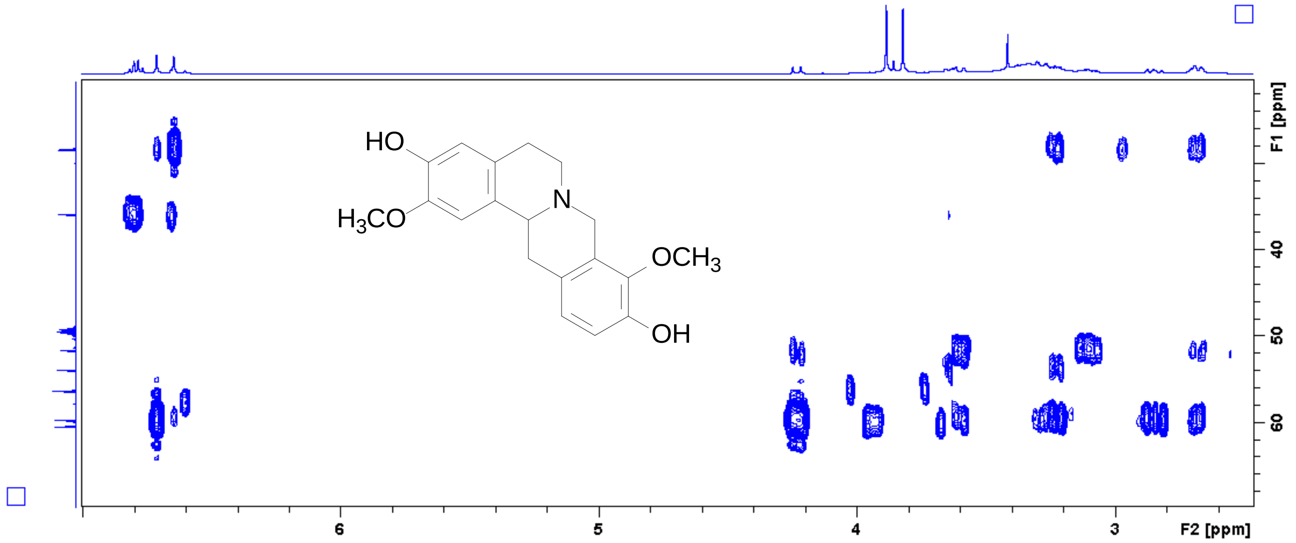

Figure S250. Expanded view 1 of the long-range ^{1}H–^{13}C HMBC correlation map of **discretamine** (**17**) recorded in $CDCl_3$ with a drop of CD_3OD at 500 MHz (^{1}H) and 125 MHz (^{13}C).

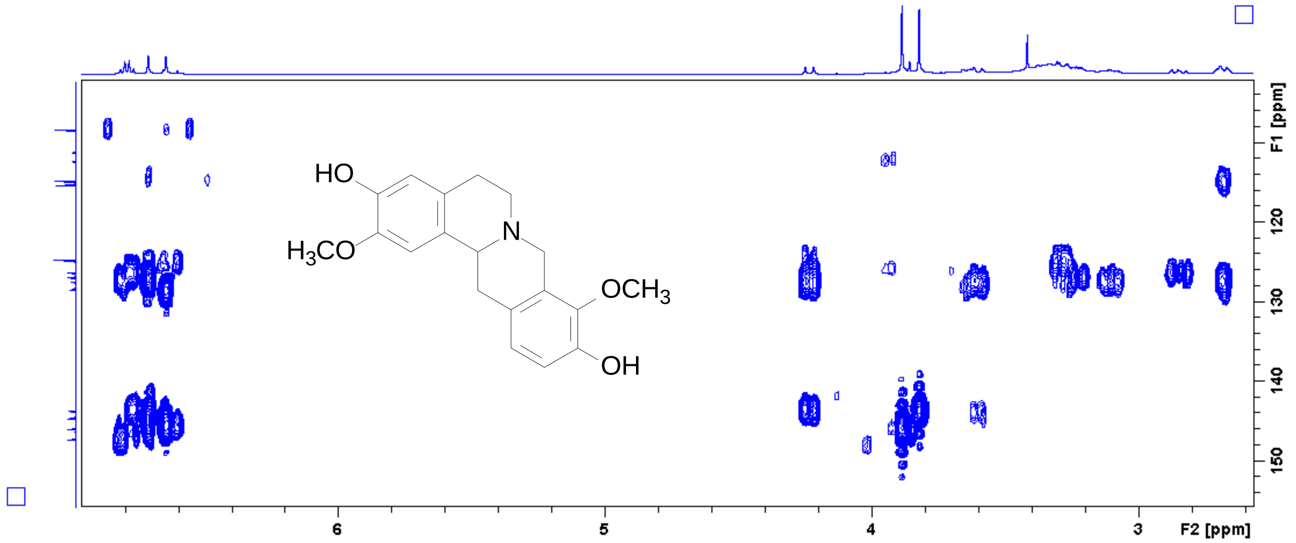

Figure S251. Expanded view 2 of the long-range ^{1}H–^{13}C HMBC correlation map of **discretamine** (**17**) recorded in $CDCl_3$ with a drop of CD_3OD at 500 MHz (^{1}H) and 125 MHz (^{13}C).

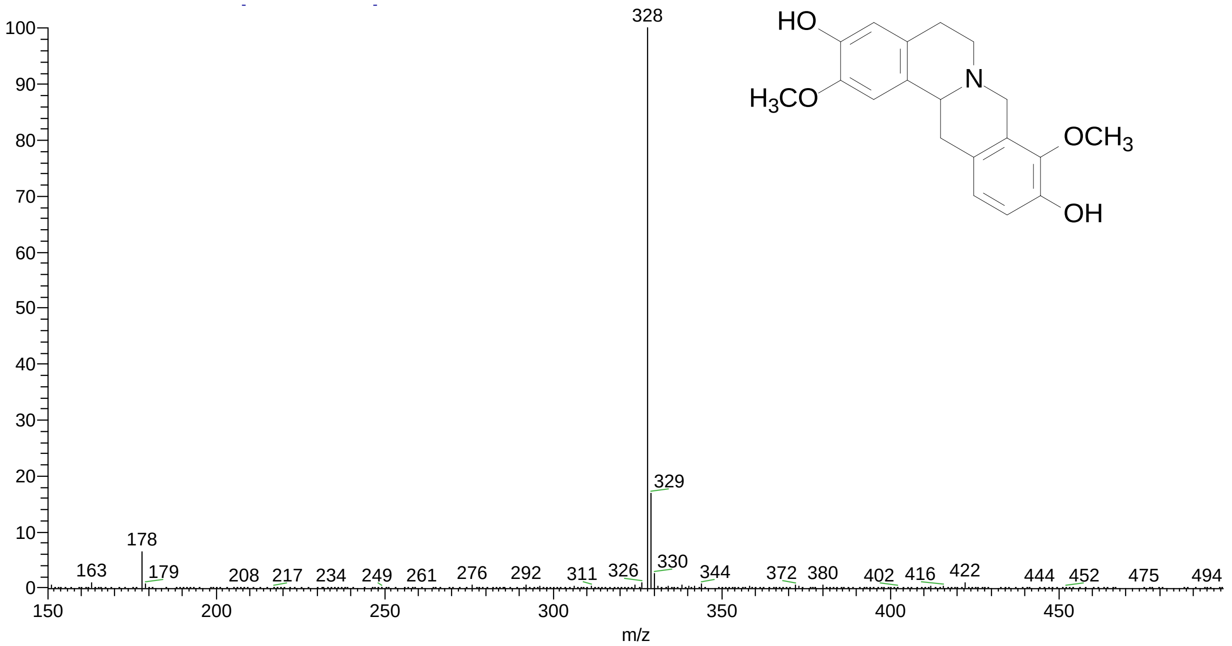

Figure S252. LR-APCI(+)-MS spectrum of **discretamine** (**17**), showing the protonated molecular ion at *m/z* 328 $[M + H]^+$.

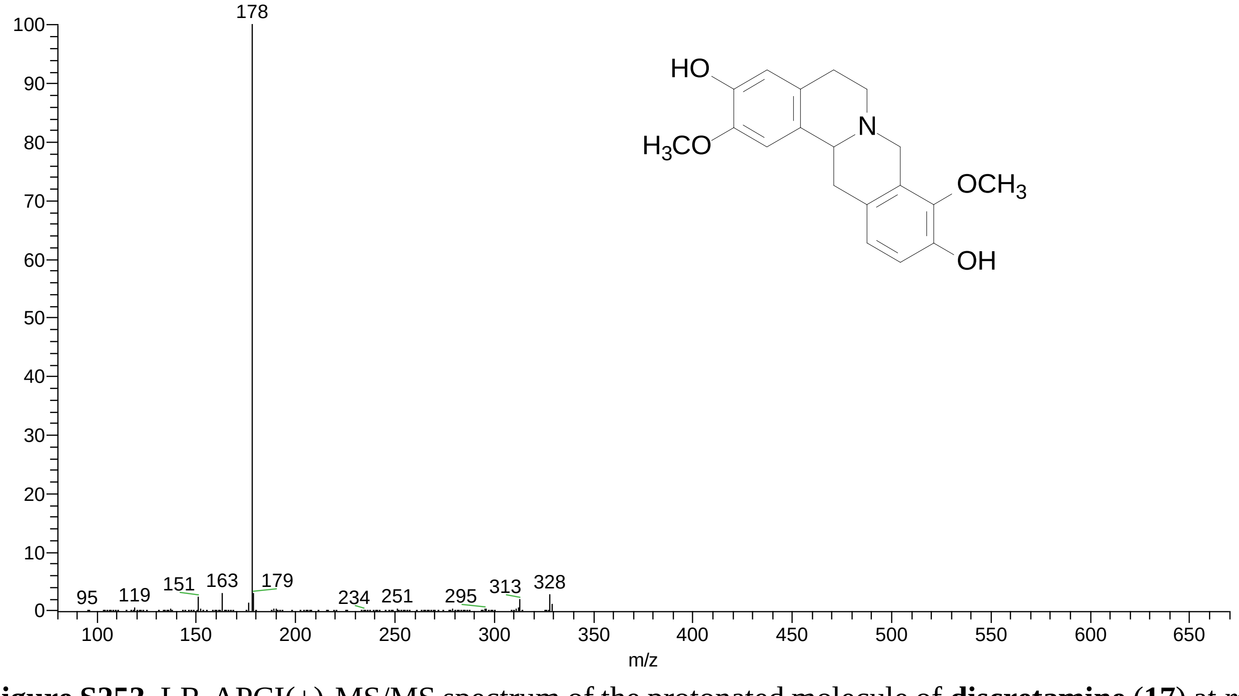

Figure S253. LR-APCI(+)-MS/MS spectrum of the protonated molecule of **discretamine** (**17**) at *m/z* 328 $[M + H]^+$.

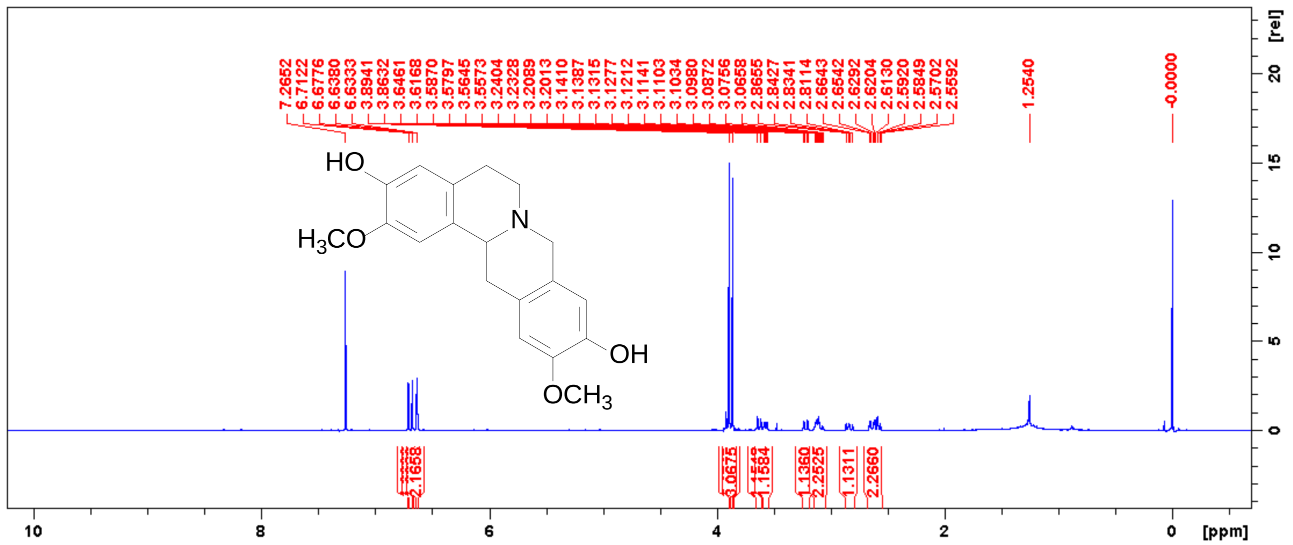

Figure S254. Full ^{1}H NMR spectrum of **10-demethyldiscretine** (**18**) recorded in $CDCl_3$ with a drop of CD_3OD at 500 MHz.

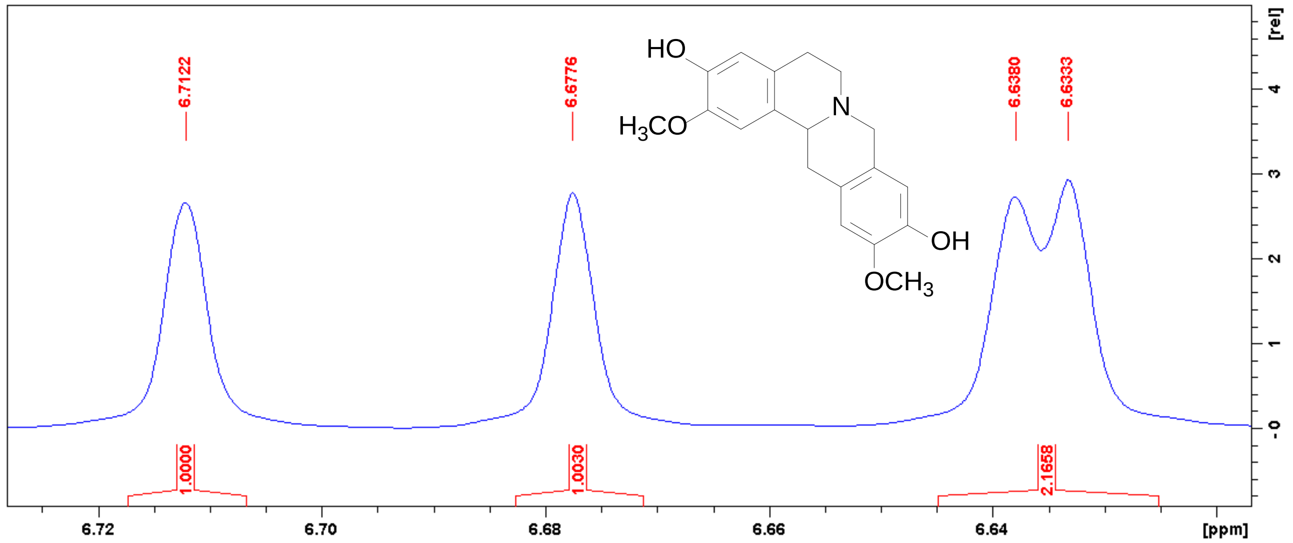

Figure S255. Expanded view 1 of the aromatic proton region in the ^{1}H NMR spectrum of **10-demethyldiscretine** (**18**) recorded in $CDCl_3$ with a drop of CD_3OD at 500 MHz.

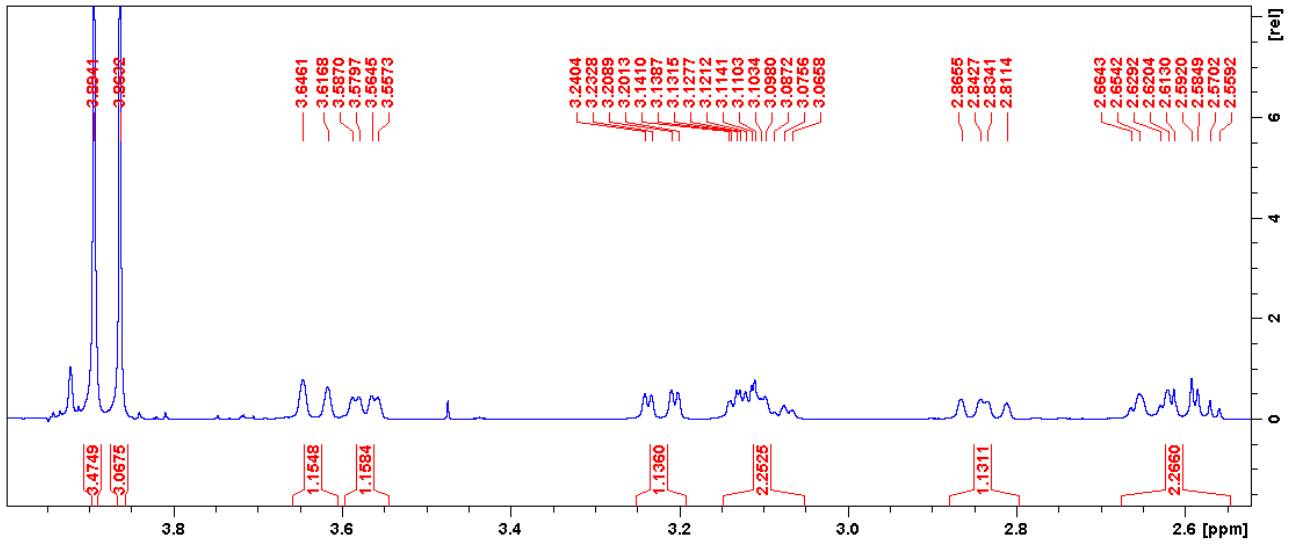

Figure S256. Expanded view 2 of the aliphatic proton region in the ^{1}H NMR spectrum of **10-demethyldiscretine** (**18**) recorded in $CDCl_3$ with a drop of CD_3OD at 500 MHz.

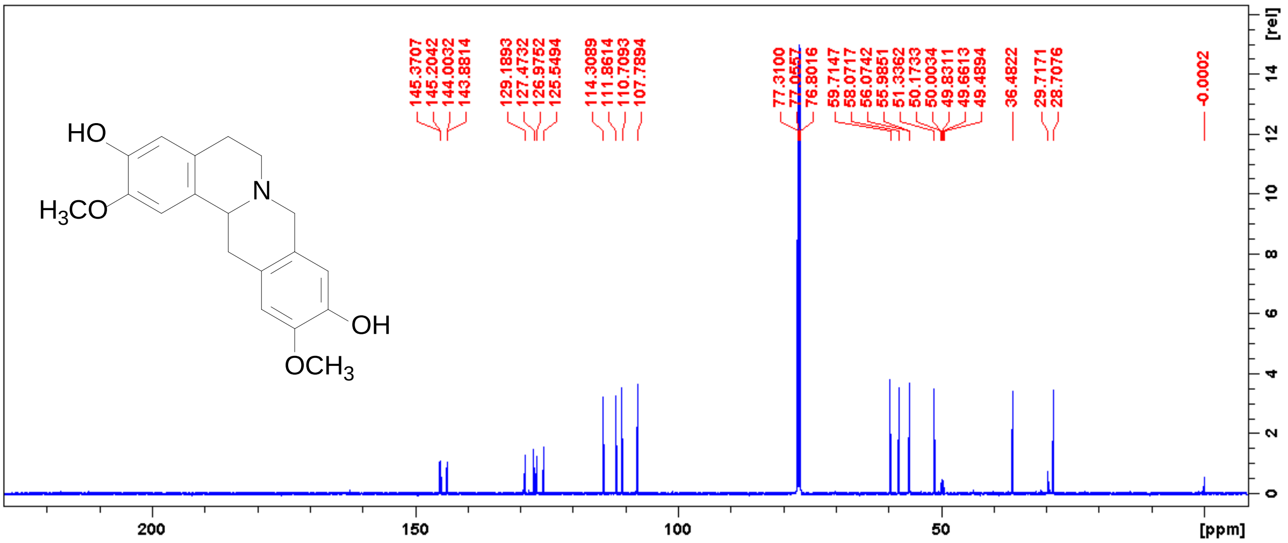

Figure S257. Full ^{13}C NMR spectrum of **10-demethyldiscretine** (**18**) recorded in $CDCl_3$ with a drop of CD_3OD at 125 MHz.

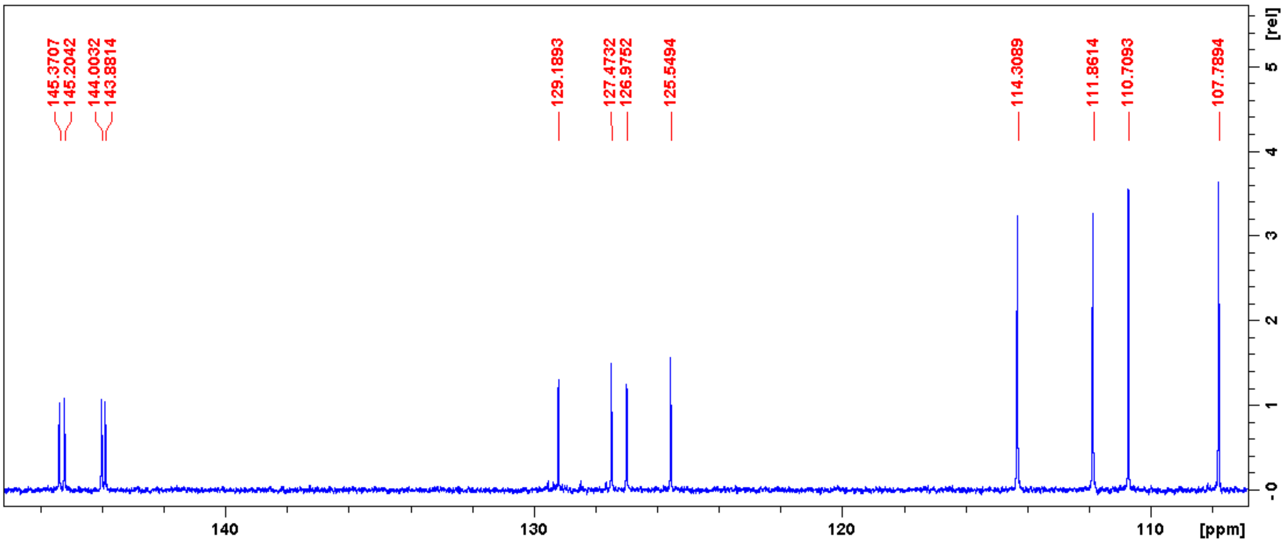

Figure S258. Expanded view of the carbon region (δ_C 147.1–106.9 ppm) in the ^{13}C NMR spectrum of **10-demethyldiscretine** (**18**) recorded in $CDCl_3$ with a drop of CD_3OD at 125 MHz.

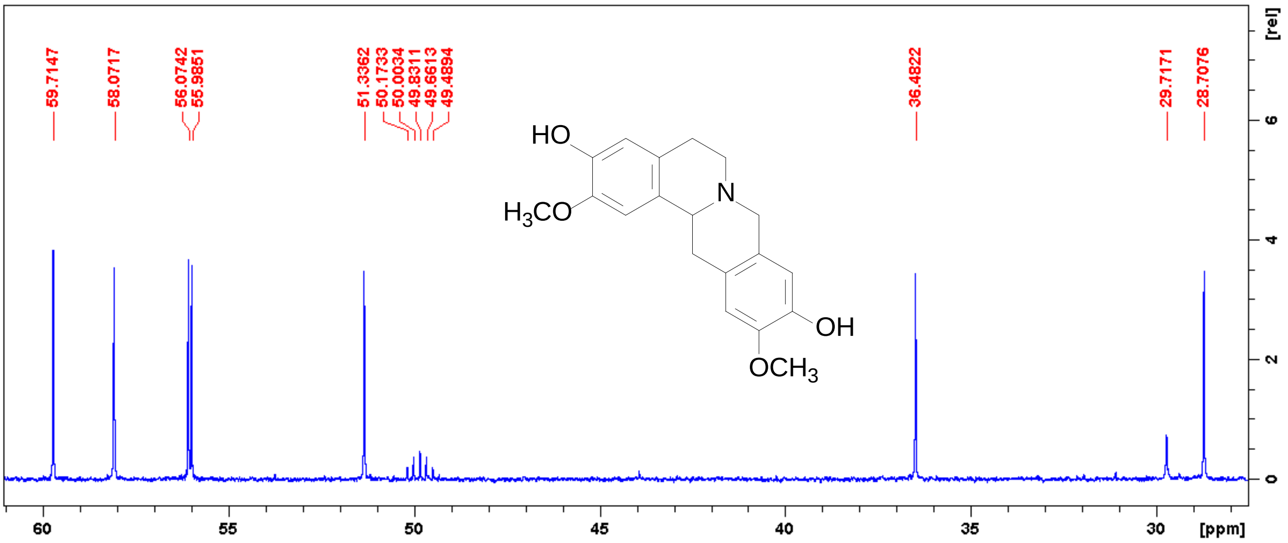

Figure S259. Expanded view of the carbon region (δ_C 61.0–27.5 ppm) in the ^{13}C NMR spectrum of **10-demethyldiscretine** (**18**) recorded in $CDCl_3$ with a drop of CD_3OD at 125 MHz.

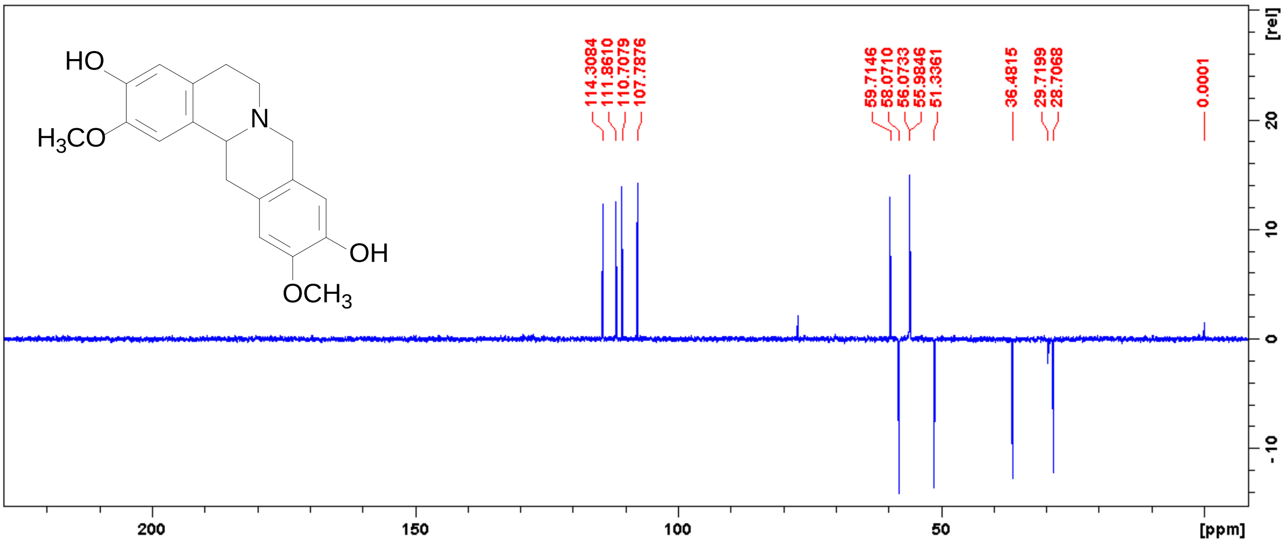

Figure S260. Full DEPT-135 ^{13}C NMR spectrum of **10-demethyldiscretine** (**18**) recorded in $CDCl_3$ with a drop of CD_3OD at 125 MHz.

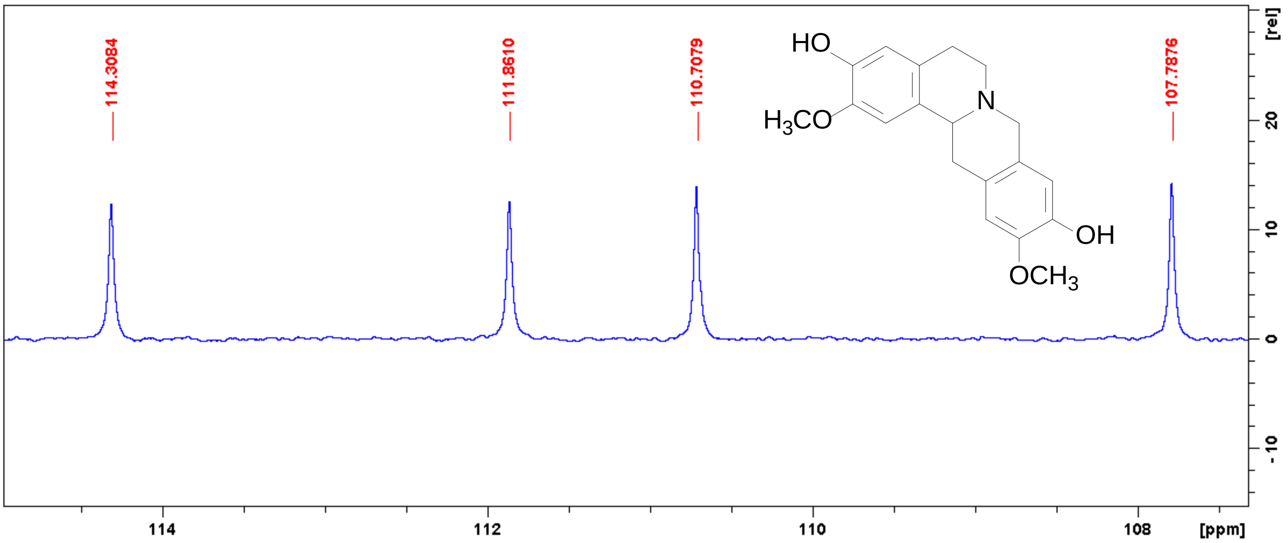

Figure S261. Expanded view of the carbon region (δ_C 114.9–107.3 ppm) in the DEPT-135 ^{13}C NMR spectrum of **10-demethyldiscretine** (**18**) recorded in $CDCl_3$ with a drop of CD_3OD at 125 MHz.

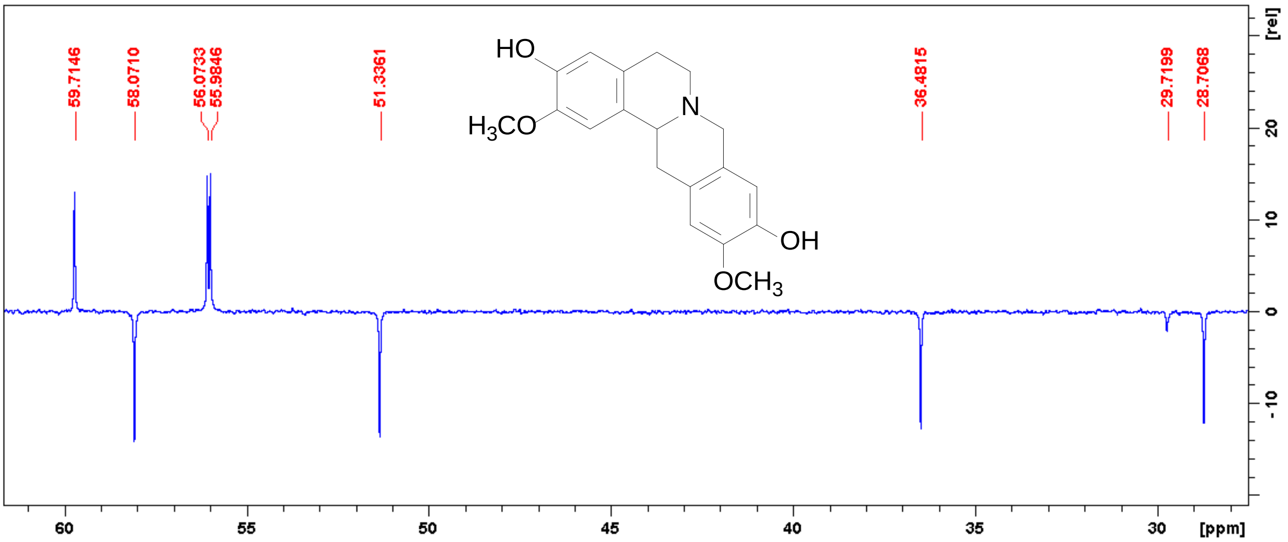

Figure S262. Expanded view of the carbon region (δ_C 61.4–27.5 ppm) in the DEPT-135 ^{13}C NMR spectrum of **10-demethyldiscretine** (**18**) recorded in $CDCl_3$ with a drop of CD_3OD at 125 MHz.

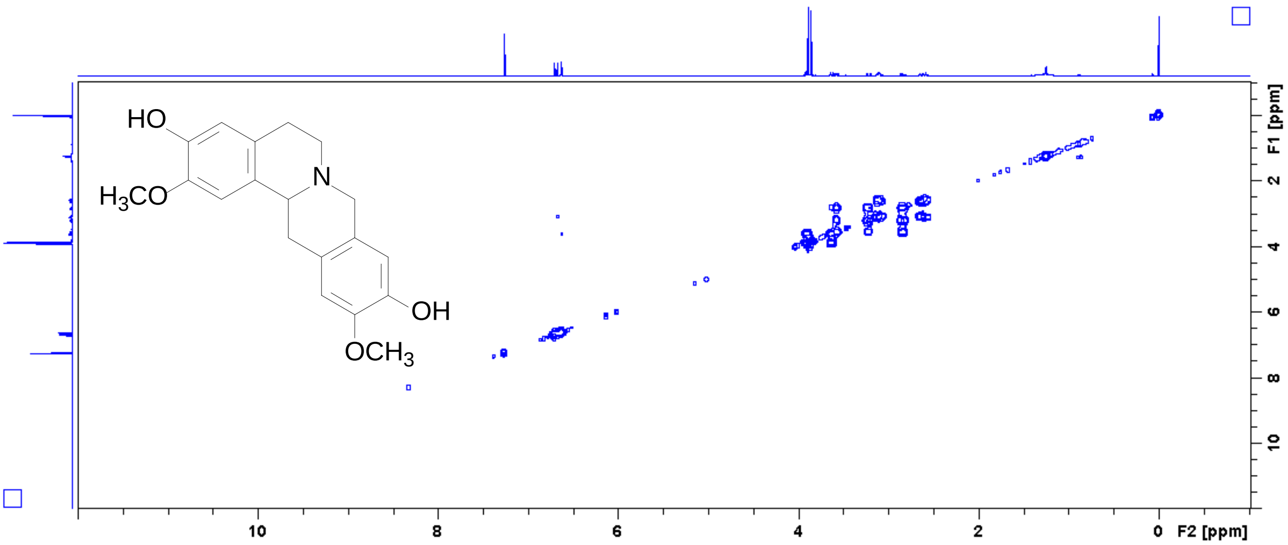

Figure S263. ^{1}H–^{1}H COSY correlation map of **10-demethyldiscretine** (**18**) recorded in $CDCl_3$ with a drop of CD_3OD at 500 MHz.

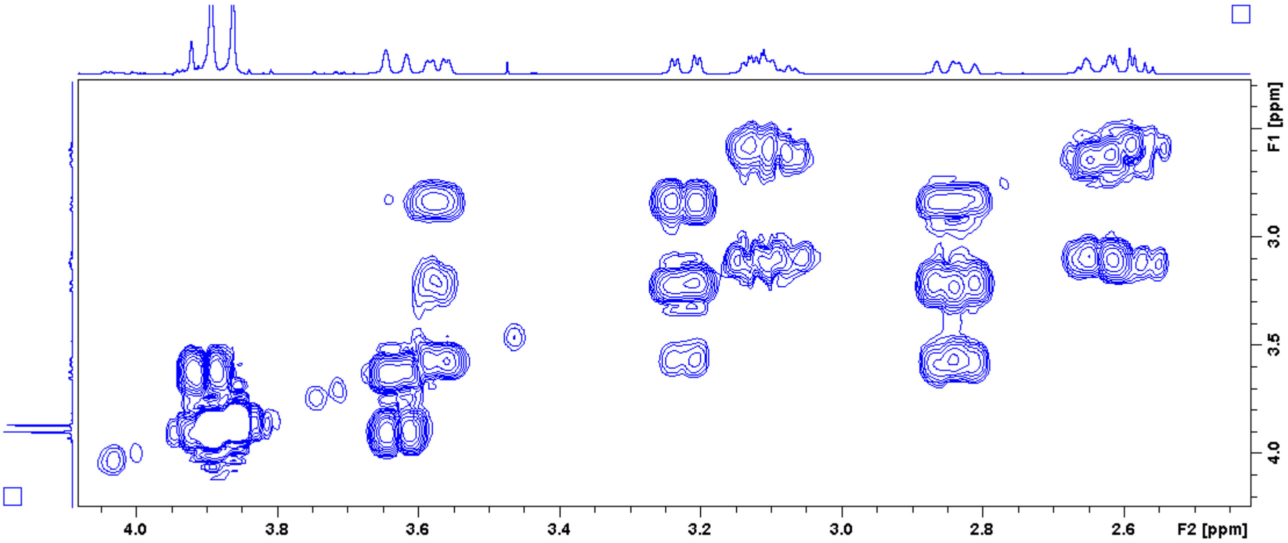

Figure S264. Expanded view 1 of the ^{1}H–^{1}H COSY correlation map of **10-demethyldiscretine** (**18**) recorded in $CDCl_3$ with a drop of CD_3OD at 500 MHz.

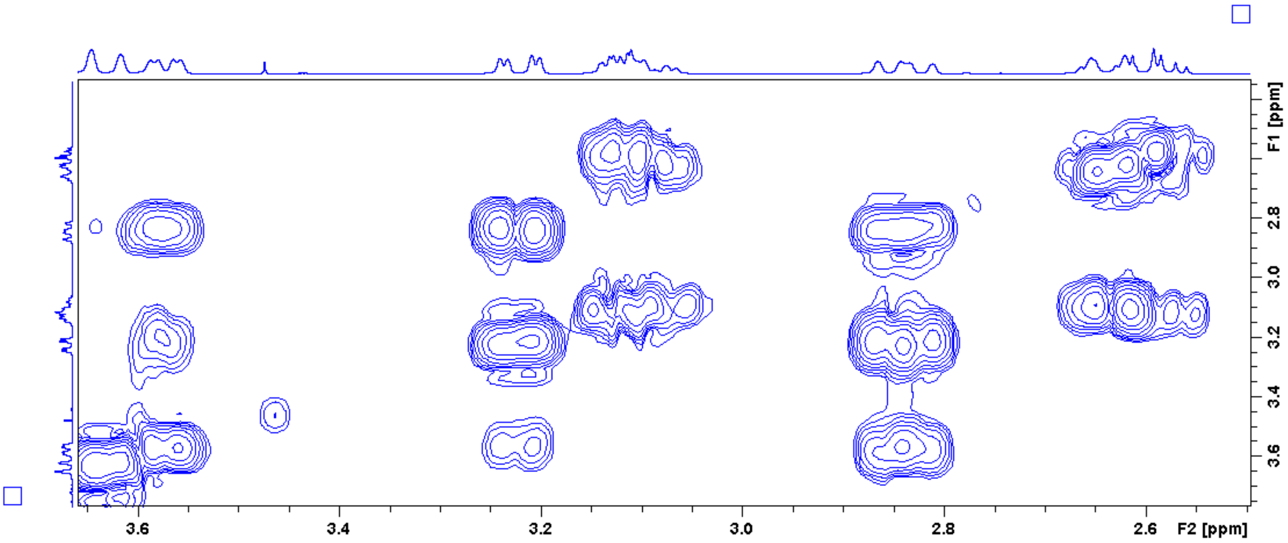

Figure S265. Expanded view 2 of the ^{1}H–^{1}H COSY correlation map of **10-demethyldiscretine** (**18**) recorded in $CDCl_3$ with a drop of CD_3OD at 500 MHz.

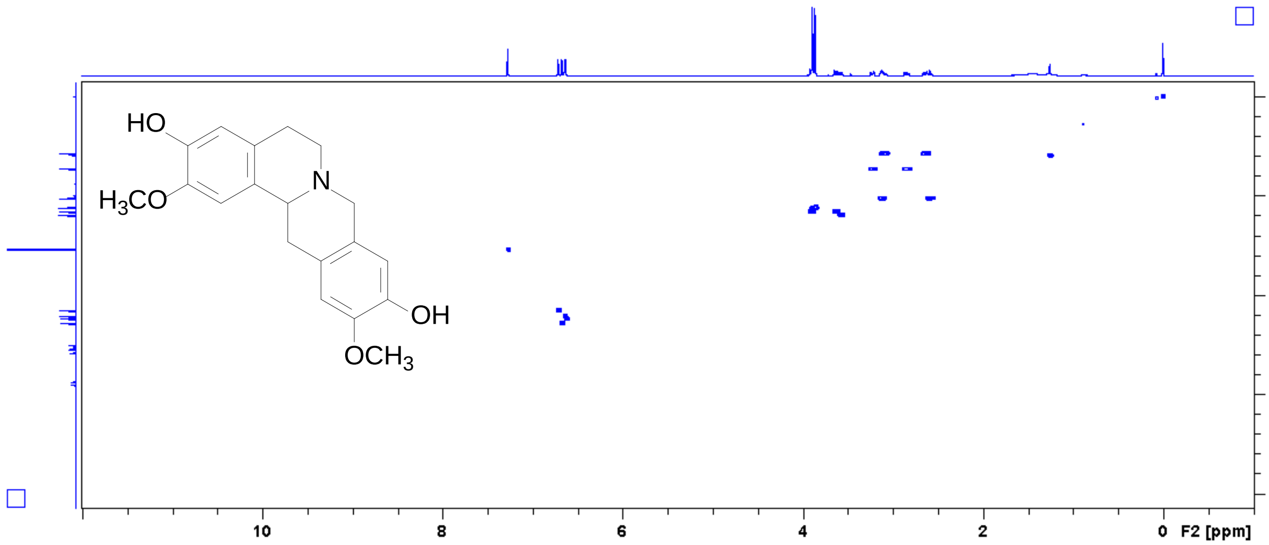

Figure S266. One-bond $^1H–^{13}C$ HSQC correlation map of **10-demethyldiscretine** (**18**) recorded in $CDCl_3$ with a drop of CD_3OD at 500 MHz (^1H) and 125 MHz (^{13}C).

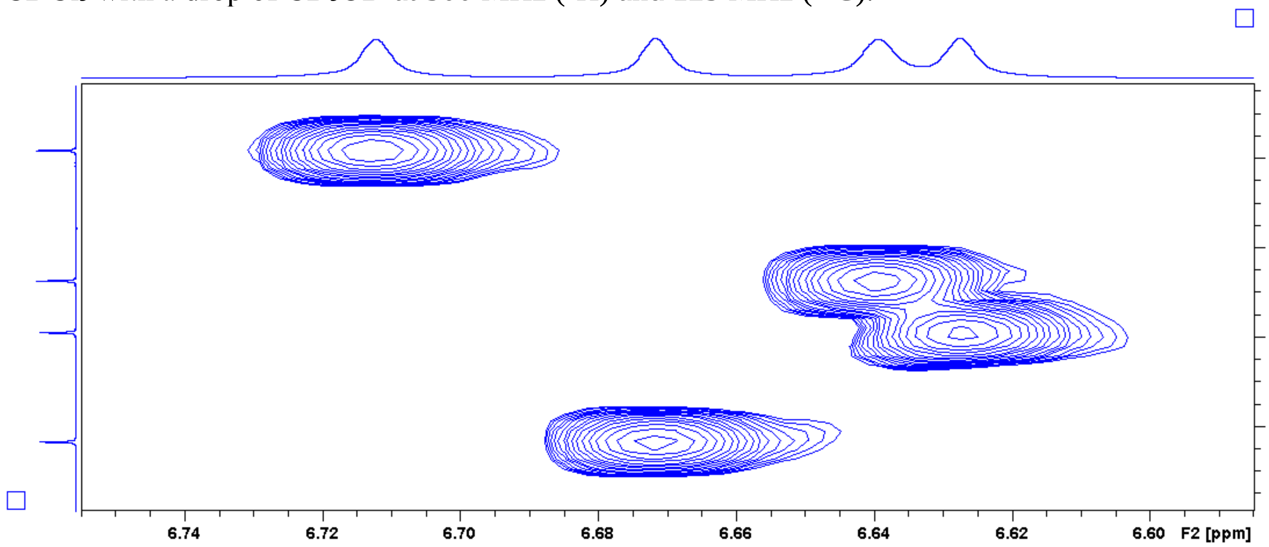

Figure S267. Expanded view 1 of the one-bond $^1H–^{13}C$ HSQC correlation map of **10-demethyldiscretine** (**18**) recorded in $CDCl_3$ with a drop of CD_3OD at 500 MHz (^1H) and 125 MHz (^{13}C).

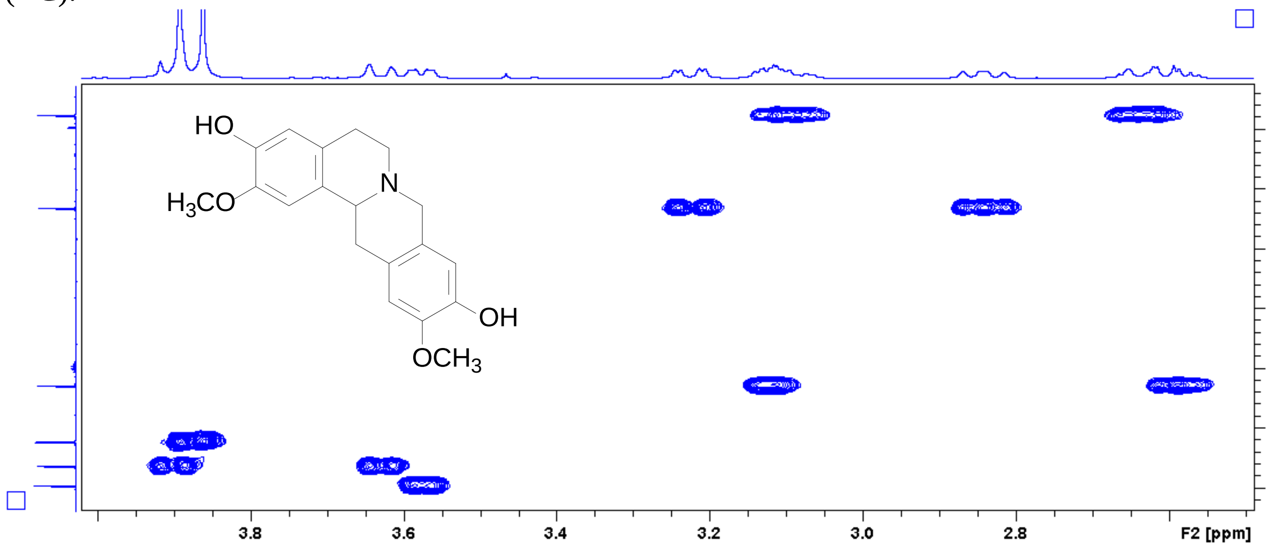

Figure S268. Expanded view 2 of the one-bond $^1H–^{13}C$ HSQC correlation of **10-demethyldiscretine** (**18**) recorded in $CDCl_3$ with a drop of CD_3OD at 500 MHz (^1H) and 125 MHz (^{13}C).

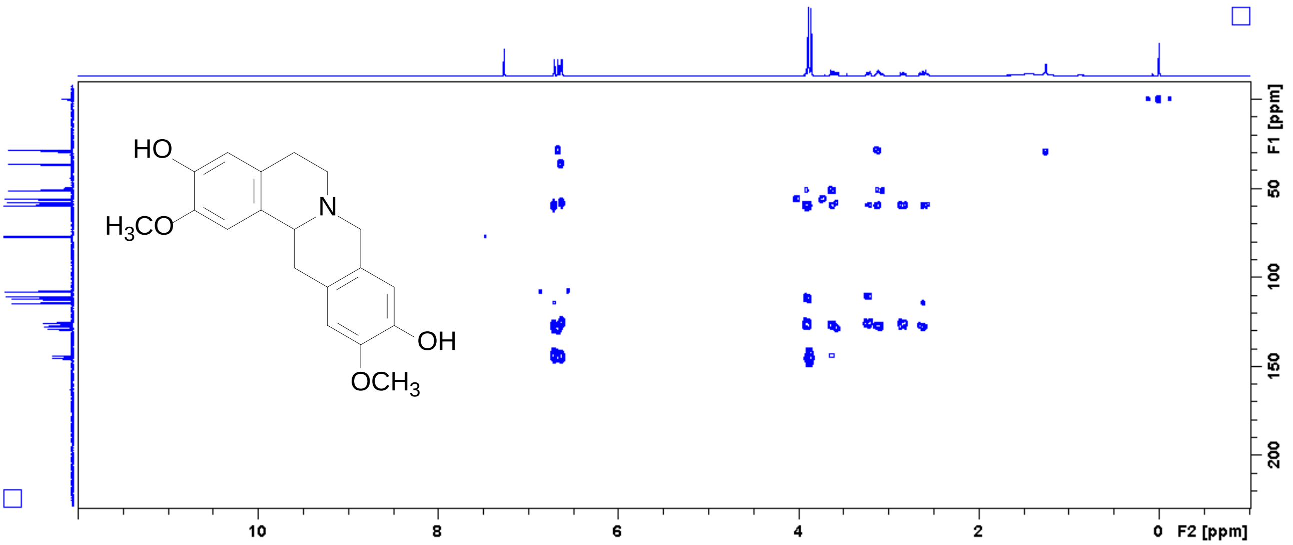

Figure S269. Long-range ^{1}H–^{13}C HMBC correlation map of **10-demethyldiscretine** (**18**) recorded in $CDCl_3$ with a drop of CD_3OD at 500 MHz (^{1}H) and 125 MHz (^{13}C).

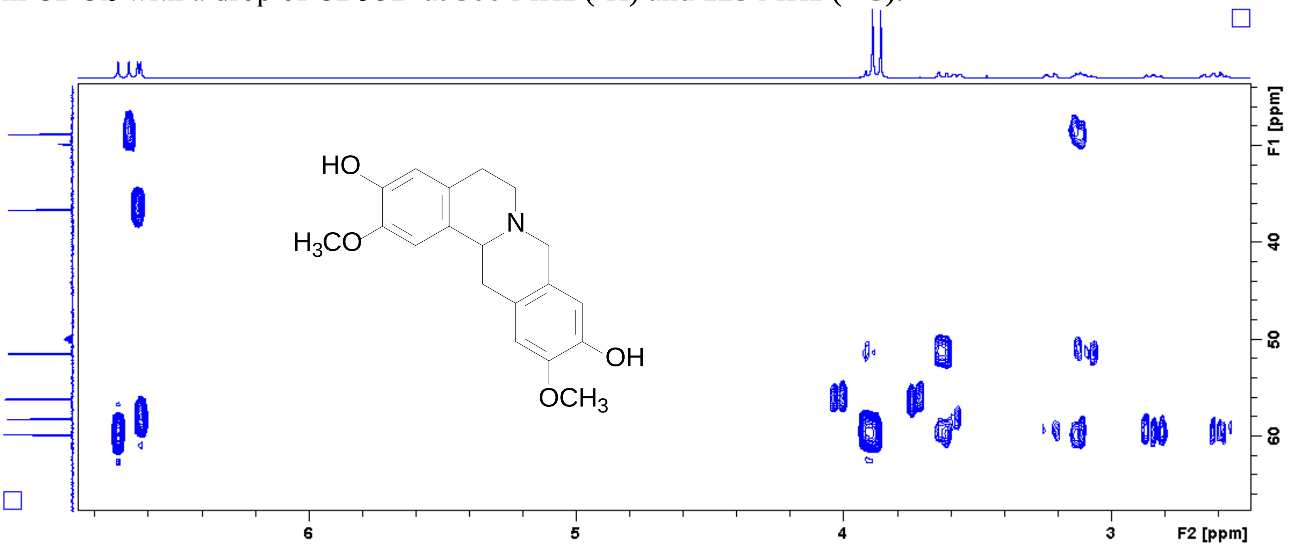

Figure S270. Expanded view 1 of the long-range ^{1}H–^{13}C HMBC correlation map of **10-demethyldiscretine** (**18**) recorded in $CDCl_3$ with a drop of CD_3OD at 500 MHz (^{1}H) and 125 MHz (^{13}C).

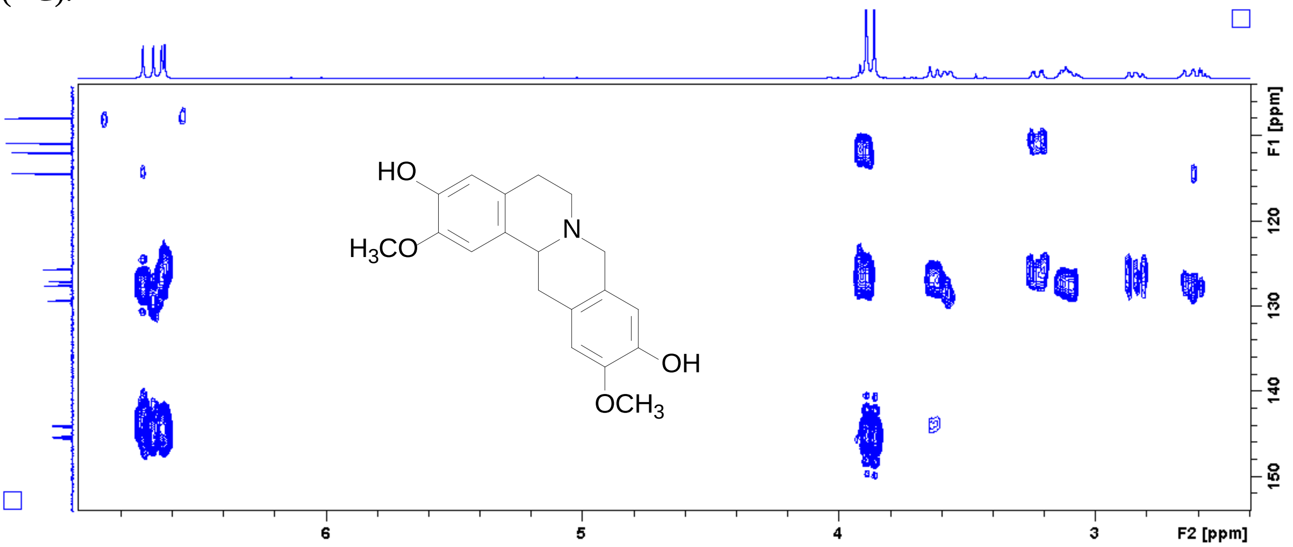

Figure S271. Expanded view 2 of the long-range ^{1}H–^{13}C HMBC correlation map of **10-demethyldiscretine** (**18**) recorded in $CDCl_3$ with a drop of CD_3OD at 500 MHz (^{1}H) and 125 MHz (^{13}C).

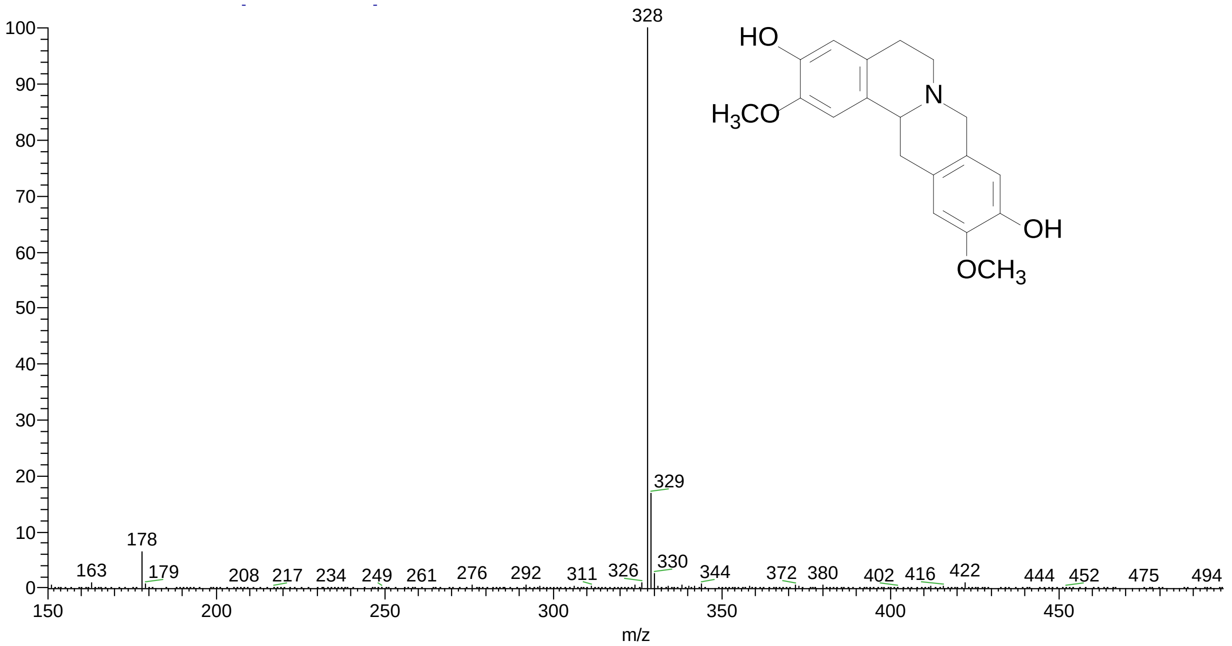

Figure S272. LR-APCI(+)-MS spectrum of **10-demethyldiscretine** (**18**), showing the protonated molecular ion at *m/z* 328 $[M + H]^+$.

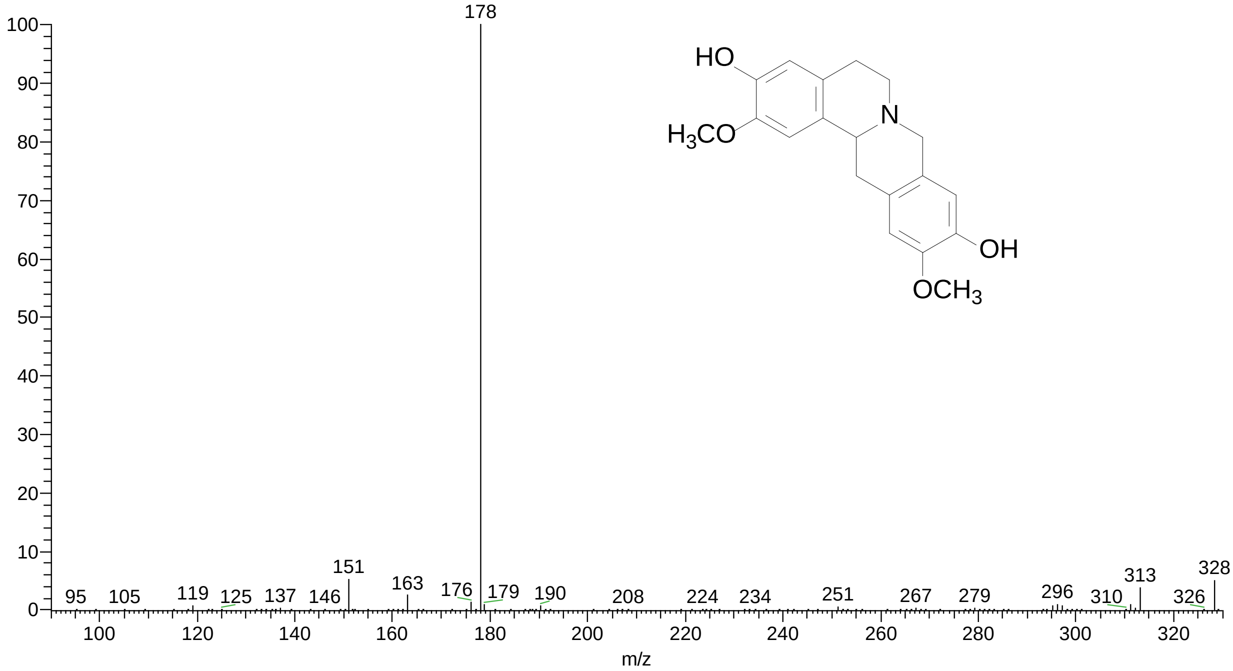

Figure S273. LR-APCI(+)-MS/MS spectrum of the protonated molecule of **10-demethyldiscretine** (**18**) at *m/z* 328 $[M + H]^+$.
